# BSim: An Agent-Based Tool for Modeling Bacterial Populations in Systems and Synthetic Biology

**DOI:** 10.1371/journal.pone.0042790

**Published:** 2012-08-24

**Authors:** Thomas E. Gorochowski, Antoni Matyjaszkiewicz, Thomas Todd, Neeraj Oak, Kira Kowalska, Stephen Reid, Krasimira T. Tsaneva-Atanasova, Nigel J. Savery, Claire S. Grierson, Mario di Bernardo

**Affiliations:** 1 Bristol Centre for Complexity Sciences, Department of Engineering Mathematics, University of Bristol, Bristol, United Kingdom; 2 Department of Engineering Mathematics, University of Bristol, Bristol, United Kingdom; 3 School of Biochemistry, Medical Sciences, University Walk, Bristol, United Kingdom; 4 School of Biological Sciences, University of Bristol, Bristol, United Kingdom; 5 Department of Systems and Computer Engineering, University of Naples Federico II, Napoli, Italy; Mount Sinai School of Medicine, United States of America

## Abstract

Large-scale collective behaviors such as synchronization and coordination spontaneously arise in many bacterial populations. With systems biology attempting to understand these phenomena, and synthetic biology opening up the possibility of engineering them for our own benefit, there is growing interest in how bacterial populations are best modeled. Here we introduce BSim, a highly flexible agent-based computational tool for analyzing the relationships between single-cell dynamics and population level features. BSim includes reference implementations of many bacterial traits to enable the quick development of new models partially built from existing ones. Unlike existing modeling tools, BSim fully considers spatial aspects of a model allowing for the description of intricate micro-scale structures, enabling the modeling of bacterial behavior in more realistic three-dimensional, complex environments. The new opportunities that BSim opens are illustrated through several diverse examples covering: spatial multicellular computing, modeling complex environments, population dynamics of the *lac* operon, and the synchronization of genetic oscillators. BSim is open source software that is freely available from http://bsim-bccs.sf.net and distributed under the Open Source Initiative (OSI) recognized MIT license. Developer documentation and a wide range of example simulations are also available from the website. BSim requires Java version 1.6 or higher.

## Introduction

Systems and synthetic biology rely on mathematical modeling and computational simulation to predict the behavior of biological systems and facilitate the design of novel systems [1]. As it is unfeasible to test every possible hypothesis experimentally, modeling and simulation can reduce time consuming lab work, investigate functional properties and limits and analyze system robustness [2], [3]. So far, much of the modeling in systems and synthetic biology has focused on intracellular dynamics; describing how the concentrations of key chemicals, mRNAs and proteins vary over time within a single cell. These sorts of model are essential to understand the actions of individual cells. However, many interesting behaviors occur at the population level e.g., bacterial coordination [4], communication [5] and cooperative growth [6] are often important during infection [7]. To better understand how these mechanisms work it is necessary to consider not only the intracellular dynamics, but also the interactions between individual cells in the population and those between cells and their shared environments.

A common method to capture how low-level interactions between cells give rise to population level behaviors is to use agent-based or individual-based models (AbMs, IbMs). These consider populations of autonomous agents, each following a set of internal rules and interacting with each other within a shared virtual environment. Unlike modeling approaches that consider only a single level of representation, agent-based models enable an understanding of the relationship between microscopic rules of the agents and macroscopic behaviors of the population. A further benefit of agent-based models is that they are able to incorporate heterogeneity due to both spatial features of an environment and differences between individual agents. This is particularly relevant in biology, where cellular and environmental heterogeneity is unavoidable due to the intrinsic noise of biochemical processes and the complexity of natural environments.

A wide variety of agent-based frameworks are currently available. Each of these provides a different set of features and make alternative tradeoffs in order to allow for the efficient simulation of particular types of system. Highly extendable frameworks such as NetLogo [8] and FLAME [9] provide a bare minimum of functions with the intention that a user can easily add the functionality they require. In principle this helps to simplify using the framework and broadens the possible applications. However, in the context of bacterial systems this general approach is often not well suited. Firstly, standard bacterial traits are often common to many models (e.g. chemotaxis), however, a user may not necessarily wish to think about the intricacies of these processes directly or have the time and desire to implement them from scratch. Secondly, these frameworks do not consider the realistic environmental physics necessary for capturing bacterial movement and the interactions that can take place within complex small-scale environments. Simulating these features is far removed from many of the biological questions being asked and by not providing them as core tested features the use of these tools is hampered. More specialized approaches have been proposed such as CompuCell3D [10] and Chaste [11]. While these partially fulfill some of these shortcomings, e.g., accurately modeling cellular dynamics and environmental physics, because their attention is instead directed towards the simulation of dense tissue like systems, the computational representations used do not allow for the efficient simulation of bacterial populations that become spare or migrate large distances over time.

The focus of agent-based approaches for studying bacterial populations has so far been to develop models that can accurately replicate known results and to understand how these are affected by heterogeneity within a bacterial population. One of the first studies in this area was by Kreft *et al.* looking at biofilm formation and growth [12], [13]. This work resulted in the development of BacSim [12], an agent-based model which considered substrate uptake, metabolism, maintenance, division and growth of individual cells, and a simple two-dimensional lattice model for substrate diffusion within the shared environment. Comparisons were made with an established biomass-based model [14] and it was shown that the agent-based approach was in good agreement with overall growth characteristics. This approach has since been extended to consider a simple three-dimensional environment and model the effect of various physical and biological factors on biofilm formation [15]–[17]. Furthermore, Fozard *et al.* have used agent-based models of bio-films to test possible treatments based on quorum-sensing inhibition [18].

More recently, agent-based models have been employed to study bacterial chemotaxis. Emonet *et al.* developed AgentCell [19] to analyze how stochastic intracellular events affected cellular motility. An accurate stochastic model of the biochemical reactions taking place within each cell was used. By coupling the cell models to environmental properties, such as chemoattractant gradients, the resulting agent-based model was able to reproduce many of the features seen in experimental results, both at the single cell and population levels.

Whilst these models have been successful in replicating experimental results, they are also of great use in synthetic biology where the ability to manipulate a population of bacteria opens up many new opportunities [20], [21]. Tamsir *et al.* [20] illustrated this by showing that spatially arranged populations of engineered bacteria could act as logic gates, and when wired together using chemical signaling were able to compute the solution of a logic function. By using populations instead of individual cells, errors due to environmental noise or faulty cells could be managed effectively. This is common in nature where processes that are unreliable and error prone for individual cells are transformed to become highly accurate and resilient by a population working in unison e.g., joint responses through quorum sensing [4] and the synchronization of rhythmic processes [22].

A major obstacle when using existing modeling frameworks to describe other features of bacterial populations is their specificity. Previous approaches have focused on aspects of behavior most relevant to the problem being studied e.g., bio-mass production or the chemotaxis biochemical regulatory network. These specific descriptions do not permit the necessary customization for studying variances due to diseased cell states, allow for the consideration of purely synthetic regulatory networks driving dynamics, or new types of entity that might play an important role e.g., outer membrane vesicles. This has resulted in tools that are inflexible to new requirements, hampering the sharing of knowledge and reuse of work across communities. Furthermore, whilst spatial aspects have been partially considered, no existing framework allows for a full description of complex spatial structures, including their influence on other features of a model such as chemical diffusion or bacterial motility. Therefore, any effects these might have are neglected.

With the aim of meeting these shortcomings we present BSim, a modeling tool designed to act as a common framework for building and characterizing agent based models of bacterial populations. BSim builds on previous work to: (i) save time and effort by allowing for the reuse of many built-in bacterial traits; (ii) enable an accurate description of complex micro structures and environments through user defined meshes; (iii) allow for multiple levels of detail through coarse graining from individual agents to continuous fields; (iv) provide realistic bacterial dynamics through simulated gene regulatory networks in the form of ordinary differential equations (ODEs); (v) enable its functionality to be easily customized or extended through the use of object-orientated programming techniques, and (vi) be easy to learn and use through a wide range of example simulations. Unlike the general frameworks of NetLogo [8] and FLAME [9] we support the user with a suite of targeted core functionality that reduces the time and effort necessary to create working bacterial simulations and allow for the inclusion of common mathematical models that describe the internal dynamics of individual cells. Furthermore, an underlying particle based representation allows for efficient simulation of bacterial interactions in complex environments, alleviating the problems experienced by tools such as CompuCell3D [10].

## Design and Implementation

BSim is a highly customizable agent-based modeling tool that allows for the study of how population level behaviors arise from the dynamics of individual cells. BSim has been designed with the principle that, while important biological functions should have accurate built-in reference implementations, all aspects of a model should be able to be updated, extended or replaced by user defined versions. This approach permits the rapid development of simulations that capture the main characteristics of a system, while allowing for these to be refined as further experimental data becomes available.

Models in BSim take the form of Java programs and can be developed using any standard text editor or software development suite. To allow for the modeling and simulation of physical processes in complex spatial environments, data structures such as octrees have been used to efficiently store multi-level representations (Figures S1, S2 and Text S1). Additional libraries have also been used to assist with the creation and rendering of three-dimensional objects during simulation (Text S1).

**Figure 1:**
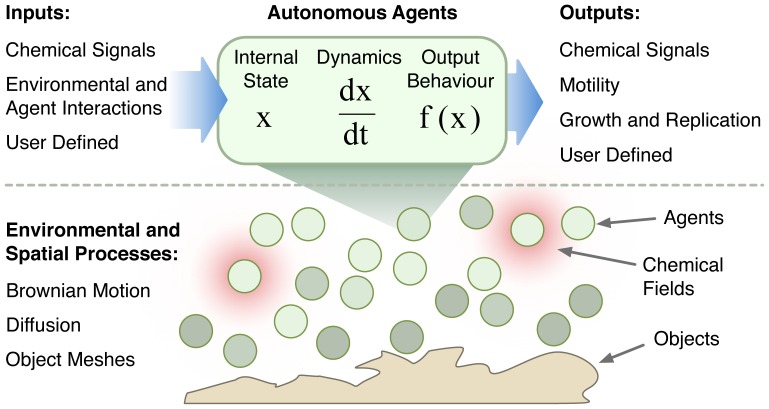
Schematic of a BSim model. BSim models consist of two main levels: 1. the individual agent (top) and 2. the shared environment (bottom). Individual agents are used to model any autonomous entity, such as a bacterium, outer membrane vesicle, etc, and contain an internal state vector which can change over time. BSim provides support for ordinary differential equations or user defined rules when specifying agent dynamics. Agents can sense various environmental factors as inputs and generate outputs within the local environment. The environment provides a shared medium in which agents can move, communicate (using chemical signaling), interact (through physical contact) with other agents or objects, and can be detailed and heterogeneous.

BSim began life as part of the BCCS-Bristol team entry into the 2008–10 iGEM competition (Text S1), but has since been developed to provide a wide array of functionality for modeling bacterial populations. The main features of a BSim model are shown in Figure 1 and detailed in what follows. Key features are illustrated by case-studies highlighting the potential of the modeling platform.

## Results

### Spatial Interactions and Processes

Simulations in BSim take place within a three-dimensional fluid filled environment. The extent of this environment is defined by a rectangular cuboid and aspects of the fluid such as viscosity and temperature can be altered to match experimental conditions. Environmental boundaries can be defined as solid (reflecting), physically constraining movement, or wrapping (periodic), to allow for the approximation of a larger space where little heterogeneity is observed. Wrapping boundaries cause any objects moving through an edge to be placed at the same relative location on the opposite side of the environment. It is assumed all objects are small enough that their momentum within the fluid is negligible [23], [24] i.e., the system has a very small Reynolds number. This is a realistic assumption to make for bacterial systems and allows the use of Stokes' law when calculating the movement of an object experiencing a force.

The importance of including spatial features in models has previously been highlighted by Durrett *et al.* [25] and Tilman *et al.* [26] and is clear to see in the work of Tamsir *et al.* [20], where several spatially separated populations of engineered bacteria were used to calculate a logic function. Each population comprised of bacteria containing the same genetic construct, encoding a basic logic gate. The specific type of gate varied between populations, dependent on the overall function that needed to be calculated. To connect these simple gates together and perform more complex calculations, bacteria were also designed to sense and emit different types of chemical which acted as “chemical wires” by diffusing through the environment and enabling communication between specific populations.

**Figure 2:**
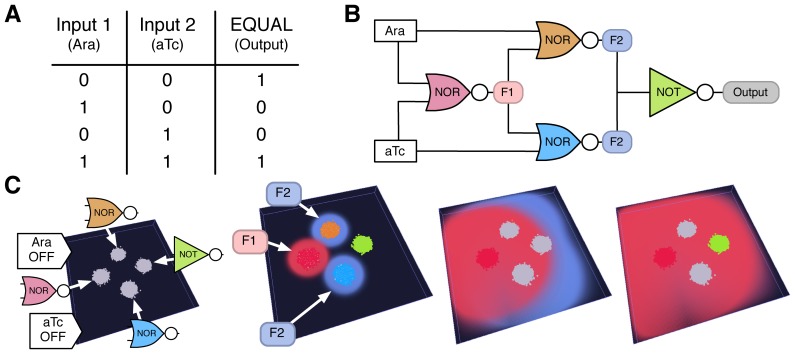
Computing with synthetic bacterial populations. Recreation of experimental results from [20] that implement the EQUAL boolean function. Each population implements a logic gate with activation of bacteria shown by color intensity and different colors being used for each population. The environment consists of a flat surface 150
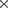
150
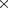
12 microns in size with each population containing 20000 bacteria. Inputs are in the form of constant chemical fields throughout the environment, specifically arabinose (Ara) and anhydrotetracycline (aTc). A) Truth table of the EQUAL function. B) Circuit used to implement the EQUAL function. C) Left to right, simulation output when both Ara and aTc are not present. The logic gates implemented by each population and the chemical fields used to connect these together have been highlighted. Notice the several stages of activation and inactivation that occur before the system reaches the correct steady-state value, shown by activation of the rightmost population. Also see Video S1.

To illustrate BSim's virtual environment and spatial processes such as chemical diffusion, we modeled the experimental results from [20] *in-silico* (Text S1, Figure S3 and Video S1). To capture the bacterial dynamics a delayed Boolean rule was implemented in each agent, representing the logic gate construct present. Heterogeneity between bacteria was incorporated through varying delays that represented (i) the time to reach a fully active state when activated (related to protein production), and (ii) the time to become inactive when an active state becomes repressed (related to protein degradation). Realistic delays were chosen for the majority of simulations (Text S1). However, we also assessed the robustness of the system by varying the mean delay times to include non-physical values. In all cases, simulations generated the correct steady-state output (Figures 2, S4, S5, S6, S7 and Text S1), illustrating that simple bacterium dynamics, population averaging and spatial separation are viable approaches for improving system robustness.

### Gene Regulatory Networks Driving Agent Dynamics

Much research effort in systems and synthetic biology has been devoted to understanding and designing gene regulatory networks (GRNs). Due to their widespread use and ability to drive bacterial dynamics we allow for GRNs to be modeled within BSim as systems of coupled ordinary differential equations (ODEs). GRNs can be incorporated in any existing agent and can interact with the local environment through the excretion or sensing of chemicals, or direct contact with other agents or objects.

To simulate the dynamics of these ODE systems each agent is provided with an independent built-in fourth order Runge-Kutta solver, allowing for accurate and robust integration over the time scales present in the majority of GRNs. Solvers associated with different agents can run independently, enabling efficient separation of time scales where required i.e., solvers can have different time steps reducing the need for bacteria exhibiting slow dynamics (large time step) to be updated at the same frequency as those experiencing fast dynamics (short time step). Furthermore, the open manner in which ODEs are represented enables the future inclusion of more advanced solver types e.g., to simulate noisy processes possibly represented by stochastic ODEs or rule based dynamics [27].

**Figure 3:**
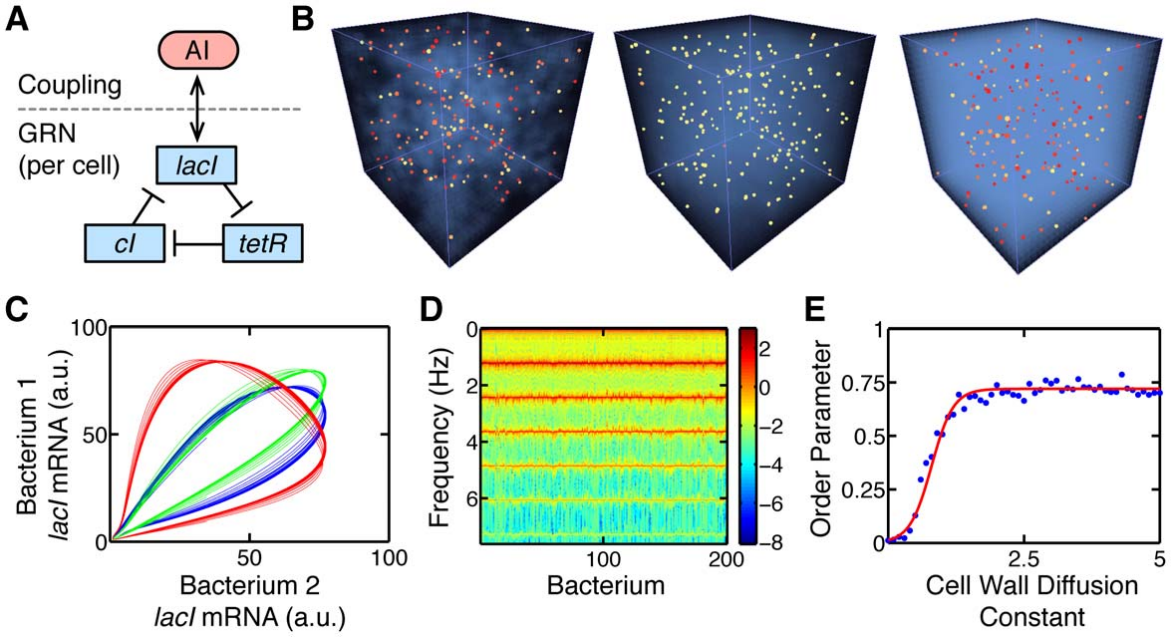
Synchronized genetic oscillators. Results from studying the synchronization of a population of 200 bacteria, each containing a repressilator GRN model [28]. A) Repressilator GRN with external coupling. B–D) Simulations performed with a chemical field diffusivity of 100 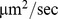 and a cell wall diffusion constant of 1 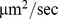. B) Left to right, simulation output for times 0, 5.5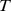 and 40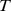, where 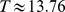 mins is the GRN period of oscillation. The color of the bacteria corresponds to their internal level of *lacI* mRNA, yellow for low and red for high. External autoinducer level is represented by the intensity of the blue field surrounding the bacteria. Initial mRNA and protein levels for each bacterium were chosen at random. However, synchronization quickly increases over time. Also see Video S2. C) Phase portraits for 3 pairs of bacteria. For clarity the first 2.5 hours of data, where the bacteria were extremely asynchronous, are omitted. Over time, each pair becomes more synchronized. D) Amplitude spectra for all bacteria with colors representing 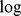
(amplitude) in arbitrary units (a.u.). The clear peaks correspond to the fundamental frequencies of the GRN where phase locked synchronization has occurred. E) Phase transition to synchronization as the cell wall diffusion constant is increased.

Most existing work related to GRNs has focused on intracellular dynamics. Much less is known about how such networks might interact and behave across a population. BSim opens up the opportunity to investigate this question, helping to understand the effects spatial positioning, the diffusion of intracellular signals, and bacterial heterogeneity might have. To illustrate how these capabilities can be used in BSim, the study of Garcia-Ojalvo *et al.* [28] was used as a case study. Specifically, a population of bacteria containing an oscillatory GRN was considered. This GRN was a coupled version of the repressilator first introduced in [29], and contains three genes in an inhibitory feedback loop with each gene repressing the next in sequence (Figure 3A). To allow for coupling between cells, autoinducer (AI) signaling molecules are generated by the GRN and influence its dynamics. Unlike other gene products, the AI is allowed to diffuse out of the cell and into the wider environment. A high-dimensional system of nonlinear ODEs was used to model this system without considering the possible effects of spatial heterogeneity [28]. This analysis showed that communication via the AI enabled a diverse population to become robustly self-synchronized.

By using a diffusive AI as a form of communication, it is likely spatial features will affect the GRN dynamics exhibited by any physical implementation of this system. To explore this possibility a BSim model was developed where bacterial agents were equipped with the GRN previously described (Text S1). We assessed how the cell wall diffusion constant and chemical diffusivity of the AI affected synchronization of the population.

Simulation results showed that for a chemical field diffusivity of 100 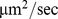 and a cell wall diffusion constant of 1 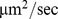 (physically realistic parameters), clear population level synchronization was obtained (Figure 3 and Video S2). Furthermore, moving from low to high cell wall diffusion constants saw a phase transition as a value of 0.9 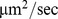 was exceeded (Figure 3E), a feature also seen previously in [28]. To assess the robustness of this phase transition to other model parameters, several supplementary simulations were run varying the diffusivity of the chemical field and mRNA decay rates. In all cases, a similar transition was present.

One difference between the previous model and BSim was the overall maximum synchronization achieved. We found a reduced fraction of synchronized bacteria, with an order parameter 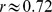 (Figure 3E) as opposed to 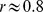 previously reported in [28]. This difference is likely attributable to the spatial aspects of our simulations which add varying delays when communicating via a diffusive medium to randomly moving bacteria. While further investigation is required to verify this conjecture, it does highlight a possible constraint neglected by previous approaches and suggest possible limitations in the size and shape of a population that can exhibit high synchrony. This is supported by recent experimental results that show the emergence of traveling waves for populations of coupled genetic oscillators in microfludic chambers with particular geometries [30].

### Complex Micro-Scale Structures

In many situations bacteria encounter and interact with complex structures at scales of a similar order of magnitude. For example, bacteria growing on heart valves are surrounded by collagenous fibrils 1–3 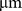 across [31]. Unlike many existing models that ignore the effect these interactions might have, BSim allows for micro-scale structures to be represented as generic meshes. These can be generated by code or imported from files stored in the popular and standardized Wavefront OBJ format. Meshes define an arbitrarily shaped region in space and can be used in many different ways. Collision detection can be used to define intricate boundary geometries and spatially varying environmental or behavioral parameters. Meshes can be used at varying levels of detail to minimize computation during simulation.

In synthetic biology, modeling complex structures at the micro-scale level is becoming more important as techniques such as microfluidics gain popularity. Although most existing microfluidic chambers have fairly simple geometries, it has been shown that alternative designs can lead to differing population level dynamics. For example, Danino *et al.* in [30] showed the existence of both population level synchronization and traveling wave dynamics depending on chamber size and design. Furthermore, systems biology faces the need to model intricate structures such as spatial features of the natural environment in which a bacterial population lives.

**Figure 4:**
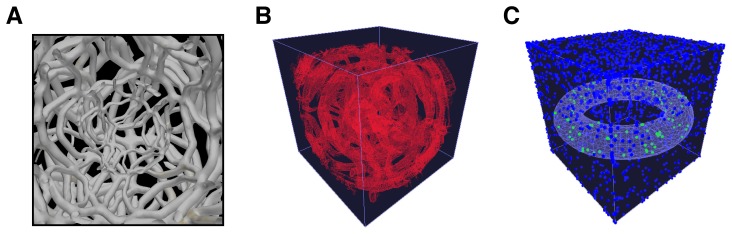
Describing complex spatial environments with meshes. A) User generated mesh that could be used to approximate a fibrous matrix, similar to that found in cotton wool. B) The same mesh loaded into a BSim environment. C) Torus shaped mesh used to influence behavioral characteristics of the bacteria. In this case, altering the output color they emit (blue outside and green inside the mesh). Also see Video S3.

To show how micro-scale structures can be used within BSim, we constructed several example meshes to constrain motility and influence the behavior of bacterial agents (Figure 4, Text S1 and Video S3). The first of these represents a fibrous material that could be used to approximate an environment that may arise in areas such as wound healing (Figures 4A and 4B). Simulations of random bacterial movement both unconstrained and when interacting with this mesh showed significant difference in the overall motility achieved. Finally, a simpler structure in the form of a torus was chosen to show how meshes can be used to modify behavioral characteristics (Figure 4C), helpful for defining spatially varying inputs e.g., light sources.

While here we have manually generated the mesh structures, we envisage that more realistic approximations can be produced directly from real data. For example, by using micrographs and image recognition software it would be possible to automatically extract structural details of an experiment and build a realistic mesh to represent these features.

### Modeling Effects at Multiple Levels

BSim has been designed to allow users to take multiple levels of detail and scale into account throughout modeling and simulation. To illustrate this feature we consider the process of chemical diffusion. For very low concentrations of chemicals, agents can be used to represent the individual chemical molecules that diffuse randomly due to Brownian forces. Modeling at this level enables the stochastic nature of the process to be fully captured, but requires large computational resources if the number of molecules grows. For larger numbers of molecules a continuous approximation can be made, modeling diffusion at the level of concentration. Less intensive numerical methods, such as finite-difference, can then be used to capture the more deterministic nature of the process, removing the need to simulate individual agents. Both of these schemes are available in BSim and the ability to tune the level of representation of specific aspects of a model is a powerful way of ensuring essential characteristics of a process are captured, while reducing the computational burden during simulation. Due to possible interdependencies between various types of agent and physical process, the choice of level of representation for various aspects of a model must be defined by the user when specifying a simulation and remains fixed during execution.

**Figure 5:**
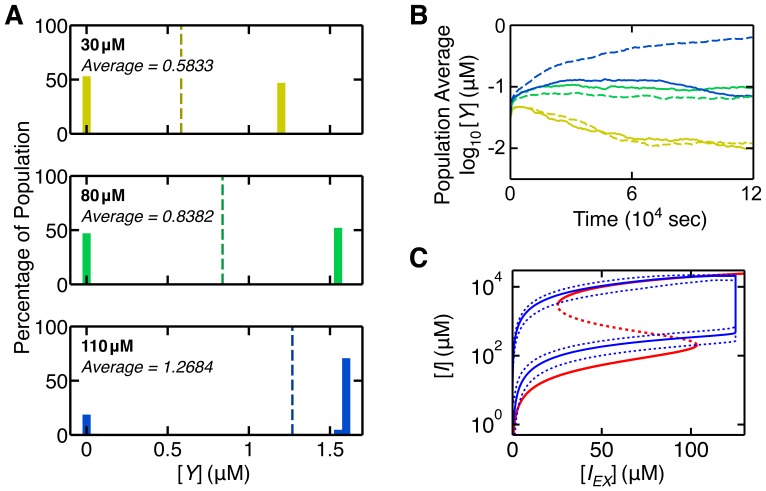
Multi-level effects of the *lac* operon. Results from a model of the *lac* operon that considers the states of individual cells as well as the population as a whole. A) Bimodal state distributions including the external inducer concentration and level of *lac* permease, 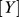. Since the model does not explicitly include an indicator, 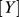 was used as a proxy measure. Low and high external inducer concentrations bias the population toward an uninduced or induced state respectively, and all concentrations see coexistence of states in the form of a bimodal distribution of 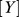. Dashed line indicates the overall population induction (average) that would be measured by purely observing at the population level, i.e., not taking into account the bimodal distribution of individual states. B) Effect of growth rate on coexistence of induced and uninduced states within the population. Line color indicates external inducer concentration, 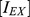, (yellow = 30 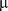
M, green = 80 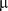
M, blue = 110 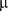
M,), solid and dashed lines indicate simulations where induction did and did not inhibit growth respectively. C) Bifurcation diagram showing bistability in the intracellular inducer concentration, 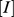. Red line illustrates the equilibrium state of 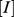 in the deterministic GRN equations for a single cell as a function of external inducer, 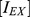, computed via numerical continuation (solid and dashed lines indicate stable and unstable equilibrium respectively); blue line illustrates ensemble average 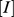 concentration in a BSim simulation which incorporates this deterministic GRN and stochastic agent creation and removal in which 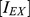 was slowly varied (dashed lines indicate population minimum and maximum).

Another important area where multiple levels naturally arise is when the dynamics of individual agents lead to emergent features of the population as a whole. This is clearly shown in studies of induction of the *E.coli lac* operon [32], [33]. Addition of low concentrations of inducer causes a smooth increase in the level of *lac* operon expression when measured at a population level. However, more detailed analysis reveals that this induction results not from a uniform increase in *lac* operon expression in all members of the population, but from changes in an underlying bimodal distribution of individual bacterial states, with each bacterium being classed as either “induced” or “uninduced”. The importance of bistable behavior at the level of individual bacteria, but an overall smooth population output, makes this real-world system an ideal candidate for BSim's agent-based approach. We developed a BSim model where each bacterium implemented an ODE model representing the *lac* operon GRN from [34] (Text S1 and Table S2). Effects of external inducer on the system were incorporated via a diffusing chemical field, with interaction between this and each individual bacterium fed as an input to its internal GRN. Because of the comparatively large time scale of the process, in the order of hours, an additional factor in the form of probabilistic bacterial replication was included. Our key results are summarized in Figure 5.

We investigated two crucial aspects of the *lac* operon's behavior that have been observed experimentally, but are difficult to model using existing techniques such as ODEs due to the requirement for multiple levels of description, from individual bacterium to populations, and the need to include other biological processes such as cell replication. Specifically, we considered the bimodal distribution of the bacterial induction states [32], and changes in the ability of the two bacterial states to coexist resulting from induction affecting growth rate [33]. As expected, our model exhibited an increase in the average level of *lac* operon expression as inducer concentration increased. In agreement with experimental data [32], the gradual induction observed at the population level was due to a change in a bimodal distribution of uninduced and induced individuals, rather than to a gradual and uniform increase in the expression levels in all individuals (Figure 5A). This highlights the potential importance of measuring induction at the microscopic (bacterium) level as opposed to a macroscopic (population) average.

Finally, we tested the ability of our model to correctly predict the consequences of competition between the induced and uninduced sub-populations of bacteria. Gratuitous induction of the *lac* operon i.e., induction when there is no lactose available to support growth, causes induced cells to grow more slowly than uninduced cells, biasing the bimodal distribution of the population and leading to a drop in the average *lac* operon expression [33]. We compared BSim simulations in which induction carried a growth rate penalty with simulations in which the two populations had the same growth rate. In agreement with experimental observations [33], higher inducer concentrations lead to a lower population average *lac* operon expression when induction decreased growth rate (Figure 5B). This was again the result of an underlying bimodal distribution, but with an increased proportion of the population in an uninduced state.

### Extendible Agents Through a Modular Design

As our understanding of biology continually improves, it is important to be able to readily incorporate new insights into existing modeling tools. To meet this requirement, BSim has been designed so that, where possible, each independent element of a model is captured within a fully separable module (Table S1). Modules can be extended to include additional required functionality, or replaced with alternative implementations. This enables BSim to be easily customized and allows for historic models to readily make use of improved knowledge, through new modules, as they become available.

This modular design also extends to the agents that are used to represent all forms of autonomous entities e.g., bacteria, vesicles, proteins or even individual molecules. Rather than creating fully independent definitions for every type of agent, the concept of a ‘particle’ is used to capture the common properties shared by them all. Particles are assumed to be spherical in shape and have an associated size, position and orientation. Particles form the most basic type of agent, with new agents being created by inheriting these common attributes and extending specific functionality as necessary. This means that BSim can be extended by including new types of agent. These may implement features previously unseen in BSim but, because of a shared underlying particle representation, these agents can be incorporated and their physical behavior modeled correctly.

A further benefit of this approach is that families of agents can be created with increasingly specific characteristics. This allows for a standard bacterium agent to capture normal control-like behaviors, while engineered or mutated versions can be produced by extending and altering the dynamics of this agent appropriately (Text S1). This not only saves time during the development of new models, but also helps to increase quality through the reuse of previously tested functionality.

## Availability and Future Directions

BSim is open source software requiring Java version 1.6 or higher and is available for download from http://bsim-bccs.sf.net or as supporting material accompanying this paper (Software S1). Developer documentation and a wide range of example simulations are available from the website. BSim is distributed under the Open Source Initiative (OSI) recognized MIT license. It should be noted that while source code is freely available, access to the main repository is managed by a team of administrators. This allows for new submissions to the library are validated before being incorporated as part of the official BSim distribution. Furthermore, code versioning is performed to allow for changes to be tracked over time and the efficient rollback of errors that may be introduced between releases.

To summarize, BSim enables the quick development of models from built-in bacterial traits or through existing GRN models. A core set of functionality has been specifically implemented that is essential to modeling all types of bacterial system. This focused approach helps to reduce the effort required by users to create a working bacterial-based simulation and enables the incremental development of models with increasing levels of detail as additional core functionality is enabled. Furthermore, BSim allows for full consideration of spatial factors and opens the opportunity to examine multi-level phenomenon *in-silico* where physical single cell measurements are either difficult or infeasible. This is made possible by the agent-based architecture underlying the framework, that allows for virtual measurements to be taken at varying levels of detail.

While great progress has been made understanding the behavior of individual bacteria in isolation, it is still unclear how these results might be predictably scaled for larger multicellular systems. BSim enables the exploration of links between intracellular dynamics and population level features in a framework that simplifies the modeling of complex biological systems, and supports the design of synthetic biological circuits.

## Supporting Information

Text S1 **Further information about the development of the BSim software and models.**
(PDF)

Figure S1 **Schematic of an octree spatial representation.** (a) An octree allows for the representation of a three-dimensional volume with regions of varying resolution and is used to store the underlying structure over which chemical diffusion in BSim is calculated. Nodes in the data structure represent regions of space and children a further subdivision of the same region. (b) Boundaries are approximated using a recursive subdivision method. First, the lattice is divided into two regions by a mesh, represented as a dotted curve. Each square that contains a segment of the mesh is subdivided into four equal parts. This process continues recursively until a user specified number of subdivisions is reached, halting the process.
(TIF)

Figure S2 **Scaling characteristics of simulation time to number of bacterial agents.** Each data point represents the execution time of 10 minutes of simulation time for the synchronized genetic oscillators model with varying numbers of bacteria and model complexity. We see a linear growth rate with gradient approximately 0.03 secs/bacterium.
(TIF)

Figure S3 **BSim environmental geometry and population placement for the multicellular computing model.** Central positions of each population are shown in the form (x, y, z).
(TIF)

Figure S4 **Results** **for the EQUAL simulations.** (a) EQUAL truth table. (b) Circuit used to implement EQUAL in BSim. Each gate is represented as a population of bacteria. F1 and F2 are diffusive chemical fields used for communication between bacterial populations. Ara and aTc act as inputs to the circuit. (c) Circuit simulation results for all possible inputs. Each plot relates to a particular population from 1–4 (top–bottom) with colors related to the circuit diagram.
(TIF)

Figure S5 **Results** **for the XOR simulations.** (a) XOR truth table. (b) Circuit used to implement XOR in BSim. Each gate is represented as a population of bacteria. F1 and F2 are diffusive chemical fields used for communication between bacterial populations. Ara and aTc act as inputs to the circuit. (c) Circuit simulation results for all possible inputs. Each plot relates to a particular population from 1–4 (top–bottom) with colors related to the circuit diagram.
(TIF)

Figure S6 **Results** **for the NAND simulations.** (a) NAND truth table. (b) Circuit used to implement NAND in BSim. Each gate is represented as a population of bacteria. F2 is a diffusive chemical field used for communication between bacterial populations. Ara and aTc act as inputs to the circuit. (c) Circuit simulation results for all possible inputs. Each plot relates to a particular population from 1–3 (top–bottom) with colors related to the circuit diagram.
(TIF)

Figure S7 **Results** **for the AND simulations.** (a) AND truth table. (b) Circuit used to implement AND in BSim. Each gate is represented as a population of bacteria. F2 is a diffusive chemical field used for communication between bacterial populations. Ara and aTc act as inputs to the circuit. (c) Circuit simulation results for all possible inputs. Each plot relates to a particular population from 1–3 (top–bottom) with colors related to the circuit diagram.
(TIF)

Table S1 **Main classes and interfaces of BSim.** These have been split into the packages in which they are found and includes a brief description of their purpose.
(PDF)

Table S2 **Parameter values for the** ***lac*** **operon simulations.**
(PDF)

Video S1 **Multicellular computing with chemical wires.** Simulation of four populations of bacteria (20000 bacteria per population) communicating via chemical signals to calculate the EQUAL logic function. Inputs take the form of chemicals within the medium that remain constant throughout the simulation, specifically, arabinose (Ara) and anhydrotetracycline (aTc). Communication between populations is via two diffusive chemical fields (3OC12-HSL in red and C4-HSL in blue), and activation of individual bacteria is shown by their color with grey being inactive. For this simulation Ara and aTc are not present with the final resting state of population 4 being active, shown by the green color.
(MOV)

Video S2 **Synchronized genetic oscillators.** Simulation of 200 bacteria swimming randomly in a 100×100×100 micron volume with wrapping boundary conditions. Each bacterium uses a system of ODEs to model the essential dynamics of the repressilator gene regulatory network. All bacteria are initialized with random initial conditions, but quickly become synchronized due to AHL communication. The color of the bacteria corresponds to their internal level of *lacI* mRNA, with yellow representing a low and red a high level. AHL concentration within the environment is shown in blue.
(MOV)

Video S3 **Modeling complex spatial environments.** Examples of how complex meshes can be used in BSim. Part 1 illustrates a complex fibrous-like mesh acting as a hard surface to restrict bacterial movement. Part 2 shows a mesh defining a spatially varying parameter, that could be used to alter the behavior of any bacteria. In this simulation the internal state of the bacteria, governed by being inside or outside the torus, is represented by the green and blue colors respectively.
(MOV)

Software S1 **Snapshot of the BSim software from 18th July 2012.** For the latest version see: http://bsim-bccs.sf.net. The BSim software requires Java version 1.6 or higher.
(ZIP)
