# Supplementary material for: BSim: An Agent-Based Tool for Modeling Bacterial Populations in Systems and Synthetic Biology
Source: PLoS One. 2012 Aug 24;7(8):e42790. doi: 10.1371/journal.pone.0042790 (PMC3427305; doi:10.1371/journal.pone.0042790)
Supplement: Text S1 — Further information about the development of the BSim software and models. (PDF) [file pone.0042790.s001.pdf]

BSim: An Agent-Based Tool for Modeling Bacterial Populations in Systems and Synthetic Biology

Thomas E. Gorochowski, Antoni Matyjaszkiewicz, Thomas Todd, Neeraj Oak, Kira Kowalska, Stephen Reid, Krasimira T. Tsaneva-Atanasova, Nigel J. Savery, Claire S. Grierson, Mario di Bernardo

Supporting Text

A Brief History of BSim Page 2
Design and Implementation Page 3
Multicellular computing model details Page 5
Synchronized genetic oscillators model details Page 7
Modeling complex environments Page 9
Population dynamics of the *lac* operon model details Page 10

A Brief History of BSim

BSim began life as part of the BCCS-Bristol team entry into the 2008 International Genetically Engineering Machines (iGEM) competition, see `http://www.igem.org` for further information. Initially, the focus was on developing a computational environment in which to study bacterial chemotaxis, chemical signaling and physical interactions between micro-scale objects, with the aim of understanding how bacterial movement might be used to carry out micro-scale construction (`http://2008.igem.org/Team:BCCS-Bristol`). Since then, in 2009 BSim was re-architected to improve its modular design, provide a three-dimensional environment and use ordinary differential equations to describe bacterial behaviors. These enhancements allowed for the analysis of vesicle production as a method for inter-cellular communication (`http://2009.igem.org/Team:BCCS-Bristol`). Finally, in 2010 enhancements were made to allow for objects with arbitrary geometry enabling the study of how complex environments affect the movement of bacteria, specifically the pore size of a gel bead (`http://2010.igem.org/Team:BCCS-Bristol`).

Design and Implementation

BSim has been developed using standard object-orientated programming techniques and is available for all platforms that support Java version 1.6 or greater, including Windows, Linux and Mac OS X. Source code is hosted on a Google Code repository and latest versions of the software are available from http://bsim-bccs.sf.net. Where possible, reference implementations of standard biological processes have been included, such as chemotaxis, cell division and vesiculation. All reference implementations have been fully tested and to ensure traceability of results developer documentation contains links to original references describing the particular model implemented. A general overview of the main classes and packages is shown in Table S1. For specific details as to defining simulations and some of the validation that has occurred during development please see the user guide and developer documentation included with the software package.

To enable the creation of animated three-dimensional graphics the Processing library released under the GNU Lesser General Public License (LGPL) was used (see http://www.processing.org). This library automatically handles the rendering of graphics in software or hardware, has numerous functions to aid in the creation of graphical objects and provides support for the export of animations to file. In addition, we also made use of the OBJ_IMPORT library written by Fabien Sanglard and released under the GNU LGPL license for loading Wavefront .obj mesh files, and the QuickTimeWriter library written by Werner Randelshofer and released under the Creative Commons Attribution 3.0 license for exporting simulations to video files.

Representing arbitrary chemical fields

Fick's law is used to calculate the diffusion of chemicals within the BSim environment. Due to the possibility of complex user defined structures, this involves solving a differential equation across an arbitrarily shaped three-dimensional space. To do this we use an octree [1] to represent the lattice-like structure over which a finite-difference method is then used to calculate updated chemical concentrations.

An octree is a hierarchical data structure comprising of levels of nested nodes. The root node represents the whole of the initial environment, and links are made to children that subdivide that region into eight equal smaller parts. As you progress down this tree you reach finer and finer resolutions until some predefined cut off. Each node in the tree stores the quantity of chemical present, a diffusivity constant and links to its parent and child nodes. Figure S1a shows a partitioned environment and the corresponding octree representation.

To generate partitions that allow for a good approximation of a user defined structure we

use a three-dimensional version of the method shown in Figure S1b. This recursively partitions regions at the boundary of the object to some required depth. Although this method will not allow for perfect reconstruction of the underlying shape, the adaptive properties allow for a close approximation helping to reduce the computational effort required when numerically solving the diffusion equations.

Performance Characteristics

The performance characteristics of BSim are highly dependant on the particular features of a simulation and the computer hardware on which it is running. Even so, we have carried out analysis for a representative example that includes varying levels of complexity to provide some indication as to running times for different sizes of model. We chose the synchronized genetic oscillators model, described in a later section, because it includes agents with internal ODE dynamics (7 separate equations/states per bacterium) and diffusion via a chemical field. To assess the performance as the number of agents (bacteria) increased we recorded the execution time for 10 minutes of simulation time. We also ran simulations with varying levels of complexity i.e., with and without ODEs and chemical diffusion, to see the effect this had. Simulations were run on a MacBook Pro containing a 2.8GHz Intel Core 2 Duo processor with 8GB DDR3 RAM and using Java 1.6 on Mac OS X 10.7 (Lion). Figure S2 shows the final results which display a linear scaling rate of $\approx$ 0.03 secs/bacterium. It should be noted that simulations were not implemented to make use of parallel computing resources and ran on a single processor core.

Multicellular computing model details

To illustrate the importance of spatial factors when modeling bacterial populations we modeled the results of Tamsir *et al.* in [2]. We considered a scaled down version of the experiment and maintained intracellular activation dynamics to allow for a comparison between results. A BSim environment was created $150 \times 150 \times 12$ microns in size with fixed boundaries (chemical diffusion was contained with no diffusion out of the environment) and 4 locations at which bacterial populations could be placed, see Figure S3. Each population in our simulations consisted of 20000 bacteria which were spatially distributed randomly according to a 2-dimensional Gaussian distribution in the x-y plane with a standard deviation of 3 microns and fixed z-coordinate of 6 microns. This resulted in higher population densities than those observed in experiments [2], but was necessary because the internal agent dynamics activation were based upon realistic absolute concentrations, specifically 30115 molecules/μm^3. Under experimental conditions it is possible to reach these concentrations, even after diffusion, due to the huge numbers of bacteria present in each population. For the scaled down model this concentration could not be met from such a small population and so rather than scale activation constants, we allowed for overcrowding of the populations such that sufficient signalling chemical could be produced.

Input to the system was provided through two chemicals fields, arabinose (Ara) and anhydrotetracycline (aTc), present in the medium and modeled as having fixed concentrations throughout the simulations. Communication between populations used two forms of AHL, specifically, 3OC12-HSL (produced by LasI) and C4-HSL (produced by RhlI). These were modeled as diffusive chemical fields with the same diffusivity and decay rates of 160 μm^2 sec^{-1} and 3.3×10^{-6} molecules sec^{-1} respectively, taken from [2]. Each of these fields was discretised into $150\times150\times2$ regions.

Each virtual bacterium in our model implemented a form of delayed boolean rule, and parameter heterogeneity across each population was captured by variability in the delay values. Specifically, all bacteria start in an inactive state, $a_i(t_0) = 0\ \forall i$, with activation of bacterium i at time t given by,

$$\text{If } a_i(t-dt) = 0 \text{ then, } a_i(t) = \begin{cases} 1, & a_i(t-\tau_i^a) \text{ to } a_i(t) \geq \beta, \\ 0, & \text{otherwise,} \end{cases} \tag{S1}$$

$$\text{else, } a_i(t) = \begin{cases} 0, & a_i(t-\tau_i^d) \text{ to } a_i(t) < \beta, \\ 1, & \text{otherwise,} \end{cases} \tag{S2}$$

where $\tau_i^a \sim \text{Normal}(600, 240)$ and $\tau_i^d \sim \text{Normal}(1200, 480)$ are the activation and inactivation delays in seconds for bacterium i, and $\beta = 30115$ molecules μm^{-3} is the threshold concentration

of the input chemical field for activation (taken from [2] and calculated from their results for the OR gate).

To simplify the creation of different circuits a set of customizable bacterial logic gates were developed, exploiting the modular design of BSim:

1. **BSimLogicBacterium** – Parent bacterium type of all logic gates capturing the essential characteristics of every gate e.g., being able to specify inputs, outputs and a logical function.

2. **BSimNOTBacterium** – NOT gate takes a single chemical field as input and outputs to a reporter.

3. **BSimBufferBacterium** – Buffer that integrates a single chemical field input and outputs to a reporter.

4. **BSimORBacterium** – OR gate that takes two chemical fields as inputs and outputs to a reporter.

5. **BSimNORBacterium** – NOR gate that takes two chemical fields as inputs and outputs to another chemical field or a reporter.

By selecting the appropriate logic gate types for each population and specifying the input and output chemical fields, all of the circuits from [2] could be generated. We replicated the `EQUAL` (Figure S4), `XOR` (Figure S5), `NAND` (Figure S6) and `AND` (Figure S7) circuits. These displayed behaviors that matched experimental results presented in [2].

Synchronized genetic oscillators model details

To analyze the synchronization of genetic oscillators we developed a BSim model to reproduce the results from [3]. We created an environment 100×100×100 microns in size with wrapping boundaries and included 200 randomly moving bacteria. Each of these implemented an ODE model of the GRN presented in [3] to drive behavior. The ODE model consists of 7 ODEs: 3 for mRNA concentrations, 3 for protein concentrations and 1 for the coupling autoinducer. Specifically, mRNA concentration dynamics were given by,

$$\frac{da_i}{dt} = -a_i + \frac{\alpha}{1 + C_i^n}, \tag{S3}$$

$$\frac{db_i}{dt} = -b_i + \frac{\alpha}{1 + A_i^n}, \tag{S4}$$

$$\frac{dc_i}{dt} = -c_i + \frac{\alpha}{1 + B_i^n} + \frac{\kappa S_i}{1 + S_i}, \tag{S5}$$

where a_i, b_i, and c_i refer to the concentration in bacterium i of mRNA transcribed from *tetR*, *cI*, and *lacI*, $\alpha = 216$ is related to the maximal transcription rate, $n = 2$ is a Hill coefficient modeling cooperativity, $\kappa = 20$ is related to the maximal effect of the autoinducer on the transcriptional rate, and S_i is in the internal concentration of the autoinducer. Corresponding proteins are denoted by A_i, B_i, and C_i and their dynamics modeled by,

$$\frac{dX_i}{dt} = \beta \left(x_i - X_i \right), \tag{S6}$$

where the parameter β represents the ratio between the mRNA and protein lifetimes. This was varied across the population being chosen randomly from a Gaussian distribution $\beta \sim \text{Normal}(1, 0.05)$.

To couple individual repressilators together, an autoinducer in the form of AHL was included with internal bacterial dynamics of the form,

$$\frac{dS_i}{dt} = -k_{s0} S_i + k_{s1} A_i - \eta \left(S_i - S_e \right). \tag{S7}$$

Here, η corresponds to the diffusion rate of AHL across the cell membrane and can be calculated from the cell surface area A, cell wall permeability σ, and cell volume V as $\eta = \frac{\sigma A}{V}$, $k_{S0} = 1$ and $k_{S1} = 0.01$ are dimensionless rate parameters, and S_i and S_e are the internal and external cellular concentrations of AHL.

In contrast to the original paper, we used a BSim chemical field rather than a single ODE to

model the spatial characteristics of external AHL. This was governed by the following equation,

$$\frac{\partial \phi(\mathbf{x},t)}{\partial t} = D\nabla^2\phi(\mathbf{x},t) - \delta\phi(\mathbf{x},t) + B(\mathbf{x},t), \tag{S8}$$

where D is the diffusion constant, δ is the decay rate of the chemical, and $B(\mathbf{x},t)$ is creation or extraction of chemical at a particular location due to bacterial agents in the environment. We further allowed for chemicals to diffuse in and out of each bacterial cell. In simulations, AHL chemical field diffusivity D, and decay rate δ were varied, however, in most instances took values of $100\mu\text{m}^2\ \text{sec}^{-1}$ and $0.01\ \text{sec}^{-1}$, respectively. This field was discretised into $25\times25\times25$ regions.

The amount synchronization exhibited by the population was calculated using the order parameter presented in [3]. Specifically, the order parameter $R \in [0,1]$ was given by,

$$R = \frac{\langle M^2\rangle - \langle M\rangle^2}{\overline{\langle b_i^2\rangle - \langle b_i\rangle^2}}, \tag{S9}$$

where $\langle \ldots \rangle$ and $\overline{\ldots}$ denote time and population averages respectively, $b_i(t)$ denotes the local signal for bacterium i, and $M(t)$ is the average population signal calculated using,

$$M(t) = \left(\frac{1}{N}\right)\sum_{i=0}^{N} b_i(t), \tag{S10}$$

where N is the total number of bacteria in the population.

Modeling complex environments

BSim allows for complex spatial features to be represented as meshes that can be used to alter the movement or behavioral characteristics of any agents. To generate the meshes used in this work the freely available application Blender was used, see `http://blender.org` for further information. To allow for these to be compatible with BSim the meshes were saved in the OBJ Wavefront format.

Population dynamics of the *lac* operon model details

To show how macroscopic features can sometimes hide more complex behaviors at the microscopic level, we modeled the well studied *lac* operon system. An environment of 100×100×100 microns was created and we reduced the overall model complexity to focus our efforts on the aspects we were most interested in, choosing to model the dynamics of the *lac* operon at the cellular level using a system of coupled ordinary differential equations obtained from [4]. Specifically, the dynamics of each virtual bacterium was governed by,

$$\frac{d[Y]}{dt} = R_{GEN} - k_2[Y] \tag{S11}$$

$$\frac{d[I]}{dt} = \frac{\alpha[I_{EX}][Y]}{\beta + [I_{EX}]} + \delta([I_{EX}] - [I]) - k_2[I] \tag{S12}$$

$$R_{GEN} = k_1[O_T]\frac{1 + K_1[I]^2}{1 + K_1[I]^2 + K_2[R_T]}, \tag{S13}$$

where $[Y]$, $[I]$ and $[I_{EX}]$ denote the concentration in μM of permease, intracellular inducer and extracellular inducer respectively, and R_{GEN} denotes the rate of permease generation. The extracellular inducer concentration, $[I_{EX}]$, was the main controllable parameter that was varied throughout our simulations; other parameters were selected from [4] and are summarized in Table S2. A chemical field was used to model the diffusion of extracellular inducer with a discretisation into 10×10×10 regions.

Over the time-scales of interest to us, in the region of tens of hours, the simulations would have become computationally intractable if biologically realistic generation and doubling times were used. Therefore, to alleviate this problem we modeled the overall population as having reached an equilibrium density, enabling us to effectively just consider a "slice" of the total population (500 bacteria) and use a probabilistic model for agent generation and removal. At each time step and for each bacterium the generation and removal probabilities are calculated using:

$$\text{Prob(Generation)} = \frac{dt}{t_{Gen}} \times 2^{1-p/l}, \tag{S14}$$

$$\text{Prob(Removal)} = \frac{dt}{t_{Rem}} \times 2^{-1+p/l}, \tag{S15}$$

where dt is the time step size in seconds, $t_{Gen} = 3600$ is the generation time in seconds, $t_{Rem} = 3600$ is the removal time in seconds, p is the current population size, and l is the steady state population size that needs to be maintained.

References

[1] Meagher D (1982) Geometric modeling using octree encoding. Computer Graphics and Image Processing 19: 129 - 147.

[2] Tamsir A, Tabor JJ, Voigt CA (2011) Robust multicellular computing using genetically encoded NOR gates and chemical wires. Nature 469: 212-215.

[3] Garcia-Ojalvo J, Elowitz MB, Strogatz SH (2004) Modeling a synthetic multicellular clock: Repressilators coupled by quorum sensing. Proceedings of the National Academy of Sciences of the United States of America 101: 10955-10960.

[4] Chung J, Stephanopoulos G (1996) On physiological multiplicity and population heterogeneity of biological systems. Chemical engineering science 51: 1509–1521.
