# Supplementary material for: BSim: An Agent-Based Tool for Modeling Bacterial Populations in Systems and Synthetic Biology
Source: PLoS One. 2012 Aug 24;7(8):e42790. doi: 10.1371/journal.pone.0042790 (PMC3427305; doi:10.1371/journal.pone.0042790)
Supplement: Table S1 — Main classes and interfaces of BSim. These have been split into the packages in which they are found and includes a brief description of their purpose. (PDF) [file pone.0042790.s009.pdf]

Type	Name	Description
		`bsim`
Class	`BSim`	Holds all information related to a simulation e.g., boundary, etc.
Class	`BSimTicker`	Controls the main run loop, updating all dynamical processes.
Class	`BSimThreadedTicker`	Threaded version of a normal ticker to exploit parallel processors.
Class	`BSimChemicalField`	Diffusive chemical field that uses uniform discretisation of space.
Class	`BSimOctreeField`	Chemical field that uses a mesh to define non-uniform discretisation.
Class	`BSimUtils`	General functions e.g., gamma distributed random variable, etc.
		`bsim.particle`
Class	`BSimParticle`	Fundamental autonomous entity in a simulation
Class	`BSimBacterium`	Extension of a particle to include bacterial traits.
Class	`BSimVesicle`	Extension of a particle to act as an outer membrane vesicle.
		`bsim.mesh`
Class	`BSimMesh`	Definition of an arbitrary surface in a simulation.
Class	`BSimOBJMesh`	Extension of a mesh to allow loading of OBJ files.
Class	`BSimMeshUtils`	General mesh related functions e.g., nearest intersecting face.
Class	`BSimCollision`	Functions to enable collision detection and repulsion.
		`bsim.ode`
Class	`BSimOdeSolver`	Solvers for objects that implement the ODE system interface.
Interface	`BSimOdeSystem`	Methods necessary to define arbitrary ODE based dynamics.
		`bsim.draw`
Class	`BSimDrawer`	Template of a drawer of the simulation, provides no implementation.
Class	`BSimP3DDrawer`	Extension of a drawer for using the Processing P3D surface.
		`bsim.export`
Class	`BSimExporter`	Template of an exporter, provides no implementation.
Class	`BSimLogger`	Extension of an exporter to output data to text file.
Class	`BSimPngExporter`	Extension of an exporter to simulation to a PNG image file.
Class	`BSimMovExporter`	Extension of an exporter to simulation to a Quicktime movie.
