# Supplementary material for: BSim: An Agent-Based Tool for Modeling Bacterial Populations in Systems and Synthetic Biology
Source: PLoS One. 2012 Aug 24;7(8):e42790. doi: 10.1371/journal.pone.0042790 (PMC3427305; doi:10.1371/journal.pone.0042790)
Supplement: Table S2 — Parameter values for the lac operon simulations. (PDF) [file pone.0042790.s010.pdf]

Parameter	Description	Value
$[O_T]$	Total operator (bound & unbound) concentration	1×10^{-3} μM
$K_2[R_T]$	Total repressor (bound & unbound) concentration $\times$ equilibrium constant	1×10^{5} dimensionless
K_1	Equilibrium constant	0.012 $\mu\mathrm{M}^{-2}$
δ	Facilitated transport coefficient	0.82 min^{-1}
β	Permease saturation constant	500 μM
k_2	Rate of dilution of $[Y]$ and $[I]$	0.0055 min^{-1}
α	Permease turnover number	60000 min^{-1}
k_1	Kinetic rate constant (permease generation)	9 min^{-1}
