# Supplementary material for: BSim: An Agent-Based Tool for Modeling Bacterial Populations in Systems and Synthetic Biology
Source: PLoS One. 2012 Aug 24;7(8):e42790. doi: 10.1371/journal.pone.0042790 (PMC3427305; doi:10.1371/journal.pone.0042790)
Supplement: Software S1 — Snapshot of the BSim software from 18th July 2012. For the latest version see: http://bsim-bccs.sf.net. The BSim software requires Java version 1.6 or higher. (ZIP) [file pone.0042790.s014.zip › BSimSoftware/docs/javadoc/allclasses-frame.html]

All Classes


**All Classes**
  

|  |
| --- |
| AtomDataOutputStream   BSim   BSimBacterium   BSimBacterium.MotionState   BSimChemicalField   BSimCollision   BSimDdeSolver   *BSimDdeSystem*   BSimDrawer   BSimExporter   BSimLogger   BSimMesh   BSimMeshUtils   BSimMovExporter   BSimNotifier   BSimOBJMesh   BSimOctreeField   BSimOdeSolver   *BSimOdeSystem*   BSimP3DDrawer   BSimParticle   BSimPngExporter   BSimSphereMesh   BSimThreadedTicker   BSimThreadedTickerWorker   BSimTicker   BSimTriangle   BSimUtils   BSimVertex   BSimVesicle   FilterImageOutputStream   KdNode   QuickTimeOutputStream   QuickTimeOutputStream.VideoFormat |
