# Supplementary material for: BSim: An Agent-Based Tool for Modeling Bacterial Populations in Systems and Synthetic Biology
Source: PLoS One. 2012 Aug 24;7(8):e42790. doi: 10.1371/journal.pone.0042790 (PMC3427305; doi:10.1371/journal.pone.0042790)
Supplement: Software S1 — Snapshot of the BSim software from 18th July 2012. For the latest version see: http://bsim-bccs.sf.net. The BSim software requires Java version 1.6 or higher. (ZIP) [file pone.0042790.s014.zip › BSimSoftware/docs/javadoc/bsim/BSim.html]

BSim


---


|  |  |  |  |  |  |  |  |  |  |  |
| --- | --- | --- | --- | --- | --- | --- | --- | --- | --- | --- |
| |  |  |  |  |  |  |  |  | | --- | --- | --- | --- | --- | --- | --- | --- | | **Overview** | **Package** | **Class** | **Use** | **Tree** | **Deprecated** | **Index** | **Help** | | |  |
| PREV CLASS   **NEXT CLASS** | **FRAMES**    **NO FRAMES**     **All Classes** |
| SUMMARY: NESTED | FIELD | CONSTR | METHOD | DETAIL: FIELD | CONSTR | METHOD |


---


## bsim Class BSim

```
java.lang.Object
  bsim.BSim
```

---

``` public class BSim extends java.lang.Object ```

Main simulation class.
Holds information related to a simulation including the bounds, types
of boundary, temperature, etc.

---

| **Field Summary** | |
| --- | --- |
| `static double` | `BOLTZMANN`             Boltzmann constant. |


| **Constructor Summary** | |
| --- | --- |
| `BSim()` |


| **Method Summary** | |
| --- | --- |
| `void` | `addExporter(BSimExporter e)`             Add an exporter to be called during simulation. |
| `void` | `export()`             Runs and exports the simulation. |
| `javax.vecmath.Vector3d` | `getBound()`             Return the simulation bounds (microns). |
| `double` | `getDt()`             Return the timestep. |
| `java.lang.String` | `getFormattedTime()`             Return a formatted version of the current time of the simulation. |
| `boolean[]` | `getLeaky()`             Return whether the boundaries are leaky. |
| `double[]` | `getLeakyRate()`             Return the rate that chemicals can escape from the simulation (if the boundary is leaky). |
| `double` | `getSimulationTime()`             Return the length of the simulation. |
| `boolean[]` | `getSolid()`             Return whether the boundaries are solid (reflecting) or wrapping (periiodic). |
| `double` | `getTemperature()`             Return the temperature of the environment. |
| `double` | `getTime()`             Return the current time of the simulation. |
| `double` | `getTimestep()`             Return the current timestep of the simulation. |
| `double` | `getVisc()`             Return the viscosity of the environment. |
| `void` | `preview()`             Runs the simulation in a frame until the frame is closed, ignoring exporters. |
| `void` | `setBound(double x, double y, double z)`             Set the simulation bound (microns). |
| `void` | `setDrawer(BSimDrawer bSimDrawer)`             Set the drawer to be used during simulation. |
| `void` | `setDt(double d)`             Set the timestep (secs). |
| `void` | `setLeaky(boolean xTop, boolean xBottom, boolean yTop, boolean yBottom, boolean zTop, boolean zBottom)`             Set whether the boundaries are leaky. |
| `void` | `setLeakyRate(double xTop, double xBottom, double yTop, double yBottom, double zTop, double zBottom)`             Set the rate that chemicals can escape from the simulation (if the boundary is leaky). |
| `void` | `setSimulationTime(double d)`             Set the length of the simulation (secs). |
| `void` | `setSolid(boolean x, boolean y, boolean z)`             Set whether the boundaries are solid (reflecting) or wrapping (periiodic). |
| `void` | `setTemperature(double t)`             Set the temperature of the environment. |
| `void` | `setTicker(BSimTicker bSimTicker)`             Set the ticker to be used during simulation. |
| `void` | `setTimeFormat(java.lang.String s)`             Set the time format. |
| `void` | `setVisc(double v)`             Set the viscosity of the environment. |
| `int` | `timesteps(double d)`             Returns the number of complete timesteps in the duration d. |

| **Methods inherited from class java.lang.Object** |
| --- |
| `clone, equals, finalize, getClass, hashCode, notify, notifyAll, toString, wait, wait, wait` |

| **Field Detail** |
| --- |

### BOLTZMANN

```
public static double BOLTZMANN
```

:   Boltzmann constant.


| **Constructor Detail** |
| --- |

### BSim

```
public BSim()
```


| **Method Detail** |
| --- |

### setDt

```
public void setDt(double d)
```

:   Set the timestep (secs).

---


### setSimulationTime

```
public void setSimulationTime(double d)
```

:   Set the length of the simulation (secs).

---


### setTimeFormat

```
public void setTimeFormat(java.lang.String s)
```

:   Set the time format. Used to display the time on movies.

---


### setBound

```
public void setBound(double x,
                     double y,
                     double z)
```

:   Set the simulation bound (microns).

---


### setSolid

```
public void setSolid(boolean x,
                     boolean y,
                     boolean z)
```

:   Set whether the boundaries are solid (reflecting) or wrapping (periiodic). Solid = true, relecting = false.

---


### setLeaky

```
public void setLeaky(boolean xTop,
                     boolean xBottom,
                     boolean yTop,
                     boolean yBottom,
                     boolean zTop,
                     boolean zBottom)
```

:   Set whether the boundaries are leaky. A leaky boundary allows for chemicals to escape at some defined rate.

---


### setLeakyRate

```
public void setLeakyRate(double xTop,
                         double xBottom,
                         double yTop,
                         double yBottom,
                         double zTop,
                         double zBottom)
```

:   Set the rate that chemicals can escape from the simulation (if the boundary is leaky).

---


### setVisc

```
public void setVisc(double v)
```

:   Set the viscosity of the environment.

---


### setTemperature

```
public void setTemperature(double t)
```

:   Set the temperature of the environment.

---


### setTicker

```
public void setTicker(BSimTicker bSimTicker)
```

:   Set the ticker to be used during simulation.

---


### setDrawer

```
public void setDrawer(BSimDrawer bSimDrawer)
```

:   Set the drawer to be used during simulation.

---


### addExporter

```
public void addExporter(BSimExporter e)
```

:   Add an exporter to be called during simulation.

---


### getDt

```
public double getDt()
```

:   Return the timestep.

---


### getSimulationTime

```
public double getSimulationTime()
```

:   Return the length of the simulation.

---


### getTimestep

```
public double getTimestep()
```

:   Return the current timestep of the simulation.

---


### getTime

```
public double getTime()
```

:   Return the current time of the simulation.

---


### getFormattedTime

```
public java.lang.String getFormattedTime()
```

:   Return a formatted version of the current time of the simulation.

---


### getBound

```
public javax.vecmath.Vector3d getBound()
```

:   Return the simulation bounds (microns).

---


### getSolid

```
public boolean[] getSolid()
```

:   Return whether the boundaries are solid (reflecting) or wrapping (periiodic). Solid = true, relecting = false.

---


### getLeaky

```
public boolean[] getLeaky()
```

:   Return whether the boundaries are leaky.

---


### getLeakyRate

```
public double[] getLeakyRate()
```

:   Return the rate that chemicals can escape from the simulation (if the boundary is leaky).

---


### getVisc

```
public double getVisc()
```

:   Return the viscosity of the environment.

---


### getTemperature

```
public double getTemperature()
```

:   Return the temperature of the environment.

---


### preview

```
public void preview()
```

:   Runs the simulation in a frame until the frame is closed, ignoring exporters.

---


### export

```
public void export()
```

:   Runs and exports the simulation.

---


### timesteps

```
public int timesteps(double d)
```

:   Returns the number of complete timesteps in the duration d.


---


|  |  |  |  |  |  |  |  |  |  |  |
| --- | --- | --- | --- | --- | --- | --- | --- | --- | --- | --- |
| |  |  |  |  |  |  |  |  | | --- | --- | --- | --- | --- | --- | --- | --- | | **Overview** | **Package** | **Class** | **Use** | **Tree** | **Deprecated** | **Index** | **Help** | | |  |
| PREV CLASS   **NEXT CLASS** | **FRAMES**    **NO FRAMES**     **All Classes** |
| SUMMARY: NESTED | FIELD | CONSTR | METHOD | DETAIL: FIELD | CONSTR | METHOD |


---
