# Supplementary material for: BSim: An Agent-Based Tool for Modeling Bacterial Populations in Systems and Synthetic Biology
Source: PLoS One. 2012 Aug 24;7(8):e42790. doi: 10.1371/journal.pone.0042790 (PMC3427305; doi:10.1371/journal.pone.0042790)
Supplement: Software S1 — Snapshot of the BSim software from 18th July 2012. For the latest version see: http://bsim-bccs.sf.net. The BSim software requires Java version 1.6 or higher. (ZIP) [file pone.0042790.s014.zip › BSimSoftware/docs/javadoc/bsim/BSimChemicalField.html]

BSimChemicalField


---


|  |  |  |  |  |  |  |  |  |  |  |
| --- | --- | --- | --- | --- | --- | --- | --- | --- | --- | --- |
| |  |  |  |  |  |  |  |  | | --- | --- | --- | --- | --- | --- | --- | --- | | **Overview** | **Package** | **Class** | **Use** | **Tree** | **Deprecated** | **Index** | **Help** | | |  |
| **PREV CLASS**   **NEXT CLASS** | **FRAMES**    **NO FRAMES**     **All Classes** |
| SUMMARY: NESTED | FIELD | CONSTR | METHOD | DETAIL: FIELD | CONSTR | METHOD |


---


## bsim Class BSimChemicalField

```
java.lang.Object
  bsim.BSimChemicalField
```

---

``` public class BSimChemicalField extends java.lang.Object ```

Standard chemical field (uniform division of space) .
Uses regular division of the three-dimensional space into boxes that
then allow for diffusion of chemical quantities between the boxes at
some rate (diffusivity).

---

| **Field Summary** | |
| --- | --- |
| `protected  javax.vecmath.Vector3d` | `bound`             sim.getBound(). |
| `protected  double[]` | `box`             Box size. |
| `protected  int[]` | `boxes`             Number of boxes in each dimension. |
| `protected  double` | `boxVolume`             Volume of each box (microns^3). |
| `protected  double` | `decayRate`             Fraction of chemical decaying per second, quantity(t+dt) = quantity(t)\*(1-decayRate\*dt). |
| `protected  double` | `diffusivity`             Diffusivity of the chemical field. |
| `protected  double[][][]` | `quantity`             The quantity of chemical in the box (i,j,k). |
| `protected  BSim` | `sim`             Simulation the chemical field is associated with. |


| **Constructor Summary** | |
| --- | --- |
| `BSimChemicalField(BSim sim, int[] boxes, double diffusivity, double decayRate)`             Constructor that creates a new chemical field with attached to a particular simulation and with a specified number of boxes, chemical diffusivity and decay rate. |


| **Method Summary** | |
| --- | --- |
| `void` | `addQuantity(int x, int y, int z, double q)`             Adds a quantity of chemical to the box (x,y,z). |
| `void` | `addQuantity(javax.vecmath.Vector3d v, double q)`             Adds a quantity of chemical to the box containing position v. |
| `int[]` | `boxCoords(javax.vecmath.Vector3d v)`             Returns the integer coordinates of the box containing the position v. |
| `void` | `decay()`             Decay the chemical present in the field. |
| `void` | `diffuse()`             Diffuse the chemical present in the field. |
| `double[]` | `getBox()`             Return the size of each box (x,y,z) in microns. |
| `int[]` | `getBoxes()`             Return the number of boxes in (x,y,z) directions. |
| `double` | `getConc(int i, int j, int k)`             Gets the concentration of the field in the box (x,y,z) in molecules/(micron)^3. |
| `double` | `getConc(javax.vecmath.Vector3d v)`             Gets the concentration of the field at the position v in molecules/(micron)^3. |
| `void` | `linearGradient(int axis, double startConc, double endConc)`             Creates a linear concentration gradient in the direction specified by 'axis' (x=0, y=1, z=2) |
| `void` | `setConc(double c)`             Sets the concentration of the field |
| `void` | `setConc(int x, int y, int z, double c)`             Sets the concentration of the box (x,y,z). |
| `void` | `setConc(javax.vecmath.Vector3d v, double c)`             Sets the concentration of the box containing position v. |
| `double` | `totalQuantity()`             Returns the total quantity of chemical in the field. |
| `void` | `update()`             Update the chemical field by diffusing and decaying the chemical present. |

| **Methods inherited from class java.lang.Object** |
| --- |
| `clone, equals, finalize, getClass, hashCode, notify, notifyAll, toString, wait, wait, wait` |

| **Field Detail** |
| --- |

### sim

```
protected BSim sim
```

:   Simulation the chemical field is associated with.

---


### diffusivity

```
protected double diffusivity
```

:   Diffusivity of the chemical field.

---


### decayRate

```
protected double decayRate
```

:   Fraction of chemical decaying per second, quantity(t+dt) = quantity(t)\*(1-decayRate\*dt).

---


### quantity

```
protected double[][][] quantity
```

:   The quantity of chemical in the box (i,j,k).

---


### bound

```
protected javax.vecmath.Vector3d bound
```

:   sim.getBound().

---


### boxes

```
protected int[] boxes
```

:   Number of boxes in each dimension.

---


### box

```
protected double[] box
```

:   Box size.

---


### boxVolume

```
protected double boxVolume
```

:   Volume of each box (microns^3).


| **Constructor Detail** |
| --- |

### BSimChemicalField

```
public BSimChemicalField(BSim sim,
                         int[] boxes,
                         double diffusivity,
                         double decayRate)
```

:   Constructor that creates a new chemical field with attached to a particular simulation and
    with a specified number of boxes, chemical diffusivity and decay rate.

    **Parameters:**: `sim` - Associated simulation.: `boxes` - Number of boxes in the (x,y,z) directions.: `diffusivity` - Diffusivity of the chemical (microns)^2/s.: `decayRate` - Decay rate of the chemical (molecules/s).


| **Method Detail** |
| --- |

### getBoxes

```
public int[] getBoxes()
```

:   Return the number of boxes in (x,y,z) directions.

---


### getBox

```
public double[] getBox()
```

:   Return the size of each box (x,y,z) in microns.

---


### linearGradient

```
public void linearGradient(int axis,
                           double startConc,
                           double endConc)
```

:   Creates a linear concentration gradient in the direction specified by 'axis' (x=0, y=1, z=2)

---


### addQuantity

```
public void addQuantity(javax.vecmath.Vector3d v,
                        double q)
```

:   Adds a quantity of chemical to the box containing position v.

---


### addQuantity

```
public void addQuantity(int x,
                        int y,
                        int z,
                        double q)
```

:   Adds a quantity of chemical to the box (x,y,z).

---


### setConc

```
public void setConc(javax.vecmath.Vector3d v,
                    double c)
```

:   Sets the concentration of the box containing position v.

---


### setConc

```
public void setConc(int x,
                    int y,
                    int z,
                    double c)
```

:   Sets the concentration of the box (x,y,z).

---


### setConc

```
public void setConc(double c)
```

:   Sets the concentration of the field

---


### getConc

```
public double getConc(javax.vecmath.Vector3d v)
```

:   Gets the concentration of the field at the position v in molecules/(micron)^3.

---


### getConc

```
public double getConc(int i,
                      int j,
                      int k)
```

:   Gets the concentration of the field in the box (x,y,z) in molecules/(micron)^3.

---


### totalQuantity

```
public double totalQuantity()
```

:   Returns the total quantity of chemical in the field.

---


### boxCoords

```
public int[] boxCoords(javax.vecmath.Vector3d v)
```

:   Returns the integer coordinates of the box containing the position v.

---


### update

```
public void update()
```

:   Update the chemical field by diffusing and decaying the chemical present.

---


### decay

```
public void decay()
```

:   Decay the chemical present in the field.

---


### diffuse

```
public void diffuse()
```

:   Diffuse the chemical present in the field.


---


|  |  |  |  |  |  |  |  |  |  |  |
| --- | --- | --- | --- | --- | --- | --- | --- | --- | --- | --- |
| |  |  |  |  |  |  |  |  | | --- | --- | --- | --- | --- | --- | --- | --- | | **Overview** | **Package** | **Class** | **Use** | **Tree** | **Deprecated** | **Index** | **Help** | | |  |
| **PREV CLASS**   **NEXT CLASS** | **FRAMES**    **NO FRAMES**     **All Classes** |
| SUMMARY: NESTED | FIELD | CONSTR | METHOD | DETAIL: FIELD | CONSTR | METHOD |


---
