# Supplementary material for: BSim: An Agent-Based Tool for Modeling Bacterial Populations in Systems and Synthetic Biology
Source: PLoS One. 2012 Aug 24;7(8):e42790. doi: 10.1371/journal.pone.0042790 (PMC3427305; doi:10.1371/journal.pone.0042790)
Supplement: Software S1 — Snapshot of the BSim software from 18th July 2012. For the latest version see: http://bsim-bccs.sf.net. The BSim software requires Java version 1.6 or higher. (ZIP) [file pone.0042790.s014.zip › BSimSoftware/docs/javadoc/bsim/BSimNotifier.html]

BSimNotifier


---


|  |  |  |  |  |  |  |  |  |  |  |
| --- | --- | --- | --- | --- | --- | --- | --- | --- | --- | --- |
| |  |  |  |  |  |  |  |  | | --- | --- | --- | --- | --- | --- | --- | --- | | **Overview** | **Package** | **Class** | **Use** | **Tree** | **Deprecated** | **Index** | **Help** | | |  |
| **PREV CLASS**   **NEXT CLASS** | **FRAMES**    **NO FRAMES**     **All Classes** |
| SUMMARY: NESTED | FIELD | CONSTR | METHOD | DETAIL: FIELD | CONSTR | METHOD |


---


## bsim Class BSimNotifier

```
java.lang.Object
  bsim.BSimNotifier
```

---

``` public class BSimNotifier extends java.lang.Object ```

Notifier used for multi-threaded tickers.
Threads wait on this notifier to ensure that they are all synchronised
where necessary.

---

| **Constructor Summary** | |
| --- | --- |
| `BSimNotifier()`             Constructor of a notifier (no options available). |


| **Method Summary** | |
| --- | --- |
| `void` | `notifyAllWaiters()`             Notifies all objects waiting on this notifier. |
| `void` | `notifyWaiter()`             Notifies the a single object waiting on this notifier. |
| `void` | `waitForNotify()`             Places object that calls this method in wait cycle. |

| **Methods inherited from class java.lang.Object** |
| --- |
| `clone, equals, finalize, getClass, hashCode, notify, notifyAll, toString, wait, wait, wait` |

| **Constructor Detail** |
| --- |

### BSimNotifier

```
public BSimNotifier()
```

:   Constructor of a notifier (no options available).


| **Method Detail** |
| --- |

### waitForNotify

```
public void waitForNotify()
                   throws java.lang.InterruptedException
```

:   Places object that calls this method in wait cycle.

    :   **Throws:**: `java.lang.InterruptedException` - Thrown when object has been notified.

---


### notifyWaiter

```
public void notifyWaiter()
```

:   Notifies the a single object waiting on this notifier.

---


### notifyAllWaiters

```
public void notifyAllWaiters()
```

:   Notifies all objects waiting on this notifier.


---


|  |  |  |  |  |  |  |  |  |  |  |
| --- | --- | --- | --- | --- | --- | --- | --- | --- | --- | --- |
| |  |  |  |  |  |  |  |  | | --- | --- | --- | --- | --- | --- | --- | --- | | **Overview** | **Package** | **Class** | **Use** | **Tree** | **Deprecated** | **Index** | **Help** | | |  |
| **PREV CLASS**   **NEXT CLASS** | **FRAMES**    **NO FRAMES**     **All Classes** |
| SUMMARY: NESTED | FIELD | CONSTR | METHOD | DETAIL: FIELD | CONSTR | METHOD |


---
