# Supplementary material for: BSim: An Agent-Based Tool for Modeling Bacterial Populations in Systems and Synthetic Biology
Source: PLoS One. 2012 Aug 24;7(8):e42790. doi: 10.1371/journal.pone.0042790 (PMC3427305; doi:10.1371/journal.pone.0042790)
Supplement: Software S1 — Snapshot of the BSim software from 18th July 2012. For the latest version see: http://bsim-bccs.sf.net. The BSim software requires Java version 1.6 or higher. (ZIP) [file pone.0042790.s014.zip › BSimSoftware/docs/javadoc/bsim/BSimOctreeField.html]

BSimOctreeField


---


|  |  |  |  |  |  |  |  |  |  |  |
| --- | --- | --- | --- | --- | --- | --- | --- | --- | --- | --- |
| |  |  |  |  |  |  |  |  | | --- | --- | --- | --- | --- | --- | --- | --- | | **Overview** | **Package** | **Class** | **Use** | **Tree** | **Deprecated** | **Index** | **Help** | | |  |
| **PREV CLASS**   **NEXT CLASS** | **FRAMES**    **NO FRAMES**     **All Classes** |
| SUMMARY: NESTED | FIELD | CONSTR | METHOD | DETAIL: FIELD | CONSTR | METHOD |


---


## bsim Class BSimOctreeField

```
java.lang.Object
  bsim.BSimOctreeField
```

---

``` public class BSimOctreeField extends java.lang.Object ```

Octree chemical field (non-uniform division of space).
Uses an octree datatype to compute a chemical field of an arbitrary shape. Only one
BSimOctreeField object needs to be created by the user, it subdivides into an appropriate
shape with the fitFieldtoMesh() method. Decay and diffusion are performed through post
order traversals.

---

| **Field Summary** | |
| --- | --- |
| `protected  javax.vecmath.Vector3d` | `centre`             Location of centre of node in space. |
| `protected  int` | `depth`             Depth in octree structure, root has depth 0. |
| `protected  double` | `diffusivity`             Diffusivity of node. |
| `protected  double` | `length`             Dimension of the node. |
| `protected  java.awt.Color` | `nodeColor`             Color of the node, used when drawing. |
| `protected  BSimOctreeField` | `parent`             Parent of node, for root this is null. |
| `boolean` | `processed`             For checking in division algorithm. |
| `protected  double` | `quantity`             number of molecules in the chemical field box |
| `protected  BSimOctreeField[]` | `subNodes`             subNodes of octree, these can have subnodes of their own. |
| `protected  double` | `volume`             Volume of node (simply length^3). |


| **Constructor Summary** | |
| --- | --- |
| `BSimOctreeField()`             Constructor for a basic BSimOctreeField (default settings). |
| `BSimOctreeField(javax.vecmath.Vector3d Centre, double Length)`             Root Constructor - only used to make first root Octree Node |


| **Method Summary** | |
| --- | --- |
| `void` | `colorFromCentre(BSimOctreeField t)`             Sets the nodeColor value as a function of the position of octree, useful for troubleshooting. |
| `void` | `colorFromConc()`             Sets nodeCololr value as a function of amount of chemical in box. |
| `void` | `decay(BSimOctreeField t, double decayRate, double Dt)`             Decays the chemical field in an octreeField Node,visits each node in the tree structure using a post-order traverse. |
| `void` | `diffuse(BSimOctreeField t, double diffusivity, double Dt, int depth)`             Diffuses chemicals through whole the octreeField structure, using Fick's law to determine how much of the chemical gets pushed into neighboring nodes over each time iteration |
| `javax.vecmath.Vector3d` | `getCentre()`             Return the centre. |
| `int` | `getDepth()`             Return the depth. |
| `double` | `getDiffusivity()`             Return the diffusivity. |
| `double` | `getLength()`             Return the length. |
| `java.awt.Color` | `getnodeColor()`             Return the node colour. |
| `double` | `getQuantity()`             Return the chemical quantity. |
| `BSimOctreeField` | `getsubNode(int i)`             Return the subNode (i is index of subnode). |
| `static void` | `inOrderfull(BSimOctreeField t)`             In-Order traverse, traverses from the deepest subnode, to the root and then back down to other deep nodes. |
| `static boolean` | `intersectVectorTriangle(javax.vecmath.Vector3d startPos, javax.vecmath.Vector3d endPos, BSimTriangle tri)` |
| `BSimOctreeField` | `nodeFinder(BSimOctreeField t, javax.vecmath.Vector3d location)`             Gets a subnode of given index from lowest depth. |
| `static void` | `postOrderfull(BSimOctreeField t)`             Post-Order traverse with visit function. |
| `static void` | `preOrderfull(BSimOctreeField t)`             Pre-Order full traverse - traverses from the root, a direction to the deepest subnode, back to the node, and then down into other roots. |
| `void` | `setColor(java.awt.Color c)`             Set the colour. |
| `void` | `setKids()`             Initializes all the subNodes with appropriate neighbors/centers and lengths. |
| `void` | `setNodestoMesh(BSimMesh theMesh, BSimOctreeField t)`             Fits octreeField against a mesh and splits into subNodes when there is a collision with the mesh boundary Creates a finer octree structure each time this function is called. |
| `void` | `visit(BSimOctreeField t)`             The visit method simply prints the location and depth of a node, useful for troubleshooting. |

| **Methods inherited from class java.lang.Object** |
| --- |
| `clone, equals, finalize, getClass, hashCode, notify, notifyAll, toString, wait, wait, wait` |

| **Field Detail** |
| --- |

### parent

```
protected BSimOctreeField parent
```

:   Parent of node, for root this is null.

---


### nodeColor

```
protected java.awt.Color nodeColor
```

:   Color of the node, used when drawing.

---


### centre

```
protected javax.vecmath.Vector3d centre
```

:   Location of centre of node in space.

---


### length

```
protected double length
```

:   Dimension of the node.

---


### diffusivity

```
protected double diffusivity
```

:   Diffusivity of node. Unless otherwise specified this is inherited from parent.

---


### volume

```
protected double volume
```

:   Volume of node (simply length^3).

---


### depth

```
protected int depth
```

:   Depth in octree structure, root has depth 0.

---


### subNodes

```
protected BSimOctreeField[] subNodes
```

:   subNodes of octree, these can have subnodes of their own.

---


### quantity

```
protected double quantity
```

:   number of molecules in the chemical field box

---


### processed

```
public boolean processed
```

:   For checking in division algorithm.


| **Constructor Detail** |
| --- |

### BSimOctreeField

```
public BSimOctreeField()
```

:   Constructor for a basic BSimOctreeField (default settings).

---


### BSimOctreeField

```
public BSimOctreeField(javax.vecmath.Vector3d Centre,
                       double Length)
```

:   Root Constructor - only used to make first root Octree Node


| **Method Detail** |
| --- |

### getDepth

```
public int getDepth()
```

:   Return the depth.

---


### getCentre

```
public javax.vecmath.Vector3d getCentre()
```

:   Return the centre.

---


### getLength

```
public double getLength()
```

:   Return the length.

---


### getsubNode

```
public BSimOctreeField getsubNode(int i)
```

:   Return the subNode (i is index of subnode).

---


### getnodeColor

```
public java.awt.Color getnodeColor()
```

:   Return the node colour.

---


### getDiffusivity

```
public double getDiffusivity()
```

:   Return the diffusivity.

---


### getQuantity

```
public double getQuantity()
```

:   Return the chemical quantity.

---


### setColor

```
public void setColor(java.awt.Color c)
```

:   Set the colour.

---


### setKids

```
public void setKids()
```

:   Initializes all the subNodes with appropriate neighbors/centers and lengths.
    All other properties are inherited from the parent node.

---


### setNodestoMesh

```
public void setNodestoMesh(BSimMesh theMesh,
                           BSimOctreeField t)
```

:   Fits octreeField against a mesh and splits into subNodes when there is a collision with the mesh boundary
    Creates a finer octree structure each time this function is called. Should be called
    in a loop,with the number of repetitions being the maximum depth of the octree structure

    :   **Parameters:**: `theMesh` - - BSimMesh object that the OctreeField is going to be fit to: `t` - - the OctreeField that will be fit to the mesh

---


### preOrderfull

```
public static void preOrderfull(BSimOctreeField t)
```

:   Pre-Order full traverse - traverses from the root, a direction to the
    deepest subnode, back to the node, and then down into other roots.
    Not recommended, instead see postOrderfull.

---


### inOrderfull

```
public static void inOrderfull(BSimOctreeField t)
```

:   In-Order traverse, traverses from the deepest subnode, to the root
    and then back down to other deep nodes.
    Not recommended, instead see postOrderfull().

---


### postOrderfull

```
public static void postOrderfull(BSimOctreeField t)
```

:   Post-Order traverse with visit function. This is the most logical
    traverse and visits octrees in 'left to right, bottom to top' sense
    this is used to traversing octree structures.

---


### nodeFinder

```
public BSimOctreeField nodeFinder(BSimOctreeField t,
                                  javax.vecmath.Vector3d location)
```

:   Gets a subnode of given index from lowest depth.

---


### visit

```
public void visit(BSimOctreeField t)
```

:   The visit method simply prints the location and depth of
    a node, useful for troubleshooting.

---


### colorFromCentre

```
public void colorFromCentre(BSimOctreeField t)
```

:   Sets the nodeColor value as a function of the position of octree,
    useful for troubleshooting.

---


### colorFromConc

```
public void colorFromConc()
```

:   Sets nodeCololr value as a function of amount of chemical in box.

---


### diffuse

```
public void diffuse(BSimOctreeField t,
                    double diffusivity,
                    double Dt,
                    int depth)
```

:   Diffuses chemicals through whole the octreeField structure, using Fick's law to determine
    how much of the chemical gets pushed into neighboring nodes over each time iteration

    :   **Parameters:**: `diffusivity` - The diffusivity in (microns)^2/s.: `Dt` - Time step in seconds.: `depth` - Maximum depth to go to in diffusion.

---


### decay

```
public void decay(BSimOctreeField t,
                  double decayRate,
                  double Dt)
```

:   Decays the chemical field in an octreeField Node,visits each node in the tree structure
    using a post-order traverse.

    :   **Parameters:**: `t` - The octree node.: `decayRate` - Decay rate of the chemical (seconds^-1).: `Dt` - Timestep to use (seconds).

---


### intersectVectorTriangle

```
public static boolean intersectVectorTriangle(javax.vecmath.Vector3d startPos,
                                              javax.vecmath.Vector3d endPos,
                                              BSimTriangle tri)
```


---


|  |  |  |  |  |  |  |  |  |  |  |
| --- | --- | --- | --- | --- | --- | --- | --- | --- | --- | --- |
| |  |  |  |  |  |  |  |  | | --- | --- | --- | --- | --- | --- | --- | --- | | **Overview** | **Package** | **Class** | **Use** | **Tree** | **Deprecated** | **Index** | **Help** | | |  |
| **PREV CLASS**   **NEXT CLASS** | **FRAMES**    **NO FRAMES**     **All Classes** |
| SUMMARY: NESTED | FIELD | CONSTR | METHOD | DETAIL: FIELD | CONSTR | METHOD |


---
