# Supplementary material for: BSim: An Agent-Based Tool for Modeling Bacterial Populations in Systems and Synthetic Biology
Source: PLoS One. 2012 Aug 24;7(8):e42790. doi: 10.1371/journal.pone.0042790 (PMC3427305; doi:10.1371/journal.pone.0042790)
Supplement: Software S1 — Snapshot of the BSim software from 18th July 2012. For the latest version see: http://bsim-bccs.sf.net. The BSim software requires Java version 1.6 or higher. (ZIP) [file pone.0042790.s014.zip › BSimSoftware/docs/javadoc/bsim/BSimThreadedTicker.html]

BSimThreadedTicker


---


|  |  |  |  |  |  |  |  |  |  |  |
| --- | --- | --- | --- | --- | --- | --- | --- | --- | --- | --- |
| |  |  |  |  |  |  |  |  | | --- | --- | --- | --- | --- | --- | --- | --- | | **Overview** | **Package** | **Class** | **Use** | **Tree** | **Deprecated** | **Index** | **Help** | | |  |
| **PREV CLASS**   **NEXT CLASS** | **FRAMES**    **NO FRAMES**     **All Classes** |
| SUMMARY: NESTED | FIELD | CONSTR | METHOD | DETAIL: FIELD | CONSTR | METHOD |


---


## bsim Class BSimThreadedTicker

```
java.lang.Object
  bsim.BSimTicker
      bsim.BSimThreadedTicker
```

---

``` public abstract class BSimThreadedTicker extends BSimTicker ```

Multi-threaded ticker.
This can be used instead of a standard ticker and will allow for concurrent
updating of appropriate parts of a simulation. Care must be taken to ensure
that data structures are not updated and read by two different threads. A
user must define a BSimThreadedTickerWorker object that captures how the
ticker should split up the update task.

---

| **Field Summary** | |
| --- | --- |
| `protected  BSimThreadedTickerWorker` | `myWorker`             A local working for the main thread to call directly. |
| `protected  int` | `threads`             Total number of threads (including main one). |
| `protected  java.util.Vector<BSimThreadedTickerWorker>` | `workers`             List of workers to call upon. |


| **Constructor Summary** | |
| --- | --- |
| `BSimThreadedTicker(int threads)`             Constructor that creates a threaded ticker that uses a fixed size pool of threads. |


| **Method Summary** | |
| --- | --- |
| `abstract  BSimThreadedTickerWorker` | `createWorker(int threadID, int threads)`             For the user to overwrite to create suitable workers for this ticker. |
| `abstract  void` | `sequentialAfter()` |
| `abstract  void` | `sequentialBefore()`             Overwrite these with sequential operations to run before and after the parallel block. |
| `void` | `tick()`             Called at each time step by the BSim simulation object. |

| **Methods inherited from class java.lang.Object** |
| --- |
| `clone, equals, finalize, getClass, hashCode, notify, notifyAll, toString, wait, wait, wait` |

| **Field Detail** |
| --- |

### threads

```
protected int threads
```

:   Total number of threads (including main one).

---


### workers

```
protected java.util.Vector<BSimThreadedTickerWorker> workers
```

:   List of workers to call upon.

---


### myWorker

```
protected BSimThreadedTickerWorker myWorker
```

:   A local working for the main thread to call directly.


| **Constructor Detail** |
| --- |

### BSimThreadedTicker

```
public BSimThreadedTicker(int threads)
```

:   Constructor that creates a threaded ticker that uses a fixed size pool of threads. These are
    not recreated each call due to the sizable overhead in Java, but instead
    a pool of worker threads is blocked and notified to carry out work each
    time the ticker is called.

    **Parameters:**: `threads` - Total number of threads to use.


| **Method Detail** |
| --- |

### tick

```
public final void tick()
```

:   Called at each time step by the BSim simulation object. Runs the sequential before, the
    workers code, and then the sequential after.

    :   **Specified by:**: `tick` in class `BSimTicker`

---


### sequentialBefore

```
public abstract void sequentialBefore()
```

:   Overwrite these with sequential operations to run before and after
    the parallel block.

---


### sequentialAfter

```
public abstract void sequentialAfter()
```

---


### createWorker

```
public abstract BSimThreadedTickerWorker createWorker(int threadID,
                                                      int threads)
```

:   For the user to overwrite to create suitable workers for this ticker.

    :   **Parameters:**: `threadID` - Unique thread ID: `threads` - Total number of threads **Returns:**: New worker object that will be called in parallel


---


|  |  |  |  |  |  |  |  |  |  |  |
| --- | --- | --- | --- | --- | --- | --- | --- | --- | --- | --- |
| |  |  |  |  |  |  |  |  | | --- | --- | --- | --- | --- | --- | --- | --- | | **Overview** | **Package** | **Class** | **Use** | **Tree** | **Deprecated** | **Index** | **Help** | | |  |
| **PREV CLASS**   **NEXT CLASS** | **FRAMES**    **NO FRAMES**     **All Classes** |
| SUMMARY: NESTED | FIELD | CONSTR | METHOD | DETAIL: FIELD | CONSTR | METHOD |


---
