# Supplementary material for: BSim: An Agent-Based Tool for Modeling Bacterial Populations in Systems and Synthetic Biology
Source: PLoS One. 2012 Aug 24;7(8):e42790. doi: 10.1371/journal.pone.0042790 (PMC3427305; doi:10.1371/journal.pone.0042790)
Supplement: Software S1 — Snapshot of the BSim software from 18th July 2012. For the latest version see: http://bsim-bccs.sf.net. The BSim software requires Java version 1.6 or higher. (ZIP) [file pone.0042790.s014.zip › BSimSoftware/docs/javadoc/bsim/BSimThreadedTickerWorker.html]

BSimThreadedTickerWorker


---


|  |  |  |  |  |  |  |  |  |  |  |
| --- | --- | --- | --- | --- | --- | --- | --- | --- | --- | --- |
| |  |  |  |  |  |  |  |  | | --- | --- | --- | --- | --- | --- | --- | --- | | **Overview** | **Package** | **Class** | **Use** | **Tree** | **Deprecated** | **Index** | **Help** | | |  |
| **PREV CLASS**   **NEXT CLASS** | **FRAMES**    **NO FRAMES**     **All Classes** |
| SUMMARY: NESTED | FIELD | CONSTR | METHOD | DETAIL: FIELD | CONSTR | METHOD |


---


## bsim Class BSimThreadedTickerWorker

```
java.lang.Object
  bsim.BSimThreadedTickerWorker
```

**All Implemented Interfaces:**: java.lang.Runnable

---

``` public abstract class BSimThreadedTickerWorker extends java.lang.Object implements java.lang.Runnable ```

Multi-threaded ticker worker.
This carries out the actual update task and captures how individual threads
should work on separate parts of the simulation.

---

| **Field Summary** | |
| --- | --- |
| `protected static java.util.concurrent.CyclicBarrier` | `barrier1`             Shared barrier to enable synchronisation of all threads at start of update. |
| `protected static java.util.concurrent.CyclicBarrier` | `barrier2`             Shared barrier to enable synchronisation of all threads at end of update. |
| `protected  int` | `threadID`             The ID of this worker. |
| `protected  int` | `threads`             Total number of threads in the pool. |


| **Constructor Summary** | |
| --- | --- |
| `BSimThreadedTickerWorker(int threadID, int threads)`             Constructor to create a new worker for the BSimThreadedTicker. |


| **Method Summary** | |
| --- | --- |
| `void` | `run()`             Threaded function. |
| `abstract  void` | `threadedTick(int threadID, int threads)`             Run each timestep in parallel, use the threadID to figure out which part of the problem to work on. |

| **Methods inherited from class java.lang.Object** |
| --- |
| `clone, equals, finalize, getClass, hashCode, notify, notifyAll, toString, wait, wait, wait` |

| **Field Detail** |
| --- |

### threadID

```
protected int threadID
```

:   The ID of this worker.

---


### threads

```
protected int threads
```

:   Total number of threads in the pool.

---


### barrier1

```
protected static java.util.concurrent.CyclicBarrier barrier1
```

:   Shared barrier to enable synchronisation of all threads at start of update.

---


### barrier2

```
protected static java.util.concurrent.CyclicBarrier barrier2
```

:   Shared barrier to enable synchronisation of all threads at end of update.


| **Constructor Detail** |
| --- |

### BSimThreadedTickerWorker

```
public BSimThreadedTickerWorker(int threadID,
                                int threads)
```

:   Constructor to create a new worker for the BSimThreadedTicker.

    **Parameters:**: `threadID` - Unique thread ID for the worker.: `threads` - Total number of threads.


| **Method Detail** |
| --- |

### run

```
public final void run()
```

:   Threaded function. The first (threadID = 0) is treated specially as this is
    the main application thread and therefore should not enter a waiting state.

    :   **Specified by:**: `run` in interface `java.lang.Runnable`

---


### threadedTick

```
public abstract void threadedTick(int threadID,
                                  int threads)
```

:   Run each timestep in parallel, use the threadID to figure
    out which part of the problem to work on. threads is the
    total number of threads created. To be overwritten by user.


---


|  |  |  |  |  |  |  |  |  |  |  |
| --- | --- | --- | --- | --- | --- | --- | --- | --- | --- | --- |
| |  |  |  |  |  |  |  |  | | --- | --- | --- | --- | --- | --- | --- | --- | | **Overview** | **Package** | **Class** | **Use** | **Tree** | **Deprecated** | **Index** | **Help** | | |  |
| **PREV CLASS**   **NEXT CLASS** | **FRAMES**    **NO FRAMES**     **All Classes** |
| SUMMARY: NESTED | FIELD | CONSTR | METHOD | DETAIL: FIELD | CONSTR | METHOD |


---
