# Supplementary material for: BSim: An Agent-Based Tool for Modeling Bacterial Populations in Systems and Synthetic Biology
Source: PLoS One. 2012 Aug 24;7(8):e42790. doi: 10.1371/journal.pone.0042790 (PMC3427305; doi:10.1371/journal.pone.0042790)
Supplement: Software S1 — Snapshot of the BSim software from 18th July 2012. For the latest version see: http://bsim-bccs.sf.net. The BSim software requires Java version 1.6 or higher. (ZIP) [file pone.0042790.s014.zip › BSimSoftware/docs/javadoc/bsim/BSimUtils.html]

BSimUtils


---


|  |  |  |  |  |  |  |  |  |  |  |
| --- | --- | --- | --- | --- | --- | --- | --- | --- | --- | --- |
| |  |  |  |  |  |  |  |  | | --- | --- | --- | --- | --- | --- | --- | --- | | **Overview** | **Package** | **Class** | **Use** | **Tree** | **Deprecated** | **Index** | **Help** | | |  |
| **PREV CLASS**   NEXT CLASS | **FRAMES**    **NO FRAMES**     **All Classes** |
| SUMMARY: NESTED | FIELD | CONSTR | METHOD | DETAIL: FIELD | CONSTR | METHOD |


---


## bsim Class BSimUtils

```
java.lang.Object
  bsim.BSimUtils
```

---

``` public class BSimUtils extends java.lang.Object ```

Utility functions.
Defines a wide array of utility functions used throughout BSim.

---

| **Constructor Summary** | |
| --- | --- |
| `BSimUtils()` |


| **Method Summary** | |
| --- | --- |
| `static java.lang.String` | `generateDirectoryPath(java.lang.String dirPath)`             Checks the existence of a desired path. |
| `static java.lang.String` | `padInt2(int val)`             Returns a padded version of the number to a size of two. |
| `static void` | `rotate(javax.vecmath.Vector3d v, javax.vecmath.Vector3d axis, double theta)`             Rotates the vector v towards the specified axis by an angle theta. |
| `static void` | `rotatePerp(javax.vecmath.Vector3d v, double theta)`             Rotates the vector v by an angle theta in a random direction perpendicular to v. |
| `static double` | `sampleGamma(double k, double theta)`             Sample from a gamma distribution. |
| `static java.lang.String` | `timeStamp()`             Returns a string representation of the current date and time. |

| **Methods inherited from class java.lang.Object** |
| --- |
| `clone, equals, finalize, getClass, hashCode, notify, notifyAll, toString, wait, wait, wait` |

| **Constructor Detail** |
| --- |

### BSimUtils

```
public BSimUtils()
```


| **Method Detail** |
| --- |

### sampleGamma

```
public static double sampleGamma(double k,
                                 double theta)
```

:   Sample from a gamma distribution. See:
    http://vyshemirsky.blogspot.com/2007/11/sample-from-gamma-distribution-in-java.html

---


### rotatePerp

```
public static void rotatePerp(javax.vecmath.Vector3d v,
                              double theta)
```

:   Rotates the vector v by an angle theta in a random direction perpendicular to v.

---


### rotate

```
public static void rotate(javax.vecmath.Vector3d v,
                          javax.vecmath.Vector3d axis,
                          double theta)
```

:   Rotates the vector v towards the specified axis by an angle theta.

---


### timeStamp

```
public static java.lang.String timeStamp()
```

:   Returns a string representation of the current date and time.

    :   **Returns:**: String representation of the current date and time.

---


### padInt2

```
public static java.lang.String padInt2(int val)
```

:   Returns a padded version of the number to a size of two.

---


### generateDirectoryPath

```
public static java.lang.String generateDirectoryPath(java.lang.String dirPath)
```

:   Checks the existence of a desired path. If the path does not exist,
    then the method will generate the necessary directories.

    :   **Parameters:**: `dirPath` - The desired directory path to check and generate. **Returns:**: String representation of the desired directory path.


---


|  |  |  |  |  |  |  |  |  |  |  |
| --- | --- | --- | --- | --- | --- | --- | --- | --- | --- | --- |
| |  |  |  |  |  |  |  |  | | --- | --- | --- | --- | --- | --- | --- | --- | | **Overview** | **Package** | **Class** | **Use** | **Tree** | **Deprecated** | **Index** | **Help** | | |  |
| **PREV CLASS**   NEXT CLASS | **FRAMES**    **NO FRAMES**     **All Classes** |
| SUMMARY: NESTED | FIELD | CONSTR | METHOD | DETAIL: FIELD | CONSTR | METHOD |


---
