# Supplementary material for: BSim: An Agent-Based Tool for Modeling Bacterial Populations in Systems and Synthetic Biology
Source: PLoS One. 2012 Aug 24;7(8):e42790. doi: 10.1371/journal.pone.0042790 (PMC3427305; doi:10.1371/journal.pone.0042790)
Supplement: Software S1 — Snapshot of the BSim software from 18th July 2012. For the latest version see: http://bsim-bccs.sf.net. The BSim software requires Java version 1.6 or higher. (ZIP) [file pone.0042790.s014.zip › BSimSoftware/docs/javadoc/bsim/class-use/BSim.html]

Uses of Class bsim.BSim


---


|  |  |  |  |  |  |  |  |  |  |  |
| --- | --- | --- | --- | --- | --- | --- | --- | --- | --- | --- |
| |  |  |  |  |  |  |  |  | | --- | --- | --- | --- | --- | --- | --- | --- | | **Overview** | **Package** | **Class** | **Use** | **Tree** | **Deprecated** | **Index** | **Help** | | |  |
| PREV   NEXT | **FRAMES**    **NO FRAMES**     **All Classes** |


---


## **Uses of Class bsim.BSim**

| Packages that use BSim | |
| --- | --- |
| **bsim** |  |
| **bsim.draw** |  |
| **bsim.export** |  |
| **bsim.particle** |  |

| Uses of BSim in bsim | |
| --- | --- |

| Fields in bsim declared as BSim | |
| --- | --- |
| `protected  BSim` | `BSimChemicalField.sim`             Simulation the chemical field is associated with. |

| Constructors in bsim with parameters of type BSim | |
| --- | --- |
| `BSimChemicalField(BSim sim, int[] boxes, double diffusivity, double decayRate)`             Constructor that creates a new chemical field with attached to a particular simulation and with a specified number of boxes, chemical diffusivity and decay rate. |

| Uses of BSim in bsim.draw | |
| --- | --- |

| Fields in bsim.draw declared as BSim | |
| --- | --- |
| `protected  BSim` | `BSimDrawer.sim`             The simulation. |

| Constructors in bsim.draw with parameters of type BSim | |
| --- | --- |
| `BSimDrawer(BSim sim, int width, int height)`             Constructor for a drawer. |
| `BSimP3DDrawer(BSim sim, int width, int height)`             Default constructor for initialising a Processing3D rendering context. |

| Uses of BSim in bsim.export | |
| --- | --- |

| Fields in bsim.export declared as BSim | |
| --- | --- |
| `protected  BSim` | `BSimExporter.sim`             Associated simulation. |

| Constructors in bsim.export with parameters of type BSim | |
| --- | --- |
| `BSimExporter(BSim sim)`             Constructor of a basic exporter. |
| `BSimLogger(BSim sim, java.lang.String filename)`             Constructor for a file logger. |
| `BSimMovExporter(BSim sim, BSimDrawer drawer, java.lang.String filename)`             Constructor for the movie exporter. |
| `BSimPngExporter(BSim sim, BSimDrawer drawer, java.lang.String directory)`             Constructor for the image exporter |

| Uses of BSim in bsim.particle | |
| --- | --- |

| Fields in bsim.particle declared as BSim | |
| --- | --- |
| `protected  BSim` | `BSimParticle.sim` |

| Constructors in bsim.particle with parameters of type BSim | |
| --- | --- |
| `BSimBacterium(BSim sim, javax.vecmath.Vector3d position)`             Creates a RUNNING bacterium at the specified position, facing in a random direction |
| `BSimParticle(BSim sim, javax.vecmath.Vector3d position, double radius)` |
| `BSimVesicle(BSim sim, javax.vecmath.Vector3d position, double radius)`             Constructor for a vesicle at a position and of a given size. |

---


|  |  |  |  |  |  |  |  |  |  |  |
| --- | --- | --- | --- | --- | --- | --- | --- | --- | --- | --- |
| |  |  |  |  |  |  |  |  | | --- | --- | --- | --- | --- | --- | --- | --- | | **Overview** | **Package** | **Class** | **Use** | **Tree** | **Deprecated** | **Index** | **Help** | | |  |
| PREV   NEXT | **FRAMES**    **NO FRAMES**     **All Classes** |


---
