# Supplementary material for: BSim: An Agent-Based Tool for Modeling Bacterial Populations in Systems and Synthetic Biology
Source: PLoS One. 2012 Aug 24;7(8):e42790. doi: 10.1371/journal.pone.0042790 (PMC3427305; doi:10.1371/journal.pone.0042790)
Supplement: Software S1 — Snapshot of the BSim software from 18th July 2012. For the latest version see: http://bsim-bccs.sf.net. The BSim software requires Java version 1.6 or higher. (ZIP) [file pone.0042790.s014.zip › BSimSoftware/docs/javadoc/bsim/class-use/BSimChemicalField.html]

Uses of Class bsim.BSimChemicalField


---


|  |  |  |  |  |  |  |  |  |  |  |
| --- | --- | --- | --- | --- | --- | --- | --- | --- | --- | --- |
| |  |  |  |  |  |  |  |  | | --- | --- | --- | --- | --- | --- | --- | --- | | **Overview** | **Package** | **Class** | **Use** | **Tree** | **Deprecated** | **Index** | **Help** | | |  |
| PREV   NEXT | **FRAMES**    **NO FRAMES**     **All Classes** |


---


## **Uses of Class bsim.BSimChemicalField**

| Packages that use BSimChemicalField | |
| --- | --- |
| **bsim.draw** |  |
| **bsim.particle** |  |

| Uses of BSimChemicalField in bsim.draw | |
| --- | --- |

| Methods in bsim.draw with parameters of type BSimChemicalField | |
| --- | --- |
| `void` | `BSimP3DDrawer.draw(BSimChemicalField field, java.awt.Color c, double alphaGrad, double alphaMax)`             Draws a chemical field structure based on its defined parameters, with custom transparency (alpha) parameters. |
| `void` | `BSimP3DDrawer.draw(BSimChemicalField field, java.awt.Color c, float alphaGrad)`             Draw a chemical field structure based on its defined parameters (default alpha). |

| Uses of BSimChemicalField in bsim.particle | |
| --- | --- |

| Fields in bsim.particle declared as BSimChemicalField | |
| --- | --- |
| `protected  BSimChemicalField` | `BSimBacterium.goal`             Bacteria tend to swim towards higher concentrations of this chemical field. |

| Methods in bsim.particle with parameters of type BSimChemicalField | |
| --- | --- |
| `void` | `BSimBacterium.setGoal(BSimChemicalField goal)`             Set this chemical field as the goal field. |

---


|  |  |  |  |  |  |  |  |  |  |  |
| --- | --- | --- | --- | --- | --- | --- | --- | --- | --- | --- |
| |  |  |  |  |  |  |  |  | | --- | --- | --- | --- | --- | --- | --- | --- | | **Overview** | **Package** | **Class** | **Use** | **Tree** | **Deprecated** | **Index** | **Help** | | |  |
| PREV   NEXT | **FRAMES**    **NO FRAMES**     **All Classes** |


---
