# Supplementary material for: BSim: An Agent-Based Tool for Modeling Bacterial Populations in Systems and Synthetic Biology
Source: PLoS One. 2012 Aug 24;7(8):e42790. doi: 10.1371/journal.pone.0042790 (PMC3427305; doi:10.1371/journal.pone.0042790)
Supplement: Software S1 — Snapshot of the BSim software from 18th July 2012. For the latest version see: http://bsim-bccs.sf.net. The BSim software requires Java version 1.6 or higher. (ZIP) [file pone.0042790.s014.zip › BSimSoftware/docs/javadoc/bsim/class-use/BSimOctreeField.html]

Uses of Class bsim.BSimOctreeField


---


|  |  |  |  |  |  |  |  |  |  |  |
| --- | --- | --- | --- | --- | --- | --- | --- | --- | --- | --- |
| |  |  |  |  |  |  |  |  | | --- | --- | --- | --- | --- | --- | --- | --- | | **Overview** | **Package** | **Class** | **Use** | **Tree** | **Deprecated** | **Index** | **Help** | | |  |
| PREV   NEXT | **FRAMES**    **NO FRAMES**     **All Classes** |


---


## **Uses of Class bsim.BSimOctreeField**

| Packages that use BSimOctreeField | |
| --- | --- |
| **bsim** |  |
| **bsim.draw** |  |
| **bsim.geometry** |  |

| Uses of BSimOctreeField in bsim | |
| --- | --- |

| Fields in bsim declared as BSimOctreeField | |
| --- | --- |
| `protected  BSimOctreeField` | `BSimOctreeField.parent`             Parent of node, for root this is null. |
| `protected  BSimOctreeField[]` | `BSimOctreeField.subNodes`             subNodes of octree, these can have subnodes of their own. |

| Methods in bsim that return BSimOctreeField | |
| --- | --- |
| `BSimOctreeField` | `BSimOctreeField.getsubNode(int i)`             Return the subNode (i is index of subnode). |
| `BSimOctreeField` | `BSimOctreeField.nodeFinder(BSimOctreeField t, javax.vecmath.Vector3d location)`             Gets a subnode of given index from lowest depth. |

| Methods in bsim with parameters of type BSimOctreeField | |
| --- | --- |
| `void` | `BSimOctreeField.colorFromCentre(BSimOctreeField t)`             Sets the nodeColor value as a function of the position of octree, useful for troubleshooting. |
| `void` | `BSimOctreeField.decay(BSimOctreeField t, double decayRate, double Dt)`             Decays the chemical field in an octreeField Node,visits each node in the tree structure using a post-order traverse. |
| `void` | `BSimOctreeField.diffuse(BSimOctreeField t, double diffusivity, double Dt, int depth)`             Diffuses chemicals through whole the octreeField structure, using Fick's law to determine how much of the chemical gets pushed into neighboring nodes over each time iteration |
| `static void` | `BSimOctreeField.inOrderfull(BSimOctreeField t)`             In-Order traverse, traverses from the deepest subnode, to the root and then back down to other deep nodes. |
| `BSimOctreeField` | `BSimOctreeField.nodeFinder(BSimOctreeField t, javax.vecmath.Vector3d location)`             Gets a subnode of given index from lowest depth. |
| `static void` | `BSimOctreeField.postOrderfull(BSimOctreeField t)`             Post-Order traverse with visit function. |
| `static void` | `BSimOctreeField.preOrderfull(BSimOctreeField t)`             Pre-Order full traverse - traverses from the root, a direction to the deepest subnode, back to the node, and then down into other roots. |
| `void` | `BSimOctreeField.setNodestoMesh(BSimMesh theMesh, BSimOctreeField t)`             Fits octreeField against a mesh and splits into subNodes when there is a collision with the mesh boundary Creates a finer octree structure each time this function is called. |
| `void` | `BSimOctreeField.visit(BSimOctreeField t)`             The visit method simply prints the location and depth of a node, useful for troubleshooting. |

| Uses of BSimOctreeField in bsim.draw | |
| --- | --- |

| Methods in bsim.draw with parameters of type BSimOctreeField | |
| --- | --- |
| `void` | `BSimP3DDrawer.draw(BSimOctreeField t, java.awt.Color c, float alphaGrad)`             Draw a BSimOctreeField in given colour. |
| `void` | `BSimP3DDrawer.draw(BSimOctreeField t, float alphaGrad)`             Draw a BSimOctreeField. |

| Uses of BSimOctreeField in bsim.geometry | |
| --- | --- |

| Methods in bsim.geometry with parameters of type BSimOctreeField | |
| --- | --- |
| `static boolean` | `BSimMeshUtils.intersectTriOctreeNode(BSimTriangle t, BSimOctreeField n)`             Test for intersection of a triangle against an octree node |

---


|  |  |  |  |  |  |  |  |  |  |  |
| --- | --- | --- | --- | --- | --- | --- | --- | --- | --- | --- |
| |  |  |  |  |  |  |  |  | | --- | --- | --- | --- | --- | --- | --- | --- | | **Overview** | **Package** | **Class** | **Use** | **Tree** | **Deprecated** | **Index** | **Help** | | |  |
| PREV   NEXT | **FRAMES**    **NO FRAMES**     **All Classes** |


---
