# Supplementary material for: BSim: An Agent-Based Tool for Modeling Bacterial Populations in Systems and Synthetic Biology
Source: PLoS One. 2012 Aug 24;7(8):e42790. doi: 10.1371/journal.pone.0042790 (PMC3427305; doi:10.1371/journal.pone.0042790)
Supplement: Software S1 — Snapshot of the BSim software from 18th July 2012. For the latest version see: http://bsim-bccs.sf.net. The BSim software requires Java version 1.6 or higher. (ZIP) [file pone.0042790.s014.zip › BSimSoftware/docs/javadoc/bsim/class-use/BSimThreadedTickerWorker.html]

Uses of Class bsim.BSimThreadedTickerWorker


---


|  |  |  |  |  |  |  |  |  |  |  |
| --- | --- | --- | --- | --- | --- | --- | --- | --- | --- | --- |
| |  |  |  |  |  |  |  |  | | --- | --- | --- | --- | --- | --- | --- | --- | | **Overview** | **Package** | **Class** | **Use** | **Tree** | **Deprecated** | **Index** | **Help** | | |  |
| PREV   NEXT | **FRAMES**    **NO FRAMES**     **All Classes** |


---


## **Uses of Class bsim.BSimThreadedTickerWorker**

| Packages that use BSimThreadedTickerWorker | |
| --- | --- |
| **bsim** |  |

| Uses of BSimThreadedTickerWorker in bsim | |
| --- | --- |

| Fields in bsim declared as BSimThreadedTickerWorker | |
| --- | --- |
| `protected  BSimThreadedTickerWorker` | `BSimThreadedTicker.myWorker`             A local working for the main thread to call directly. |

| Fields in bsim with type parameters of type BSimThreadedTickerWorker | |
| --- | --- |
| `protected  java.util.Vector<BSimThreadedTickerWorker>` | `BSimThreadedTicker.workers`             List of workers to call upon. |

| Methods in bsim that return BSimThreadedTickerWorker | |
| --- | --- |
| `abstract  BSimThreadedTickerWorker` | `BSimThreadedTicker.createWorker(int threadID, int threads)`             For the user to overwrite to create suitable workers for this ticker. |

---


|  |  |  |  |  |  |  |  |  |  |  |
| --- | --- | --- | --- | --- | --- | --- | --- | --- | --- | --- |
| |  |  |  |  |  |  |  |  | | --- | --- | --- | --- | --- | --- | --- | --- | | **Overview** | **Package** | **Class** | **Use** | **Tree** | **Deprecated** | **Index** | **Help** | | |  |
| PREV   NEXT | **FRAMES**    **NO FRAMES**     **All Classes** |


---
