# Supplementary material for: BSim: An Agent-Based Tool for Modeling Bacterial Populations in Systems and Synthetic Biology
Source: PLoS One. 2012 Aug 24;7(8):e42790. doi: 10.1371/journal.pone.0042790 (PMC3427305; doi:10.1371/journal.pone.0042790)
Supplement: Software S1 — Snapshot of the BSim software from 18th July 2012. For the latest version see: http://bsim-bccs.sf.net. The BSim software requires Java version 1.6 or higher. (ZIP) [file pone.0042790.s014.zip › BSimSoftware/docs/javadoc/bsim/class-use/OctreeNode.html]

Uses of Class bsim.OctreeNode


---


|  |  |  |  |  |  |  |  |  |  |  |
| --- | --- | --- | --- | --- | --- | --- | --- | --- | --- | --- |
| |  |  |  |  |  |  |  |  | | --- | --- | --- | --- | --- | --- | --- | --- | | **Overview** | **Package** | **Class** | **Use** | **Tree** | **Deprecated** | **Index** | **Help** | | |  |
| PREV   NEXT | **FRAMES**    **NO FRAMES**     **All Classes** |


---


## **Uses of Class bsim.OctreeNode**

| Packages that use OctreeNode | |
| --- | --- |
| **bsim** |  |
| **bsim.draw** |  |
| **bsim.geometry** |  |

| Uses of OctreeNode in bsim | |
| --- | --- |

| Fields in bsim declared as OctreeNode | |
| --- | --- |
| `protected  OctreeNode` | `OctreeNode.parent`             This is the root of the Octree data structure. |
| `protected  OctreeNode[]` | `OctreeNode.subNodes`             subNodes of Octree, these can have subnodes of their own. |

| Methods in bsim that return OctreeNode | |
| --- | --- |
| `OctreeNode` | `OctreeNode.getsubNode(int i)`             Return the nodes subNode, i is index of subNode. |
| `OctreeNode` | `OctreeNode.NodeFinder(OctreeNode t, int depth)`             Gets a subnode of given index from lowest depth |

| Methods in bsim with parameters of type OctreeNode | |
| --- | --- |
| `void` | `OctreeNode.colorFromCentre(OctreeNode t)`             Sets the nodeColor value as a function of the position of octree, useful for troubleshooting. |
| `void` | `OctreeNode.decay(OctreeNode t, double decayRate, double Dt)`             Decays the chemical field in an octree Node,visits each node in the tree structure using a post-order traverse. |
| `void` | `OctreeNode.diffuse(OctreeNode t, double diffusivity, double Dt, int depth)`             Diffuses chemicals through whole the octree structure, using Fick's law to determine how much of the chemical gets pushed into neighboring nodes over each time iteration. |
| `static void` | `OctreeNode.inOrderfull(OctreeNode t)`             In-Order full traverse, traverses from the deepest subnode, to the root and then back down to other deep nodes. |
| `OctreeNode` | `OctreeNode.NodeFinder(OctreeNode t, int depth)`             Gets a subnode of given index from lowest depth |
| `static void` | `OctreeNode.postOrderfull(OctreeNode t)`             Post-Order traverse with visit function. |
| `static void` | `OctreeNode.preOrderfull(OctreeNode t)`             Pre-Order full traverse - traverses from the root, a direction to the deepest subnode, back to the node, and then down into other roots. |
| `void` | `OctreeNode.setNodestoMesh(BSimMesh theMesh, OctreeNode t)`             Takes an octree node and divides its subnodes in such a way that the nodes conform to a coarse grained version of the shape of the mesh. |
| `void` | `OctreeNode.visit(OctreeNode t)`             The visit method simply prints the location and depth of a node, useful for troubleshooting. |

| Uses of OctreeNode in bsim.draw | |
| --- | --- |

| Methods in bsim.draw with parameters of type OctreeNode | |
| --- | --- |
| `void` | `BSimP3DDrawer.draw(OctreeNode t, java.awt.Color c, float alphaGrad)`             Post order hierarchy display drawing function for Octree. |
| `void` | `BSimP3DDrawer.draw(OctreeNode t, float alphaGrad)`             Post order hierarchy display drawing - uses internal color value for individual nodes Not the fastest while rendering.... |

| Uses of OctreeNode in bsim.geometry | |
| --- | --- |

| Methods in bsim.geometry with parameters of type OctreeNode | |
| --- | --- |
| `static boolean` | `BSimMeshUtils.intersectTriOctreeNode(BSimTriangle t, OctreeNode n)`             Test for intersection of a triangle against an octree node |

---


|  |  |  |  |  |  |  |  |  |  |  |
| --- | --- | --- | --- | --- | --- | --- | --- | --- | --- | --- |
| |  |  |  |  |  |  |  |  | | --- | --- | --- | --- | --- | --- | --- | --- | | **Overview** | **Package** | **Class** | **Use** | **Tree** | **Deprecated** | **Index** | **Help** | | |  |
| PREV   NEXT | **FRAMES**    **NO FRAMES**     **All Classes** |


---
