# Supplementary material for: BSim: An Agent-Based Tool for Modeling Bacterial Populations in Systems and Synthetic Biology
Source: PLoS One. 2012 Aug 24;7(8):e42790. doi: 10.1371/journal.pone.0042790 (PMC3427305; doi:10.1371/journal.pone.0042790)
Supplement: Software S1 — Snapshot of the BSim software from 18th July 2012. For the latest version see: http://bsim-bccs.sf.net. The BSim software requires Java version 1.6 or higher. (ZIP) [file pone.0042790.s014.zip › BSimSoftware/docs/javadoc/bsim/dde/BSimDdeSolver.html]

BSimDdeSolver


---


|  |  |  |  |  |  |  |  |  |  |  |
| --- | --- | --- | --- | --- | --- | --- | --- | --- | --- | --- |
| |  |  |  |  |  |  |  |  | | --- | --- | --- | --- | --- | --- | --- | --- | | **Overview** | **Package** | **Class** | **Use** | **Tree** | **Deprecated** | **Index** | **Help** | | |  |
| PREV CLASS   **NEXT CLASS** | **FRAMES**    **NO FRAMES**     **All Classes** |
| SUMMARY: NESTED | FIELD | CONSTR | METHOD | DETAIL: FIELD | CONSTR | METHOD |


---


## bsim.dde Class BSimDdeSolver

```
java.lang.Object
  bsim.dde.BSimDdeSolver
```

---

``` public class BSimDdeSolver extends java.lang.Object ```

Solver routines for numerical simulation of DDEs (Fixed time-step).
These make use of basic ODE solvers and so stability is not ensured
in all cases. Be sure to run simulations with smaller time steps to
check that the results are accurate.

---

| **Constructor Summary** | |
| --- | --- |
| `BSimDdeSolver()` |


| **Method Summary** | |
| --- | --- |
| `static java.util.Vector<double[]>` | `euler(BSimDdeSystem ddes, double t, java.util.Vector<double[]> ys, double h)`             Numerically solve an DDE system with Euler's method. |
| `static double[]` | `getDelayedState(java.util.Vector<double[]> ys, double h, double delay)`             Calculates a delayed (historic) state. |
| `static java.util.Vector<double[]>` | `getInitialState(BSimDdeSystem ddes, double h)`             Create an initial state, including history. |
| `static java.util.Vector<double[]>` | `rungeKutta23(BSimDdeSystem ddes, double t, java.util.Vector<double[]> ys, double h)`             Numerically solve an DDE system with 2nd order Runge-Kutta method. |
| `static java.util.Vector<double[]>` | `rungeKutta45(BSimDdeSystem ddes, double t, java.util.Vector<double[]> ys, double h)`             Numerically solve an DDE system with 4th order Runge-Kutta method. |
| `static void` | `shiftState(java.util.Vector<double[]> ys)`             Shifts all elements in the state vector by one timestep. |

| **Methods inherited from class java.lang.Object** |
| --- |
| `clone, equals, finalize, getClass, hashCode, notify, notifyAll, toString, wait, wait, wait` |

| **Constructor Detail** |
| --- |

### BSimDdeSolver

```
public BSimDdeSolver()
```


| **Method Detail** |
| --- |

### getInitialState

```
public static java.util.Vector<double[]> getInitialState(BSimDdeSystem ddes,
                                                         double h)
```

:   Create an initial state, including history.

    :   **Parameters:**: `ddes` - The DDE system.: `h` - Timestep. **Returns:**: Initial state vector.

---


### getDelayedState

```
public static double[] getDelayedState(java.util.Vector<double[]> ys,
                                       double h,
                                       double delay)
```

:   Calculates a delayed (historic) state. Should be used by the DDE system
    when retrieving historic states of the system.

    :   **Parameters:**: `ys` - The state vector.: `h` - Timestep: `delay` - Required delayed state vector **Returns:**: Delayed state vector.

---


### shiftState

```
public static void shiftState(java.util.Vector<double[]> ys)
```

:   Shifts all elements in the state vector by one timestep.
    Looses last historic state vector.

    :   **Parameters:**: `ys` - The state vector.

---


### euler

```
public static java.util.Vector<double[]> euler(BSimDdeSystem ddes,
                                               double t,
                                               java.util.Vector<double[]> ys,
                                               double h)
```

:   Numerically solve an DDE system with Euler's method.

    :   **Parameters:**: `odes` - The `BSimDdeSystem` to solve.: `t` - Independent variable.: `ys` - Vector of dependent variables including history.: `h` - Time step for ode solution. **Returns:**: Vector of dependent variables at next time step including history.

---


### rungeKutta23

```
public static java.util.Vector<double[]> rungeKutta23(BSimDdeSystem ddes,
                                                      double t,
                                                      java.util.Vector<double[]> ys,
                                                      double h)
```

:   Numerically solve an DDE system with 2nd order Runge-Kutta method.

    :   **Parameters:**: `odes` - The `BSimDdeSystem` to solve.: `t` - Independent variable.: `y` - Vector of dependent variables.: `h` - Time step for ode solution. **Returns:**: Vector of dependent variables at next time step.

---


### rungeKutta45

```
public static java.util.Vector<double[]> rungeKutta45(BSimDdeSystem ddes,
                                                      double t,
                                                      java.util.Vector<double[]> ys,
                                                      double h)
```

:   Numerically solve an DDE system with 4th order Runge-Kutta method.

    :   **Parameters:**: `odes` - The `BSimDdeSystem` to solve.: `t` - Independent variable.: `y` - Vector of dependent variables.: `h` - Time step for ode solution. **Returns:**: Vector of dependent variables at next time step.


---


|  |  |  |  |  |  |  |  |  |  |  |
| --- | --- | --- | --- | --- | --- | --- | --- | --- | --- | --- |
| |  |  |  |  |  |  |  |  | | --- | --- | --- | --- | --- | --- | --- | --- | | **Overview** | **Package** | **Class** | **Use** | **Tree** | **Deprecated** | **Index** | **Help** | | |  |
| PREV CLASS   **NEXT CLASS** | **FRAMES**    **NO FRAMES**     **All Classes** |
| SUMMARY: NESTED | FIELD | CONSTR | METHOD | DETAIL: FIELD | CONSTR | METHOD |


---
