# Supplementary material for: BSim: An Agent-Based Tool for Modeling Bacterial Populations in Systems and Synthetic Biology
Source: PLoS One. 2012 Aug 24;7(8):e42790. doi: 10.1371/journal.pone.0042790 (PMC3427305; doi:10.1371/journal.pone.0042790)
Supplement: Software S1 — Snapshot of the BSim software from 18th July 2012. For the latest version see: http://bsim-bccs.sf.net. The BSim software requires Java version 1.6 or higher. (ZIP) [file pone.0042790.s014.zip › BSimSoftware/docs/javadoc/bsim/dde/BSimDdeSystem.html]

BSimDdeSystem


---


|  |  |  |  |  |  |  |  |  |  |  |
| --- | --- | --- | --- | --- | --- | --- | --- | --- | --- | --- |
| |  |  |  |  |  |  |  |  | | --- | --- | --- | --- | --- | --- | --- | --- | | **Overview** | **Package** | **Class** | **Use** | **Tree** | **Deprecated** | **Index** | **Help** | | |  |
| **PREV CLASS**   NEXT CLASS | **FRAMES**    **NO FRAMES**     **All Classes** |
| SUMMARY: NESTED | FIELD | CONSTR | METHOD | DETAIL: FIELD | CONSTR | METHOD |


---


## bsim.dde Interface BSimDdeSystem

---

``` public interface BSimDdeSystem ```

Interface used for defining a system of DDEs.
Defines the DDEs, the number of equations and the initial conditions.

---

| **Method Summary** | |
| --- | --- |
| `double[]` | `derivativeSystem(double x, double[] y, java.util.Vector<double[]> ys)`             Defines a system of derivatives dy[0] = ..., dy[1] = ..., etc and returns dy[] |
| `double[]` | `getICs()`             Get the initial conditions: y1(0), y2(0), etc.. |
| `double` | `getMaxDelay()`             Get the maximum delay for the system |
| `int` | `getNumEq()`             Get the number of equations in the system (Corresponding to the number in derivativeSystem) |
| `void` | `setInitialHistory(java.util.Vector<double[]> ys)`             Sets the initial history when solving. |

| **Method Detail** |
| --- |

### derivativeSystem

```
double[] derivativeSystem(double x,
                          double[] y,
                          java.util.Vector<double[]> ys)
```

:   Defines a system of derivatives dy[0] = ..., dy[1] = ..., etc and returns dy[]

---


### getNumEq

```
int getNumEq()
```

:   Get the number of equations in the system
    (Corresponding to the number in derivativeSystem)

---


### getMaxDelay

```
double getMaxDelay()
```

:   Get the maximum delay for the system

    :   **Returns:**: maximum delay

---


### getICs

```
double[] getICs()
```

:   Get the initial conditions: y1(0), y2(0), etc..

---


### setInitialHistory

```
void setInitialHistory(java.util.Vector<double[]> ys)
```

:   Sets the initial history when solving.

    :   **Parameters:**: `ys` - Vector of historic states (double[])


---


|  |  |  |  |  |  |  |  |  |  |  |
| --- | --- | --- | --- | --- | --- | --- | --- | --- | --- | --- |
| |  |  |  |  |  |  |  |  | | --- | --- | --- | --- | --- | --- | --- | --- | | **Overview** | **Package** | **Class** | **Use** | **Tree** | **Deprecated** | **Index** | **Help** | | |  |
| **PREV CLASS**   NEXT CLASS | **FRAMES**    **NO FRAMES**     **All Classes** |
| SUMMARY: NESTED | FIELD | CONSTR | METHOD | DETAIL: FIELD | CONSTR | METHOD |


---
