# Supplementary material for: BSim: An Agent-Based Tool for Modeling Bacterial Populations in Systems and Synthetic Biology
Source: PLoS One. 2012 Aug 24;7(8):e42790. doi: 10.1371/journal.pone.0042790 (PMC3427305; doi:10.1371/journal.pone.0042790)
Supplement: Software S1 — Snapshot of the BSim software from 18th July 2012. For the latest version see: http://bsim-bccs.sf.net. The BSim software requires Java version 1.6 or higher. (ZIP) [file pone.0042790.s014.zip › BSimSoftware/docs/javadoc/bsim/draw/BSimDrawer.html]

BSimDrawer


---


|  |  |  |  |  |  |  |  |  |  |  |
| --- | --- | --- | --- | --- | --- | --- | --- | --- | --- | --- |
| |  |  |  |  |  |  |  |  | | --- | --- | --- | --- | --- | --- | --- | --- | | **Overview** | **Package** | **Class** | **Use** | **Tree** | **Deprecated** | **Index** | **Help** | | |  |
| PREV CLASS   **NEXT CLASS** | **FRAMES**    **NO FRAMES**     **All Classes** |
| SUMMARY: NESTED | FIELD | CONSTR | METHOD | DETAIL: FIELD | CONSTR | METHOD |


---


## bsim.draw Class BSimDrawer

```
java.lang.Object
  bsim.draw.BSimDrawer
```

**Direct Known Subclasses:**: BSimP3DDrawer

---

``` public abstract class BSimDrawer extends java.lang.Object ```

Drawer base class.
Does not implement any drawing itself, but should be extended by the user.

---

| **Field Summary** | |
| --- | --- |
| `protected  int` | `height`             Height of the display (pixels). |
| `protected  BSim` | `sim`             The simulation. |
| `protected  int` | `width`             Width of the display (pixels). |


| **Constructor Summary** | |
| --- | --- |
| `BSimDrawer(BSim sim, int width, int height)`             Constructor for a drawer. |


| **Method Summary** | |
| --- | --- |
| `abstract  void` | `draw(java.awt.Graphics2D g)`             Abstract method to draw the simulation to a graphics context (to be overwritten by the user). |
| `int` | `getHeight()`             Return the height of the display (pixels). |
| `int` | `getWidth()`             Return the width of the display (pixels). |

| **Methods inherited from class java.lang.Object** |
| --- |
| `clone, equals, finalize, getClass, hashCode, notify, notifyAll, toString, wait, wait, wait` |

| **Field Detail** |
| --- |

### sim

```
protected BSim sim
```

:   The simulation.

---


### width

```
protected int width
```

:   Width of the display (pixels).

---


### height

```
protected int height
```

:   Height of the display (pixels).


| **Constructor Detail** |
| --- |

### BSimDrawer

```
public BSimDrawer(BSim sim,
                  int width,
                  int height)
```

:   Constructor for a drawer. Sets internal references and size of the display.

    **Parameters:**: `sim` - The simulation.: `width` - Width of the display (pixels).: `height` - Height of the display (pixels).


| **Method Detail** |
| --- |

### draw

```
public abstract void draw(java.awt.Graphics2D g)
```

:   Abstract method to draw the simulation to a graphics context (to be overwritten by the user).

    :   **Parameters:**: `g` - Graphics context to draw the scene to.

---


### getWidth

```
public int getWidth()
```

:   Return the width of the display (pixels).

---


### getHeight

```
public int getHeight()
```

:   Return the height of the display (pixels).


---


|  |  |  |  |  |  |  |  |  |  |  |
| --- | --- | --- | --- | --- | --- | --- | --- | --- | --- | --- |
| |  |  |  |  |  |  |  |  | | --- | --- | --- | --- | --- | --- | --- | --- | | **Overview** | **Package** | **Class** | **Use** | **Tree** | **Deprecated** | **Index** | **Help** | | |  |
| PREV CLASS   **NEXT CLASS** | **FRAMES**    **NO FRAMES**     **All Classes** |
| SUMMARY: NESTED | FIELD | CONSTR | METHOD | DETAIL: FIELD | CONSTR | METHOD |


---
