# Supplementary material for: BSim: An Agent-Based Tool for Modeling Bacterial Populations in Systems and Synthetic Biology
Source: PLoS One. 2012 Aug 24;7(8):e42790. doi: 10.1371/journal.pone.0042790 (PMC3427305; doi:10.1371/journal.pone.0042790)
Supplement: Software S1 — Snapshot of the BSim software from 18th July 2012. For the latest version see: http://bsim-bccs.sf.net. The BSim software requires Java version 1.6 or higher. (ZIP) [file pone.0042790.s014.zip › BSimSoftware/docs/javadoc/bsim/draw/BSimP3DDrawer.html]

BSimP3DDrawer


---


|  |  |  |  |  |  |  |  |  |  |  |
| --- | --- | --- | --- | --- | --- | --- | --- | --- | --- | --- |
| |  |  |  |  |  |  |  |  | | --- | --- | --- | --- | --- | --- | --- | --- | | **Overview** | **Package** | **Class** | **Use** | **Tree** | **Deprecated** | **Index** | **Help** | | |  |
| **PREV CLASS**   NEXT CLASS | **FRAMES**    **NO FRAMES**     **All Classes** |
| SUMMARY: NESTED | FIELD | CONSTR | METHOD | DETAIL: FIELD | CONSTR | METHOD |


---


## bsim.draw Class BSimP3DDrawer

```
java.lang.Object
  bsim.draw.BSimDrawer
      bsim.draw.BSimP3DDrawer
```

---

``` public abstract class BSimP3DDrawer extends BSimDrawer ```

Scene preview and visualisation renderer (extends BSimDrawer). Uses
Processing3D libraries to render the scene. The resulting image is
then drawn to a native Java 2D graphics context which can be used
within a GUI.

---

| **Field Summary** | |
| --- | --- |
| `protected  javax.vecmath.Vector3d` | `bound`             Size of the simulation. |
| `protected  javax.vecmath.Vector3d` | `boundCentre`             Centre of the simulation. |
| `protected  processing.core.PFont` | `font`             Font used when rendering text. |
| `protected  processing.core.PGraphics3D` | `p3d`             Processing graphics context used for drawing. |

| **Fields inherited from class bsim.draw.BSimDrawer** |
| --- |
| `height, sim, width` |


| **Constructor Summary** | |
| --- | --- |
| `BSimP3DDrawer(BSim sim, int width, int height)`             Default constructor for initialising a Processing3D rendering context. |


| **Method Summary** | |
| --- | --- |
| `void` | `boundaries()`             Draw the default cuboid boundary of the simulation as a partially transparent box with a wireframe outline surrounding it. |
| `void` | `boundaryOutline()`             Draw the default cuboid boundary of the simulation as a wireframe outline. |
| `void` | `draw(BSimChemicalField field, java.awt.Color c, double alphaGrad, double alphaMax)`             Draws a chemical field structure based on its defined parameters, with custom transparency (alpha) parameters. |
| `void` | `draw(BSimChemicalField field, java.awt.Color c, float alphaGrad)`             Draw a chemical field structure based on its defined parameters (default alpha). |
| `void` | `draw(BSimMesh mesh, java.awt.Color c, double normalScaleFactor)`             Draw a mesh with a given colour (draws each triangle of the mesh individually). |
| `void` | `draw(BSimMesh mesh, double normalScaleFactor)`             Draw a mesh, default colour. |
| `void` | `draw(BSimOctreeField t, java.awt.Color c, float alphaGrad)`             Draw a BSimOctreeField in given colour. |
| `void` | `draw(BSimOctreeField t, float alphaGrad)`             Draw a BSimOctreeField. |
| `void` | `draw(BSimParticle p, java.awt.Color c)`             Draw a BSimParticle as a point if it is very small (radius < 1), or a sphere otherwise. |
| `void` | `draw(BSimVesicle v, java.awt.Color c)`             Draw a vesicle as a pixel surrounded by a 'halo' to make it easier to spot. |
| `void` | `draw(java.awt.Graphics2D g)`             Render all simulation and scene elements to the Processing3D graphics context 'p3d' (effectively the render buffer), then draw the rendered contents to the native Java graphics context. |
| `void` | `point(javax.vecmath.Vector3d position, java.awt.Color c)`             Draw a point (pixel); parametrised helper function. |
| `abstract  void` | `scene(processing.core.PGraphics3D p3d)`             Draws remaining scene objects to the PGraphics3D object. |
| `void` | `sphere(javax.vecmath.Vector3d position, double radius, java.awt.Color c, int alpha)`             Draw sphere; helper function which draws a parametrised sphere. |
| `void` | `time()`             Draw the formatted simulation time to screen. |
| `void` | `vector(javax.vecmath.Vector3d origin, javax.vecmath.Vector3d theVector, double scaleFactor, java.awt.Color c)`             Draw a 'vector' originating at a point, represented by a line. |
| `void` | `vertex(javax.vecmath.Vector3d newPoint)`             Define a p3d vertex when constructing shapes, directly from a Point3d. |

| **Methods inherited from class bsim.draw.BSimDrawer** |
| --- |
| `getHeight, getWidth` |

| **Methods inherited from class java.lang.Object** |
| --- |
| `clone, equals, finalize, getClass, hashCode, notify, notifyAll, toString, wait, wait, wait` |

| **Field Detail** |
| --- |

### p3d

```
protected processing.core.PGraphics3D p3d
```

:   Processing graphics context used for drawing.

---


### font

```
protected processing.core.PFont font
```

:   Font used when rendering text.

---


### bound

```
protected javax.vecmath.Vector3d bound
```

:   Size of the simulation.

---


### boundCentre

```
protected javax.vecmath.Vector3d boundCentre
```

:   Centre of the simulation. Used for camera positioning.


| **Constructor Detail** |
| --- |

### BSimP3DDrawer

```
public BSimP3DDrawer(BSim sim,
                     int width,
                     int height)
```

:   Default constructor for initialising a Processing3D rendering context.

    **Parameters:**: `sim` - The simulation we wish to render.: `width` - The desired horizontal resolution (pixels).: `height` - The desired vertical resolution (pixels).


| **Method Detail** |
| --- |

### draw

```
public void draw(java.awt.Graphics2D g)
```

:   Render all simulation and scene elements to the Processing3D graphics
    context 'p3d' (effectively the render buffer), then draw the rendered
    contents to the native Java graphics context.

    :   **Specified by:**: `draw` in class `BSimDrawer`
    :   **Parameters:**: `g` - The native Java graphics context to which we wish to draw our rendered scene.

---


### scene

```
public abstract void scene(processing.core.PGraphics3D p3d)
```

:   Draws remaining scene objects to the PGraphics3D object.
    Abstract method that should be overridden in a BSim\_X\_Example to render objects
    that are specific to a simulation.

---


### boundaries

```
public void boundaries()
```

:   Draw the default cuboid boundary of the simulation as a partially transparent box
    with a wireframe outline surrounding it.

---


### boundaryOutline

```
public void boundaryOutline()
```

:   Draw the default cuboid boundary of the simulation as a wireframe outline.

---


### time

```
public void time()
```

:   Draw the formatted simulation time to screen.

---


### draw

```
public void draw(BSimParticle p,
                 java.awt.Color c)
```

:   Draw a BSimParticle as a point if it is very small (radius < 1), or a sphere otherwise.

    :   **Parameters:**: `p` - The BSimParticle to be rendered.: `c` - The desired colour of the particle.

---


### draw

```
public void draw(BSimVesicle v,
                 java.awt.Color c)
```

:   Draw a vesicle as a pixel surrounded by a 'halo' to make it easier to spot.

    :   **Parameters:**: `v` - The BSimVesicle to be rendered.: `c` - The desired vesicle colour.

---


### draw

```
public void draw(BSimMesh mesh,
                 java.awt.Color c,
                 double normalScaleFactor)
```

:   Draw a mesh with a given colour (draws each triangle of the mesh individually).
    Also draws face normals as a red line from the face.

    :   **Parameters:**: `mesh` - The mesh you want to draw...: `c` - Mesh face colour.: `normalScaleFactor` - Scale factor for normal vector length. Set to zero to disable normal drawing.

---


### draw

```
public void draw(BSimMesh mesh,
                 double normalScaleFactor)
```

:   Draw a mesh, default colour.

    :   **Parameters:**: `mesh` - Mesh to draw.: `normalScaleFactor` - Scaling factor.

---


### vector

```
public void vector(javax.vecmath.Vector3d origin,
                   javax.vecmath.Vector3d theVector,
                   double scaleFactor,
                   java.awt.Color c)
```

:   Draw a 'vector' originating at a point, represented by a line.

    :   **Parameters:**: `origin` - Point from which the vector originates.: `theVector` - The vector.: `scaleFactor` - Scalar by which the vector is multiplied for drawing purposes.

---


### vertex

```
public void vertex(javax.vecmath.Vector3d newPoint)
```

:   Define a p3d vertex when constructing shapes, directly from a Point3d.

    :   **Parameters:**: `newPoint` - The Point3d representing the vertex coordinates.

---


### sphere

```
public void sphere(javax.vecmath.Vector3d position,
                   double radius,
                   java.awt.Color c,
                   int alpha)
```

:   Draw sphere; helper function which draws a parametrised sphere.

    :   **Parameters:**: `position` - The Cartesian coordinates of the sphere's centre in 3-D space.: `radius` - Sphere radius.: `c` - Colour of the sphere.: `alpha` - Sphere transparency.

---


### point

```
public void point(javax.vecmath.Vector3d position,
                  java.awt.Color c)
```

:   Draw a point (pixel); parametrised helper function.

    :   **Parameters:**: `position` - The Cartesian coordinates of the point in 3-D space.: `c` - The colour of the point.

---


### draw

```
public void draw(BSimChemicalField field,
                 java.awt.Color c,
                 double alphaGrad,
                 double alphaMax)
```

:   Draws a chemical field structure based on its defined parameters, with custom transparency (alpha) parameters.

    :   **Parameters:**: `field` - The chemical field structure to be rendered.: `c` - Desired colour of the chemical field.: `alphaGrad` - Alpha per unit concentration of the field.: `alphaMax` - Maximum alpha value (enables better viewing).

---


### draw

```
public void draw(BSimChemicalField field,
                 java.awt.Color c,
                 float alphaGrad)
```

:   Draw a chemical field structure based on its defined parameters (default alpha).

    :   **Parameters:**: `field` - The chemical field to be drawn.: `c` - The desired colour.: `alphaGrad` - The alpha-per-unit-concentration.

---


### draw

```
public void draw(BSimOctreeField t,
                 java.awt.Color c,
                 float alphaGrad)
```

:   Draw a BSimOctreeField in given colour. Post order hierarchy used for drawing.

    :   **Parameters:**: `t` - Octree to be drawn.: `c` - Desired colour.: `alphaGrad` - The alpha-per-unit-concentration.

---


### draw

```
public void draw(BSimOctreeField t,
                 float alphaGrad)
```

:   Draw a BSimOctreeField. Post order hierarchy used for drawing.

    :   **Parameters:**: `t` - Octree to be drawn.: `alphaGrad` - The alpha-per-unit-concentration.


---


|  |  |  |  |  |  |  |  |  |  |  |
| --- | --- | --- | --- | --- | --- | --- | --- | --- | --- | --- |
| |  |  |  |  |  |  |  |  | | --- | --- | --- | --- | --- | --- | --- | --- | | **Overview** | **Package** | **Class** | **Use** | **Tree** | **Deprecated** | **Index** | **Help** | | |  |
| **PREV CLASS**   NEXT CLASS | **FRAMES**    **NO FRAMES**     **All Classes** |
| SUMMARY: NESTED | FIELD | CONSTR | METHOD | DETAIL: FIELD | CONSTR | METHOD |


---
