# Supplementary material for: BSim: An Agent-Based Tool for Modeling Bacterial Populations in Systems and Synthetic Biology
Source: PLoS One. 2012 Aug 24;7(8):e42790. doi: 10.1371/journal.pone.0042790 (PMC3427305; doi:10.1371/journal.pone.0042790)
Supplement: Software S1 — Snapshot of the BSim software from 18th July 2012. For the latest version see: http://bsim-bccs.sf.net. The BSim software requires Java version 1.6 or higher. (ZIP) [file pone.0042790.s014.zip › BSimSoftware/docs/javadoc/bsim/draw/class-use/BSimDrawer.html]

Uses of Class bsim.draw.BSimDrawer


---


|  |  |  |  |  |  |  |  |  |  |  |
| --- | --- | --- | --- | --- | --- | --- | --- | --- | --- | --- |
| |  |  |  |  |  |  |  |  | | --- | --- | --- | --- | --- | --- | --- | --- | | **Overview** | **Package** | **Class** | **Use** | **Tree** | **Deprecated** | **Index** | **Help** | | |  |
| PREV   NEXT | **FRAMES**    **NO FRAMES**     **All Classes** |


---


## **Uses of Class bsim.draw.BSimDrawer**

| Packages that use BSimDrawer | |
| --- | --- |
| **bsim** |  |
| **bsim.draw** |  |
| **bsim.export** |  |

| Uses of BSimDrawer in bsim | |
| --- | --- |

| Methods in bsim with parameters of type BSimDrawer | |
| --- | --- |
| `void` | `BSim.setDrawer(BSimDrawer bSimDrawer)`             Set the drawer to be used during simulation. |

| Uses of BSimDrawer in bsim.draw | |
| --- | --- |

| Subclasses of BSimDrawer in bsim.draw | |
| --- | --- |
| `class` | `BSimP3DDrawer`             Scene preview and visualisation renderer (extends BSimDrawer). |

| Uses of BSimDrawer in bsim.export | |
| --- | --- |

| Fields in bsim.export declared as BSimDrawer | |
| --- | --- |
| `protected  BSimDrawer` | `BSimPngExporter.drawer`             Drawer to generate each image. |
| `protected  BSimDrawer` | `BSimMovExporter.drawer`             Drawer to generate a movie frame. |

| Constructors in bsim.export with parameters of type BSimDrawer | |
| --- | --- |
| `BSimMovExporter(BSim sim, BSimDrawer drawer, java.lang.String filename)`             Constructor for the movie exporter. |
| `BSimPngExporter(BSim sim, BSimDrawer drawer, java.lang.String directory)`             Constructor for the image exporter |

---


|  |  |  |  |  |  |  |  |  |  |  |
| --- | --- | --- | --- | --- | --- | --- | --- | --- | --- | --- |
| |  |  |  |  |  |  |  |  | | --- | --- | --- | --- | --- | --- | --- | --- | | **Overview** | **Package** | **Class** | **Use** | **Tree** | **Deprecated** | **Index** | **Help** | | |  |
| PREV   NEXT | **FRAMES**    **NO FRAMES**     **All Classes** |


---
