# Supplementary material for: BSim: An Agent-Based Tool for Modeling Bacterial Populations in Systems and Synthetic Biology
Source: PLoS One. 2012 Aug 24;7(8):e42790. doi: 10.1371/journal.pone.0042790 (PMC3427305; doi:10.1371/journal.pone.0042790)
Supplement: Software S1 — Snapshot of the BSim software from 18th July 2012. For the latest version see: http://bsim-bccs.sf.net. The BSim software requires Java version 1.6 or higher. (ZIP) [file pone.0042790.s014.zip › BSimSoftware/docs/javadoc/bsim/export/BSimExporter.html]

BSimExporter


---


|  |  |  |  |  |  |  |  |  |  |  |
| --- | --- | --- | --- | --- | --- | --- | --- | --- | --- | --- |
| |  |  |  |  |  |  |  |  | | --- | --- | --- | --- | --- | --- | --- | --- | | **Overview** | **Package** | **Class** | **Use** | **Tree** | **Deprecated** | **Index** | **Help** | | |  |
| PREV CLASS   **NEXT CLASS** | **FRAMES**    **NO FRAMES**     **All Classes** |
| SUMMARY: NESTED | FIELD | CONSTR | METHOD | DETAIL: FIELD | CONSTR | METHOD |


---


## bsim.export Class BSimExporter

```
java.lang.Object
  bsim.export.BSimExporter
```

**Direct Known Subclasses:**: BSimLogger, BSimMovExporter, BSimPngExporter

---

``` public abstract class BSimExporter extends java.lang.Object ```

Exporter base class. @see BSim#export()

---

| **Field Summary** | |
| --- | --- |
| `protected  double` | `dt`             Timestp. |
| `protected  BSim` | `sim`             Associated simulation. |


| **Constructor Summary** | |
| --- | --- |
| `BSimExporter(BSim sim)`             Constructor of a basic exporter. |


| **Method Summary** | |
| --- | --- |
| `abstract  void` | `after()`             Called after a simulation finishes (overwrite). |
| `abstract  void` | `before()`             Called before a simulation starts (overwrite). |
| `abstract  void` | `during()`             Called each timestep (overwrite). |
| `double` | `getDt()`             Return the timestep. |
| `void` | `setDt(double d)`             Set the time interval that the exporter is called. |

| **Methods inherited from class java.lang.Object** |
| --- |
| `clone, equals, finalize, getClass, hashCode, notify, notifyAll, toString, wait, wait, wait` |

| **Field Detail** |
| --- |

### sim

```
protected BSim sim
```

:   Associated simulation.

---


### dt

```
protected double dt
```

:   Timestp.


| **Constructor Detail** |
| --- |

### BSimExporter

```
public BSimExporter(BSim sim)
```

:   Constructor of a basic exporter. Abstract class to be extended
    for particular need.

    **Parameters:**: `sim` - Associated simulation.


| **Method Detail** |
| --- |

### before

```
public abstract void before()
```

:   Called before a simulation starts (overwrite).

---


### during

```
public abstract void during()
```

:   Called each timestep (overwrite).

---


### after

```
public abstract void after()
```

:   Called after a simulation finishes (overwrite).

---


### setDt

```
public void setDt(double d)
```

:   Set the time interval that the exporter is called.
    The exporter is called every d seconds in simulation time.
    Defaults to sim.getDt().

---


### getDt

```
public double getDt()
```

:   Return the timestep.


---


|  |  |  |  |  |  |  |  |  |  |  |
| --- | --- | --- | --- | --- | --- | --- | --- | --- | --- | --- |
| |  |  |  |  |  |  |  |  | | --- | --- | --- | --- | --- | --- | --- | --- | | **Overview** | **Package** | **Class** | **Use** | **Tree** | **Deprecated** | **Index** | **Help** | | |  |
| PREV CLASS   **NEXT CLASS** | **FRAMES**    **NO FRAMES**     **All Classes** |
| SUMMARY: NESTED | FIELD | CONSTR | METHOD | DETAIL: FIELD | CONSTR | METHOD |


---
