# Supplementary material for: BSim: An Agent-Based Tool for Modeling Bacterial Populations in Systems and Synthetic Biology
Source: PLoS One. 2012 Aug 24;7(8):e42790. doi: 10.1371/journal.pone.0042790 (PMC3427305; doi:10.1371/journal.pone.0042790)
Supplement: Software S1 — Snapshot of the BSim software from 18th July 2012. For the latest version see: http://bsim-bccs.sf.net. The BSim software requires Java version 1.6 or higher. (ZIP) [file pone.0042790.s014.zip › BSimSoftware/docs/javadoc/bsim/export/BSimLogger.html]

BSimLogger


---


|  |  |  |  |  |  |  |  |  |  |  |
| --- | --- | --- | --- | --- | --- | --- | --- | --- | --- | --- |
| |  |  |  |  |  |  |  |  | | --- | --- | --- | --- | --- | --- | --- | --- | | **Overview** | **Package** | **Class** | **Use** | **Tree** | **Deprecated** | **Index** | **Help** | | |  |
| **PREV CLASS**   **NEXT CLASS** | **FRAMES**    **NO FRAMES**     **All Classes** |
| SUMMARY: NESTED | FIELD | CONSTR | METHOD | DETAIL: FIELD | CONSTR | METHOD |


---


## bsim.export Class BSimLogger

```
java.lang.Object
  bsim.export.BSimExporter
      bsim.export.BSimLogger
```

---

``` public abstract class BSimLogger extends BSimExporter ```

Text file exporter.
Can be used to export simulation data to a text file. The during() method
must be overwritten to write the required data to file.

---

| **Field Summary** | |
| --- | --- |
| `protected  java.io.BufferedWriter` | `bufferedWriter`             Object to write output to. |
| `protected  java.lang.String` | `filename`             Filename of output. |

| **Fields inherited from class bsim.export.BSimExporter** |
| --- |
| `dt, sim` |


| **Constructor Summary** | |
| --- | --- |
| `BSimLogger(BSim sim, java.lang.String filename)`             Constructor for a file logger. |


| **Method Summary** | |
| --- | --- |
| `void` | `after()`             Called after a simulation ends. |
| `void` | `before()`             Called before a simulation starts. |
| `void` | `write(java.lang.String text)`             Writes text to the output file. |

| **Methods inherited from class bsim.export.BSimExporter** |
| --- |
| `during, getDt, setDt` |

| **Methods inherited from class java.lang.Object** |
| --- |
| `clone, equals, finalize, getClass, hashCode, notify, notifyAll, toString, wait, wait, wait` |

| **Field Detail** |
| --- |

### bufferedWriter

```
protected java.io.BufferedWriter bufferedWriter
```

:   Object to write output to.

---


### filename

```
protected java.lang.String filename
```

:   Filename of output.


| **Constructor Detail** |
| --- |

### BSimLogger

```
public BSimLogger(BSim sim,
                  java.lang.String filename)
```

:   Constructor for a file logger. Will output data to a specified file.

    **Parameters:**: `sim` - Associated simulation.: `filename` - Output filename.


| **Method Detail** |
| --- |

### before

```
public void before()
```

:   Called before a simulation starts. Can be extended by a user if necessary.

    :   **Specified by:**: `before` in class `BSimExporter`

---


### write

```
public void write(java.lang.String text)
```

:   Writes text to the output file.

    :   **Parameters:**: `text` - Text to write to file.

---


### after

```
public void after()
```

:   Called after a simulation ends. Can be extended by a user if necessary.

    :   **Specified by:**: `after` in class `BSimExporter`


---


|  |  |  |  |  |  |  |  |  |  |  |
| --- | --- | --- | --- | --- | --- | --- | --- | --- | --- | --- |
| |  |  |  |  |  |  |  |  | | --- | --- | --- | --- | --- | --- | --- | --- | | **Overview** | **Package** | **Class** | **Use** | **Tree** | **Deprecated** | **Index** | **Help** | | |  |
| **PREV CLASS**   **NEXT CLASS** | **FRAMES**    **NO FRAMES**     **All Classes** |
| SUMMARY: NESTED | FIELD | CONSTR | METHOD | DETAIL: FIELD | CONSTR | METHOD |


---
