# Supplementary material for: BSim: An Agent-Based Tool for Modeling Bacterial Populations in Systems and Synthetic Biology
Source: PLoS One. 2012 Aug 24;7(8):e42790. doi: 10.1371/journal.pone.0042790 (PMC3427305; doi:10.1371/journal.pone.0042790)
Supplement: Software S1 — Snapshot of the BSim software from 18th July 2012. For the latest version see: http://bsim-bccs.sf.net. The BSim software requires Java version 1.6 or higher. (ZIP) [file pone.0042790.s014.zip › BSimSoftware/docs/javadoc/bsim/export/BSimMovExporter.html]

BSimMovExporter


---


|  |  |  |  |  |  |  |  |  |  |  |
| --- | --- | --- | --- | --- | --- | --- | --- | --- | --- | --- |
| |  |  |  |  |  |  |  |  | | --- | --- | --- | --- | --- | --- | --- | --- | | **Overview** | **Package** | **Class** | **Use** | **Tree** | **Deprecated** | **Index** | **Help** | | |  |
| **PREV CLASS**   **NEXT CLASS** | **FRAMES**    **NO FRAMES**     **All Classes** |
| SUMMARY: NESTED | FIELD | CONSTR | METHOD | DETAIL: FIELD | CONSTR | METHOD |


---


## bsim.export Class BSimMovExporter

```
java.lang.Object
  bsim.export.BSimExporter
      bsim.export.BSimMovExporter
```

---

``` public class BSimMovExporter extends BSimExporter ```

Movie file exporter.

---

| **Field Summary** | |
| --- | --- |
| `protected  BSimDrawer` | `drawer`             Drawer to generate a movie frame. |
| `protected  java.lang.String` | `filename`             Filename of the output movie. |
| `protected  QuickTimeOutputStream` | `outputStream`             Movie that is output to. |
| `protected  int` | `speed`             Speed of the movie. |

| **Fields inherited from class bsim.export.BSimExporter** |
| --- |
| `dt, sim` |


| **Constructor Summary** | |
| --- | --- |
| `BSimMovExporter(BSim sim, BSimDrawer drawer, java.lang.String filename)`             Constructor for the movie exporter. |


| **Method Summary** | |
| --- | --- |
| `void` | `after()`             Called after the simulation ends. |
| `void` | `before()`             Called before the simulation starts. |
| `void` | `during()`             Called at each timestep of the simulation. |
| `void` | `setSpeed(int f)`             Speeds up the movie relative to simulation time by a factor f, that is, 1 second in the simulation will last 1/f seconds in the movie |

| **Methods inherited from class bsim.export.BSimExporter** |
| --- |
| `getDt, setDt` |

| **Methods inherited from class java.lang.Object** |
| --- |
| `clone, equals, finalize, getClass, hashCode, notify, notifyAll, toString, wait, wait, wait` |

| **Field Detail** |
| --- |

### outputStream

```
protected QuickTimeOutputStream outputStream
```

:   Movie that is output to.

---


### filename

```
protected java.lang.String filename
```

:   Filename of the output movie.

---


### speed

```
protected int speed
```

:   Speed of the movie.

---


### drawer

```
protected BSimDrawer drawer
```

:   Drawer to generate a movie frame.


| **Constructor Detail** |
| --- |

### BSimMovExporter

```
public BSimMovExporter(BSim sim,
                       BSimDrawer drawer,
                       java.lang.String filename)
```

:   Constructor for the movie exporter.

    **Parameters:**: `sim` - Associated simulation.: `drawer` - Drawer to generate movie frames.: `filename` - Output movie filename.


| **Method Detail** |
| --- |

### setSpeed

```
public void setSpeed(int f)
```

:   Speeds up the movie relative to simulation time by a factor f, that is,
    1 second in the simulation will last 1/f seconds in the movie

---


### before

```
public void before()
```

:   Called before the simulation starts.

    :   **Specified by:**: `before` in class `BSimExporter`

---


### during

```
public void during()
```

:   Called at each timestep of the simulation.

    :   **Specified by:**: `during` in class `BSimExporter`

---


### after

```
public void after()
```

:   Called after the simulation ends.

    :   **Specified by:**: `after` in class `BSimExporter`


---


|  |  |  |  |  |  |  |  |  |  |  |
| --- | --- | --- | --- | --- | --- | --- | --- | --- | --- | --- |
| |  |  |  |  |  |  |  |  | | --- | --- | --- | --- | --- | --- | --- | --- | | **Overview** | **Package** | **Class** | **Use** | **Tree** | **Deprecated** | **Index** | **Help** | | |  |
| **PREV CLASS**   **NEXT CLASS** | **FRAMES**    **NO FRAMES**     **All Classes** |
| SUMMARY: NESTED | FIELD | CONSTR | METHOD | DETAIL: FIELD | CONSTR | METHOD |


---
