# Supplementary material for: BSim: An Agent-Based Tool for Modeling Bacterial Populations in Systems and Synthetic Biology
Source: PLoS One. 2012 Aug 24;7(8):e42790. doi: 10.1371/journal.pone.0042790 (PMC3427305; doi:10.1371/journal.pone.0042790)
Supplement: Software S1 — Snapshot of the BSim software from 18th July 2012. For the latest version see: http://bsim-bccs.sf.net. The BSim software requires Java version 1.6 or higher. (ZIP) [file pone.0042790.s014.zip › BSimSoftware/docs/javadoc/bsim/export/BSimPngExporter.html]

BSimPngExporter


---


|  |  |  |  |  |  |  |  |  |  |  |
| --- | --- | --- | --- | --- | --- | --- | --- | --- | --- | --- |
| |  |  |  |  |  |  |  |  | | --- | --- | --- | --- | --- | --- | --- | --- | | **Overview** | **Package** | **Class** | **Use** | **Tree** | **Deprecated** | **Index** | **Help** | | |  |
| **PREV CLASS**   NEXT CLASS | **FRAMES**    **NO FRAMES**     **All Classes** |
| SUMMARY: NESTED | FIELD | CONSTR | METHOD | DETAIL: FIELD | CONSTR | METHOD |


---


## bsim.export Class BSimPngExporter

```
java.lang.Object
  bsim.export.BSimExporter
      bsim.export.BSimPngExporter
```

---

``` public class BSimPngExporter extends BSimExporter ```

Image file exporter.
Images are generated for each timestep and output to a given directory.
These are named with a timestamp and output in a PNG format.

---

| **Field Summary** | |
| --- | --- |
| `protected  java.lang.String` | `directory`             Directory to output images to. |
| `protected  BSimDrawer` | `drawer`             Drawer to generate each image. |

| **Fields inherited from class bsim.export.BSimExporter** |
| --- |
| `dt, sim` |


| **Constructor Summary** | |
| --- | --- |
| `BSimPngExporter(BSim sim, BSimDrawer drawer, java.lang.String directory)`             Constructor for the image exporter |


| **Method Summary** | |
| --- | --- |
| `void` | `after()`             Called after the simulation ends. |
| `void` | `before()`             Called before a simulation starts. |
| `void` | `during()`             Called at each timestep of the simulation. |

| **Methods inherited from class bsim.export.BSimExporter** |
| --- |
| `getDt, setDt` |

| **Methods inherited from class java.lang.Object** |
| --- |
| `clone, equals, finalize, getClass, hashCode, notify, notifyAll, toString, wait, wait, wait` |

| **Field Detail** |
| --- |

### directory

```
protected java.lang.String directory
```

:   Directory to output images to.

---


### drawer

```
protected BSimDrawer drawer
```

:   Drawer to generate each image.


| **Constructor Detail** |
| --- |

### BSimPngExporter

```
public BSimPngExporter(BSim sim,
                       BSimDrawer drawer,
                       java.lang.String directory)
```

:   Constructor for the image exporter

    **Parameters:**: `sim` - Associated simulation.: `drawer` - Drawer to generate images.: `directory` - Directory to output images to.


| **Method Detail** |
| --- |

### before

```
public void before()
```

:   Called before a simulation starts.

    :   **Specified by:**: `before` in class `BSimExporter`

---


### during

```
public void during()
```

:   Called at each timestep of the simulation.

    :   **Specified by:**: `during` in class `BSimExporter`

---


### after

```
public void after()
```

:   Called after the simulation ends.

    :   **Specified by:**: `after` in class `BSimExporter`


---


|  |  |  |  |  |  |  |  |  |  |  |
| --- | --- | --- | --- | --- | --- | --- | --- | --- | --- | --- |
| |  |  |  |  |  |  |  |  | | --- | --- | --- | --- | --- | --- | --- | --- | | **Overview** | **Package** | **Class** | **Use** | **Tree** | **Deprecated** | **Index** | **Help** | | |  |
| **PREV CLASS**   NEXT CLASS | **FRAMES**    **NO FRAMES**     **All Classes** |
| SUMMARY: NESTED | FIELD | CONSTR | METHOD | DETAIL: FIELD | CONSTR | METHOD |


---
