# Supplementary material for: BSim: An Agent-Based Tool for Modeling Bacterial Populations in Systems and Synthetic Biology
Source: PLoS One. 2012 Aug 24;7(8):e42790. doi: 10.1371/journal.pone.0042790 (PMC3427305; doi:10.1371/journal.pone.0042790)
Supplement: Software S1 — Snapshot of the BSim software from 18th July 2012. For the latest version see: http://bsim-bccs.sf.net. The BSim software requires Java version 1.6 or higher. (ZIP) [file pone.0042790.s014.zip › BSimSoftware/docs/javadoc/bsim/export/class-use/BSimExporter.html]

Uses of Class bsim.export.BSimExporter


---


|  |  |  |  |  |  |  |  |  |  |  |
| --- | --- | --- | --- | --- | --- | --- | --- | --- | --- | --- |
| |  |  |  |  |  |  |  |  | | --- | --- | --- | --- | --- | --- | --- | --- | | **Overview** | **Package** | **Class** | **Use** | **Tree** | **Deprecated** | **Index** | **Help** | | |  |
| PREV   NEXT | **FRAMES**    **NO FRAMES**     **All Classes** |


---


## **Uses of Class bsim.export.BSimExporter**

| Packages that use BSimExporter | |
| --- | --- |
| **bsim** |  |
| **bsim.export** |  |

| Uses of BSimExporter in bsim | |
| --- | --- |

| Methods in bsim with parameters of type BSimExporter | |
| --- | --- |
| `void` | `BSim.addExporter(BSimExporter e)`             Add an exporter to be called during simulation. |

| Uses of BSimExporter in bsim.export | |
| --- | --- |

| Subclasses of BSimExporter in bsim.export | |
| --- | --- |
| `class` | `BSimLogger`             Text file exporter. |
| `class` | `BSimMovExporter`             Movie file exporter. |
| `class` | `BSimPngExporter`             Image file exporter. |

---


|  |  |  |  |  |  |  |  |  |  |  |
| --- | --- | --- | --- | --- | --- | --- | --- | --- | --- | --- |
| |  |  |  |  |  |  |  |  | | --- | --- | --- | --- | --- | --- | --- | --- | | **Overview** | **Package** | **Class** | **Use** | **Tree** | **Deprecated** | **Index** | **Help** | | |  |
| PREV   NEXT | **FRAMES**    **NO FRAMES**     **All Classes** |


---
