# Supplementary material for: BSim: An Agent-Based Tool for Modeling Bacterial Populations in Systems and Synthetic Biology
Source: PLoS One. 2012 Aug 24;7(8):e42790. doi: 10.1371/journal.pone.0042790 (PMC3427305; doi:10.1371/journal.pone.0042790)
Supplement: Software S1 — Snapshot of the BSim software from 18th July 2012. For the latest version see: http://bsim-bccs.sf.net. The BSim software requires Java version 1.6 or higher. (ZIP) [file pone.0042790.s014.zip › BSimSoftware/docs/javadoc/bsim/export/quicktime/AtomDataOutputStream.html]

AtomDataOutputStream


---


|  |  |  |  |  |  |  |  |  |  |  |
| --- | --- | --- | --- | --- | --- | --- | --- | --- | --- | --- |
| |  |  |  |  |  |  |  |  | | --- | --- | --- | --- | --- | --- | --- | --- | | **Overview** | **Package** | **Class** | **Use** | **Tree** | **Deprecated** | **Index** | **Help** | | |  |
| PREV CLASS   **NEXT CLASS** | **FRAMES**    **NO FRAMES**     **All Classes** |
| SUMMARY: NESTED | FIELD | CONSTR | METHOD | DETAIL: FIELD | CONSTR | METHOD |


---


## bsim.export.quicktime Class AtomDataOutputStream

```
java.lang.Object
  java.io.OutputStream
      java.io.FilterOutputStream
          bsim.export.quicktime.AtomDataOutputStream
```

**All Implemented Interfaces:**: java.io.Closeable, java.io.Flushable

---

``` public class AtomDataOutputStream extends java.io.FilterOutputStream ```

This output stream filter supports common data types used inside
of QuickTime Atoms.

**Version:**
:   1.0.1 2008-06-22 Use ASCII instead of MacRoman for encoding
    type strings.
      
    1.0 Jun 15, 2008 Created.

**Author:**
:   Werner Randelshofer

---

| **Field Summary** | |
| --- | --- |
| `protected static long` | `MAC_TIMESTAMP_EPOCH` |
| `protected  long` | `written`             The number of bytes written to the data output stream so far. |

| **Fields inherited from class java.io.FilterOutputStream** |
| --- |
| `out` |


| **Constructor Summary** | |
| --- | --- |
| `AtomDataOutputStream(java.io.OutputStream out)` |


| **Method Summary** | |
| --- | --- |
| `protected  void` | `incCount(int value)`             Increases the written counter by the specified value until it reaches Long.MAX\_VALUE. |
| `long` | `size()`             Returns the current value of the counter `written`, the number of bytes written to this data output stream so far. |
| `void` | `write(byte[] b, int off, int len)`             Writes `len` bytes from the specified byte array starting at offset `off` to the underlying output stream. |
| `void` | `write(int b)`             Writes the specified byte (the low eight bits of the argument `b`) to the underlying output stream. |
| `void` | `writeBCD2(int v)`             Writes a `BCD2` to the underlying output stream. |
| `void` | `writeBCD4(int v)`             Writes a `BCD4` to the underlying output stream. |
| `void` | `writeByte(int v)`             Writes out a `byte` to the underlying output stream as a 1-byte value. |
| `void` | `writeFixed16D16(double f)`             Writes 32-bit fixed-point number divided as 16.16. |
| `void` | `writeFixed2D30(double f)`             Writes 32-bit fixed-point number divided as 2.30. |
| `void` | `writeFixed8D8(float f)`             Writes 16-bit fixed-point number divided as 8.8. |
| `void` | `writeInt(int v)`             Writes an `int` to the underlying output stream as four bytes, high byte first. |
| `void` | `writeLong(long v)` |
| `void` | `writeMacTimestamp(java.util.Date date)`             Writes a 32-bit Mac timestamp (seconds since 1902). |
| `void` | `writePString(java.lang.String s)`             Writes a Pascal String. |
| `void` | `writeShort(int v)`             Writes a signed 16 bit integer value. |
| `void` | `writeType(java.lang.String s)`             Writes an Atom Type identifier (4 bytes). |
| `void` | `writeUInt(long v)`             Writes an unsigned 32 bit integer value. |
| `void` | `writeUShort(int v)` |

| **Methods inherited from class java.io.FilterOutputStream** |
| --- |
| `close, flush, write` |

| **Methods inherited from class java.lang.Object** |
| --- |
| `clone, equals, finalize, getClass, hashCode, notify, notifyAll, toString, wait, wait, wait` |

| **Field Detail** |
| --- |

### MAC\_TIMESTAMP\_EPOCH

```
protected static final long MAC_TIMESTAMP_EPOCH
```

---


### written

```
protected long written
```

:   The number of bytes written to the data output stream so far.
    If this counter overflows, it will be wrapped to Integer.MAX\_VALUE.


| **Constructor Detail** |
| --- |

### AtomDataOutputStream

```
public AtomDataOutputStream(java.io.OutputStream out)
```


| **Method Detail** |
| --- |

### writeType

```
public void writeType(java.lang.String s)
               throws java.io.IOException
```

:   Writes an Atom Type identifier (4 bytes).

    :   **Parameters:**: `type` - A string with a length of 4 characters. **Throws:**: `java.io.IOException`

---


### writeByte

```
public final void writeByte(int v)
                     throws java.io.IOException
```

:   Writes out a `byte` to the underlying output stream as
    a 1-byte value. If no exception is thrown, the counter
    `written` is incremented by `1`.

    :   **Parameters:**: `v` - a `byte` value to be written. **Throws:**: `java.io.IOException` - if an I/O error occurs. **See Also:**: `FilterOutputStream.out`

---


### write

```
public void write(byte[] b,
                  int off,
                  int len)
           throws java.io.IOException
```

:   Writes `len` bytes from the specified byte array
    starting at offset `off` to the underlying output stream.
    If no exception is thrown, the counter `written` is
    incremented by `len`.

    :   **Overrides:**: `write` in class `java.io.FilterOutputStream`
    :   **Parameters:**: `b` - the data.: `off` - the start offset in the data.: `len` - the number of bytes to write. **Throws:**: `java.io.IOException` - if an I/O error occurs. **See Also:**: `FilterOutputStream.out`

---


### write

```
public void write(int b)
           throws java.io.IOException
```

:   Writes the specified byte (the low eight bits of the argument
    `b`) to the underlying output stream. If no exception
    is thrown, the counter `written` is incremented by
    `1`.

    Implements the `write` method of `OutputStream`.

    :   **Overrides:**: `write` in class `java.io.FilterOutputStream`
    :   **Parameters:**: `b` - the `byte` to be written. **Throws:**: `java.io.IOException` - if an I/O error occurs. **See Also:**: `FilterOutputStream.out`

---


### writeInt

```
public void writeInt(int v)
              throws java.io.IOException
```

:   Writes an `int` to the underlying output stream as four
    bytes, high byte first. If no exception is thrown, the counter
    `written` is incremented by `4`.

    :   **Parameters:**: `v` - an `int` to be written. **Throws:**: `java.io.IOException` - if an I/O error occurs. **See Also:**: `FilterOutputStream.out`

---


### writeUInt

```
public void writeUInt(long v)
               throws java.io.IOException
```

:   Writes an unsigned 32 bit integer value.

    :   **Parameters:**: `v` - The value **Throws:**: `java.io.IOException`

---


### writeShort

```
public void writeShort(int v)
                throws java.io.IOException
```

:   Writes a signed 16 bit integer value.

    :   **Parameters:**: `v` - The value **Throws:**: `java.io.IOException`

---


### writeBCD2

```
public void writeBCD2(int v)
               throws java.io.IOException
```

:   Writes a `BCD2` to the underlying output stream.

    :   **Parameters:**: `v` - an `int` to be written. **Throws:**: `java.io.IOException` - if an I/O error occurs. **See Also:**: `FilterOutputStream.out`

---


### writeBCD4

```
public void writeBCD4(int v)
               throws java.io.IOException
```

:   Writes a `BCD4` to the underlying output stream.

    :   **Parameters:**: `v` - an `int` to be written. **Throws:**: `java.io.IOException` - if an I/O error occurs. **See Also:**: `FilterOutputStream.out`

---


### writeMacTimestamp

```
public void writeMacTimestamp(java.util.Date date)
                       throws java.io.IOException
```

:   Writes a 32-bit Mac timestamp (seconds since 1902).

    :   **Parameters:**: `date` - **Throws:**: `java.io.IOException`

---


### writeFixed16D16

```
public void writeFixed16D16(double f)
                     throws java.io.IOException
```

:   Writes 32-bit fixed-point number divided as 16.16.

    :   **Parameters:**: `v` - an `int` to be written. **Throws:**: `java.io.IOException` - if an I/O error occurs. **See Also:**: `FilterOutputStream.out`

---


### writeFixed2D30

```
public void writeFixed2D30(double f)
                    throws java.io.IOException
```

:   Writes 32-bit fixed-point number divided as 2.30.

    :   **Parameters:**: `v` - an `int` to be written. **Throws:**: `java.io.IOException` - if an I/O error occurs. **See Also:**: `FilterOutputStream.out`

---


### writeFixed8D8

```
public void writeFixed8D8(float f)
                   throws java.io.IOException
```

:   Writes 16-bit fixed-point number divided as 8.8.

    :   **Parameters:**: `v` - an `int` to be written. **Throws:**: `java.io.IOException` - if an I/O error occurs. **See Also:**: `FilterOutputStream.out`

---


### writePString

```
public void writePString(java.lang.String s)
                  throws java.io.IOException
```

:   Writes a Pascal String.

    :   **Parameters:**: `s` - **Throws:**: `java.io.IOException`

---


### writeLong

```
public void writeLong(long v)
               throws java.io.IOException
```

:   **Throws:**: `java.io.IOException`

---


### writeUShort

```
public void writeUShort(int v)
                 throws java.io.IOException
```

:   **Throws:**: `java.io.IOException`

---


### incCount

```
protected void incCount(int value)
```

:   Increases the written counter by the specified value
    until it reaches Long.MAX\_VALUE.

---


### size

```
public final long size()
```

:   Returns the current value of the counter `written`,
    the number of bytes written to this data output stream so far.
    If the counter overflows, it will be wrapped to Integer.MAX\_VALUE.

    :   **Returns:**: the value of the `written` field. **See Also:**: `DataOutputStream.written`


---


|  |  |  |  |  |  |  |  |  |  |  |
| --- | --- | --- | --- | --- | --- | --- | --- | --- | --- | --- |
| |  |  |  |  |  |  |  |  | | --- | --- | --- | --- | --- | --- | --- | --- | | **Overview** | **Package** | **Class** | **Use** | **Tree** | **Deprecated** | **Index** | **Help** | | |  |
| PREV CLASS   **NEXT CLASS** | **FRAMES**    **NO FRAMES**     **All Classes** |
| SUMMARY: NESTED | FIELD | CONSTR | METHOD | DETAIL: FIELD | CONSTR | METHOD |


---
