# Supplementary material for: BSim: An Agent-Based Tool for Modeling Bacterial Populations in Systems and Synthetic Biology
Source: PLoS One. 2012 Aug 24;7(8):e42790. doi: 10.1371/journal.pone.0042790 (PMC3427305; doi:10.1371/journal.pone.0042790)
Supplement: Software S1 — Snapshot of the BSim software from 18th July 2012. For the latest version see: http://bsim-bccs.sf.net. The BSim software requires Java version 1.6 or higher. (ZIP) [file pone.0042790.s014.zip › BSimSoftware/docs/javadoc/bsim/export/quicktime/class-use/QuickTimeOutputStream.VideoFormat.html]

Uses of Class bsim.export.quicktime.QuickTimeOutputStream.VideoFormat


---


|  |  |  |  |  |  |  |  |  |  |  |
| --- | --- | --- | --- | --- | --- | --- | --- | --- | --- | --- |
| |  |  |  |  |  |  |  |  | | --- | --- | --- | --- | --- | --- | --- | --- | | **Overview** | **Package** | **Class** | **Use** | **Tree** | **Deprecated** | **Index** | **Help** | | |  |
| PREV   NEXT | **FRAMES**    **NO FRAMES**     **All Classes** |


---


## **Uses of Class bsim.export.quicktime.QuickTimeOutputStream.VideoFormat**

| Packages that use QuickTimeOutputStream.VideoFormat | |
| --- | --- |
| **bsim.export.quicktime** |  |

| Uses of QuickTimeOutputStream.VideoFormat in bsim.export.quicktime | |
| --- | --- |

| Methods in bsim.export.quicktime that return QuickTimeOutputStream.VideoFormat | |
| --- | --- |
| `static QuickTimeOutputStream.VideoFormat` | `QuickTimeOutputStream.VideoFormat.valueOf(java.lang.String name)`             Returns the enum constant of this type with the specified name. |
| `static QuickTimeOutputStream.VideoFormat[]` | `QuickTimeOutputStream.VideoFormat.values()`             Returns an array containing the constants of this enum type, in the order they are declared. |

| Constructors in bsim.export.quicktime with parameters of type QuickTimeOutputStream.VideoFormat | |
| --- | --- |
| `QuickTimeOutputStream(java.io.File file, QuickTimeOutputStream.VideoFormat format)`             Creates a new output stream with the specified image videoFormat and framerate. |

---


|  |  |  |  |  |  |  |  |  |  |  |
| --- | --- | --- | --- | --- | --- | --- | --- | --- | --- | --- |
| |  |  |  |  |  |  |  |  | | --- | --- | --- | --- | --- | --- | --- | --- | | **Overview** | **Package** | **Class** | **Use** | **Tree** | **Deprecated** | **Index** | **Help** | | |  |
| PREV   NEXT | **FRAMES**    **NO FRAMES**     **All Classes** |


---
