# Supplementary material for: BSim: An Agent-Based Tool for Modeling Bacterial Populations in Systems and Synthetic Biology
Source: PLoS One. 2012 Aug 24;7(8):e42790. doi: 10.1371/journal.pone.0042790 (PMC3427305; doi:10.1371/journal.pone.0042790)
Supplement: Software S1 — Snapshot of the BSim software from 18th July 2012. For the latest version see: http://bsim-bccs.sf.net. The BSim software requires Java version 1.6 or higher. (ZIP) [file pone.0042790.s014.zip › BSimSoftware/docs/javadoc/bsim/export/quicktime/FilterImageOutputStream.html]

FilterImageOutputStream


---


|  |  |  |  |  |  |  |  |  |  |  |
| --- | --- | --- | --- | --- | --- | --- | --- | --- | --- | --- |
| |  |  |  |  |  |  |  |  | | --- | --- | --- | --- | --- | --- | --- | --- | | **Overview** | **Package** | **Class** | **Use** | **Tree** | **Deprecated** | **Index** | **Help** | | |  |
| **PREV CLASS**   **NEXT CLASS** | **FRAMES**    **NO FRAMES**     **All Classes** |
| SUMMARY: NESTED | FIELD | CONSTR | METHOD | DETAIL: FIELD | CONSTR | METHOD |


---


## bsim.export.quicktime Class FilterImageOutputStream

```
java.lang.Object
  java.io.OutputStream
      java.io.FilterOutputStream
          bsim.export.quicktime.FilterImageOutputStream
```

**All Implemented Interfaces:**: java.io.Closeable, java.io.Flushable

---

``` public class FilterImageOutputStream extends java.io.FilterOutputStream ```

FilterImageOutputStream adapts a ImageOutputStream to the FilterOutputStream
interface.

**Version:**
:   1.0 15.06.2008 Created.

**Author:**
:   Werner Randelshofer

---

| **Field Summary** | |
| --- | --- |

| **Fields inherited from class java.io.FilterOutputStream** |
| --- |
| `out` |


| **Constructor Summary** | |
| --- | --- |
| `FilterImageOutputStream(javax.imageio.stream.ImageOutputStream iOut)` |


| **Method Summary** | |
| --- | --- |
| `void` | `close()`             Closes this output stream and releases any system resources associated with the stream. |
| `void` | `flush()`             Flushes this output stream and forces any buffered output bytes to be written out to the stream. |
| `void` | `write(byte[] b, int off, int len)`             Writes `len` bytes from the specified `byte` array starting at offset `off` to this output stream. |
| `void` | `write(int b)`             Writes the specified `byte` to this output stream. |

| **Methods inherited from class java.io.FilterOutputStream** |
| --- |
| `write` |

| **Methods inherited from class java.lang.Object** |
| --- |
| `clone, equals, finalize, getClass, hashCode, notify, notifyAll, toString, wait, wait, wait` |

| **Constructor Detail** |
| --- |

### FilterImageOutputStream

```
public FilterImageOutputStream(javax.imageio.stream.ImageOutputStream iOut)
```


| **Method Detail** |
| --- |

### write

```
public void write(int b)
           throws java.io.IOException
```

:   Writes the specified `byte` to this output stream.

    The `write` method of `FilterOutputStream`
    calls the `write` method of its underlying output stream,
    that is, it performs out.write(b).

    Implements the abstract write method of OutputStream.

    :   **Overrides:**: `write` in class `java.io.FilterOutputStream`
    :   **Parameters:**: `b` - the `byte`. **Throws:**: `java.io.IOException` - if an I/O error occurs.

---


### write

```
public void write(byte[] b,
                  int off,
                  int len)
           throws java.io.IOException
```

:   Writes `len` bytes from the specified
    `byte` array starting at offset `off` to
    this output stream.

    The `write` method of `FilterOutputStream`
    calls the `write` method of one argument on each
    `byte` to output.

    Note that this method does not call the `write` method
    of its underlying input stream with the same arguments. Subclasses
    of `FilterOutputStream` should provide a more efficient
    implementation of this method.

    :   **Overrides:**: `write` in class `java.io.FilterOutputStream`
    :   **Parameters:**: `b` - the data.: `off` - the start offset in the data.: `len` - the number of bytes to write. **Throws:**: `java.io.IOException` - if an I/O error occurs. **See Also:**: `FilterOutputStream.write(int)`

---


### flush

```
public void flush()
           throws java.io.IOException
```

:   Flushes this output stream and forces any buffered output bytes
    to be written out to the stream.

    The `flush` method of `FilterOutputStream`
    calls the `flush` method of its underlying output stream.

    :   **Specified by:**: `flush` in interface `java.io.Flushable` **Overrides:**: `flush` in class `java.io.FilterOutputStream`
    :   **Throws:**: `java.io.IOException` - if an I/O error occurs. **See Also:**: `FilterOutputStream.out`

---


### close

```
public void close()
           throws java.io.IOException
```

:   Closes this output stream and releases any system resources
    associated with the stream.

    The `close` method of `FilterOutputStream`
    calls its `flush` method, and then calls the
    `close` method of its underlying output stream.

    :   **Specified by:**: `close` in interface `java.io.Closeable` **Overrides:**: `close` in class `java.io.FilterOutputStream`
    :   **Throws:**: `java.io.IOException` - if an I/O error occurs. **See Also:**: `FilterOutputStream.flush()`, `FilterOutputStream.out`


---


|  |  |  |  |  |  |  |  |  |  |  |
| --- | --- | --- | --- | --- | --- | --- | --- | --- | --- | --- |
| |  |  |  |  |  |  |  |  | | --- | --- | --- | --- | --- | --- | --- | --- | | **Overview** | **Package** | **Class** | **Use** | **Tree** | **Deprecated** | **Index** | **Help** | | |  |
| **PREV CLASS**   **NEXT CLASS** | **FRAMES**    **NO FRAMES**     **All Classes** |
| SUMMARY: NESTED | FIELD | CONSTR | METHOD | DETAIL: FIELD | CONSTR | METHOD |


---
