# Supplementary material for: BSim: An Agent-Based Tool for Modeling Bacterial Populations in Systems and Synthetic Biology
Source: PLoS One. 2012 Aug 24;7(8):e42790. doi: 10.1371/journal.pone.0042790 (PMC3427305; doi:10.1371/journal.pone.0042790)
Supplement: Software S1 — Snapshot of the BSim software from 18th July 2012. For the latest version see: http://bsim-bccs.sf.net. The BSim software requires Java version 1.6 or higher. (ZIP) [file pone.0042790.s014.zip › BSimSoftware/docs/javadoc/bsim/export/quicktime/package-frame.html]

bsim.export.quicktime


bsim.export.quicktime

|  |
| --- |
| Classes    AtomDataOutputStream   FilterImageOutputStream   QuickTimeOutputStream |

|  |
| --- |
| Enums    QuickTimeOutputStream.VideoFormat |
