# Supplementary material for: BSim: An Agent-Based Tool for Modeling Bacterial Populations in Systems and Synthetic Biology
Source: PLoS One. 2012 Aug 24;7(8):e42790. doi: 10.1371/journal.pone.0042790 (PMC3427305; doi:10.1371/journal.pone.0042790)
Supplement: Software S1 — Snapshot of the BSim software from 18th July 2012. For the latest version see: http://bsim-bccs.sf.net. The BSim software requires Java version 1.6 or higher. (ZIP) [file pone.0042790.s014.zip › BSimSoftware/docs/javadoc/bsim/export/quicktime/QuickTimeOutputStream.html]

QuickTimeOutputStream


---


|  |  |  |  |  |  |  |  |  |  |  |
| --- | --- | --- | --- | --- | --- | --- | --- | --- | --- | --- |
| |  |  |  |  |  |  |  |  | | --- | --- | --- | --- | --- | --- | --- | --- | | **Overview** | **Package** | **Class** | **Use** | **Tree** | **Deprecated** | **Index** | **Help** | | |  |
| **PREV CLASS**   **NEXT CLASS** | **FRAMES**    **NO FRAMES**     **All Classes** |
| SUMMARY: NESTED | FIELD | CONSTR | METHOD | DETAIL: FIELD | CONSTR | METHOD |


---


## bsim.export.quicktime Class QuickTimeOutputStream

```
java.lang.Object
  bsim.export.quicktime.QuickTimeOutputStream
```

---

``` public class QuickTimeOutputStream extends java.lang.Object ```

This class supports writing of images as frames into the video track of
a QuickTime movie file.

All frames are encoded either using the JPG or the PNG video format.
Each frame can have an individual encoding quality and duration.

For detailed information about the QuickTime file format see:
http://developer.apple.com/documentation/QuickTime/QTFF/

**Version:**
:   1.0.1 2008-06-18 WideLeafAtom wrote incorrect header for
    atoms larger than 4 GB. The default value of time scale is now 600.
    Renamed method writeFrame to writeFrame. Added writeFrame methods
    which take a file or an input stream as argument.
      
    1.0 Jun 15, 2008 Created.

**Author:**
:   Werner Randelshofer

---

| **Nested Class Summary** | |
| --- | --- |
| `static class` | `QuickTimeOutputStream.VideoFormat`             Supported video encodings. |


| **Constructor Summary** | |
| --- | --- |
| `QuickTimeOutputStream(java.io.File file, QuickTimeOutputStream.VideoFormat format)`             Creates a new output stream with the specified image videoFormat and framerate. |


| **Method Summary** | |
| --- | --- |
| `void` | `close()`             Closes the movie file as well as the stream being filtered. |
| `void` | `finish()`             Finishes writing the contents of the QuickTime output stream without closing the underlying stream. |
| `int` | `getTimeScale()`             Returns the time scale of this media. |
| `float` | `getVideoCompressionQuality()`             Returns the video compression quality. |
| `void` | `setTimeScale(int newValue)`             Sets the time scale for this media, that is, the number of time units that pass per second in its time coordinate system. |
| `void` | `setVideoCompressionQuality(float newValue)`             Sets the compression quality of the video track. |
| `void` | `setVideoDimension(int width, int height)`             Sets the dimension of the video track. |
| `void` | `writeFrame(java.awt.image.BufferedImage image, int duration)`             Writes a frame to the video track. |
| `void` | `writeFrame(java.io.File file, int duration)`             Writes a frame from a file to the video track. |
| `void` | `writeFrame(java.io.InputStream in, int duration)`             Writes a frame to the video track. |

| **Methods inherited from class java.lang.Object** |
| --- |
| `clone, equals, finalize, getClass, hashCode, notify, notifyAll, toString, wait, wait, wait` |

| **Constructor Detail** |
| --- |

### QuickTimeOutputStream

```
public QuickTimeOutputStream(java.io.File file,
                             QuickTimeOutputStream.VideoFormat format)
                      throws java.io.IOException
```

:   Creates a new output stream with the specified image videoFormat and
    framerate.

    **Parameters:**: `file` - the output file: `videoFormat` - the video videoFormat "JPG" or "PNG".: `framerate` - the number of videoFrames per section **Throws:**: `java.lang.IllegalArgumentException` - if videoFormat is null or if framerate is <= 0: `java.io.IOException`


| **Method Detail** |
| --- |

### setTimeScale

```
public void setTimeScale(int newValue)
```

:   Sets the time scale for this media, that is, the number of time units
    that pass per second in its time coordinate system.

    The default value is 600.

    :   **Parameters:**: `newValue` -

---


### getTimeScale

```
public int getTimeScale()
```

:   Returns the time scale of this media.

    :   **Returns:**: time scale

---


### setVideoCompressionQuality

```
public void setVideoCompressionQuality(float newValue)
```

:   Sets the compression quality of the video track.
    A value of 0 stands for "high compression is important" a value of
    1 for "high image quality is important".

    Changing this value affects frames which are subsequently written
    to the QuickTimeOutputStream. Frames which have already been written
    are not changed.

    This value has no effect on videos encoded with the PNG format.

    The default value is 0.9.

    :   **Parameters:**: `newValue` -

---


### getVideoCompressionQuality

```
public float getVideoCompressionQuality()
```

:   Returns the video compression quality.

    :   **Returns:**: video compression quality

---


### setVideoDimension

```
public void setVideoDimension(int width,
                              int height)
```

:   Sets the dimension of the video track.

    You need to explicitly set the dimension, if you add all frames from
    files or input streams.

    If you add frames from buffered images, then QuickTimeOutputStream
    can determine the video dimension from the image width and height.

    :   **Parameters:**: `width` -: `height` -

---


### writeFrame

```
public void writeFrame(java.awt.image.BufferedImage image,
                       int duration)
                throws java.io.IOException
```

:   Writes a frame to the video track.

    If the dimension of the video track has not been specified yet, it
    is derived from the first buffered image added to the QuickTimeOutputStream.

    :   **Parameters:**: `image` - The frame image.: `duration` - The duration of the frame in time scale units. **Throws:**: `java.lang.IllegalArgumentException` - if the duration is less than 1, or if the dimension of the frame does not match the dimension of the video track.: `java.io.IOException` - if writing the image failed.

---


### writeFrame

```
public void writeFrame(java.io.File file,
                       int duration)
                throws java.io.IOException
```

:   Writes a frame from a file to the video track.

    This method does not inspect the contents of the file.
    Its your responsibility to only add JPG files if you have chosen
    the JPEG video format, and only PNG files if you have chosen the PNG
    video format.

    If you add all frames from files or from input streams, then you
    have to explicitly set the dimension of the video track before you
    call finish() or close().

    :   **Parameters:**: `file` - The file which holds the image data.: `duration` - The duration of the frame in time scale units. **Throws:**: `java.lang.IllegalStateException` - if the duration is less than 1.: `java.io.IOException` - if writing the image failed.

---


### writeFrame

```
public void writeFrame(java.io.InputStream in,
                       int duration)
                throws java.io.IOException
```

:   Writes a frame to the video track.

    This method does not inspect the contents of the file.
    Its your responsibility to only add JPG files if you have chosen
    the JPEG video format, and only PNG files if you have chosen the PNG
    video format.

    If you add all frames from files or from input streams, then you
    have to explicitly set the dimension of the video track before you
    call finish() or close().

    :   **Parameters:**: `in` - The input stream which holds the image data.: `duration` - The duration of the frame in time scale units. **Throws:**: `java.lang.IllegalArgumentException` - if the duration is less than 1.: `java.io.IOException` - if writing the image failed.

---


### close

```
public void close()
           throws java.io.IOException
```

:   Closes the movie file as well as the stream being filtered.

    :   **Throws:**: `java.io.IOException` - if an I/O error has occurred

---


### finish

```
public void finish()
            throws java.io.IOException
```

:   Finishes writing the contents of the QuickTime output stream without closing
    the underlying stream. Use this method when applying multiple filters
    in succession to the same output stream.

    :   **Throws:**: `java.lang.IllegalStateException` - if the dimension of the video track has not been specified or determined yet.: `java.io.IOException` - if an I/O exception has occurred


---


|  |  |  |  |  |  |  |  |  |  |  |
| --- | --- | --- | --- | --- | --- | --- | --- | --- | --- | --- |
| |  |  |  |  |  |  |  |  | | --- | --- | --- | --- | --- | --- | --- | --- | | **Overview** | **Package** | **Class** | **Use** | **Tree** | **Deprecated** | **Index** | **Help** | | |  |
| **PREV CLASS**   **NEXT CLASS** | **FRAMES**    **NO FRAMES**     **All Classes** |
| SUMMARY: NESTED | FIELD | CONSTR | METHOD | DETAIL: FIELD | CONSTR | METHOD |


---
