# Supplementary material for: BSim: An Agent-Based Tool for Modeling Bacterial Populations in Systems and Synthetic Biology
Source: PLoS One. 2012 Aug 24;7(8):e42790. doi: 10.1371/journal.pone.0042790 (PMC3427305; doi:10.1371/journal.pone.0042790)
Supplement: Software S1 — Snapshot of the BSim software from 18th July 2012. For the latest version see: http://bsim-bccs.sf.net. The BSim software requires Java version 1.6 or higher. (ZIP) [file pone.0042790.s014.zip › BSimSoftware/docs/javadoc/bsim/geometry/BSimCollision.html]

BSimCollision


---


|  |  |  |  |  |  |  |  |  |  |  |
| --- | --- | --- | --- | --- | --- | --- | --- | --- | --- | --- |
| |  |  |  |  |  |  |  |  | | --- | --- | --- | --- | --- | --- | --- | --- | | **Overview** | **Package** | **Class** | **Use** | **Tree** | **Deprecated** | **Index** | **Help** | | |  |
| PREV CLASS   **NEXT CLASS** | **FRAMES**    **NO FRAMES**     **All Classes** |
| SUMMARY: NESTED | FIELD | CONSTR | METHOD | DETAIL: FIELD | CONSTR | METHOD |


---


## bsim.geometry Class BSimCollision

```
java.lang.Object
  bsim.geometry.BSimCollision
```

---

``` public class BSimCollision extends java.lang.Object ```

Collision related methods.

---

| **Field Summary** | |
| --- | --- |
| `protected  javax.vecmath.Vector3d` | `pos` |
| `static boolean` | `recursiveCollisions` |
| `protected  double` | `t` |


| **Constructor Summary** | |
| --- | --- |
| `BSimCollision()`             Constructor for an empty collision. |


| **Method Summary** | |
| --- | --- |
| `static boolean` | `collideAndCross(javax.vecmath.Vector3d p1, javax.vecmath.Vector3d p2, BSimMesh theMesh)`             Check if mesh is crossed. |
| `static void` | `collideAndReflect(javax.vecmath.Vector3d p1, javax.vecmath.Vector3d p2, BSimMesh theMesh)`             Check to see if intersection with mesh and reflect. |
| `static void` | `collideAndRepel(BSimParticle p, BSimMesh theMesh)`             Check for collision between particle and mesh, and add repulsion force. |
| `javax.vecmath.Vector3d` | `getLocation()`             Return location of collision. |
| `double` | `getTVal()`             Return the t value. |
| `void` | `set(BSimCollision col)`             Set the collision values. |
| `void` | `set(BSimTriangle tri, double tVal, double u, double v, double w)` |
| `void` | `set(double tVal, javax.vecmath.Vector3d qp, javax.vecmath.Vector3d startPos)` |
| `static void` | `setRecursiveCollisions(boolean recursiveCollisionsActive)`             Set whether recursive collisions are enabled. |

| **Methods inherited from class java.lang.Object** |
| --- |
| `clone, equals, finalize, getClass, hashCode, notify, notifyAll, toString, wait, wait, wait` |

| **Field Detail** |
| --- |

### t

```
protected double t
```

---


### pos

```
protected javax.vecmath.Vector3d pos
```

---


### recursiveCollisions

```
public static boolean recursiveCollisions
```


| **Constructor Detail** |
| --- |

### BSimCollision

```
public BSimCollision()
```

:   Constructor for an empty collision.


| **Method Detail** |
| --- |

### set

```
public void set(BSimTriangle tri,
                double tVal,
                double u,
                double v,
                double w)
```

---


### set

```
public void set(double tVal,
                javax.vecmath.Vector3d qp,
                javax.vecmath.Vector3d startPos)
```

---


### set

```
public void set(BSimCollision col)
```

:   Set the collision values.

---


### getTVal

```
public double getTVal()
```

:   Return the t value.

---


### getLocation

```
public javax.vecmath.Vector3d getLocation()
```

:   Return location of collision.

---


### setRecursiveCollisions

```
public static void setRecursiveCollisions(boolean recursiveCollisionsActive)
```

:   Set whether recursive collisions are enabled.

---


### collideAndRepel

```
public static void collideAndRepel(BSimParticle p,
                                   BSimMesh theMesh)
```

:   Check for collision between particle and mesh, and add repulsion force.

    :   **Parameters:**: `p` - Particle to check.: `theMesh` - Mesh to check for intersection with.

---


### collideAndReflect

```
public static void collideAndReflect(javax.vecmath.Vector3d p1,
                                     javax.vecmath.Vector3d p2,
                                     BSimMesh theMesh)
```

:   Check to see if intersection with mesh and reflect. End point (p2) is updated to the correct
    reflected position. Note that this is colliding a ray with a triangle (NOT in fact
    a swept sphere vs triangle).

    :   **Parameters:**: `p1` - Start point: `p2` - End point: `theMesh` - Mesh to check for intersection with.

---


### collideAndCross

```
public static boolean collideAndCross(javax.vecmath.Vector3d p1,
                                      javax.vecmath.Vector3d p2,
                                      BSimMesh theMesh)
```

:   Check if mesh is crossed.

    :   **Parameters:**: `p1` - Start point.: `p2` - End point: `theMesh` - Mesh to check for intersection with. **Returns:**: Boolean whether crossing occurs.


---


|  |  |  |  |  |  |  |  |  |  |  |
| --- | --- | --- | --- | --- | --- | --- | --- | --- | --- | --- |
| |  |  |  |  |  |  |  |  | | --- | --- | --- | --- | --- | --- | --- | --- | | **Overview** | **Package** | **Class** | **Use** | **Tree** | **Deprecated** | **Index** | **Help** | | |  |
| PREV CLASS   **NEXT CLASS** | **FRAMES**    **NO FRAMES**     **All Classes** |
| SUMMARY: NESTED | FIELD | CONSTR | METHOD | DETAIL: FIELD | CONSTR | METHOD |


---
