# Supplementary material for: BSim: An Agent-Based Tool for Modeling Bacterial Populations in Systems and Synthetic Biology
Source: PLoS One. 2012 Aug 24;7(8):e42790. doi: 10.1371/journal.pone.0042790 (PMC3427305; doi:10.1371/journal.pone.0042790)
Supplement: Software S1 — Snapshot of the BSim software from 18th July 2012. For the latest version see: http://bsim-bccs.sf.net. The BSim software requires Java version 1.6 or higher. (ZIP) [file pone.0042790.s014.zip › BSimSoftware/docs/javadoc/bsim/geometry/BSimMesh.html]

BSimMesh


---


|  |  |  |  |  |  |  |  |  |  |  |
| --- | --- | --- | --- | --- | --- | --- | --- | --- | --- | --- |
| |  |  |  |  |  |  |  |  | | --- | --- | --- | --- | --- | --- | --- | --- | | **Overview** | **Package** | **Class** | **Use** | **Tree** | **Deprecated** | **Index** | **Help** | | |  |
| **PREV CLASS**   **NEXT CLASS** | **FRAMES**    **NO FRAMES**     **All Classes** |
| SUMMARY: NESTED | FIELD | CONSTR | METHOD | DETAIL: FIELD | CONSTR | METHOD |


---


## bsim.geometry Class BSimMesh

```
java.lang.Object
  bsim.geometry.BSimMesh
```

**Direct Known Subclasses:**: BSimOBJMesh, BSimSphereMesh, KdNode.TestMesh

---

``` public abstract class BSimMesh extends java.lang.Object ```

Abstract 3-D mesh surface class. Represented as indexed list of vertices,
or points in 3-D, of which the mesh faces are composed.

---

| **Field Summary** | |
| --- | --- |
| `protected  java.util.ArrayList<BSimTriangle>` | `faces`             List of faces, each face stores the indices of the vertices which compose that face. |
| `protected  java.util.ArrayList<BSimVertex>` | `vertices`             The actual locations (3D coordinates) of all mesh vertices |


| **Constructor Summary** | |
| --- | --- |
| `BSimMesh()`             Default constructor, initialises the vertex and triangle lists. |


| **Method Summary** | |
| --- | --- |
| `void` | `addTriangle(BSimTriangle t)`             Add an existing triangle to the face list |
| `void` | `addTriangle(int v1, int v2, int v3)`             Add a triangular face to the face list. |
| `int` | `addVertex(double newX, double newY, double newZ)`             Add a vertex to the vertex list (based on x,y,z coordinates). |
| `int` | `addVertex(javax.vecmath.Vector3d p)`             Add a vertex to the vertex list (using a Vector3d). |
| `javax.vecmath.Vector3d` | `averagedCentreOfMesh()`             Compute the (unweighted) average centre coordinate of all mesh vertices. |
| `protected  void` | `calcVertexFaces()`             Compute which faces index each vertex, and store this as a list parameter in each vertex object. |
| `protected  void` | `cleanUp(boolean stats)`             Trim down the arrayLists, and compute vertex-face connectivity. |
| `void` | `computeNormal(BSimTriangle t)`             Compute the normal vector of a face. |
| `void` | `computeNormals()`             Compute all normals of the mesh |
| `protected abstract  void` | `createMesh()`             Abstract method in which the vertices and faces of the mesh should be defined. |
| `void` | `flipNormals()`             Flip normals of all faces |
| `void` | `flipNormals(int[] faceList)`             Flip normals of selected faces |
| `BSimTriangle` | `getFace(int i)` |
| `java.util.ArrayList<BSimTriangle>` | `getFaces()` |
| `javax.vecmath.Vector3d` | `getTCentre(BSimTriangle t)`             Compute the coordinates of the centre of a triangle |
| `javax.vecmath.Vector3d` | `getVertCoords(int vertIndex)` |
| `javax.vecmath.Vector3d` | `getVertCoordsOfTri(BSimTriangle t, int i)`             Get the vertex coordinates of a given triangle |
| `BSimVertex` | `getVertex(int i)` |
| `java.util.ArrayList<BSimVertex>` | `getVertices()` |
| `void` | `printStats()`             Print mesh statistics (face vertices, vertex coords, vertex faces, normals...) |
| `void` | `scale(double scaleFactor)`             Scale mesh on origin (0, 0, 0). |
| `void` | `scale(double scaleFactor, javax.vecmath.Vector3d scaleOn)`             Scale mesh on arbitrary point |
| `void` | `translate(javax.vecmath.Vector3d translation)`             Translate the mesh in an arbitrary direction. |
| `void` | `translateAbsolute(javax.vecmath.Vector3d newLocation)`             Translate the whole mesh so that it is centred on a new point in 3d space. |

| **Methods inherited from class java.lang.Object** |
| --- |
| `clone, equals, finalize, getClass, hashCode, notify, notifyAll, toString, wait, wait, wait` |

| **Field Detail** |
| --- |

### vertices

```
protected java.util.ArrayList<BSimVertex> vertices
```

:   The actual locations (3D coordinates) of all mesh vertices

---


### faces

```
protected java.util.ArrayList<BSimTriangle> faces
```

:   List of faces, each face stores the indices of the vertices which compose that face.


| **Constructor Detail** |
| --- |

### BSimMesh

```
public BSimMesh()
```

:   Default constructor, initialises the vertex and triangle lists.


| **Method Detail** |
| --- |

### createMesh

```
protected abstract void createMesh()
```

:   Abstract method in which the vertices and faces of the mesh should be defined.

---


### addVertex

```
public int addVertex(double newX,
                     double newY,
                     double newZ)
```

:   Add a vertex to the vertex list (based on x,y,z coordinates).

---


### addVertex

```
public int addVertex(javax.vecmath.Vector3d p)
```

:   Add a vertex to the vertex list (using a Vector3d).

---


### addTriangle

```
public void addTriangle(int v1,
                        int v2,
                        int v3)
```

:   Add a triangular face to the face list.
    Parameters are the indices in the vertex list of the three corner points of the triangle.

    :   **Parameters:**: `v1` - Index in list 'vertices' of face vertex 1.: `v2` - Index in list 'vertices' of face vertex 2.: `v3` - Index in list 'vertices' of face vertex 3.

---


### addTriangle

```
public void addTriangle(BSimTriangle t)
```

:   Add an existing triangle to the face list

    :   **Parameters:**: `t` - The BSimTriangle to be added

---


### calcVertexFaces

```
protected void calcVertexFaces()
```

:   Compute which faces index each vertex, and store this as a list parameter in each vertex object.
    Face connectivity from a vertex should be a useful parameter when doing space subdivision etc.

---


### cleanUp

```
protected void cleanUp(boolean stats)
```

:   Trim down the arrayLists, and compute vertex-face connectivity.
    Minimises storage and hopefully increases efficiency (unless we will be changing these lists later).

    :   **Parameters:**: `stats` - (if true, print mesh statistics after clean-up is done.)

---


### computeNormal

```
public void computeNormal(BSimTriangle t)
```

:   Compute the normal vector of a face.

---


### computeNormals

```
public void computeNormals()
```

:   Compute all normals of the mesh

---


### flipNormals

```
public void flipNormals()
```

:   Flip normals of all faces

---


### flipNormals

```
public void flipNormals(int[] faceList)
```

:   Flip normals of selected faces

    :   **Parameters:**: `normalsList` - Array of integer indices corresponding to the faces we wish to flip.

---


### scale

```
public void scale(double scaleFactor,
                  javax.vecmath.Vector3d scaleOn)
```

:   Scale mesh on arbitrary point

    :   **Parameters:**: `scaleFactor` - The factor by which the mesh will be scaled (1.0 = no scaling, 2.0 = double size, 0.5 = half size, etc.): `scaleOn` - The point from which the mesh will be scaled.

---


### scale

```
public void scale(double scaleFactor)
```

:   Scale mesh on origin (0, 0, 0).

    :   **Parameters:**: `scaleFactor` - The factor by which the mesh will be scaled.

---


### averagedCentreOfMesh

```
public javax.vecmath.Vector3d averagedCentreOfMesh()
```

:   Compute the (unweighted) average centre coordinate of all mesh vertices.

    :   **Returns:**

---


### translateAbsolute

```
public void translateAbsolute(javax.vecmath.Vector3d newLocation)
```

:   Translate the whole mesh so that it is centred on a new point in 3d space.

    :   **Parameters:**: `newLocation` - The location on which the mesh will be centred.

---


### translate

```
public void translate(javax.vecmath.Vector3d translation)
```

:   Translate the mesh in an arbitrary direction.

    :   **Parameters:**: `translation` - The vector by which all vertices are translated

---


### getVertices

```
public java.util.ArrayList<BSimVertex> getVertices()
```

---


### getVertex

```
public BSimVertex getVertex(int i)
```

---


### getFaces

```
public java.util.ArrayList<BSimTriangle> getFaces()
```

---


### getFace

```
public BSimTriangle getFace(int i)
```

---


### getVertCoords

```
public javax.vecmath.Vector3d getVertCoords(int vertIndex)
```

---


### getVertCoordsOfTri

```
public javax.vecmath.Vector3d getVertCoordsOfTri(BSimTriangle t,
                                                 int i)
```

:   Get the vertex coordinates of a given triangle

    :   **Parameters:**: `t` -: `i` - Index of the vertex for which to get coordinates (0, 1, 2) **Returns:**

---


### getTCentre

```
public javax.vecmath.Vector3d getTCentre(BSimTriangle t)
```

:   Compute the coordinates of the centre of a triangle

    :   **Parameters:**: `t` - **Returns:**

---


### printStats

```
public void printStats()
```

:   Print mesh statistics (face vertices, vertex coords, vertex faces, normals...)


---


|  |  |  |  |  |  |  |  |  |  |  |
| --- | --- | --- | --- | --- | --- | --- | --- | --- | --- | --- |
| |  |  |  |  |  |  |  |  | | --- | --- | --- | --- | --- | --- | --- | --- | | **Overview** | **Package** | **Class** | **Use** | **Tree** | **Deprecated** | **Index** | **Help** | | |  |
| **PREV CLASS**   **NEXT CLASS** | **FRAMES**    **NO FRAMES**     **All Classes** |
| SUMMARY: NESTED | FIELD | CONSTR | METHOD | DETAIL: FIELD | CONSTR | METHOD |


---
