# Supplementary material for: BSim: An Agent-Based Tool for Modeling Bacterial Populations in Systems and Synthetic Biology
Source: PLoS One. 2012 Aug 24;7(8):e42790. doi: 10.1371/journal.pone.0042790 (PMC3427305; doi:10.1371/journal.pone.0042790)
Supplement: Software S1 — Snapshot of the BSim software from 18th July 2012. For the latest version see: http://bsim-bccs.sf.net. The BSim software requires Java version 1.6 or higher. (ZIP) [file pone.0042790.s014.zip › BSimSoftware/docs/javadoc/bsim/geometry/BSimMeshUtils.html]

BSimMeshUtils


---


|  |  |  |  |  |  |  |  |  |  |  |
| --- | --- | --- | --- | --- | --- | --- | --- | --- | --- | --- |
| |  |  |  |  |  |  |  |  | | --- | --- | --- | --- | --- | --- | --- | --- | | **Overview** | **Package** | **Class** | **Use** | **Tree** | **Deprecated** | **Index** | **Help** | | |  |
| **PREV CLASS**   **NEXT CLASS** | **FRAMES**    **NO FRAMES**     **All Classes** |
| SUMMARY: NESTED | FIELD | CONSTR | METHOD | DETAIL: FIELD | CONSTR | METHOD |


---


## bsim.geometry Class BSimMeshUtils

```
java.lang.Object
  bsim.geometry.BSimMeshUtils
```

---

``` public class BSimMeshUtils extends java.lang.Object ```

Utility functions for meshes.

---

| **Constructor Summary** | |
| --- | --- |
| `BSimMeshUtils()` |


| **Method Summary** | |
| --- | --- |
| `static javax.vecmath.Vector3d` | `closestPtPointTriangle(javax.vecmath.Vector3d p, javax.vecmath.Vector3d a, javax.vecmath.Vector3d b, javax.vecmath.Vector3d c)`             Closest point on triangle to a given point in 3d space. |
| `static boolean` | `intersectPlaneAAB(javax.vecmath.Vector3d normal, double d, javax.vecmath.Vector3d boxCentre, javax.vecmath.Vector3d boxDim)`             Test for intersection between a plane and an axis-aligned box. |
| `static boolean` | `intersectSpherePlane(BSimParticle s, javax.vecmath.Vector3d planeNormal, javax.vecmath.Vector3d a)`             Computes the intersection of a sphere with a plane |
| `static boolean` | `intersectSphereTriangle(BSimParticle s, javax.vecmath.Vector3d a, javax.vecmath.Vector3d b, javax.vecmath.Vector3d c, javax.vecmath.Vector3d p)`             Intersection of a sphere with a triangle |
| `static boolean` | `intersectTriangleAAB(BSimTriangle t, javax.vecmath.Vector3d boxCentre, javax.vecmath.Vector3d boxDim)`             Test for intersection of a triangle against an axis aligned box |
| `static boolean` | `intersectTriOctreeNode(BSimTriangle t, BSimOctreeField n)`             Test for intersection of a triangle against an octree node |
| `static BSimCollision` | `intersectVectorPlane(javax.vecmath.Vector3d p1, javax.vecmath.Vector3d direction, javax.vecmath.Vector3d normalPlane, double dPlane)`             Compute the intersection of a vector p1 + t\*dir (line segment) and a plane |
| `static boolean` | `intersectVectorTriangle(javax.vecmath.Vector3d startPos, javax.vecmath.Vector3d endPos, BSimTriangle tri, BSimCollision coll)`             Computes intersection of a vector in 3d space (e.g. |

| **Methods inherited from class java.lang.Object** |
| --- |
| `clone, equals, finalize, getClass, hashCode, notify, notifyAll, toString, wait, wait, wait` |

| **Constructor Detail** |
| --- |

### BSimMeshUtils

```
public BSimMeshUtils()
```


| **Method Detail** |
| --- |

### intersectSphereTriangle

```
public static boolean intersectSphereTriangle(BSimParticle s,
                                              javax.vecmath.Vector3d a,
                                              javax.vecmath.Vector3d b,
                                              javax.vecmath.Vector3d c,
                                              javax.vecmath.Vector3d p)
```

:   Intersection of a sphere with a triangle

    :   **Parameters:**: `s` -: `a` -: `b` -: `c` -: `p` - the point of intersection **Returns:**: true = the sphere does intersect the triangle

---


### closestPtPointTriangle

```
public static javax.vecmath.Vector3d closestPtPointTriangle(javax.vecmath.Vector3d p,
                                                            javax.vecmath.Vector3d a,
                                                            javax.vecmath.Vector3d b,
                                                            javax.vecmath.Vector3d c)
```

:   Closest point on triangle to a given point in 3d space.
    RTCD chapter 5.

    :   **Parameters:**: `p` - Point to test: `a` - Triangle vertex a (v0): `b` - Triangle vertex b (v1): `c` - Triangle vertex c (v2) **Returns:**

---


### intersectSpherePlane

```
public static boolean intersectSpherePlane(BSimParticle s,
                                           javax.vecmath.Vector3d planeNormal,
                                           javax.vecmath.Vector3d a)
```

:   Computes the intersection of a sphere with a plane

    :   **Parameters:**: `s` - the particle to test: `planeNormal` - normal of the plane to be tested: `a` - a point in the plane **Returns:**

---


### intersectVectorTriangle

```
public static boolean intersectVectorTriangle(javax.vecmath.Vector3d startPos,
                                              javax.vecmath.Vector3d endPos,
                                              BSimTriangle tri,
                                              BSimCollision coll)
```

:   Computes intersection of a vector in 3d space (e.g. the direction a particle is moving in)
    and a triangle (e.g. a boundary, substrate, etc)

    :   **Parameters:**: `v` - the vector to be tested: `t` - the triangle to be tested

---


### intersectVectorPlane

```
public static BSimCollision intersectVectorPlane(javax.vecmath.Vector3d p1,
                                                 javax.vecmath.Vector3d direction,
                                                 javax.vecmath.Vector3d normalPlane,
                                                 double dPlane)
```

:   Compute the intersection of a vector p1 + t\*dir (line segment) and a plane

    :   **Parameters:**: `p1` - Line segment origin: `direction` - Line segment direction (UNNORMALISED = p2 - p1): `normalPlane` - Plane normal: `dPlane` - Plane d value = DOT(normal, point\_on\_plane) **Returns:**

---


### intersectTriOctreeNode

```
public static boolean intersectTriOctreeNode(BSimTriangle t,
                                             BSimOctreeField n)
```

:   Test for intersection of a triangle against an octree node

    :   **Parameters:**: `t` - BSimTriangle to test: `n` - Octree node to test against **Returns:**: true if an intersection occurs

---


### intersectTriangleAAB

```
public static boolean intersectTriangleAAB(BSimTriangle t,
                                           javax.vecmath.Vector3d boxCentre,
                                           javax.vecmath.Vector3d boxDim)
```

:   Test for intersection of a triangle against an axis aligned box

    :   **Parameters:**: `t` - BSimTriangle to test: `boxCentre` - Centre coordinate of the box: `boxDim` - Box length in x,y,z directions **Returns:**: true if an intersection occurs

---


### intersectPlaneAAB

```
public static boolean intersectPlaneAAB(javax.vecmath.Vector3d normal,
                                        double d,
                                        javax.vecmath.Vector3d boxCentre,
                                        javax.vecmath.Vector3d boxDim)
```

:   Test for intersection between a plane and an axis-aligned box.

    :   **Parameters:**: `normal` - Plane normal: `d` - Plane d value (from plane equation, d = normal.dot(v0)) where v0 is some point on the plane: `boxCentre` - Box centre coordinates: `boxDim` - Box side lengths in x,y,z directions **Returns:**: true if an intersection occurs


---


|  |  |  |  |  |  |  |  |  |  |  |
| --- | --- | --- | --- | --- | --- | --- | --- | --- | --- | --- |
| |  |  |  |  |  |  |  |  | | --- | --- | --- | --- | --- | --- | --- | --- | | **Overview** | **Package** | **Class** | **Use** | **Tree** | **Deprecated** | **Index** | **Help** | | |  |
| **PREV CLASS**   **NEXT CLASS** | **FRAMES**    **NO FRAMES**     **All Classes** |
| SUMMARY: NESTED | FIELD | CONSTR | METHOD | DETAIL: FIELD | CONSTR | METHOD |


---
