# Supplementary material for: BSim: An Agent-Based Tool for Modeling Bacterial Populations in Systems and Synthetic Biology
Source: PLoS One. 2012 Aug 24;7(8):e42790. doi: 10.1371/journal.pone.0042790 (PMC3427305; doi:10.1371/journal.pone.0042790)
Supplement: Software S1 — Snapshot of the BSim software from 18th July 2012. For the latest version see: http://bsim-bccs.sf.net. The BSim software requires Java version 1.6 or higher. (ZIP) [file pone.0042790.s014.zip › BSimSoftware/docs/javadoc/bsim/geometry/BSimSphereMesh.html]

BSimSphereMesh


---


|  |  |  |  |  |  |  |  |  |  |  |
| --- | --- | --- | --- | --- | --- | --- | --- | --- | --- | --- |
| |  |  |  |  |  |  |  |  | | --- | --- | --- | --- | --- | --- | --- | --- | | **Overview** | **Package** | **Class** | **Use** | **Tree** | **Deprecated** | **Index** | **Help** | | |  |
| **PREV CLASS**   **NEXT CLASS** | **FRAMES**    **NO FRAMES**     **All Classes** |
| SUMMARY: NESTED | FIELD | CONSTR | METHOD | DETAIL: FIELD | CONSTR | METHOD |


---


## bsim.geometry Class BSimSphereMesh

```
java.lang.Object
  bsim.geometry.BSimMesh
      bsim.geometry.BSimSphereMesh
```

---

``` public class BSimSphereMesh extends BSimMesh ```

Sphere mesh, uses face-vertex representation.

---

| **Field Summary** | |
| --- | --- |

| **Fields inherited from class bsim.geometry.BSimMesh** |
| --- |
| `faces, vertices` |


| **Constructor Summary** | |
| --- | --- |
| `BSimSphereMesh(javax.vecmath.Vector3d centre, double radius, int subdivisionRecursions)`             Main constructor. |


| **Method Summary** | |
| --- | --- |
| `protected  int` | `addScaledVertex(javax.vecmath.Vector3d p)`             Helper wrapper: ultimately the same method as BSimFVMesh.addVertex(), but scales the vertex position to be a unit distance from the origin. |
| `protected  void` | `createMesh()`             Defines the vertices and faces for a geodesic sphere. |
| `protected  int` | `getMiddle(int p1, int p2)`             Create the middle vertex between two vertices if it doesn't already exist. |

| **Methods inherited from class bsim.geometry.BSimMesh** |
| --- |
| `addTriangle, addTriangle, addVertex, addVertex, averagedCentreOfMesh, calcVertexFaces, cleanUp, computeNormal, computeNormals, flipNormals, flipNormals, getFace, getFaces, getTCentre, getVertCoords, getVertCoordsOfTri, getVertex, getVertices, printStats, scale, scale, translate, translateAbsolute` |

| **Methods inherited from class java.lang.Object** |
| --- |
| `clone, equals, finalize, getClass, hashCode, notify, notifyAll, toString, wait, wait, wait` |

| **Constructor Detail** |
| --- |

### BSimSphereMesh

```
public BSimSphereMesh(javax.vecmath.Vector3d centre,
                      double radius,
                      int subdivisionRecursions)
```

:   Main constructor. Generates the unit sphere on the origin and then transforms it to
    conform to the specified parameters.

    **Parameters:**: `centre` - The desired centre coordinates of the sphere in 3D space.: `radius` - The desired sphere radius.: `subdivisionRecursions` - Desired number of subdivision and smoothing iterations (note, no. of faces increases by a factor of 4 for each iteration).


| **Method Detail** |
| --- |

### createMesh

```
protected void createMesh()
```

:   Defines the vertices and faces for a geodesic sphere.
    Initially defines an icosahedron, which is then recursively subdivided and smoothed.

    :   **Specified by:**: `createMesh` in class `BSimMesh`

---


### addScaledVertex

```
protected int addScaledVertex(javax.vecmath.Vector3d p)
```

:   Helper wrapper: ultimately the same method as BSimFVMesh.addVertex(),
    but scales the vertex position to be a unit distance from the origin.

    :   **Parameters:**: `p` - The vertex coordinates in 3D space.

---


### getMiddle

```
protected int getMiddle(int p1,
                        int p2)
```

:   Create the middle vertex between two vertices if it doesn't already exist.
    Accesses BSimSphereMesh's hashed midpoint cache to ensure that vertices are
    not duplicated. Symmetry means that multiple faces will share midpoint
    vertices so this saves time and storage.

    :   **Parameters:**: `p1` - First vertex index.: `p2` - Second vertex index.


---


|  |  |  |  |  |  |  |  |  |  |  |
| --- | --- | --- | --- | --- | --- | --- | --- | --- | --- | --- |
| |  |  |  |  |  |  |  |  | | --- | --- | --- | --- | --- | --- | --- | --- | | **Overview** | **Package** | **Class** | **Use** | **Tree** | **Deprecated** | **Index** | **Help** | | |  |
| **PREV CLASS**   **NEXT CLASS** | **FRAMES**    **NO FRAMES**     **All Classes** |
| SUMMARY: NESTED | FIELD | CONSTR | METHOD | DETAIL: FIELD | CONSTR | METHOD |


---
