# Supplementary material for: BSim: An Agent-Based Tool for Modeling Bacterial Populations in Systems and Synthetic Biology
Source: PLoS One. 2012 Aug 24;7(8):e42790. doi: 10.1371/journal.pone.0042790 (PMC3427305; doi:10.1371/journal.pone.0042790)
Supplement: Software S1 — Snapshot of the BSim software from 18th July 2012. For the latest version see: http://bsim-bccs.sf.net. The BSim software requires Java version 1.6 or higher. (ZIP) [file pone.0042790.s014.zip › BSimSoftware/docs/javadoc/bsim/geometry/BSimTriangle.html]

BSimTriangle


---


|  |  |  |  |  |  |  |  |  |  |  |
| --- | --- | --- | --- | --- | --- | --- | --- | --- | --- | --- |
| |  |  |  |  |  |  |  |  | | --- | --- | --- | --- | --- | --- | --- | --- | | **Overview** | **Package** | **Class** | **Use** | **Tree** | **Deprecated** | **Index** | **Help** | | |  |
| **PREV CLASS**   **NEXT CLASS** | **FRAMES**    **NO FRAMES**     **All Classes** |
| SUMMARY: NESTED | FIELD | CONSTR | METHOD | DETAIL: FIELD | CONSTR | METHOD |


---


## bsim.geometry Class BSimTriangle

```
java.lang.Object
  bsim.geometry.BSimTriangle
```

---

``` public class BSimTriangle extends java.lang.Object ```

Triangular face of a 3-D mesh surface.
Stores the indices of its three vertices, and a cached face normal vector.

---

| **Field Summary** | |
| --- | --- |
| `protected  javax.vecmath.Vector3d` | `normal`             Triangle normal. |
| `protected  BSimMesh` | `parentMesh`             The mesh to which this triangle belongs. |
| `protected  long` | `timeStamp`             Time stamp. |
| `protected  int[]` | `tVertices` |


| **Constructor Summary** | |
| --- | --- |
| `BSimTriangle(BSimTriangle tri)` |
| `BSimTriangle(int[] newPoints, BSimMesh mesh)`             Constructor: New triangular face from array of vertex indices. |
| `BSimTriangle(int newP1Index, int newP2Index, int newP3Index, BSimMesh mesh)`             Constructor: New triangular face from three individual vertex indices. |


| **Method Summary** | |
| --- | --- |
| `protected  void` | `flipNormal()`             Flip the face normal if you want it to point the other way. |
| `javax.vecmath.Vector3d` | `getNormal()`             Gets the triangle normal vector. |
| `int` | `getP1()` |
| `int` | `getP2()` |
| `int` | `getP3()` |
| `BSimMesh` | `getParentMesh()` |
| `int[]` | `getPoints()` |
| `long` | `getTimeStamp()` |
| `javax.vecmath.Vector3d` | `getVertCoords(int i)`             Get the vertex coordinates of a given triangle |
| `protected  void` | `setMesh(BSimMesh mesh)`             Set parent mesh to which this face belongs. |
| `void` | `setTimeStamp(long newTimeStamp)`             Set the time stamp associated with the triangle. |
| `protected  void` | `updateNormal(javax.vecmath.Vector3d newNormal)`             Update the normal vector of this face with a new vector. |

| **Methods inherited from class java.lang.Object** |
| --- |
| `clone, equals, finalize, getClass, hashCode, notify, notifyAll, toString, wait, wait, wait` |

| **Field Detail** |
| --- |

### tVertices

```
protected int[] tVertices
```

---


### normal

```
protected javax.vecmath.Vector3d normal
```

:   Triangle normal. Assumed to be normalised on creation.

---


### parentMesh

```
protected BSimMesh parentMesh
```

:   The mesh to which this triangle belongs.

---


### timeStamp

```
protected long timeStamp
```

:   Time stamp. Used in collision calculations.


| **Constructor Detail** |
| --- |

### BSimTriangle

```
public BSimTriangle(int newP1Index,
                    int newP2Index,
                    int newP3Index,
                    BSimMesh mesh)
```

:   Constructor: New triangular face from three individual vertex indices.

---


### BSimTriangle

```
public BSimTriangle(int[] newPoints,
                    BSimMesh mesh)
```

:   Constructor: New triangular face from array of vertex indices.

---


### BSimTriangle

```
public BSimTriangle(BSimTriangle tri)
```


| **Method Detail** |
| --- |

### updateNormal

```
protected void updateNormal(javax.vecmath.Vector3d newNormal)
```

:   Update the normal vector of this face with a new vector.

---


### flipNormal

```
protected void flipNormal()
```

:   Flip the face normal if you want it to point the other way.

---


### getVertCoords

```
public javax.vecmath.Vector3d getVertCoords(int i)
```

:   Get the vertex coordinates of a given triangle

    :   **Parameters:**: `t` -: `i` - Index of the vertex for which to get coordinates (0, 1, 2) **Returns:**

---


### setMesh

```
protected void setMesh(BSimMesh mesh)
```

:   Set parent mesh to which this face belongs.

    :   **Parameters:**: `mesh` - Parent BSimMesh

---


### setTimeStamp

```
public void setTimeStamp(long newTimeStamp)
```

:   Set the time stamp associated with the triangle.

---


### getNormal

```
public javax.vecmath.Vector3d getNormal()
```

:   Gets the triangle normal vector. Assumed normalised in BSimMesh

---


### getTimeStamp

```
public long getTimeStamp()
```

---


### getParentMesh

```
public BSimMesh getParentMesh()
```

---


### getP1

```
public int getP1()
```

---


### getP2

```
public int getP2()
```

---


### getP3

```
public int getP3()
```

---


### getPoints

```
public int[] getPoints()
```


---


|  |  |  |  |  |  |  |  |  |  |  |
| --- | --- | --- | --- | --- | --- | --- | --- | --- | --- | --- |
| |  |  |  |  |  |  |  |  | | --- | --- | --- | --- | --- | --- | --- | --- | | **Overview** | **Package** | **Class** | **Use** | **Tree** | **Deprecated** | **Index** | **Help** | | |  |
| **PREV CLASS**   **NEXT CLASS** | **FRAMES**    **NO FRAMES**     **All Classes** |
| SUMMARY: NESTED | FIELD | CONSTR | METHOD | DETAIL: FIELD | CONSTR | METHOD |


---
