# Supplementary material for: BSim: An Agent-Based Tool for Modeling Bacterial Populations in Systems and Synthetic Biology
Source: PLoS One. 2012 Aug 24;7(8):e42790. doi: 10.1371/journal.pone.0042790 (PMC3427305; doi:10.1371/journal.pone.0042790)
Supplement: Software S1 — Snapshot of the BSim software from 18th July 2012. For the latest version see: http://bsim-bccs.sf.net. The BSim software requires Java version 1.6 or higher. (ZIP) [file pone.0042790.s014.zip › BSimSoftware/docs/javadoc/bsim/geometry/BSimVertex.html]

BSimVertex


---


|  |  |  |  |  |  |  |  |  |  |  |
| --- | --- | --- | --- | --- | --- | --- | --- | --- | --- | --- |
| |  |  |  |  |  |  |  |  | | --- | --- | --- | --- | --- | --- | --- | --- | | **Overview** | **Package** | **Class** | **Use** | **Tree** | **Deprecated** | **Index** | **Help** | | |  |
| **PREV CLASS**   **NEXT CLASS** | **FRAMES**    **NO FRAMES**     **All Classes** |
| SUMMARY: NESTED | FIELD | CONSTR | METHOD | DETAIL: FIELD | CONSTR | METHOD |


---


## bsim.geometry Class BSimVertex

```
java.lang.Object
  bsim.geometry.BSimVertex
```

---

``` public class BSimVertex extends java.lang.Object ```

Mesh vertex.
Defines the location of a mesh vertex in 3-D space as a Vector3d,
and also stores a cached list of the faces which are attached to
this vertex.

---

| **Field Summary** | |
| --- | --- |
| `protected  java.util.ArrayList<java.lang.Integer>` | `faces`             List of the indices of the faces which use this vertex. |
| `protected  javax.vecmath.Vector3d` | `location`             The Cartesian coordinates of the vertex in 3-D space. |


| **Constructor Summary** | |
| --- | --- |
| `BSimVertex(double newX, double newY, double newZ)`             Constructor: create a new mesh vertex from three points; x,y,z |
| `BSimVertex(javax.vecmath.Vector3d newLocation)`             Constructor: create a new mesh vertex from a Vector3d |


| **Method Summary** | |
| --- | --- |
| `java.util.ArrayList<java.lang.Integer>` | `getFaces()` |
| `javax.vecmath.Vector3d` | `getLocation()` |

| **Methods inherited from class java.lang.Object** |
| --- |
| `clone, equals, finalize, getClass, hashCode, notify, notifyAll, toString, wait, wait, wait` |

| **Field Detail** |
| --- |

### location

```
protected javax.vecmath.Vector3d location
```

:   The Cartesian coordinates of the vertex in 3-D space.

---


### faces

```
protected java.util.ArrayList<java.lang.Integer> faces
```

:   List of the indices of the faces which use this vertex.
    Currently this is not updated from within BSimVertex but from the
    surface mesh implementation (e.g BSimFVMesh).


| **Constructor Detail** |
| --- |

### BSimVertex

```
public BSimVertex(double newX,
                  double newY,
                  double newZ)
```

:   Constructor: create a new mesh vertex from three points; x,y,z

---


### BSimVertex

```
public BSimVertex(javax.vecmath.Vector3d newLocation)
```

:   Constructor: create a new mesh vertex from a Vector3d


| **Method Detail** |
| --- |

### getLocation

```
public javax.vecmath.Vector3d getLocation()
```

---


### getFaces

```
public java.util.ArrayList<java.lang.Integer> getFaces()
```


---


|  |  |  |  |  |  |  |  |  |  |  |
| --- | --- | --- | --- | --- | --- | --- | --- | --- | --- | --- |
| |  |  |  |  |  |  |  |  | | --- | --- | --- | --- | --- | --- | --- | --- | | **Overview** | **Package** | **Class** | **Use** | **Tree** | **Deprecated** | **Index** | **Help** | | |  |
| **PREV CLASS**   **NEXT CLASS** | **FRAMES**    **NO FRAMES**     **All Classes** |
| SUMMARY: NESTED | FIELD | CONSTR | METHOD | DETAIL: FIELD | CONSTR | METHOD |


---
