# Supplementary material for: BSim: An Agent-Based Tool for Modeling Bacterial Populations in Systems and Synthetic Biology
Source: PLoS One. 2012 Aug 24;7(8):e42790. doi: 10.1371/journal.pone.0042790 (PMC3427305; doi:10.1371/journal.pone.0042790)
Supplement: Software S1 — Snapshot of the BSim software from 18th July 2012. For the latest version see: http://bsim-bccs.sf.net. The BSim software requires Java version 1.6 or higher. (ZIP) [file pone.0042790.s014.zip › BSimSoftware/docs/javadoc/bsim/geometry/class-use/BSimCollision.html]

Uses of Class bsim.geometry.BSimCollision


---


|  |  |  |  |  |  |  |  |  |  |  |
| --- | --- | --- | --- | --- | --- | --- | --- | --- | --- | --- |
| |  |  |  |  |  |  |  |  | | --- | --- | --- | --- | --- | --- | --- | --- | | **Overview** | **Package** | **Class** | **Use** | **Tree** | **Deprecated** | **Index** | **Help** | | |  |
| PREV   NEXT | **FRAMES**    **NO FRAMES**     **All Classes** |


---


## **Uses of Class bsim.geometry.BSimCollision**

| Packages that use BSimCollision | |
| --- | --- |
| **bsim.geometry** |  |

| Uses of BSimCollision in bsim.geometry | |
| --- | --- |

| Methods in bsim.geometry that return BSimCollision | |
| --- | --- |
| `static BSimCollision` | `BSimMeshUtils.intersectVectorPlane(javax.vecmath.Vector3d p1, javax.vecmath.Vector3d direction, javax.vecmath.Vector3d normalPlane, double dPlane)`             Compute the intersection of a vector p1 + t\*dir (line segment) and a plane |

| Methods in bsim.geometry with parameters of type BSimCollision | |
| --- | --- |
| `static boolean` | `BSimMeshUtils.intersectVectorTriangle(javax.vecmath.Vector3d startPos, javax.vecmath.Vector3d endPos, BSimTriangle tri, BSimCollision coll)`             Computes intersection of a vector in 3d space (e.g. |
| `void` | `BSimCollision.set(BSimCollision col)`             Set the collision values. |

---


|  |  |  |  |  |  |  |  |  |  |  |
| --- | --- | --- | --- | --- | --- | --- | --- | --- | --- | --- |
| |  |  |  |  |  |  |  |  | | --- | --- | --- | --- | --- | --- | --- | --- | | **Overview** | **Package** | **Class** | **Use** | **Tree** | **Deprecated** | **Index** | **Help** | | |  |
| PREV   NEXT | **FRAMES**    **NO FRAMES**     **All Classes** |


---
