# Supplementary material for: BSim: An Agent-Based Tool for Modeling Bacterial Populations in Systems and Synthetic Biology
Source: PLoS One. 2012 Aug 24;7(8):e42790. doi: 10.1371/journal.pone.0042790 (PMC3427305; doi:10.1371/journal.pone.0042790)
Supplement: Software S1 — Snapshot of the BSim software from 18th July 2012. For the latest version see: http://bsim-bccs.sf.net. The BSim software requires Java version 1.6 or higher. (ZIP) [file pone.0042790.s014.zip › BSimSoftware/docs/javadoc/bsim/geometry/class-use/BSimMesh.html]

Uses of Class bsim.geometry.BSimMesh


---


|  |  |  |  |  |  |  |  |  |  |  |
| --- | --- | --- | --- | --- | --- | --- | --- | --- | --- | --- |
| |  |  |  |  |  |  |  |  | | --- | --- | --- | --- | --- | --- | --- | --- | | **Overview** | **Package** | **Class** | **Use** | **Tree** | **Deprecated** | **Index** | **Help** | | |  |
| PREV   NEXT | **FRAMES**    **NO FRAMES**     **All Classes** |


---


## **Uses of Class bsim.geometry.BSimMesh**

| Packages that use BSimMesh | |
| --- | --- |
| **bsim** |  |
| **bsim.draw** |  |
| **bsim.geometry** |  |

| Uses of BSimMesh in bsim | |
| --- | --- |

| Methods in bsim with parameters of type BSimMesh | |
| --- | --- |
| `void` | `BSimOctreeField.setNodestoMesh(BSimMesh theMesh, BSimOctreeField t)`             Fits octreeField against a mesh and splits into subNodes when there is a collision with the mesh boundary Creates a finer octree structure each time this function is called. |

| Uses of BSimMesh in bsim.draw | |
| --- | --- |

| Methods in bsim.draw with parameters of type BSimMesh | |
| --- | --- |
| `void` | `BSimP3DDrawer.draw(BSimMesh mesh, java.awt.Color c, double normalScaleFactor)`             Draw a mesh with a given colour (draws each triangle of the mesh individually). |
| `void` | `BSimP3DDrawer.draw(BSimMesh mesh, double normalScaleFactor)`             Draw a mesh, default colour. |

| Uses of BSimMesh in bsim.geometry | |
| --- | --- |

| Subclasses of BSimMesh in bsim.geometry | |
| --- | --- |
| `class` | `BSimOBJMesh`             Wavefront OBJ importer. |
| `class` | `BSimSphereMesh`             Sphere mesh, uses face-vertex representation. |
| `(package private)  class` | `KdNode.TestMesh` |

| Fields in bsim.geometry declared as BSimMesh | |
| --- | --- |
| `BSimMesh` | `KdNode.parentMesh` |
| `protected  BSimMesh` | `BSimTriangle.parentMesh`             The mesh to which this triangle belongs. |

| Methods in bsim.geometry that return BSimMesh | |
| --- | --- |
| `BSimMesh` | `BSimTriangle.getParentMesh()` |

| Methods in bsim.geometry with parameters of type BSimMesh | |
| --- | --- |
| `static boolean` | `BSimCollision.collideAndCross(javax.vecmath.Vector3d p1, javax.vecmath.Vector3d p2, BSimMesh theMesh)`             Check if mesh is crossed. |
| `static void` | `BSimCollision.collideAndReflect(javax.vecmath.Vector3d p1, javax.vecmath.Vector3d p2, BSimMesh theMesh)`             Check to see if intersection with mesh and reflect. |
| `static void` | `BSimCollision.collideAndRepel(BSimParticle p, BSimMesh theMesh)`             Check for collision between particle and mesh, and add repulsion force. |
| `KdNode` | `KdNode.kdTreeFromMesh(BSimMesh theMesh)` |
| `KdNode` | `KdNode.makeTree(BSimMesh theMesh, KdNode.Indexed3d[] points, int depth)` |
| `protected  void` | `BSimTriangle.setMesh(BSimMesh mesh)`             Set parent mesh to which this face belongs. |

| Constructors in bsim.geometry with parameters of type BSimMesh | |
| --- | --- |
| `BSimTriangle(int[] newPoints, BSimMesh mesh)`             Constructor: New triangular face from array of vertex indices. |
| `BSimTriangle(int newP1Index, int newP2Index, int newP3Index, BSimMesh mesh)`             Constructor: New triangular face from three individual vertex indices. |

---


|  |  |  |  |  |  |  |  |  |  |  |
| --- | --- | --- | --- | --- | --- | --- | --- | --- | --- | --- |
| |  |  |  |  |  |  |  |  | | --- | --- | --- | --- | --- | --- | --- | --- | | **Overview** | **Package** | **Class** | **Use** | **Tree** | **Deprecated** | **Index** | **Help** | | |  |
| PREV   NEXT | **FRAMES**    **NO FRAMES**     **All Classes** |


---
