# Supplementary material for: BSim: An Agent-Based Tool for Modeling Bacterial Populations in Systems and Synthetic Biology
Source: PLoS One. 2012 Aug 24;7(8):e42790. doi: 10.1371/journal.pone.0042790 (PMC3427305; doi:10.1371/journal.pone.0042790)
Supplement: Software S1 — Snapshot of the BSim software from 18th July 2012. For the latest version see: http://bsim-bccs.sf.net. The BSim software requires Java version 1.6 or higher. (ZIP) [file pone.0042790.s014.zip › BSimSoftware/docs/javadoc/bsim/geometry/class-use/BSimTriangle.html]

Uses of Class bsim.geometry.BSimTriangle


---


|  |  |  |  |  |  |  |  |  |  |  |
| --- | --- | --- | --- | --- | --- | --- | --- | --- | --- | --- |
| |  |  |  |  |  |  |  |  | | --- | --- | --- | --- | --- | --- | --- | --- | | **Overview** | **Package** | **Class** | **Use** | **Tree** | **Deprecated** | **Index** | **Help** | | |  |
| PREV   NEXT | **FRAMES**    **NO FRAMES**     **All Classes** |


---


## **Uses of Class bsim.geometry.BSimTriangle**

| Packages that use BSimTriangle | |
| --- | --- |
| **bsim** |  |
| **bsim.geometry** |  |

| Uses of BSimTriangle in bsim | |
| --- | --- |

| Methods in bsim with parameters of type BSimTriangle | |
| --- | --- |
| `static boolean` | `BSimOctreeField.intersectVectorTriangle(javax.vecmath.Vector3d startPos, javax.vecmath.Vector3d endPos, BSimTriangle tri)` |

| Uses of BSimTriangle in bsim.geometry | |
| --- | --- |

| Fields in bsim.geometry with type parameters of type BSimTriangle | |
| --- | --- |
| `protected  java.util.ArrayList<BSimTriangle>` | `BSimMesh.faces`             List of faces, each face stores the indices of the vertices which compose that face. |

| Methods in bsim.geometry that return BSimTriangle | |
| --- | --- |
| `BSimTriangle` | `BSimMesh.getFace(int i)` |

| Methods in bsim.geometry that return types with arguments of type BSimTriangle | |
| --- | --- |
| `java.util.ArrayList<BSimTriangle>` | `BSimMesh.getFaces()` |

| Methods in bsim.geometry with parameters of type BSimTriangle | |
| --- | --- |
| `void` | `BSimMesh.addTriangle(BSimTriangle t)`             Add an existing triangle to the face list |
| `void` | `BSimMesh.computeNormal(BSimTriangle t)`             Compute the normal vector of a face. |
| `javax.vecmath.Vector3d` | `BSimMesh.getTCentre(BSimTriangle t)`             Compute the coordinates of the centre of a triangle |
| `javax.vecmath.Vector3d` | `BSimMesh.getVertCoordsOfTri(BSimTriangle t, int i)`             Get the vertex coordinates of a given triangle |
| `static boolean` | `BSimMeshUtils.intersectTriangleAAB(BSimTriangle t, javax.vecmath.Vector3d boxCentre, javax.vecmath.Vector3d boxDim)`             Test for intersection of a triangle against an axis aligned box |
| `static boolean` | `BSimMeshUtils.intersectTriOctreeNode(BSimTriangle t, BSimOctreeField n)`             Test for intersection of a triangle against an octree node |
| `static boolean` | `BSimMeshUtils.intersectVectorTriangle(javax.vecmath.Vector3d startPos, javax.vecmath.Vector3d endPos, BSimTriangle tri, BSimCollision coll)`             Computes intersection of a vector in 3d space (e.g. |
| `void` | `BSimCollision.set(BSimTriangle tri, double tVal, double u, double v, double w)` |

| Constructors in bsim.geometry with parameters of type BSimTriangle | |
| --- | --- |
| `BSimTriangle(BSimTriangle tri)` |

---


|  |  |  |  |  |  |  |  |  |  |  |
| --- | --- | --- | --- | --- | --- | --- | --- | --- | --- | --- |
| |  |  |  |  |  |  |  |  | | --- | --- | --- | --- | --- | --- | --- | --- | | **Overview** | **Package** | **Class** | **Use** | **Tree** | **Deprecated** | **Index** | **Help** | | |  |
| PREV   NEXT | **FRAMES**    **NO FRAMES**     **All Classes** |


---
