# Supplementary material for: BSim: An Agent-Based Tool for Modeling Bacterial Populations in Systems and Synthetic Biology
Source: PLoS One. 2012 Aug 24;7(8):e42790. doi: 10.1371/journal.pone.0042790 (PMC3427305; doi:10.1371/journal.pone.0042790)
Supplement: Software S1 — Snapshot of the BSim software from 18th July 2012. For the latest version see: http://bsim-bccs.sf.net. The BSim software requires Java version 1.6 or higher. (ZIP) [file pone.0042790.s014.zip › BSimSoftware/docs/javadoc/bsim/geometry/class-use/BSimVertex.html]

Uses of Class bsim.geometry.BSimVertex


---


|  |  |  |  |  |  |  |  |  |  |  |
| --- | --- | --- | --- | --- | --- | --- | --- | --- | --- | --- |
| |  |  |  |  |  |  |  |  | | --- | --- | --- | --- | --- | --- | --- | --- | | **Overview** | **Package** | **Class** | **Use** | **Tree** | **Deprecated** | **Index** | **Help** | | |  |
| PREV   NEXT | **FRAMES**    **NO FRAMES**     **All Classes** |


---


## **Uses of Class bsim.geometry.BSimVertex**

| Packages that use BSimVertex | |
| --- | --- |
| **bsim.geometry** |  |

| Uses of BSimVertex in bsim.geometry | |
| --- | --- |

| Fields in bsim.geometry with type parameters of type BSimVertex | |
| --- | --- |
| `protected  java.util.ArrayList<BSimVertex>` | `BSimMesh.vertices`             The actual locations (3D coordinates) of all mesh vertices |

| Methods in bsim.geometry that return BSimVertex | |
| --- | --- |
| `BSimVertex` | `BSimMesh.getVertex(int i)` |

| Methods in bsim.geometry that return types with arguments of type BSimVertex | |
| --- | --- |
| `java.util.ArrayList<BSimVertex>` | `BSimMesh.getVertices()` |

---


|  |  |  |  |  |  |  |  |  |  |  |
| --- | --- | --- | --- | --- | --- | --- | --- | --- | --- | --- |
| |  |  |  |  |  |  |  |  | | --- | --- | --- | --- | --- | --- | --- | --- | | **Overview** | **Package** | **Class** | **Use** | **Tree** | **Deprecated** | **Index** | **Help** | | |  |
| PREV   NEXT | **FRAMES**    **NO FRAMES**     **All Classes** |


---
