# Supplementary material for: BSim: An Agent-Based Tool for Modeling Bacterial Populations in Systems and Synthetic Biology
Source: PLoS One. 2012 Aug 24;7(8):e42790. doi: 10.1371/journal.pone.0042790 (PMC3427305; doi:10.1371/journal.pone.0042790)
Supplement: Software S1 — Snapshot of the BSim software from 18th July 2012. For the latest version see: http://bsim-bccs.sf.net. The BSim software requires Java version 1.6 or higher. (ZIP) [file pone.0042790.s014.zip › BSimSoftware/docs/javadoc/bsim/geometry/class-use/KdNode.html]

Uses of Class bsim.geometry.KdNode


---


|  |  |  |  |  |  |  |  |  |  |  |
| --- | --- | --- | --- | --- | --- | --- | --- | --- | --- | --- |
| |  |  |  |  |  |  |  |  | | --- | --- | --- | --- | --- | --- | --- | --- | | **Overview** | **Package** | **Class** | **Use** | **Tree** | **Deprecated** | **Index** | **Help** | | |  |
| PREV   NEXT | **FRAMES**    **NO FRAMES**     **All Classes** |


---


## **Uses of Class bsim.geometry.KdNode**

| Packages that use KdNode | |
| --- | --- |
| **bsim.geometry** |  |

| Uses of KdNode in bsim.geometry | |
| --- | --- |

| Fields in bsim.geometry declared as KdNode | |
| --- | --- |
| `KdNode` | `KdNode.leftChild`             Left child: coordinate of interest < splitting plane |
| `KdNode` | `KdNode.rightChild`             Right child: coordinate of interest > splitting plane |

| Methods in bsim.geometry that return KdNode | |
| --- | --- |
| `KdNode` | `KdNode.kdTreeFromMesh(BSimMesh theMesh)` |
| `KdNode` | `KdNode.kdTreeMeshTest()` |
| `KdNode` | `KdNode.makeTree(BSimMesh theMesh, KdNode.Indexed3d[] points, int depth)` |

| Methods in bsim.geometry with parameters of type KdNode | |
| --- | --- |
| `void` | `KdNode.assignTrianglesFromParentMesh(KdNode kn)`             Assigns triangles from the parent mesh of the k-d tree to each (leaf) node. |
| `static java.util.ArrayList<java.util.ArrayList<java.lang.Integer>>` | `KdNode.intersectVectorKdNode(javax.vecmath.Vector3d p1, javax.vecmath.Vector3d p2, KdNode theKdNode)`             Intersect a direction vector segment with KdNode (or hierarchy) |

---


|  |  |  |  |  |  |  |  |  |  |  |
| --- | --- | --- | --- | --- | --- | --- | --- | --- | --- | --- |
| |  |  |  |  |  |  |  |  | | --- | --- | --- | --- | --- | --- | --- | --- | | **Overview** | **Package** | **Class** | **Use** | **Tree** | **Deprecated** | **Index** | **Help** | | |  |
| PREV   NEXT | **FRAMES**    **NO FRAMES**     **All Classes** |


---
