# Supplementary material for: BSim: An Agent-Based Tool for Modeling Bacterial Populations in Systems and Synthetic Biology
Source: PLoS One. 2012 Aug 24;7(8):e42790. doi: 10.1371/journal.pone.0042790 (PMC3427305; doi:10.1371/journal.pone.0042790)
Supplement: Software S1 — Snapshot of the BSim software from 18th July 2012. For the latest version see: http://bsim-bccs.sf.net. The BSim software requires Java version 1.6 or higher. (ZIP) [file pone.0042790.s014.zip › BSimSoftware/docs/javadoc/bsim/geometry/class-use/KdNode.Indexed3d.html]

Uses of Class bsim.geometry.KdNode.Indexed3d


---


|  |  |  |  |  |  |  |  |  |  |  |
| --- | --- | --- | --- | --- | --- | --- | --- | --- | --- | --- |
| |  |  |  |  |  |  |  |  | | --- | --- | --- | --- | --- | --- | --- | --- | | **Overview** | **Package** | **Class** | **Use** | **Tree** | **Deprecated** | **Index** | **Help** | | |  |
| PREV   NEXT | **FRAMES**    **NO FRAMES**     **All Classes** |


---


## **Uses of Class bsim.geometry.KdNode.Indexed3d**

| Packages that use KdNode.Indexed3d | |
| --- | --- |
| **bsim.geometry** |  |

| Uses of KdNode.Indexed3d in bsim.geometry | |
| --- | --- |

| Methods in bsim.geometry that return KdNode.Indexed3d | |
| --- | --- |
| `KdNode.Indexed3d[]` | `KdNode.getSubSet(KdNode.Indexed3d[] fullSet, int start, int end)` |

| Methods in bsim.geometry with parameters of type KdNode.Indexed3d | |
| --- | --- |
| `void` | `KdNode.findMedian(KdNode.Indexed3d[] dList, int axis, int firstIndex, int lastIndex, int medianPos)` |
| `KdNode.Indexed3d[]` | `KdNode.getSubSet(KdNode.Indexed3d[] fullSet, int start, int end)` |
| `KdNode` | `KdNode.makeTree(BSimMesh theMesh, KdNode.Indexed3d[] points, int depth)` |
| `int` | `KdNode.partition(KdNode.Indexed3d[] dList, int axis, int firstIndex, int lastIndex, int pivotIndex)` |
| `void` | `KdNode.Indexed3d.set(KdNode.Indexed3d id)` |

| Constructors in bsim.geometry with parameters of type KdNode.Indexed3d | |
| --- | --- |
| `KdNode.Indexed3d(KdNode.Indexed3d id)` |

---


|  |  |  |  |  |  |  |  |  |  |  |
| --- | --- | --- | --- | --- | --- | --- | --- | --- | --- | --- |
| |  |  |  |  |  |  |  |  | | --- | --- | --- | --- | --- | --- | --- | --- | | **Overview** | **Package** | **Class** | **Use** | **Tree** | **Deprecated** | **Index** | **Help** | | |  |
| PREV   NEXT | **FRAMES**    **NO FRAMES**     **All Classes** |


---
