# Supplementary material for: BSim: An Agent-Based Tool for Modeling Bacterial Populations in Systems and Synthetic Biology
Source: PLoS One. 2012 Aug 24;7(8):e42790. doi: 10.1371/journal.pone.0042790 (PMC3427305; doi:10.1371/journal.pone.0042790)
Supplement: Software S1 — Snapshot of the BSim software from 18th July 2012. For the latest version see: http://bsim-bccs.sf.net. The BSim software requires Java version 1.6 or higher. (ZIP) [file pone.0042790.s014.zip › BSimSoftware/docs/javadoc/bsim/geometry/KdNode.html]

KdNode


---


|  |  |  |  |  |  |  |  |  |  |  |
| --- | --- | --- | --- | --- | --- | --- | --- | --- | --- | --- |
| |  |  |  |  |  |  |  |  | | --- | --- | --- | --- | --- | --- | --- | --- | | **Overview** | **Package** | **Class** | **Use** | **Tree** | **Deprecated** | **Index** | **Help** | | |  |
| **PREV CLASS**   **NEXT CLASS** | **FRAMES**    **NO FRAMES**     **All Classes** |
| SUMMARY: NESTED | FIELD | CONSTR | METHOD | DETAIL: FIELD | CONSTR | METHOD |


---


## bsim.geometry Class KdNode

```
java.lang.Object
  bsim.geometry.KdNode
```

---

``` public class KdNode extends java.lang.Object ```

See, for example, http://en.wikipedia.org/wiki/Kd-tree
Effectively an axis-aligned BSP tree which alternates the splitting plane axis at each level of branching.
Median point finding via quick-select algorithm.

---

| **Nested Class Summary** | |
| --- | --- |
| `(package private)  class` | `KdNode.Indexed3d`             Class that holds a triplet of doubles and its original index. |
| `(package private)  class` | `KdNode.TestMesh` |


| **Field Summary** | |
| --- | --- |
| `KdNode` | `leftChild`             Left child: coordinate of interest < splitting plane |
| `java.util.ArrayList<java.lang.Integer>` | `leftTris`             Triangles that are classified as being to the left of the node. |
| `int` | `location`             Index of the vertex on which the splitting plane is located |
| `int` | `nodeAxis`             Axis normal to the splitting plane (0 = x, 1 = y, 2 = z) |
| `BSimMesh` | `parentMesh` |
| `KdNode` | `rightChild`             Right child: coordinate of interest > splitting plane |
| `java.util.ArrayList<java.lang.Integer>` | `rightTris`             Triangles that are classified as being to the right of this node. |


| **Constructor Summary** | |
| --- | --- |
| `KdNode()`             Default constructor. |


| **Method Summary** | |
| --- | --- |
| `void` | `assignTrianglesFromParentMesh(KdNode kn)`             Assigns triangles from the parent mesh of the k-d tree to each (leaf) node. |
| `void` | `findMedian(KdNode.Indexed3d[] dList, int axis, int firstIndex, int lastIndex, int medianPos)` |
| `KdNode.Indexed3d[]` | `getSubSet(KdNode.Indexed3d[] fullSet, int start, int end)` |
| `boolean` | `hasLeftChild()` |
| `boolean` | `hasRightChild()` |
| `static java.util.ArrayList<java.util.ArrayList<java.lang.Integer>>` | `intersectVectorKdNode(javax.vecmath.Vector3d p1, javax.vecmath.Vector3d p2, KdNode theKdNode)`             Intersect a direction vector segment with KdNode (or hierarchy) |
| `KdNode` | `kdTreeFromMesh(BSimMesh theMesh)` |
| `KdNode` | `kdTreeMeshTest()` |
| `static void` | `main(java.lang.String[] args)` |
| `KdNode` | `makeTree(BSimMesh theMesh, KdNode.Indexed3d[] points, int depth)` |
| `int` | `partition(KdNode.Indexed3d[] dList, int axis, int firstIndex, int lastIndex, int pivotIndex)` |
| `static double` | `vecGetCoord(javax.vecmath.Vector3d v, int i)`             ...GHH |

| **Methods inherited from class java.lang.Object** |
| --- |
| `clone, equals, finalize, getClass, hashCode, notify, notifyAll, toString, wait, wait, wait` |

| **Field Detail** |
| --- |

### location

```
public int location
```

:   Index of the vertex on which the splitting plane is located

---


### nodeAxis

```
public int nodeAxis
```

:   Axis normal to the splitting plane (0 = x, 1 = y, 2 = z)

---


### leftChild

```
public KdNode leftChild
```

:   Left child: coordinate of interest < splitting plane

---


### rightChild

```
public KdNode rightChild
```

:   Right child: coordinate of interest > splitting plane

---


### leftTris

```
public java.util.ArrayList<java.lang.Integer> leftTris
```

:   Triangles that are classified as being to the left of the node.

---


### rightTris

```
public java.util.ArrayList<java.lang.Integer> rightTris
```

:   Triangles that are classified as being to the right of this node.

---


### parentMesh

```
public BSimMesh parentMesh
```


| **Constructor Detail** |
| --- |

### KdNode

```
public KdNode()
```

:   Default constructor.


| **Method Detail** |
| --- |

### hasLeftChild

```
public boolean hasLeftChild()
```

---


### hasRightChild

```
public boolean hasRightChild()
```

---


### vecGetCoord

```
public static double vecGetCoord(javax.vecmath.Vector3d v,
                                 int i)
```

:   ...GHH

---


### intersectVectorKdNode

```
public static java.util.ArrayList<java.util.ArrayList<java.lang.Integer>> intersectVectorKdNode(javax.vecmath.Vector3d p1,
                                                                                                javax.vecmath.Vector3d p2,
                                                                                                KdNode theKdNode)
```

:   Intersect a direction vector segment with KdNode (or hierarchy)

    :   **Parameters:**: `p1` -

---


### main

```
public static void main(java.lang.String[] args)
```

---


### findMedian

```
public void findMedian(KdNode.Indexed3d[] dList,
                       int axis,
                       int firstIndex,
                       int lastIndex,
                       int medianPos)
```

---


### partition

```
public int partition(KdNode.Indexed3d[] dList,
                     int axis,
                     int firstIndex,
                     int lastIndex,
                     int pivotIndex)
```

---


### kdTreeMeshTest

```
public KdNode kdTreeMeshTest()
```

---


### kdTreeFromMesh

```
public KdNode kdTreeFromMesh(BSimMesh theMesh)
```

---


### assignTrianglesFromParentMesh

```
public void assignTrianglesFromParentMesh(KdNode kn)
```

:   Assigns triangles from the parent mesh of the k-d tree to each (leaf) node.

---


### makeTree

```
public KdNode makeTree(BSimMesh theMesh,
                       KdNode.Indexed3d[] points,
                       int depth)
```

---


### getSubSet

```
public KdNode.Indexed3d[] getSubSet(KdNode.Indexed3d[] fullSet,
                                    int start,
                                    int end)
```


---


|  |  |  |  |  |  |  |  |  |  |  |
| --- | --- | --- | --- | --- | --- | --- | --- | --- | --- | --- |
| |  |  |  |  |  |  |  |  | | --- | --- | --- | --- | --- | --- | --- | --- | | **Overview** | **Package** | **Class** | **Use** | **Tree** | **Deprecated** | **Index** | **Help** | | |  |
| **PREV CLASS**   **NEXT CLASS** | **FRAMES**    **NO FRAMES**     **All Classes** |
| SUMMARY: NESTED | FIELD | CONSTR | METHOD | DETAIL: FIELD | CONSTR | METHOD |


---
