# Supplementary material for: BSim: An Agent-Based Tool for Modeling Bacterial Populations in Systems and Synthetic Biology
Source: PLoS One. 2012 Aug 24;7(8):e42790. doi: 10.1371/journal.pone.0042790 (PMC3427305; doi:10.1371/journal.pone.0042790)
Supplement: Software S1 — Snapshot of the BSim software from 18th July 2012. For the latest version see: http://bsim-bccs.sf.net. The BSim software requires Java version 1.6 or higher. (ZIP) [file pone.0042790.s014.zip › BSimSoftware/docs/javadoc/bsim/geometry/KdNode.Indexed3d.html]

KdNode.Indexed3d


---


|  |  |  |  |  |  |  |  |  |  |  |
| --- | --- | --- | --- | --- | --- | --- | --- | --- | --- | --- |
| |  |  |  |  |  |  |  |  | | --- | --- | --- | --- | --- | --- | --- | --- | | **Overview** | **Package** | **Class** | **Use** | **Tree** | **Deprecated** | **Index** | **Help** | | |  |
| **PREV CLASS**   **NEXT CLASS** | **FRAMES**    **NO FRAMES**     **All Classes** |
| SUMMARY: NESTED | FIELD | CONSTR | METHOD | DETAIL: FIELD | CONSTR | METHOD |


---


## bsim.geometry Class KdNode.Indexed3d

```
java.lang.Object
  bsim.geometry.KdNode.Indexed3d
```

**Enclosing class:**: KdNode

---

``` class KdNode.Indexed3d extends java.lang.Object ```

Class that holds a triplet of doubles and its original index.
Thus we can sort by value then refer back to the original object,
without rearranging order of original data.

---

| **Field Summary** | |
| --- | --- |
| `(package private)  int` | `originalIndex` |
| `(package private)  double[]` | `val` |


| **Constructor Summary** | |
| --- | --- |
| `KdNode.Indexed3d()` |
| `KdNode.Indexed3d(double[] newVal, int newIndex)` |
| `KdNode.Indexed3d(KdNode.Indexed3d id)` |


| **Method Summary** | |
| --- | --- |
| `void` | `set(double[] newVal, int newIndex)` |
| `void` | `set(KdNode.Indexed3d id)` |

| **Methods inherited from class java.lang.Object** |
| --- |
| `clone, equals, finalize, getClass, hashCode, notify, notifyAll, toString, wait, wait, wait` |

| **Field Detail** |
| --- |

### val

```
double[] val
```

---


### originalIndex

```
int originalIndex
```


| **Constructor Detail** |
| --- |

### KdNode.Indexed3d

```
public KdNode.Indexed3d()
```

---


### KdNode.Indexed3d

```
public KdNode.Indexed3d(double[] newVal,
                        int newIndex)
```

---


### KdNode.Indexed3d

```
public KdNode.Indexed3d(KdNode.Indexed3d id)
```


| **Method Detail** |
| --- |

### set

```
public void set(KdNode.Indexed3d id)
```

---


### set

```
public void set(double[] newVal,
                int newIndex)
```


---


|  |  |  |  |  |  |  |  |  |  |  |
| --- | --- | --- | --- | --- | --- | --- | --- | --- | --- | --- |
| |  |  |  |  |  |  |  |  | | --- | --- | --- | --- | --- | --- | --- | --- | | **Overview** | **Package** | **Class** | **Use** | **Tree** | **Deprecated** | **Index** | **Help** | | |  |
| **PREV CLASS**   **NEXT CLASS** | **FRAMES**    **NO FRAMES**     **All Classes** |
| SUMMARY: NESTED | FIELD | CONSTR | METHOD | DETAIL: FIELD | CONSTR | METHOD |


---
