# Supplementary material for: BSim: An Agent-Based Tool for Modeling Bacterial Populations in Systems and Synthetic Biology
Source: PLoS One. 2012 Aug 24;7(8):e42790. doi: 10.1371/journal.pone.0042790 (PMC3427305; doi:10.1371/journal.pone.0042790)
Supplement: Software S1 — Snapshot of the BSim software from 18th July 2012. For the latest version see: http://bsim-bccs.sf.net. The BSim software requires Java version 1.6 or higher. (ZIP) [file pone.0042790.s014.zip › BSimSoftware/docs/javadoc/bsim/geometry/KdNode.TestMesh.html]

KdNode.TestMesh


---


|  |  |  |  |  |  |  |  |  |  |  |
| --- | --- | --- | --- | --- | --- | --- | --- | --- | --- | --- |
| |  |  |  |  |  |  |  |  | | --- | --- | --- | --- | --- | --- | --- | --- | | **Overview** | **Package** | **Class** | **Use** | **Tree** | **Deprecated** | **Index** | **Help** | | |  |
| **PREV CLASS**   NEXT CLASS | **FRAMES**    **NO FRAMES**     **All Classes** |
| SUMMARY: NESTED | FIELD | CONSTR | METHOD | DETAIL: FIELD | CONSTR | METHOD |


---


## bsim.geometry Class KdNode.TestMesh

```
java.lang.Object
  bsim.geometry.BSimMesh
      bsim.geometry.KdNode.TestMesh
```

**Enclosing class:**: KdNode

---

``` class KdNode.TestMesh extends BSimMesh ```

---

| **Field Summary** | |
| --- | --- |

| **Fields inherited from class bsim.geometry.BSimMesh** |
| --- |
| `faces, vertices` |


| **Constructor Summary** | |
| --- | --- |
| `KdNode.TestMesh()` |


| **Method Summary** | |
| --- | --- |
| `protected  void` | `createMesh()`             Abstract method in which the vertices and faces of the mesh should be defined. |

| **Methods inherited from class bsim.geometry.BSimMesh** |
| --- |
| `addTriangle, addTriangle, addVertex, addVertex, averagedCentreOfMesh, calcVertexFaces, cleanUp, computeNormal, computeNormals, flipNormals, flipNormals, getFace, getFaces, getTCentre, getVertCoords, getVertCoordsOfTri, getVertex, getVertices, printStats, scale, scale, translate, translateAbsolute` |

| **Methods inherited from class java.lang.Object** |
| --- |
| `clone, equals, finalize, getClass, hashCode, notify, notifyAll, toString, wait, wait, wait` |

| **Constructor Detail** |
| --- |

### KdNode.TestMesh

```
public KdNode.TestMesh()
```


| **Method Detail** |
| --- |

### createMesh

```
protected void createMesh()
```

:   **Description copied from class: `BSimMesh`**
:   Abstract method in which the vertices and faces of the mesh should be defined.

    :   **Specified by:**: `createMesh` in class `BSimMesh`


---


|  |  |  |  |  |  |  |  |  |  |  |
| --- | --- | --- | --- | --- | --- | --- | --- | --- | --- | --- |
| |  |  |  |  |  |  |  |  | | --- | --- | --- | --- | --- | --- | --- | --- | | **Overview** | **Package** | **Class** | **Use** | **Tree** | **Deprecated** | **Index** | **Help** | | |  |
| **PREV CLASS**   NEXT CLASS | **FRAMES**    **NO FRAMES**     **All Classes** |
| SUMMARY: NESTED | FIELD | CONSTR | METHOD | DETAIL: FIELD | CONSTR | METHOD |


---
