# Supplementary material for: BSim: An Agent-Based Tool for Modeling Bacterial Populations in Systems and Synthetic Biology
Source: PLoS One. 2012 Aug 24;7(8):e42790. doi: 10.1371/journal.pone.0042790 (PMC3427305; doi:10.1371/journal.pone.0042790)
Supplement: Software S1 — Snapshot of the BSim software from 18th July 2012. For the latest version see: http://bsim-bccs.sf.net. The BSim software requires Java version 1.6 or higher. (ZIP) [file pone.0042790.s014.zip › BSimSoftware/docs/javadoc/bsim/geometry/package-summary.html]

bsim.geometry


---


|  |  |  |  |  |  |  |  |  |  |  |
| --- | --- | --- | --- | --- | --- | --- | --- | --- | --- | --- |
| |  |  |  |  |  |  |  |  | | --- | --- | --- | --- | --- | --- | --- | --- | | **Overview** | **Package** | Class | **Use** | **Tree** | **Deprecated** | **Index** | **Help** | | |  |
| **PREV PACKAGE**   **NEXT PACKAGE** | **FRAMES**    **NO FRAMES**     **All Classes** |


---

## Package bsim.geometry

| **Class Summary** | |
| --- | --- |
| **BSimCollision** | Collision related methods. |
| **BSimMesh** | Abstract 3-D mesh surface class. |
| **BSimMeshUtils** | Utility functions for meshes. |
| **BSimOBJMesh** | Wavefront OBJ importer. |
| **BSimSphereMesh** | Sphere mesh, uses face-vertex representation. |
| **BSimTriangle** | Triangular face of a 3-D mesh surface. |
| **BSimVertex** | Mesh vertex. |
| **KdNode** | See, for example, http://en.wikipedia.org/wiki/Kd-tree Effectively an axis-aligned BSP tree which alternates the splitting plane axis at each level of branching. |

---


|  |  |  |  |  |  |  |  |  |  |  |
| --- | --- | --- | --- | --- | --- | --- | --- | --- | --- | --- |
| |  |  |  |  |  |  |  |  | | --- | --- | --- | --- | --- | --- | --- | --- | | **Overview** | **Package** | Class | **Use** | **Tree** | **Deprecated** | **Index** | **Help** | | |  |
| **PREV PACKAGE**   **NEXT PACKAGE** | **FRAMES**    **NO FRAMES**     **All Classes** |


---
