# Supplementary material for: BSim: An Agent-Based Tool for Modeling Bacterial Populations in Systems and Synthetic Biology
Source: PLoS One. 2012 Aug 24;7(8):e42790. doi: 10.1371/journal.pone.0042790 (PMC3427305; doi:10.1371/journal.pone.0042790)
Supplement: Software S1 — Snapshot of the BSim software from 18th July 2012. For the latest version see: http://bsim-bccs.sf.net. The BSim software requires Java version 1.6 or higher. (ZIP) [file pone.0042790.s014.zip › BSimSoftware/docs/javadoc/bsim/OctreeNode.html]

OctreeNode


---


|  |  |  |  |  |  |  |  |  |  |  |
| --- | --- | --- | --- | --- | --- | --- | --- | --- | --- | --- |
| |  |  |  |  |  |  |  |  | | --- | --- | --- | --- | --- | --- | --- | --- | | **Overview** | **Package** | **Class** | **Use** | **Tree** | **Deprecated** | **Index** | **Help** | | |  |
| **PREV CLASS**   NEXT CLASS | **FRAMES**    **NO FRAMES**     **All Classes** |
| SUMMARY: NESTED | FIELD | CONSTR | METHOD | DETAIL: FIELD | CONSTR | METHOD |


---


## bsim Class OctreeNode

```
java.lang.Object
  bsim.OctreeNode
```

---

``` public class OctreeNode extends java.lang.Object ```

---

| **Field Summary** | |
| --- | --- |
| `protected  javax.vecmath.Vector3d` | `centre`             Location of center of node in space. |
| `protected  int` | `depth`             Depth of a node in the Octree structure, root has depth 0. |
| `protected  double` | `length`             Dimension of the node. |
| `protected  java.awt.Color` | `nodeColor`             Color of the node, used when rendering it. |
| `protected  OctreeNode` | `parent`             This is the root of the Octree data structure. |
| `boolean` | `processed`             Holds a status indicating whether or not the node has been processed in diffusion algorithm. |
| `double` | `quantity`             Number of molecules in the chemical field box. |
| `protected  OctreeNode[]` | `subNodes`             subNodes of Octree, these can have subnodes of their own. |
| `protected  double` | `volume`             Volume of node (simply length^3). |


| **Constructor Summary** | |
| --- | --- |
| `OctreeNode()`             Constructor for a basic OctreeNode (default settings). |
| `OctreeNode(javax.vecmath.Vector3d Centre, double Length)`             Root Constructor used to make first Octree node, the parent node. |


| **Method Summary** | |
| --- | --- |
| `void` | `colorFromCentre(OctreeNode t)`             Sets the nodeColor value as a function of the position of octree, useful for troubleshooting. |
| `void` | `colorFromConc()`             Sets nodeCololr value as a function of amount of chemical in box. |
| `void` | `decay(OctreeNode t, double decayRate, double Dt)`             Decays the chemical field in an octree Node,visits each node in the tree structure using a post-order traverse. |
| `void` | `diffuse(OctreeNode t, double diffusivity, double Dt, int depth)`             Diffuses chemicals through whole the octree structure, using Fick's law to determine how much of the chemical gets pushed into neighboring nodes over each time iteration. |
| `javax.vecmath.Vector3d` | `getCentre()`             Return the position of the center of the node. |
| `int` | `getDepth()`             Return the depth. |
| `double` | `getLength()`             Return the length of the node. |
| `java.awt.Color` | `getnodeColor()`             Return the node colour. |
| `OctreeNode` | `getsubNode(int i)`             Return the nodes subNode, i is index of subNode. |
| `static void` | `inOrderfull(OctreeNode t)`             In-Order full traverse, traverses from the deepest subnode, to the root and then back down to other deep nodes. |
| `static boolean` | `intersectVectorTriangle(javax.vecmath.Vector3d startPos, javax.vecmath.Vector3d endPos, BSimTriangle tri)` |
| `OctreeNode` | `NodeFinder(OctreeNode t, int depth)`             Gets a subnode of given index from lowest depth |
| `static void` | `postOrderfull(OctreeNode t)`             Post-Order traverse with visit function. |
| `static void` | `preOrderfull(OctreeNode t)`             Pre-Order full traverse - traverses from the root, a direction to the deepest subnode, back to the node, and then down into other roots. |
| `void` | `setKids()`             Initializes all the subNodes with appropriate neighbors/centers and lengths. |
| `void` | `setNodestoMesh(BSimMesh theMesh, OctreeNode t)`             Takes an octree node and divides its subnodes in such a way that the nodes conform to a coarse grained version of the shape of the mesh. |
| `void` | `visit(OctreeNode t)`             The visit method simply prints the location and depth of a node, useful for troubleshooting. |

| **Methods inherited from class java.lang.Object** |
| --- |
| `clone, equals, finalize, getClass, hashCode, notify, notifyAll, toString, wait, wait, wait` |

| **Field Detail** |
| --- |

### parent

```
protected OctreeNode parent
```

:   This is the root of the Octree data structure.

---


### nodeColor

```
protected java.awt.Color nodeColor
```

:   Color of the node, used when rendering it.

---


### centre

```
protected javax.vecmath.Vector3d centre
```

:   Location of center of node in space.

---


### length

```
protected double length
```

:   Dimension of the node.

---


### volume

```
protected double volume
```

:   Volume of node (simply length^3).

---


### depth

```
protected int depth
```

:   Depth of a node in the Octree structure, root has depth 0.

---


### subNodes

```
protected OctreeNode[] subNodes
```

:   subNodes of Octree, these can have subnodes of their own.

---


### processed

```
public boolean processed
```

:   Holds a status indicating whether or not the node has been processed in diffusion algorithm.

---


### quantity

```
public double quantity
```

:   Number of molecules in the chemical field box.


| **Constructor Detail** |
| --- |

### OctreeNode

```
public OctreeNode()
```

:   Constructor for a basic OctreeNode (default settings).

---


### OctreeNode

```
public OctreeNode(javax.vecmath.Vector3d Centre,
                  double Length)
```

:   Root Constructor used to make first Octree node, the parent node. It is further
    subdivided using the setKids() function.


| **Method Detail** |
| --- |

### getDepth

```
public int getDepth()
```

:   Return the depth.

---


### getCentre

```
public javax.vecmath.Vector3d getCentre()
```

:   Return the position of the center of the node.

---


### getLength

```
public double getLength()
```

:   Return the length of the node.

---


### getsubNode

```
public OctreeNode getsubNode(int i)
```

:   Return the nodes subNode, i is index of subNode.

---


### getnodeColor

```
public java.awt.Color getnodeColor()
```

:   Return the node colour.

---


### setKids

```
public void setKids()
```

:   Initializes all the subNodes with appropriate neighbors/centers and lengths.
    All other properties are inherited from the parent node.

---


### setNodestoMesh

```
public void setNodestoMesh(BSimMesh theMesh,
                           OctreeNode t)
```

:   Takes an octree node and divides its subnodes in such a way that the
    nodes conform to a coarse grained version of the shape of the mesh.
    A similar method exists for OctreeChemicalFields, this method requires only an octree
    node.

---


### preOrderfull

```
public static void preOrderfull(OctreeNode t)
```

:   Pre-Order full traverse - traverses from the root, a direction to the
    deepest subnode, back to the node, and then down into other roots.
    Not recommended

---


### inOrderfull

```
public static void inOrderfull(OctreeNode t)
```

:   In-Order full traverse, traverses from the deepest subnode, to the root
    and then back down to other deep nodes.
    Not recommended.

---


### postOrderfull

```
public static void postOrderfull(OctreeNode t)
```

:   Post-Order traverse with visit function. This is the most logical
    traverse and visits octrees in 'left to right, bottom to top' sense
    this is used to traversing octree structures.

---


### NodeFinder

```
public OctreeNode NodeFinder(OctreeNode t,
                             int depth)
```

:   Gets a subnode of given index from lowest depth

---


### visit

```
public void visit(OctreeNode t)
```

:   The visit method simply prints the location and depth of
    a node, useful for troubleshooting.

---


### colorFromCentre

```
public void colorFromCentre(OctreeNode t)
```

:   Sets the nodeColor value as a function of the position of octree,
    useful for troubleshooting.

---


### colorFromConc

```
public void colorFromConc()
```

:   Sets nodeCololr value as a function of amount of chemical in box.

---


### diffuse

```
public void diffuse(OctreeNode t,
                    double diffusivity,
                    double Dt,
                    int depth)
```

:   Diffuses chemicals through whole the octree structure, using Fick's law to determine
    how much of the chemical gets pushed into neighboring nodes over each time iteration.

    :   **Parameters:**: `diffusivity` - The diffusivity in (microns)^2/s.: `Dt` - Time steps in seconds.: `depth` - Maximum depth to go to in diffusion.

---


### decay

```
public void decay(OctreeNode t,
                  double decayRate,
                  double Dt)
```

:   Decays the chemical field in an octree Node,visits each node in the tree structure
    using a post-order traverse.

    :   **Parameters:**: `t` - Octree Node: `decayRate` - Decay rate in seconds^-1.: `Dt` - Timestep size to use in seconds.

---


### intersectVectorTriangle

```
public static boolean intersectVectorTriangle(javax.vecmath.Vector3d startPos,
                                              javax.vecmath.Vector3d endPos,
                                              BSimTriangle tri)
```


---


|  |  |  |  |  |  |  |  |  |  |  |
| --- | --- | --- | --- | --- | --- | --- | --- | --- | --- | --- |
| |  |  |  |  |  |  |  |  | | --- | --- | --- | --- | --- | --- | --- | --- | | **Overview** | **Package** | **Class** | **Use** | **Tree** | **Deprecated** | **Index** | **Help** | | |  |
| **PREV CLASS**   NEXT CLASS | **FRAMES**    **NO FRAMES**     **All Classes** |
| SUMMARY: NESTED | FIELD | CONSTR | METHOD | DETAIL: FIELD | CONSTR | METHOD |


---
