# Supplementary material for: BSim: An Agent-Based Tool for Modeling Bacterial Populations in Systems and Synthetic Biology
Source: PLoS One. 2012 Aug 24;7(8):e42790. doi: 10.1371/journal.pone.0042790 (PMC3427305; doi:10.1371/journal.pone.0042790)
Supplement: Software S1 — Snapshot of the BSim software from 18th July 2012. For the latest version see: http://bsim-bccs.sf.net. The BSim software requires Java version 1.6 or higher. (ZIP) [file pone.0042790.s014.zip › BSimSoftware/docs/javadoc/bsim/ode/BSimOdeSolver.html]

BSimOdeSolver


---


|  |  |  |  |  |  |  |  |  |  |  |
| --- | --- | --- | --- | --- | --- | --- | --- | --- | --- | --- |
| |  |  |  |  |  |  |  |  | | --- | --- | --- | --- | --- | --- | --- | --- | | **Overview** | **Package** | **Class** | **Use** | **Tree** | **Deprecated** | **Index** | **Help** | | |  |
| PREV CLASS   **NEXT CLASS** | **FRAMES**    **NO FRAMES**     **All Classes** |
| SUMMARY: NESTED | FIELD | CONSTR | METHOD | DETAIL: FIELD | CONSTR | METHOD |


---


## bsim.ode Class BSimOdeSolver

```
java.lang.Object
  bsim.ode.BSimOdeSolver
```

---

``` public class BSimOdeSolver extends java.lang.Object ```

Solver routines for numerical simulation of ODEs (Fixed time-step):

- Euler's method
- second order Runge-Kutta
- fourth order Runge-Kutta

Each method will estimate the change of the dependent variable based
on the previous value of the dependent (y) and independent (x) variables,
and return the new value of the dependent variable.
The methods are in order of increasing accuracy for a given time-step;
Euler's method is the most basic, but the fastest as a result of having to
perform relatively few calculations, while the Runge-Kutta methods use an
intermediate trial step at the midpoint of an interval to cancel lower order
error terms. 
If the time step is too large an Euler solution will quickly diverge from
the true solution, therefore it is recommended to use a higher order solution
if the time-step cannot reasonably be decreased.

---

| **Constructor Summary** | |
| --- | --- |
| `BSimOdeSolver()` |


| **Method Summary** | |
| --- | --- |
| `static double[]` | `euler(BSimOdeSystem odes, double t, double[] y, double h)`             Numerically solve an ODE system with Euler's method. |
| `static double[]` | `rungeKutta23(BSimOdeSystem odes, double t, double[] y, double h)`             Numerically solve an ODE system with 2nd order Runge-Kutta method. |
| `static double[]` | `rungeKutta45(BSimOdeSystem odes, double t, double[] y, double h)`             Numerically solve an ODE system with 4th order Runge-Kutta method |

| **Methods inherited from class java.lang.Object** |
| --- |
| `clone, equals, finalize, getClass, hashCode, notify, notifyAll, toString, wait, wait, wait` |

| **Constructor Detail** |
| --- |

### BSimOdeSolver

```
public BSimOdeSolver()
```


| **Method Detail** |
| --- |

### euler

```
public static double[] euler(BSimOdeSystem odes,
                             double t,
                             double[] y,
                             double h)
```

:   Numerically solve an ODE system with Euler's method.

    :   **Parameters:**: `odes` - The `BSimOdeSystem` to solve.: `t` - Independent variable.: `y` - Vector of dependent variables.: `h` - Time step for ode solution. **Returns:**: Vector of dependent variables at next time step.

---


### rungeKutta23

```
public static double[] rungeKutta23(BSimOdeSystem odes,
                                    double t,
                                    double[] y,
                                    double h)
```

:   Numerically solve an ODE system with 2nd order Runge-Kutta method.

    :   **Parameters:**: `odes` - The `BSimOdeSystem` to solve.: `t` - Independent variable.: `y` - Vector of dependent variables.: `h` - Time step for ode solution. **Returns:**: Vector of dependent variables at next time step.

---


### rungeKutta45

```
public static double[] rungeKutta45(BSimOdeSystem odes,
                                    double t,
                                    double[] y,
                                    double h)
```

:   Numerically solve an ODE system with 4th order Runge-Kutta method

    :   **Parameters:**: `odes` - The `BSimOdeSystem` to solve.: `t` - Independent variable.: `y` - Vector of dependent variables.: `h` - Time step for ode solution. **Returns:**: Vector of dependent variables at next time step.


---


|  |  |  |  |  |  |  |  |  |  |  |
| --- | --- | --- | --- | --- | --- | --- | --- | --- | --- | --- |
| |  |  |  |  |  |  |  |  | | --- | --- | --- | --- | --- | --- | --- | --- | | **Overview** | **Package** | **Class** | **Use** | **Tree** | **Deprecated** | **Index** | **Help** | | |  |
| PREV CLASS   **NEXT CLASS** | **FRAMES**    **NO FRAMES**     **All Classes** |
| SUMMARY: NESTED | FIELD | CONSTR | METHOD | DETAIL: FIELD | CONSTR | METHOD |


---
