# Supplementary material for: BSim: An Agent-Based Tool for Modeling Bacterial Populations in Systems and Synthetic Biology
Source: PLoS One. 2012 Aug 24;7(8):e42790. doi: 10.1371/journal.pone.0042790 (PMC3427305; doi:10.1371/journal.pone.0042790)
Supplement: Software S1 — Snapshot of the BSim software from 18th July 2012. For the latest version see: http://bsim-bccs.sf.net. The BSim software requires Java version 1.6 or higher. (ZIP) [file pone.0042790.s014.zip › BSimSoftware/docs/javadoc/bsim/ode/BSimOdeSystem.html]

BSimOdeSystem


---


|  |  |  |  |  |  |  |  |  |  |  |
| --- | --- | --- | --- | --- | --- | --- | --- | --- | --- | --- |
| |  |  |  |  |  |  |  |  | | --- | --- | --- | --- | --- | --- | --- | --- | | **Overview** | **Package** | **Class** | **Use** | **Tree** | **Deprecated** | **Index** | **Help** | | |  |
| **PREV CLASS**   NEXT CLASS | **FRAMES**    **NO FRAMES**     **All Classes** |
| SUMMARY: NESTED | FIELD | CONSTR | METHOD | DETAIL: FIELD | CONSTR | METHOD |


---


## bsim.ode Interface BSimOdeSystem

---

``` public interface BSimOdeSystem ```

Interface used for defining a system of ODEs.
Defines the ODEs, the number of equations and the initial conditions.

---

| **Method Summary** | |
| --- | --- |
| `double[]` | `derivativeSystem(double x, double[] y)`             Define a system of derivatives - dy[0] = ..., dy[1] = ..., etc then return dy[] |
| `double[]` | `getICs()`             Get the initial conditions: y1(0), y2(0), etc.. |
| `int` | `getNumEq()`             Get the number of equations in the system (Corresponding to the number in derivativeSystem) |

| **Method Detail** |
| --- |

### derivativeSystem

```
double[] derivativeSystem(double x,
                          double[] y)
```

:   Define a system of derivatives -
    dy[0] = ..., dy[1] = ..., etc
    then return dy[]

---


### getNumEq

```
int getNumEq()
```

:   Get the number of equations in the system
    (Corresponding to the number in derivativeSystem)

---


### getICs

```
double[] getICs()
```

:   Get the initial conditions: y1(0), y2(0), etc..


---


|  |  |  |  |  |  |  |  |  |  |  |
| --- | --- | --- | --- | --- | --- | --- | --- | --- | --- | --- |
| |  |  |  |  |  |  |  |  | | --- | --- | --- | --- | --- | --- | --- | --- | | **Overview** | **Package** | **Class** | **Use** | **Tree** | **Deprecated** | **Index** | **Help** | | |  |
| **PREV CLASS**   NEXT CLASS | **FRAMES**    **NO FRAMES**     **All Classes** |
| SUMMARY: NESTED | FIELD | CONSTR | METHOD | DETAIL: FIELD | CONSTR | METHOD |


---
