# Supplementary material for: BSim: An Agent-Based Tool for Modeling Bacterial Populations in Systems and Synthetic Biology
Source: PLoS One. 2012 Aug 24;7(8):e42790. doi: 10.1371/journal.pone.0042790 (PMC3427305; doi:10.1371/journal.pone.0042790)
Supplement: Software S1 — Snapshot of the BSim software from 18th July 2012. For the latest version see: http://bsim-bccs.sf.net. The BSim software requires Java version 1.6 or higher. (ZIP) [file pone.0042790.s014.zip › BSimSoftware/docs/javadoc/bsim/ode/class-use/BSimOdeSystem.html]

Uses of Interface bsim.ode.BSimOdeSystem


---


|  |  |  |  |  |  |  |  |  |  |  |
| --- | --- | --- | --- | --- | --- | --- | --- | --- | --- | --- |
| |  |  |  |  |  |  |  |  | | --- | --- | --- | --- | --- | --- | --- | --- | | **Overview** | **Package** | **Class** | **Use** | **Tree** | **Deprecated** | **Index** | **Help** | | |  |
| PREV   NEXT | **FRAMES**    **NO FRAMES**     **All Classes** |


---


## **Uses of Interface bsim.ode.BSimOdeSystem**

| Packages that use BSimOdeSystem | |
| --- | --- |
| **bsim.ode** |  |

| Uses of BSimOdeSystem in bsim.ode | |
| --- | --- |

| Methods in bsim.ode with parameters of type BSimOdeSystem | |
| --- | --- |
| `static double[]` | `BSimOdeSolver.euler(BSimOdeSystem odes, double t, double[] y, double h)`             Numerically solve an ODE system with Euler's method. |
| `static double[]` | `BSimOdeSolver.rungeKutta23(BSimOdeSystem odes, double t, double[] y, double h)`             Numerically solve an ODE system with 2nd order Runge-Kutta method. |
| `static double[]` | `BSimOdeSolver.rungeKutta45(BSimOdeSystem odes, double t, double[] y, double h)`             Numerically solve an ODE system with 4th order Runge-Kutta method |

---


|  |  |  |  |  |  |  |  |  |  |  |
| --- | --- | --- | --- | --- | --- | --- | --- | --- | --- | --- |
| |  |  |  |  |  |  |  |  | | --- | --- | --- | --- | --- | --- | --- | --- | | **Overview** | **Package** | **Class** | **Use** | **Tree** | **Deprecated** | **Index** | **Help** | | |  |
| PREV   NEXT | **FRAMES**    **NO FRAMES**     **All Classes** |


---
