# Supplementary material for: BSim: An Agent-Based Tool for Modeling Bacterial Populations in Systems and Synthetic Biology
Source: PLoS One. 2012 Aug 24;7(8):e42790. doi: 10.1371/journal.pone.0042790 (PMC3427305; doi:10.1371/journal.pone.0042790)
Supplement: Software S1 — Snapshot of the BSim software from 18th July 2012. For the latest version see: http://bsim-bccs.sf.net. The BSim software requires Java version 1.6 or higher. (ZIP) [file pone.0042790.s014.zip › BSimSoftware/docs/javadoc/bsim/ode/package-summary.html]

bsim.ode


---


|  |  |  |  |  |  |  |  |  |  |  |
| --- | --- | --- | --- | --- | --- | --- | --- | --- | --- | --- |
| |  |  |  |  |  |  |  |  | | --- | --- | --- | --- | --- | --- | --- | --- | | **Overview** | **Package** | Class | **Use** | **Tree** | **Deprecated** | **Index** | **Help** | | |  |
| **PREV PACKAGE**   **NEXT PACKAGE** | **FRAMES**    **NO FRAMES**     **All Classes** |


---

## Package bsim.ode

| **Interface Summary** | |
| --- | --- |
| **BSimOdeSystem** | Interface used for defining a system of ODEs. |

| **Class Summary** | |
| --- | --- |
| **BSimOdeSolver** | Solver routines for numerical simulation of ODEs (Fixed time-step): Euler's method second order Runge-Kutta fourth order Runge-Kutta Each method will estimate the change of the dependent variable based on the previous value of the dependent (y) and independent (x) variables, and return the new value of the dependent variable. The methods are in order of increasing accuracy for a given time-step; Euler's method is the most basic, but the fastest as a result of having to perform relatively few calculations, while the Runge-Kutta methods use an intermediate trial step at the midpoint of an interval to cancel lower order error terms. |

---


|  |  |  |  |  |  |  |  |  |  |  |
| --- | --- | --- | --- | --- | --- | --- | --- | --- | --- | --- |
| |  |  |  |  |  |  |  |  | | --- | --- | --- | --- | --- | --- | --- | --- | | **Overview** | **Package** | Class | **Use** | **Tree** | **Deprecated** | **Index** | **Help** | | |  |
| **PREV PACKAGE**   **NEXT PACKAGE** | **FRAMES**    **NO FRAMES**     **All Classes** |


---
