# Supplementary material for: BSim: An Agent-Based Tool for Modeling Bacterial Populations in Systems and Synthetic Biology
Source: PLoS One. 2012 Aug 24;7(8):e42790. doi: 10.1371/journal.pone.0042790 (PMC3427305; doi:10.1371/journal.pone.0042790)
Supplement: Software S1 — Snapshot of the BSim software from 18th July 2012. For the latest version see: http://bsim-bccs.sf.net. The BSim software requires Java version 1.6 or higher. (ZIP) [file pone.0042790.s014.zip › BSimSoftware/docs/javadoc/bsim/ode/package-tree.html]

bsim.ode Class Hierarchy


---


|  |  |  |  |  |  |  |  |  |  |  |
| --- | --- | --- | --- | --- | --- | --- | --- | --- | --- | --- |
| |  |  |  |  |  |  |  |  | | --- | --- | --- | --- | --- | --- | --- | --- | | **Overview** | **Package** | Class | Use | **Tree** | **Deprecated** | **Index** | **Help** | | |  |
| **PREV**   **NEXT** | **FRAMES**    **NO FRAMES**     **All Classes** |


---


## Hierarchy For Package bsim.ode

**Package Hierarchies:**: All Packages

---

## Class Hierarchy

- java.lang.Object
  - bsim.ode.**BSimOdeSolver**

## Interface Hierarchy

- bsim.ode.**BSimOdeSystem**

---


|  |  |  |  |  |  |  |  |  |  |  |
| --- | --- | --- | --- | --- | --- | --- | --- | --- | --- | --- |
| |  |  |  |  |  |  |  |  | | --- | --- | --- | --- | --- | --- | --- | --- | | **Overview** | **Package** | Class | Use | **Tree** | **Deprecated** | **Index** | **Help** | | |  |
| **PREV**   **NEXT** | **FRAMES**    **NO FRAMES**     **All Classes** |


---
