# Supplementary material for: BSim: An Agent-Based Tool for Modeling Bacterial Populations in Systems and Synthetic Biology
Source: PLoS One. 2012 Aug 24;7(8):e42790. doi: 10.1371/journal.pone.0042790 (PMC3427305; doi:10.1371/journal.pone.0042790)
Supplement: Software S1 — Snapshot of the BSim software from 18th July 2012. For the latest version see: http://bsim-bccs.sf.net. The BSim software requires Java version 1.6 or higher. (ZIP) [file pone.0042790.s014.zip › BSimSoftware/docs/javadoc/bsim/package-use.html]

Uses of Package bsim


---


|  |  |  |  |  |  |  |  |  |  |  |
| --- | --- | --- | --- | --- | --- | --- | --- | --- | --- | --- |
| |  |  |  |  |  |  |  |  | | --- | --- | --- | --- | --- | --- | --- | --- | | **Overview** | **Package** | Class | **Use** | **Tree** | **Deprecated** | **Index** | **Help** | | |  |
| PREV   NEXT | **FRAMES**    **NO FRAMES**     **All Classes** |


---


## **Uses of Package bsim**

| Packages that use bsim | |
| --- | --- |
| **bsim** |  |
| **bsim.draw** |  |
| **bsim.export** |  |
| **bsim.geometry** |  |
| **bsim.particle** |  |

| Classes in bsim used by bsim | |
| --- | --- |
| ****BSim****             Main simulation class. |
| ****BSimOctreeField****             Octree chemical field (non-uniform division of space). |
| ****BSimThreadedTickerWorker****             Multi-threaded ticker worker. |
| ****BSimTicker****             Standard ticker. |

| Classes in bsim used by bsim.draw | |
| --- | --- |
| ****BSim****             Main simulation class. |
| ****BSimChemicalField****             Standard chemical field (uniform division of space) . |
| ****BSimOctreeField****             Octree chemical field (non-uniform division of space). |

| Classes in bsim used by bsim.export | |
| --- | --- |
| ****BSim****             Main simulation class. |

| Classes in bsim used by bsim.geometry | |
| --- | --- |
| ****BSimOctreeField****             Octree chemical field (non-uniform division of space). |

| Classes in bsim used by bsim.particle | |
| --- | --- |
| ****BSim****             Main simulation class. |
| ****BSimChemicalField****             Standard chemical field (uniform division of space) . |

---


|  |  |  |  |  |  |  |  |  |  |  |
| --- | --- | --- | --- | --- | --- | --- | --- | --- | --- | --- |
| |  |  |  |  |  |  |  |  | | --- | --- | --- | --- | --- | --- | --- | --- | | **Overview** | **Package** | Class | **Use** | **Tree** | **Deprecated** | **Index** | **Help** | | |  |
| PREV   NEXT | **FRAMES**    **NO FRAMES**     **All Classes** |


---
