# Supplementary material for: BSim: An Agent-Based Tool for Modeling Bacterial Populations in Systems and Synthetic Biology
Source: PLoS One. 2012 Aug 24;7(8):e42790. doi: 10.1371/journal.pone.0042790 (PMC3427305; doi:10.1371/journal.pone.0042790)
Supplement: Software S1 — Snapshot of the BSim software from 18th July 2012. For the latest version see: http://bsim-bccs.sf.net. The BSim software requires Java version 1.6 or higher. (ZIP) [file pone.0042790.s014.zip › BSimSoftware/docs/javadoc/bsim/particle/BSimBacterium.html]

BSimBacterium


---


|  |  |  |  |  |  |  |  |  |  |  |
| --- | --- | --- | --- | --- | --- | --- | --- | --- | --- | --- |
| |  |  |  |  |  |  |  |  | | --- | --- | --- | --- | --- | --- | --- | --- | | **Overview** | **Package** | **Class** | **Use** | **Tree** | **Deprecated** | **Index** | **Help** | | |  |
| PREV CLASS   **NEXT CLASS** | **FRAMES**    **NO FRAMES**     **All Classes** |
| SUMMARY: NESTED | FIELD | CONSTR | METHOD | DETAIL: FIELD | CONSTR | METHOD |


---


## bsim.particle Class BSimBacterium

```
java.lang.Object
  bsim.particle.BSimParticle
      bsim.particle.BSimBacterium
```

---

``` public class BSimBacterium extends BSimParticle ```

Class representing a bacterium whose run-tumble motion
is affected in a simple way by a single goal chemical.

---

| **Nested Class Summary** | |
| --- | --- |
| `static class` | `BSimBacterium.MotionState` |


| **Field Summary** | |
| --- | --- |
| `protected  java.util.Vector` | `childList`             The external list of children. |
| `protected  javax.vecmath.Vector3d` | `direction`             Direction that the cell exerts its flagellar force. |
| `protected  double` | `forceMagnitude`             Magnitude of the flagellar force produced by the cell whilst RUNNING. |
| `protected  BSimChemicalField` | `goal`             Bacteria tend to swim towards higher concentrations of this chemical field. |
| `protected  double` | `longTermMemoryDuration` |
| `protected  double` | `longTermMemoryLength`             sim.timesteps(longTermMemoryDuration) |
| `protected  double[]` | `memory`             Memory of previous concentrations of the goal field. |
| `protected  BSimBacterium.MotionState` | `motionState` |
| `protected  double` | `pEndRunElse`             Probability per per unit time of ending a run otherwise |
| `protected  double` | `pEndRunUp`             Probability per per unit time of ending a run when moving up a chemical gradient |
| `protected  double` | `pEndTumble`             Probability per per unit time of ending a tumble |
| `protected  double` | `pVesicle`             Probability per typical vesicle surface area growth of producing a vesicle |
| `protected  double` | `replicationRadius` |
| `protected  double` | `sensitivity`             Sensitivity to differences in sequential averages (molecules/(micron)^3). |
| `protected  double` | `shortTermMemoryDuration` |
| `protected  double` | `shortTermMemoryLength`             sim.timesteps(shortTermMemoryDuration) |
| `protected  double` | `surfaceAreaGrowthRate` |
| `protected  double` | `typicalVesicleSurfaceArea` |
| `protected  java.util.Vector` | `vesicleList`             The external list of vesicles. |
| `protected  double` | `vesicleRadius` |

| **Fields inherited from class bsim.particle.BSimParticle** |
| --- |
| `brownianForceMagnitude, force, position, radius, rng, sim` |


| **Constructor Summary** | |
| --- | --- |
| `BSimBacterium(BSim sim, javax.vecmath.Vector3d position)`             Creates a RUNNING bacterium at the specified position, facing in a random direction |


| **Method Summary** | |
| --- | --- |
| `void` | `action()`             Call in BSimTicker#tick() |
| `void` | `flagellarForce()`             Applies the flagellar force. |
| `javax.vecmath.Vector3d` | `getDirection()` |
| `double` | `getMemoryDuration()` |
| `BSimBacterium.MotionState` | `getMotionState()` |
| `void` | `grow()` |
| `boolean` | `movingUpGradient()`             p23, 'Strategies for Chemotaxis', Schnizter, Berg et al. |
| `double` | `pEndRun()`             Probability per per unit time of ending a run |
| `void` | `pEndRunElse(double d)` |
| `void` | `pEndRunUp(double d)` |
| `double` | `pEndTumble()`             Probability per per unit time of ending a tumble. |
| `void` | `pEndTumble(double d)` |
| `void` | `pVesicle(double d)` |
| `void` | `replicate()` |
| `void` | `rotationalDiffusion()`             Causes the cell to rotate such that Var(theta(dt)) = 4\*D\*dt. |
| `double` | `rotationalStokesCoefficient()` |
| `void` | `setChildList(java.util.Vector v)` |
| `void` | `setDirection(javax.vecmath.Vector3d v)`             Set the direction of the cell to the direction of the vector v. |
| `void` | `setForceMagnitude(double d)` |
| `void` | `setGoal(BSimChemicalField goal)`             Set this chemical field as the goal field. |
| `void` | `setMemoryDuration(double shortTermMemoryDuration, double longTermMemoryDuration)` |
| `void` | `setMotionState(BSimBacterium.MotionState s)` |
| `void` | `setRadius()`             Sets the radius so that the surface area of the bacterium is randomly distributed between surfaceArea(replicationRadius)/2 and surfaceArea(replicationRadius) |
| `protected  void` | `setReplicationRadius(double r)` |
| `void` | `setSurfaceAreaGrowthRate()` |
| `void` | `setSurfaceAreaGrowthRate(double d)` |
| `void` | `setVesicleList(java.util.Vector v)` |
| `double` | `tumbleAngle()`             Return a tumble angle in radians distributed according to Fig. |
| `double` | `vesicleRadius()` |
| `void` | `vesicleRadius(double d)` |
| `void` | `vesiculate()` |

| **Methods inherited from class bsim.particle.BSimParticle** |
| --- |
| `addForce, bounceAbove, bounceBelow, brownianForce, distance, getForce, getPosition, getRadius, getSurfaceArea, intersection, logReaction, outerDistance, reaction, setBrownianForceMagnitude, setRadius, setRadiusFromSurfaceArea, stokesCoefficient, surfaceArea, updatePosition, wrapAbove, wrapBelow, xAbove, xBelow, yAbove, yBelow, zAbove, zBelow` |

| **Methods inherited from class java.lang.Object** |
| --- |
| `clone, equals, finalize, getClass, hashCode, notify, notifyAll, toString, wait, wait, wait` |

| **Field Detail** |
| --- |

### motionState

```
protected BSimBacterium.MotionState motionState
```

---


### forceMagnitude

```
protected double forceMagnitude
```

:   Magnitude of the flagellar force produced by the cell whilst RUNNING.
    Calculated from Stokes law F = 6\*PI\*radius\*viscosity\*speed with a radius of 1 micron,
    a viscosity of 2.7e-3 Pa s and a speed of 20 microns/s (conditions of
    'Chemotaxis in Escherichia Coli', Berg et al.)

---


### direction

```
protected javax.vecmath.Vector3d direction
```

:   Direction that the cell exerts its flagellar force.

---


### goal

```
protected BSimChemicalField goal
```

:   Bacteria tend to swim towards higher concentrations of this chemical field.

---


### memory

```
protected double[] memory
```

:   Memory of previous concentrations of the goal field.

---


### shortTermMemoryDuration

```
protected double shortTermMemoryDuration
```

---


### longTermMemoryDuration

```
protected double longTermMemoryDuration
```

---


### shortTermMemoryLength

```
protected double shortTermMemoryLength
```

:   sim.timesteps(shortTermMemoryDuration)

---


### longTermMemoryLength

```
protected double longTermMemoryLength
```

:   sim.timesteps(longTermMemoryDuration)

---


### sensitivity

```
protected double sensitivity
```

:   Sensitivity to differences in sequential averages (molecules/(micron)^3).

---


### pEndRunUp

```
protected double pEndRunUp
```

:   Probability per per unit time of ending a run when moving up a chemical gradient

---


### pEndRunElse

```
protected double pEndRunElse
```

:   Probability per per unit time of ending a run otherwise

---


### pEndTumble

```
protected double pEndTumble
```

:   Probability per per unit time of ending a tumble

---


### surfaceAreaGrowthRate

```
protected double surfaceAreaGrowthRate
```

---


### replicationRadius

```
protected double replicationRadius
```

---


### childList

```
protected java.util.Vector childList
```

:   The external list of children. Required when bacteria reach the replicationRadius

---


### vesicleRadius

```
protected double vesicleRadius
```

---


### typicalVesicleSurfaceArea

```
protected double typicalVesicleSurfaceArea
```

---


### pVesicle

```
protected double pVesicle
```

:   Probability per typical vesicle surface area growth of producing a vesicle

---


### vesicleList

```
protected java.util.Vector vesicleList
```

:   The external list of vesicles. Required when bacteria vesiculate


| **Constructor Detail** |
| --- |

### BSimBacterium

```
public BSimBacterium(BSim sim,
                     javax.vecmath.Vector3d position)
```

:   Creates a RUNNING bacterium at the specified position, facing in a
    random direction


| **Method Detail** |
| --- |

### pEndRun

```
public double pEndRun()
```

:   Probability per per unit time of ending a run

---


### pEndTumble

```
public double pEndTumble()
```

:   Probability per per unit time of ending a tumble.

---


### pEndRunUp

```
public void pEndRunUp(double d)
```

---


### pEndRunElse

```
public void pEndRunElse(double d)
```

---


### pEndTumble

```
public void pEndTumble(double d)
```

---


### setMotionState

```
public void setMotionState(BSimBacterium.MotionState s)
```

---


### setForceMagnitude

```
public void setForceMagnitude(double d)
```

---


### setDirection

```
public void setDirection(javax.vecmath.Vector3d v)
```

:   Set the direction of the cell to the direction of the vector v.

---


### setGoal

```
public void setGoal(BSimChemicalField goal)
```

:   Set this chemical field as the goal field.

---


### setMemoryDuration

```
public void setMemoryDuration(double shortTermMemoryDuration,
                              double longTermMemoryDuration)
```

---


### getDirection

```
public javax.vecmath.Vector3d getDirection()
```

---


### getMotionState

```
public BSimBacterium.MotionState getMotionState()
```

---


### getMemoryDuration

```
public double getMemoryDuration()
```

---


### flagellarForce

```
public void flagellarForce()
```

:   Applies the flagellar force.

---


### rotationalDiffusion

```
public void rotationalDiffusion()
```

:   Causes the cell to rotate such that Var(theta(dt)) = 4\*D\*dt.

---


### rotationalStokesCoefficient

```
public double rotationalStokesCoefficient()
```

---


### tumbleAngle

```
public double tumbleAngle()
```

:   Return a tumble angle in radians distributed according to Fig. 3, 'Chemotaxis
    in Escherichia Coli', Berg et al. (claim from 'AgentCell: a digital single-cell
    assay for bacterial chemotaxis', Emonet et al.).

---


### movingUpGradient

```
public boolean movingUpGradient()
```

:   p23, 'Strategies for Chemotaxis', Schnizter, Berg et al.
    Compare two sequential averages.

---


### setSurfaceAreaGrowthRate

```
public void setSurfaceAreaGrowthRate()
```

---


### setSurfaceAreaGrowthRate

```
public void setSurfaceAreaGrowthRate(double d)
```

---


### setReplicationRadius

```
protected void setReplicationRadius(double r)
```

---


### setChildList

```
public void setChildList(java.util.Vector v)
```

---


### setRadius

```
public void setRadius()
```

:   Sets the radius so that the surface area of the bacterium is randomly distributed between surfaceArea(replicationRadius)/2 and surfaceArea(replicationRadius)

---


### grow

```
public void grow()
```

---


### replicate

```
public void replicate()
```

---


### vesicleRadius

```
public double vesicleRadius()
```

---


### vesicleRadius

```
public void vesicleRadius(double d)
```

---


### pVesicle

```
public void pVesicle(double d)
```

---


### setVesicleList

```
public void setVesicleList(java.util.Vector v)
```

---


### vesiculate

```
public void vesiculate()
```

---


### action

```
public void action()
```

:   **Description copied from class: `BSimParticle`**
:   Call in BSimTicker#tick()

    :   **Overrides:**: `action` in class `BSimParticle`


---


|  |  |  |  |  |  |  |  |  |  |  |
| --- | --- | --- | --- | --- | --- | --- | --- | --- | --- | --- |
| |  |  |  |  |  |  |  |  | | --- | --- | --- | --- | --- | --- | --- | --- | | **Overview** | **Package** | **Class** | **Use** | **Tree** | **Deprecated** | **Index** | **Help** | | |  |
| PREV CLASS   **NEXT CLASS** | **FRAMES**    **NO FRAMES**     **All Classes** |
| SUMMARY: NESTED | FIELD | CONSTR | METHOD | DETAIL: FIELD | CONSTR | METHOD |


---
