# Supplementary material for: BSim: An Agent-Based Tool for Modeling Bacterial Populations in Systems and Synthetic Biology
Source: PLoS One. 2012 Aug 24;7(8):e42790. doi: 10.1371/journal.pone.0042790 (PMC3427305; doi:10.1371/journal.pone.0042790)
Supplement: Software S1 — Snapshot of the BSim software from 18th July 2012. For the latest version see: http://bsim-bccs.sf.net. The BSim software requires Java version 1.6 or higher. (ZIP) [file pone.0042790.s014.zip › BSimSoftware/docs/javadoc/bsim/particle/BSimBacterium.MotionState.html]

BSimBacterium.MotionState


---


|  |  |  |  |  |  |  |  |  |  |  |
| --- | --- | --- | --- | --- | --- | --- | --- | --- | --- | --- |
| |  |  |  |  |  |  |  |  | | --- | --- | --- | --- | --- | --- | --- | --- | | **Overview** | **Package** | **Class** | **Use** | **Tree** | **Deprecated** | **Index** | **Help** | | |  |
| **PREV CLASS**   **NEXT CLASS** | **FRAMES**    **NO FRAMES**     **All Classes** |
| SUMMARY: NESTED | ENUM CONSTANTS | FIELD | METHOD | DETAIL: ENUM CONSTANTS | FIELD | METHOD |


---


## bsim.particle Enum BSimBacterium.MotionState

```
java.lang.Object
  java.lang.Enum<BSimBacterium.MotionState>
      bsim.particle.BSimBacterium.MotionState
```

**All Implemented Interfaces:**: java.io.Serializable, java.lang.Comparable<BSimBacterium.MotionState>

**Enclosing class:**: BSimBacterium

---

``` public static enum BSimBacterium.MotionState extends java.lang.Enum<BSimBacterium.MotionState> ```

---

| **Enum Constant Summary** | |
| --- | --- |
| `RUNNING`             "When the motors turn counterclockwise, the filaments rotate in parallel in a bundle that pushes the cell body steadily forward, and the cell is said to 'run'" |
| `TUMBLING`             "When the motors turn clockwise, the flagellar filaments work independently, and the cell body moves erratically with little net displacement; the cell is then said to 'tumble'" |


| **Method Summary** | |
| --- | --- |
| `static BSimBacterium.MotionState` | `valueOf(java.lang.String name)`             Returns the enum constant of this type with the specified name. |
| `static BSimBacterium.MotionState[]` | `values()`             Returns an array containing the constants of this enum type, in the order they are declared. |

| **Methods inherited from class java.lang.Enum** |
| --- |
| `clone, compareTo, equals, finalize, getDeclaringClass, hashCode, name, ordinal, toString, valueOf` |

| **Methods inherited from class java.lang.Object** |
| --- |
| `getClass, notify, notifyAll, wait, wait, wait` |

| **Enum Constant Detail** |
| --- |

### RUNNING

```
public static final BSimBacterium.MotionState RUNNING
```

:   "When the motors turn counterclockwise, the filaments rotate in parallel in a bundle that
    pushes the cell body steadily forward, and the cell is said to 'run'"

---


### TUMBLING

```
public static final BSimBacterium.MotionState TUMBLING
```

:   "When the motors turn clockwise, the flagellar filaments work independently, and the cell body
    moves erratically with little net displacement; the cell is then said to 'tumble'"


| **Method Detail** |
| --- |

### values

```
public static BSimBacterium.MotionState[] values()
```

:   Returns an array containing the constants of this enum type, in
    the order they are declared. This method may be used to iterate
    over the constants as follows:

    ```
    for (BSimBacterium.MotionState c : BSimBacterium.MotionState.values())
        System.out.println(c);
    ```

    :   **Returns:**: an array containing the constants of this enum type, in the order they are declared

---


### valueOf

```
public static BSimBacterium.MotionState valueOf(java.lang.String name)
```

:   Returns the enum constant of this type with the specified name.
    The string must match *exactly* an identifier used to declare an
    enum constant in this type. (Extraneous whitespace characters are
    not permitted.)

    :   **Parameters:**: `name` - the name of the enum constant to be returned. **Returns:**: the enum constant with the specified name **Throws:**: `java.lang.IllegalArgumentException` - if this enum type has no constant with the specified name: `java.lang.NullPointerException` - if the argument is null


---


|  |  |  |  |  |  |  |  |  |  |  |
| --- | --- | --- | --- | --- | --- | --- | --- | --- | --- | --- |
| |  |  |  |  |  |  |  |  | | --- | --- | --- | --- | --- | --- | --- | --- | | **Overview** | **Package** | **Class** | **Use** | **Tree** | **Deprecated** | **Index** | **Help** | | |  |
| **PREV CLASS**   **NEXT CLASS** | **FRAMES**    **NO FRAMES**     **All Classes** |
| SUMMARY: NESTED | ENUM CONSTANTS | FIELD | METHOD | DETAIL: ENUM CONSTANTS | FIELD | METHOD |


---
