# Supplementary material for: BSim: An Agent-Based Tool for Modeling Bacterial Populations in Systems and Synthetic Biology
Source: PLoS One. 2012 Aug 24;7(8):e42790. doi: 10.1371/journal.pone.0042790 (PMC3427305; doi:10.1371/journal.pone.0042790)
Supplement: Software S1 — Snapshot of the BSim software from 18th July 2012. For the latest version see: http://bsim-bccs.sf.net. The BSim software requires Java version 1.6 or higher. (ZIP) [file pone.0042790.s014.zip › BSimSoftware/docs/javadoc/bsim/particle/BSimParticle.html]

BSimParticle


---


|  |  |  |  |  |  |  |  |  |  |  |
| --- | --- | --- | --- | --- | --- | --- | --- | --- | --- | --- |
| |  |  |  |  |  |  |  |  | | --- | --- | --- | --- | --- | --- | --- | --- | | **Overview** | **Package** | **Class** | **Use** | **Tree** | **Deprecated** | **Index** | **Help** | | |  |
| **PREV CLASS**   **NEXT CLASS** | **FRAMES**    **NO FRAMES**     **All Classes** |
| SUMMARY: NESTED | FIELD | CONSTR | METHOD | DETAIL: FIELD | CONSTR | METHOD |


---


## bsim.particle Class BSimParticle

```
java.lang.Object
  bsim.particle.BSimParticle
```

**Direct Known Subclasses:**: BSimBacterium, BSimVesicle

---

``` public class BSimParticle extends java.lang.Object ```

---

| **Field Summary** | |
| --- | --- |
| `protected  double` | `brownianForceMagnitude` |
| `protected  javax.vecmath.Vector3d` | `force` |
| `protected  javax.vecmath.Vector3d` | `position` |
| `protected  double` | `radius` |
| `protected static java.util.Random` | `rng` |
| `protected  BSim` | `sim` |


| **Constructor Summary** | |
| --- | --- |
| `BSimParticle(BSim sim, javax.vecmath.Vector3d position, double radius)` |


| **Method Summary** | |
| --- | --- |
| `void` | `action()`             Call in BSimTicker#tick() |
| `void` | `addForce(javax.vecmath.Vector3d f)` |
| `protected  double` | `bounceAbove(double coord, double edge)` |
| `protected  double` | `bounceBelow(double coord, double edge)` |
| `void` | `brownianForce()`             Applies a Brownian force to the particle. |
| `double` | `distance(BSimParticle p)`             Distance between particle centres (always positive) |
| `javax.vecmath.Vector3d` | `getForce()` |
| `javax.vecmath.Vector3d` | `getPosition()` |
| `double` | `getRadius()` |
| `double` | `getSurfaceArea()` |
| `boolean` | `intersection(java.util.Vector particles)`             Tests if this particle is intersecting with any in the vector |
| `void` | `logReaction(BSimParticle p, double k)`             Applies a reaction force with the properties F(0) = Inf F(this.radius + p.radius) = 0 For a particle exerting a force f, the minimum distance of approach to p is d = (this.radius + p.radius) exp(-f/k) i.e. |
| `double` | `outerDistance(BSimParticle p)`             Distance between particle edges (can be negative) |
| `void` | `reaction(BSimParticle p, double m)`             Applies a force on this of magnitude m towards this, and a force on p of magnitude m towards p. |
| `void` | `setBrownianForceMagnitude()`             Sets the magnitude of the Brownian force such that var(X(t)) = var(Y(t)) = var(Z(t)) = 2\*D\*t |
| `void` | `setRadius(double r)` |
| `void` | `setRadiusFromSurfaceArea(double s)` |
| `double` | `stokesCoefficient()` |
| `double` | `surfaceArea(double r)` |
| `void` | `updatePosition()`             Update the position of the particle according to Stokes' law |
| `protected  double` | `wrapAbove(double coord, double edge)` |
| `protected  double` | `wrapBelow(double coord, double edge)` |
| `protected  void` | `xAbove()` |
| `protected  void` | `xBelow()` |
| `protected  void` | `yAbove()` |
| `protected  void` | `yBelow()` |
| `protected  void` | `zAbove()` |
| `protected  void` | `zBelow()` |

| **Methods inherited from class java.lang.Object** |
| --- |
| `clone, equals, finalize, getClass, hashCode, notify, notifyAll, toString, wait, wait, wait` |

| **Field Detail** |
| --- |

### rng

```
protected static java.util.Random rng
```

---


### position

```
protected javax.vecmath.Vector3d position
```

---


### force

```
protected javax.vecmath.Vector3d force
```

---


### radius

```
protected double radius
```

---


### brownianForceMagnitude

```
protected double brownianForceMagnitude
```

---


### sim

```
protected BSim sim
```


| **Constructor Detail** |
| --- |

### BSimParticle

```
public BSimParticle(BSim sim,
                    javax.vecmath.Vector3d position,
                    double radius)
```


| **Method Detail** |
| --- |

### setBrownianForceMagnitude

```
public void setBrownianForceMagnitude()
```

:   Sets the magnitude of the Brownian force such that var(X(t)) = var(Y(t)) = var(Z(t)) = 2\*D\*t

---


### setRadius

```
public void setRadius(double r)
```

---


### setRadiusFromSurfaceArea

```
public void setRadiusFromSurfaceArea(double s)
```

---


### addForce

```
public void addForce(javax.vecmath.Vector3d f)
```

---


### getPosition

```
public javax.vecmath.Vector3d getPosition()
```

---


### getForce

```
public javax.vecmath.Vector3d getForce()
```

---


### getRadius

```
public double getRadius()
```

---


### getSurfaceArea

```
public double getSurfaceArea()
```

---


### surfaceArea

```
public double surfaceArea(double r)
```

---


### stokesCoefficient

```
public double stokesCoefficient()
```

---


### action

```
public void action()
```

:   Call in BSimTicker#tick()

---


### updatePosition

```
public void updatePosition()
```

:   Update the position of the particle according to Stokes' law

---


### brownianForce

```
public void brownianForce()
```

:   Applies a Brownian force to the particle. The applied force is a function of
    radius, viscosity and temperature; if viscosity or temperature is changed externally,
    you should call setBrownianForceMagnitude() again

---


### distance

```
public double distance(BSimParticle p)
```

:   Distance between particle centres (always positive)

---


### outerDistance

```
public double outerDistance(BSimParticle p)
```

:   Distance between particle edges (can be negative)

---


### intersection

```
public boolean intersection(java.util.Vector particles)
```

:   Tests if this particle is intersecting with any in the vector

---


### reaction

```
public void reaction(BSimParticle p,
                     double m)
```

:   Applies a force on this of magnitude m towards this,
    and a force on p of magnitude m towards p.

---


### logReaction

```
public void logReaction(BSimParticle p,
                        double k)
```

:   Applies a reaction force with the properties
    F(0) = Inf
    F(this.radius + p.radius) = 0
    For a particle exerting a force f, the minimum distance of approach to p is
    d = (this.radius + p.radius) exp(-f/k)
    i.e. if the particle exerts a force 1 pN, then k = 1 will prevent it from
    coming closer than (this.radius + p.radius)/e to p.

---


### xAbove

```
protected void xAbove()
```

---


### xBelow

```
protected void xBelow()
```

---


### yAbove

```
protected void yAbove()
```

---


### yBelow

```
protected void yBelow()
```

---


### zAbove

```
protected void zAbove()
```

---


### zBelow

```
protected void zBelow()
```

---


### wrapAbove

```
protected double wrapAbove(double coord,
                           double edge)
```

---


### wrapBelow

```
protected double wrapBelow(double coord,
                           double edge)
```

---


### bounceAbove

```
protected double bounceAbove(double coord,
                             double edge)
```

---


### bounceBelow

```
protected double bounceBelow(double coord,
                             double edge)
```


---


|  |  |  |  |  |  |  |  |  |  |  |
| --- | --- | --- | --- | --- | --- | --- | --- | --- | --- | --- |
| |  |  |  |  |  |  |  |  | | --- | --- | --- | --- | --- | --- | --- | --- | | **Overview** | **Package** | **Class** | **Use** | **Tree** | **Deprecated** | **Index** | **Help** | | |  |
| **PREV CLASS**   **NEXT CLASS** | **FRAMES**    **NO FRAMES**     **All Classes** |
| SUMMARY: NESTED | FIELD | CONSTR | METHOD | DETAIL: FIELD | CONSTR | METHOD |


---
