# Supplementary material for: BSim: An Agent-Based Tool for Modeling Bacterial Populations in Systems and Synthetic Biology
Source: PLoS One. 2012 Aug 24;7(8):e42790. doi: 10.1371/journal.pone.0042790 (PMC3427305; doi:10.1371/journal.pone.0042790)
Supplement: Software S1 — Snapshot of the BSim software from 18th July 2012. For the latest version see: http://bsim-bccs.sf.net. The BSim software requires Java version 1.6 or higher. (ZIP) [file pone.0042790.s014.zip › BSimSoftware/docs/javadoc/bsim/particle/BSimVesicle.html]

BSimVesicle


---


|  |  |  |  |  |  |  |  |  |  |  |
| --- | --- | --- | --- | --- | --- | --- | --- | --- | --- | --- |
| |  |  |  |  |  |  |  |  | | --- | --- | --- | --- | --- | --- | --- | --- | | **Overview** | **Package** | **Class** | **Use** | **Tree** | **Deprecated** | **Index** | **Help** | | |  |
| **PREV CLASS**   NEXT CLASS | **FRAMES**    **NO FRAMES**     **All Classes** |
| SUMMARY: NESTED | FIELD | CONSTR | METHOD | DETAIL: FIELD | CONSTR | METHOD |


---


## bsim.particle Class BSimVesicle

```
java.lang.Object
  bsim.particle.BSimParticle
      bsim.particle.BSimVesicle
```

---

``` public class BSimVesicle extends BSimParticle ```

---

| **Field Summary** | |
| --- | --- |

| **Fields inherited from class bsim.particle.BSimParticle** |
| --- |
| `brownianForceMagnitude, force, position, radius, rng, sim` |


| **Constructor Summary** | |
| --- | --- |
| `BSimVesicle(BSim sim, javax.vecmath.Vector3d position, double radius)`             Constructor for a vesicle at a position and of a given size. |


| **Method Summary** | |
| --- | --- |

| **Methods inherited from class bsim.particle.BSimParticle** |
| --- |
| `action, addForce, bounceAbove, bounceBelow, brownianForce, distance, getForce, getPosition, getRadius, getSurfaceArea, intersection, logReaction, outerDistance, reaction, setBrownianForceMagnitude, setRadius, setRadiusFromSurfaceArea, stokesCoefficient, surfaceArea, updatePosition, wrapAbove, wrapBelow, xAbove, xBelow, yAbove, yBelow, zAbove, zBelow` |

| **Methods inherited from class java.lang.Object** |
| --- |
| `clone, equals, finalize, getClass, hashCode, notify, notifyAll, toString, wait, wait, wait` |

| **Constructor Detail** |
| --- |

### BSimVesicle

```
public BSimVesicle(BSim sim,
                   javax.vecmath.Vector3d position,
                   double radius)
```

:   Constructor for a vesicle at a position and of a given size.

    **Parameters:**: `sim` - The simulation to add the vesicle to.: `position` - Position of the vesicle.: `radius` - Radius of the vesicle (microns).


---


|  |  |  |  |  |  |  |  |  |  |  |
| --- | --- | --- | --- | --- | --- | --- | --- | --- | --- | --- |
| |  |  |  |  |  |  |  |  | | --- | --- | --- | --- | --- | --- | --- | --- | | **Overview** | **Package** | **Class** | **Use** | **Tree** | **Deprecated** | **Index** | **Help** | | |  |
| **PREV CLASS**   NEXT CLASS | **FRAMES**    **NO FRAMES**     **All Classes** |
| SUMMARY: NESTED | FIELD | CONSTR | METHOD | DETAIL: FIELD | CONSTR | METHOD |


---
