# Supplementary material for: BSim: An Agent-Based Tool for Modeling Bacterial Populations in Systems and Synthetic Biology
Source: PLoS One. 2012 Aug 24;7(8):e42790. doi: 10.1371/journal.pone.0042790 (PMC3427305; doi:10.1371/journal.pone.0042790)
Supplement: Software S1 — Snapshot of the BSim software from 18th July 2012. For the latest version see: http://bsim-bccs.sf.net. The BSim software requires Java version 1.6 or higher. (ZIP) [file pone.0042790.s014.zip › BSimSoftware/docs/javadoc/bsim/particle/class-use/BSimParticle.html]

Uses of Class bsim.particle.BSimParticle


---


|  |  |  |  |  |  |  |  |  |  |  |
| --- | --- | --- | --- | --- | --- | --- | --- | --- | --- | --- |
| |  |  |  |  |  |  |  |  | | --- | --- | --- | --- | --- | --- | --- | --- | | **Overview** | **Package** | **Class** | **Use** | **Tree** | **Deprecated** | **Index** | **Help** | | |  |
| PREV   NEXT | **FRAMES**    **NO FRAMES**     **All Classes** |


---


## **Uses of Class bsim.particle.BSimParticle**

| Packages that use BSimParticle | |
| --- | --- |
| **bsim.draw** |  |
| **bsim.geometry** |  |
| **bsim.particle** |  |

| Uses of BSimParticle in bsim.draw | |
| --- | --- |

| Methods in bsim.draw with parameters of type BSimParticle | |
| --- | --- |
| `void` | `BSimP3DDrawer.draw(BSimParticle p, java.awt.Color c)`             Draw a BSimParticle as a point if it is very small (radius < 1), or a sphere otherwise. |

| Uses of BSimParticle in bsim.geometry | |
| --- | --- |

| Methods in bsim.geometry with parameters of type BSimParticle | |
| --- | --- |
| `static void` | `BSimCollision.collideAndRepel(BSimParticle p, BSimMesh theMesh)`             Check for collision between particle and mesh, and add repulsion force. |
| `static boolean` | `BSimMeshUtils.intersectSpherePlane(BSimParticle s, javax.vecmath.Vector3d planeNormal, javax.vecmath.Vector3d a)`             Computes the intersection of a sphere with a plane |
| `static boolean` | `BSimMeshUtils.intersectSphereTriangle(BSimParticle s, javax.vecmath.Vector3d a, javax.vecmath.Vector3d b, javax.vecmath.Vector3d c, javax.vecmath.Vector3d p)`             Intersection of a sphere with a triangle |

| Uses of BSimParticle in bsim.particle | |
| --- | --- |

| Subclasses of BSimParticle in bsim.particle | |
| --- | --- |
| `class` | `BSimBacterium`             Class representing a bacterium whose run-tumble motion is affected in a simple way by a single goal chemical. |
| `class` | `BSimVesicle` |

| Methods in bsim.particle with parameters of type BSimParticle | |
| --- | --- |
| `double` | `BSimParticle.distance(BSimParticle p)`             Distance between particle centres (always positive) |
| `void` | `BSimParticle.logReaction(BSimParticle p, double k)`             Applies a reaction force with the properties F(0) = Inf F(this.radius + p.radius) = 0 For a particle exerting a force f, the minimum distance of approach to p is d = (this.radius + p.radius) exp(-f/k) i.e. |
| `double` | `BSimParticle.outerDistance(BSimParticle p)`             Distance between particle edges (can be negative) |
| `void` | `BSimParticle.reaction(BSimParticle p, double m)`             Applies a force on this of magnitude m towards this, and a force on p of magnitude m towards p. |

---


|  |  |  |  |  |  |  |  |  |  |  |
| --- | --- | --- | --- | --- | --- | --- | --- | --- | --- | --- |
| |  |  |  |  |  |  |  |  | | --- | --- | --- | --- | --- | --- | --- | --- | | **Overview** | **Package** | **Class** | **Use** | **Tree** | **Deprecated** | **Index** | **Help** | | |  |
| PREV   NEXT | **FRAMES**    **NO FRAMES**     **All Classes** |


---
