# Supplementary material for: BSim: An Agent-Based Tool for Modeling Bacterial Populations in Systems and Synthetic Biology
Source: PLoS One. 2012 Aug 24;7(8):e42790. doi: 10.1371/journal.pone.0042790 (PMC3427305; doi:10.1371/journal.pone.0042790)
Supplement: Software S1 — Snapshot of the BSim software from 18th July 2012. For the latest version see: http://bsim-bccs.sf.net. The BSim software requires Java version 1.6 or higher. (ZIP) [file pone.0042790.s014.zip › BSimSoftware/docs/javadoc/index-files/index-1.html]

A-Index


---


|  |  |  |  |  |  |  |  |  |  |  |
| --- | --- | --- | --- | --- | --- | --- | --- | --- | --- | --- |
| |  |  |  |  |  |  |  |  | | --- | --- | --- | --- | --- | --- | --- | --- | | **Overview** | Package | Class | Use | **Tree** | **Deprecated** | **Index** | **Help** | | |  |
| PREV LETTER   **NEXT LETTER** | **FRAMES**    **NO FRAMES**     **All Classes** |


A B C D E F G H I K L M N O P Q R S T U V W X Y Z 

---


## **A**

**action()** - Method in class bsim.particle.BSimBacterium: **action()** - Method in class bsim.particle.BSimParticle: Call in BSimTicker#tick() **addExporter(BSimExporter)** - Method in class bsim.BSim: Add an exporter to be called during simulation. **addForce(Vector3d)** - Method in class bsim.particle.BSimParticle: **addQuantity(Vector3d, double)** - Method in class bsim.BSimChemicalField: Adds a quantity of chemical to the box containing position v. **addQuantity(int, int, int, double)** - Method in class bsim.BSimChemicalField: Adds a quantity of chemical to the box (x,y,z). **addScaledVertex(Vector3d)** - Method in class bsim.geometry.BSimSphereMesh: Helper wrapper: ultimately the same method as BSimFVMesh.addVertex(), but scales the vertex position to be a unit distance from the origin. **addTriangle(int, int, int)** - Method in class bsim.geometry.BSimMesh: Add a triangular face to the face list. **addTriangle(BSimTriangle)** - Method in class bsim.geometry.BSimMesh: Add an existing triangle to the face list **addVertex(double, double, double)** - Method in class bsim.geometry.BSimMesh: Add a vertex to the vertex list (based on x,y,z coordinates). **addVertex(Vector3d)** - Method in class bsim.geometry.BSimMesh: Add a vertex to the vertex list (using a Vector3d). **after()** - Method in class bsim.export.BSimExporter: Called after a simulation finishes (overwrite). **after()** - Method in class bsim.export.BSimLogger: Called after a simulation ends. **after()** - Method in class bsim.export.BSimMovExporter: Called after the simulation ends. **after()** - Method in class bsim.export.BSimPngExporter: Called after the simulation ends. **assignTrianglesFromParentMesh(KdNode)** - Method in class bsim.geometry.KdNode: Assigns triangles from the parent mesh of the k-d tree to each (leaf) node. **AtomDataOutputStream** - Class in bsim.export.quicktime: This output stream filter supports common data types used inside of QuickTime Atoms. **AtomDataOutputStream(OutputStream)** - Constructor for class bsim.export.quicktime.AtomDataOutputStream: **averagedCentreOfMesh()** - Method in class bsim.geometry.BSimMesh: Compute the (unweighted) average centre coordinate of all mesh vertices.

---


|  |  |  |  |  |  |  |  |  |  |  |
| --- | --- | --- | --- | --- | --- | --- | --- | --- | --- | --- |
| |  |  |  |  |  |  |  |  | | --- | --- | --- | --- | --- | --- | --- | --- | | **Overview** | Package | Class | Use | **Tree** | **Deprecated** | **Index** | **Help** | | |  |
| PREV LETTER   **NEXT LETTER** | **FRAMES**    **NO FRAMES**     **All Classes** |


A B C D E F G H I K L M N O P Q R S T U V W X Y Z 

---
