# Supplementary material for: BSim: An Agent-Based Tool for Modeling Bacterial Populations in Systems and Synthetic Biology
Source: PLoS One. 2012 Aug 24;7(8):e42790. doi: 10.1371/journal.pone.0042790 (PMC3427305; doi:10.1371/journal.pone.0042790)
Supplement: Software S1 — Snapshot of the BSim software from 18th July 2012. For the latest version see: http://bsim-bccs.sf.net. The BSim software requires Java version 1.6 or higher. (ZIP) [file pone.0042790.s014.zip › BSimSoftware/docs/javadoc/index-files/index-10.html]

K-Index


---


|  |  |  |  |  |  |  |  |  |  |  |
| --- | --- | --- | --- | --- | --- | --- | --- | --- | --- | --- |
| |  |  |  |  |  |  |  |  | | --- | --- | --- | --- | --- | --- | --- | --- | | **Overview** | Package | Class | Use | **Tree** | **Deprecated** | **Index** | **Help** | | |  |
| **PREV LETTER**   **NEXT LETTER** | **FRAMES**    **NO FRAMES**     **All Classes** |


A B C D E F G H I K L M N O P Q R S T U V W X Y Z 

---


## **K**

**KdNode** - Class in bsim.geometry: See, for example, http://en.wikipedia.org/wiki/Kd-tree Effectively an axis-aligned BSP tree which alternates the splitting plane axis at each level of branching. **KdNode()** - Constructor for class bsim.geometry.KdNode: Default constructor. **KdNode.Indexed3d** - Class in bsim.geometry: Class that holds a triplet of doubles and its original index. **KdNode.Indexed3d()** - Constructor for class bsim.geometry.KdNode.Indexed3d: **KdNode.Indexed3d(double[], int)** - Constructor for class bsim.geometry.KdNode.Indexed3d: **KdNode.Indexed3d(KdNode.Indexed3d)** - Constructor for class bsim.geometry.KdNode.Indexed3d: **KdNode.TestMesh** - Class in bsim.geometry: **KdNode.TestMesh()** - Constructor for class bsim.geometry.KdNode.TestMesh: **kdTreeFromMesh(BSimMesh)** - Method in class bsim.geometry.KdNode: **kdTreeMeshTest()** - Method in class bsim.geometry.KdNode

---


|  |  |  |  |  |  |  |  |  |  |  |
| --- | --- | --- | --- | --- | --- | --- | --- | --- | --- | --- |
| |  |  |  |  |  |  |  |  | | --- | --- | --- | --- | --- | --- | --- | --- | | **Overview** | Package | Class | Use | **Tree** | **Deprecated** | **Index** | **Help** | | |  |
| **PREV LETTER**   **NEXT LETTER** | **FRAMES**    **NO FRAMES**     **All Classes** |


A B C D E F G H I K L M N O P Q R S T U V W X Y Z 

---
