# Supplementary material for: BSim: An Agent-Based Tool for Modeling Bacterial Populations in Systems and Synthetic Biology
Source: PLoS One. 2012 Aug 24;7(8):e42790. doi: 10.1371/journal.pone.0042790 (PMC3427305; doi:10.1371/journal.pone.0042790)
Supplement: Software S1 — Snapshot of the BSim software from 18th July 2012. For the latest version see: http://bsim-bccs.sf.net. The BSim software requires Java version 1.6 or higher. (ZIP) [file pone.0042790.s014.zip › BSimSoftware/docs/javadoc/index-files/index-11.html]

L-Index


---


|  |  |  |  |  |  |  |  |  |  |  |
| --- | --- | --- | --- | --- | --- | --- | --- | --- | --- | --- |
| |  |  |  |  |  |  |  |  | | --- | --- | --- | --- | --- | --- | --- | --- | | **Overview** | Package | Class | Use | **Tree** | **Deprecated** | **Index** | **Help** | | |  |
| **PREV LETTER**   **NEXT LETTER** | **FRAMES**    **NO FRAMES**     **All Classes** |


A B C D E F G H I K L M N O P Q R S T U V W X Y Z 

---


## **L**

**leftChild** - Variable in class bsim.geometry.KdNode: Left child: coordinate of interest < splitting plane **leftTris** - Variable in class bsim.geometry.KdNode: Triangles that are classified as being to the left of the node. **length** - Variable in class bsim.BSimOctreeField: Dimension of the node. **linearGradient(int, double, double)** - Method in class bsim.BSimChemicalField: Creates a linear concentration gradient in the direction specified by 'axis' (x=0, y=1, z=2) **load(String)** - Method in class bsim.geometry.BSimOBJMesh: Loads an OBJ file from disk and puts relevant parameters into a BSimMesh **location** - Variable in class bsim.geometry.BSimVertex: The Cartesian coordinates of the vertex in 3-D space. **location** - Variable in class bsim.geometry.KdNode: Index of the vertex on which the splitting plane is located **logReaction(BSimParticle, double)** - Method in class bsim.particle.BSimParticle: Applies a reaction force with the properties F(0) = Inf F(this.radius + p.radius) = 0 For a particle exerting a force f, the minimum distance of approach to p is d = (this.radius + p.radius) exp(-f/k) i.e. **longTermMemoryDuration** - Variable in class bsim.particle.BSimBacterium: **longTermMemoryLength** - Variable in class bsim.particle.BSimBacterium: sim.timesteps(longTermMemoryDuration)

---


|  |  |  |  |  |  |  |  |  |  |  |
| --- | --- | --- | --- | --- | --- | --- | --- | --- | --- | --- |
| |  |  |  |  |  |  |  |  | | --- | --- | --- | --- | --- | --- | --- | --- | | **Overview** | Package | Class | Use | **Tree** | **Deprecated** | **Index** | **Help** | | |  |
| **PREV LETTER**   **NEXT LETTER** | **FRAMES**    **NO FRAMES**     **All Classes** |


A B C D E F G H I K L M N O P Q R S T U V W X Y Z 

---
