# Supplementary material for: BSim: An Agent-Based Tool for Modeling Bacterial Populations in Systems and Synthetic Biology
Source: PLoS One. 2012 Aug 24;7(8):e42790. doi: 10.1371/journal.pone.0042790 (PMC3427305; doi:10.1371/journal.pone.0042790)
Supplement: Software S1 — Snapshot of the BSim software from 18th July 2012. For the latest version see: http://bsim-bccs.sf.net. The BSim software requires Java version 1.6 or higher. (ZIP) [file pone.0042790.s014.zip › BSimSoftware/docs/javadoc/index-files/index-15.html]

P-Index


---


|  |  |  |  |  |  |  |  |  |  |  |
| --- | --- | --- | --- | --- | --- | --- | --- | --- | --- | --- |
| |  |  |  |  |  |  |  |  | | --- | --- | --- | --- | --- | --- | --- | --- | | **Overview** | Package | Class | Use | **Tree** | **Deprecated** | **Index** | **Help** | | |  |
| **PREV LETTER**   **NEXT LETTER** | **FRAMES**    **NO FRAMES**     **All Classes** |


A B C D E F G H I K L M N O P Q R S T U V W X Y Z 

---


## **P**

**p3d** - Variable in class bsim.draw.BSimP3DDrawer: Processing graphics context used for drawing. **padInt2(int)** - Static method in class bsim.BSimUtils: Returns a padded version of the number to a size of two. **parent** - Variable in class bsim.BSimOctreeField: Parent of node, for root this is null. **parentMesh** - Variable in class bsim.geometry.BSimTriangle: The mesh to which this triangle belongs. **parentMesh** - Variable in class bsim.geometry.KdNode: **partition(KdNode.Indexed3d[], int, int, int, int)** - Method in class bsim.geometry.KdNode: **pEndRun()** - Method in class bsim.particle.BSimBacterium: Probability per per unit time of ending a run **pEndRunElse** - Variable in class bsim.particle.BSimBacterium: Probability per per unit time of ending a run otherwise **pEndRunElse(double)** - Method in class bsim.particle.BSimBacterium: **pEndRunUp** - Variable in class bsim.particle.BSimBacterium: Probability per per unit time of ending a run when moving up a chemical gradient **pEndRunUp(double)** - Method in class bsim.particle.BSimBacterium: **pEndTumble** - Variable in class bsim.particle.BSimBacterium: Probability per per unit time of ending a tumble **pEndTumble()** - Method in class bsim.particle.BSimBacterium: Probability per per unit time of ending a tumble. **pEndTumble(double)** - Method in class bsim.particle.BSimBacterium: **point(Vector3d, Color)** - Method in class bsim.draw.BSimP3DDrawer: Draw a point (pixel); parametrised helper function. **pos** - Variable in class bsim.geometry.BSimCollision: **position** - Variable in class bsim.particle.BSimParticle: **postOrderfull(BSimOctreeField)** - Static method in class bsim.BSimOctreeField: Post-Order traverse with visit function. **preOrderfull(BSimOctreeField)** - Static method in class bsim.BSimOctreeField: Pre-Order full traverse - traverses from the root, a direction to the deepest subnode, back to the node, and then down into other roots. **preview()** - Method in class bsim.BSim: Runs the simulation in a frame until the frame is closed, ignoring exporters. **printStats()** - Method in class bsim.geometry.BSimMesh: Print mesh statistics (face vertices, vertex coords, vertex faces, normals...) **processed** - Variable in class bsim.BSimOctreeField: For checking in division algorithm. **pVesicle** - Variable in class bsim.particle.BSimBacterium: Probability per typical vesicle surface area growth of producing a vesicle **pVesicle(double)** - Method in class bsim.particle.BSimBacterium

---


|  |  |  |  |  |  |  |  |  |  |  |
| --- | --- | --- | --- | --- | --- | --- | --- | --- | --- | --- |
| |  |  |  |  |  |  |  |  | | --- | --- | --- | --- | --- | --- | --- | --- | | **Overview** | Package | Class | Use | **Tree** | **Deprecated** | **Index** | **Help** | | |  |
| **PREV LETTER**   **NEXT LETTER** | **FRAMES**    **NO FRAMES**     **All Classes** |


A B C D E F G H I K L M N O P Q R S T U V W X Y Z 

---
