# Supplementary material for: BSim: An Agent-Based Tool for Modeling Bacterial Populations in Systems and Synthetic Biology
Source: PLoS One. 2012 Aug 24;7(8):e42790. doi: 10.1371/journal.pone.0042790 (PMC3427305; doi:10.1371/journal.pone.0042790)
Supplement: Software S1 — Snapshot of the BSim software from 18th July 2012. For the latest version see: http://bsim-bccs.sf.net. The BSim software requires Java version 1.6 or higher. (ZIP) [file pone.0042790.s014.zip › BSimSoftware/docs/javadoc/index-files/index-17.html]

R-Index


---


|  |  |  |  |  |  |  |  |  |  |  |
| --- | --- | --- | --- | --- | --- | --- | --- | --- | --- | --- |
| |  |  |  |  |  |  |  |  | | --- | --- | --- | --- | --- | --- | --- | --- | | **Overview** | Package | Class | Use | **Tree** | **Deprecated** | **Index** | **Help** | | |  |
| **PREV LETTER**   **NEXT LETTER** | **FRAMES**    **NO FRAMES**     **All Classes** |


A B C D E F G H I K L M N O P Q R S T U V W X Y Z 

---


## **R**

**radius** - Variable in class bsim.particle.BSimParticle: **reaction(BSimParticle, double)** - Method in class bsim.particle.BSimParticle: Applies a force on this of magnitude m towards this, and a force on p of magnitude m towards p. **recursiveCollisions** - Static variable in class bsim.geometry.BSimCollision: **replicate()** - Method in class bsim.particle.BSimBacterium: **replicationRadius** - Variable in class bsim.particle.BSimBacterium: **rightChild** - Variable in class bsim.geometry.KdNode: Right child: coordinate of interest > splitting plane **rightTris** - Variable in class bsim.geometry.KdNode: Triangles that are classified as being to the right of this node. **rng** - Static variable in class bsim.particle.BSimParticle: **rotate(Vector3d, Vector3d, double)** - Static method in class bsim.BSimUtils: Rotates the vector v towards the specified axis by an angle theta. **rotatePerp(Vector3d, double)** - Static method in class bsim.BSimUtils: Rotates the vector v by an angle theta in a random direction perpendicular to v. **rotationalDiffusion()** - Method in class bsim.particle.BSimBacterium: Causes the cell to rotate such that Var(theta(dt)) = 4\*D\*dt. **rotationalStokesCoefficient()** - Method in class bsim.particle.BSimBacterium: **run()** - Method in class bsim.BSimThreadedTickerWorker: Threaded function. **rungeKutta23(BSimDdeSystem, double, Vector<double[]>, double)** - Static method in class bsim.dde.BSimDdeSolver: Numerically solve an DDE system with 2nd order Runge-Kutta method. **rungeKutta23(BSimOdeSystem, double, double[], double)** - Static method in class bsim.ode.BSimOdeSolver: Numerically solve an ODE system with 2nd order Runge-Kutta method. **rungeKutta45(BSimDdeSystem, double, Vector<double[]>, double)** - Static method in class bsim.dde.BSimDdeSolver: Numerically solve an DDE system with 4th order Runge-Kutta method. **rungeKutta45(BSimOdeSystem, double, double[], double)** - Static method in class bsim.ode.BSimOdeSolver: Numerically solve an ODE system with 4th order Runge-Kutta method

---


|  |  |  |  |  |  |  |  |  |  |  |
| --- | --- | --- | --- | --- | --- | --- | --- | --- | --- | --- |
| |  |  |  |  |  |  |  |  | | --- | --- | --- | --- | --- | --- | --- | --- | | **Overview** | Package | Class | Use | **Tree** | **Deprecated** | **Index** | **Help** | | |  |
| **PREV LETTER**   **NEXT LETTER** | **FRAMES**    **NO FRAMES**     **All Classes** |


A B C D E F G H I K L M N O P Q R S T U V W X Y Z 

---
