# Supplementary material for: BSim: An Agent-Based Tool for Modeling Bacterial Populations in Systems and Synthetic Biology
Source: PLoS One. 2012 Aug 24;7(8):e42790. doi: 10.1371/journal.pone.0042790 (PMC3427305; doi:10.1371/journal.pone.0042790)
Supplement: Software S1 — Snapshot of the BSim software from 18th July 2012. For the latest version see: http://bsim-bccs.sf.net. The BSim software requires Java version 1.6 or higher. (ZIP) [file pone.0042790.s014.zip › BSimSoftware/docs/javadoc/index-files/index-18.html]

S-Index


---


|  |  |  |  |  |  |  |  |  |  |  |
| --- | --- | --- | --- | --- | --- | --- | --- | --- | --- | --- |
| |  |  |  |  |  |  |  |  | | --- | --- | --- | --- | --- | --- | --- | --- | | **Overview** | Package | Class | Use | **Tree** | **Deprecated** | **Index** | **Help** | | |  |
| **PREV LETTER**   **NEXT LETTER** | **FRAMES**    **NO FRAMES**     **All Classes** |


A B C D E F G H I K L M N O P Q R S T U V W X Y Z 

---


## **S**

**sampleGamma(double, double)** - Static method in class bsim.BSimUtils: Sample from a gamma distribution. **scale(double, Vector3d)** - Method in class bsim.geometry.BSimMesh: Scale mesh on arbitrary point **scale(double)** - Method in class bsim.geometry.BSimMesh: Scale mesh on origin (0, 0, 0). **scene(PGraphics3D)** - Method in class bsim.draw.BSimP3DDrawer: Draws remaining scene objects to the PGraphics3D object. **sensitivity** - Variable in class bsim.particle.BSimBacterium: Sensitivity to differences in sequential averages (molecules/(micron)^3). **sequentialAfter()** - Method in class bsim.BSimThreadedTicker: **sequentialBefore()** - Method in class bsim.BSimThreadedTicker: Overwrite these with sequential operations to run before and after the parallel block. **set(BSimTriangle, double, double, double, double)** - Method in class bsim.geometry.BSimCollision: **set(double, Vector3d, Vector3d)** - Method in class bsim.geometry.BSimCollision: **set(BSimCollision)** - Method in class bsim.geometry.BSimCollision: Set the collision values. **set(KdNode.Indexed3d)** - Method in class bsim.geometry.KdNode.Indexed3d: **set(double[], int)** - Method in class bsim.geometry.KdNode.Indexed3d: **setBound(double, double, double)** - Method in class bsim.BSim: Set the simulation bound (microns). **setBrownianForceMagnitude()** - Method in class bsim.particle.BSimParticle: Sets the magnitude of the Brownian force such that var(X(t)) = var(Y(t)) = var(Z(t)) = 2\*D\*t **setChildList(Vector)** - Method in class bsim.particle.BSimBacterium: **setColor(Color)** - Method in class bsim.BSimOctreeField: Set the colour. **setConc(Vector3d, double)** - Method in class bsim.BSimChemicalField: Sets the concentration of the box containing position v. **setConc(int, int, int, double)** - Method in class bsim.BSimChemicalField: Sets the concentration of the box (x,y,z). **setConc(double)** - Method in class bsim.BSimChemicalField: Sets the concentration of the field **setDirection(Vector3d)** - Method in class bsim.particle.BSimBacterium: Set the direction of the cell to the direction of the vector v. **setDrawer(BSimDrawer)** - Method in class bsim.BSim: Set the drawer to be used during simulation. **setDt(double)** - Method in class bsim.BSim: Set the timestep (secs). **setDt(double)** - Method in class bsim.export.BSimExporter: Set the time interval that the exporter is called. **setForceMagnitude(double)** - Method in class bsim.particle.BSimBacterium: **setGoal(BSimChemicalField)** - Method in class bsim.particle.BSimBacterium: Set this chemical field as the goal field. **setInitialHistory(Vector<double[]>)** - Method in interface bsim.dde.BSimDdeSystem: Sets the initial history when solving. **setKids()** - Method in class bsim.BSimOctreeField: Initializes all the subNodes with appropriate neighbors/centers and lengths. **setLeaky(boolean, boolean, boolean, boolean, boolean, boolean)** - Method in class bsim.BSim: Set whether the boundaries are leaky. **setLeakyRate(double, double, double, double, double, double)** - Method in class bsim.BSim: Set the rate that chemicals can escape from the simulation (if the boundary is leaky). **setMemoryDuration(double, double)** - Method in class bsim.particle.BSimBacterium: **setMesh(BSimMesh)** - Method in class bsim.geometry.BSimTriangle: Set parent mesh to which this face belongs. **setMotionState(BSimBacterium.MotionState)** - Method in class bsim.particle.BSimBacterium: **setNodestoMesh(BSimMesh, BSimOctreeField)** - Method in class bsim.BSimOctreeField: Fits octreeField against a mesh and splits into subNodes when there is a collision with the mesh boundary Creates a finer octree structure each time this function is called. **setRadius()** - Method in class bsim.particle.BSimBacterium: Sets the radius so that the surface area of the bacterium is randomly distributed between surfaceArea(replicationRadius)/2 and surfaceArea(replicationRadius) **setRadius(double)** - Method in class bsim.particle.BSimParticle: **setRadiusFromSurfaceArea(double)** - Method in class bsim.particle.BSimParticle: **setRecursiveCollisions(boolean)** - Static method in class bsim.geometry.BSimCollision: Set whether recursive collisions are enabled. **setReplicationRadius(double)** - Method in class bsim.particle.BSimBacterium: **setSimulationTime(double)** - Method in class bsim.BSim: Set the length of the simulation (secs). **setSolid(boolean, boolean, boolean)** - Method in class bsim.BSim: Set whether the boundaries are solid (reflecting) or wrapping (periiodic). **setSpeed(int)** - Method in class bsim.export.BSimMovExporter: Speeds up the movie relative to simulation time by a factor f, that is, 1 second in the simulation will last 1/f seconds in the movie **setSurfaceAreaGrowthRate()** - Method in class bsim.particle.BSimBacterium: **setSurfaceAreaGrowthRate(double)** - Method in class bsim.particle.BSimBacterium: **setTemperature(double)** - Method in class bsim.BSim: Set the temperature of the environment. **setTicker(BSimTicker)** - Method in class bsim.BSim: Set the ticker to be used during simulation. **setTimeFormat(String)** - Method in class bsim.BSim: Set the time format. **setTimeScale(int)** - Method in class bsim.export.quicktime.QuickTimeOutputStream: Sets the time scale for this media, that is, the number of time units that pass per second in its time coordinate system. **setTimeStamp(long)** - Method in class bsim.geometry.BSimTriangle: Set the time stamp associated with the triangle. **setVesicleList(Vector)** - Method in class bsim.particle.BSimBacterium: **setVideoCompressionQuality(float)** - Method in class bsim.export.quicktime.QuickTimeOutputStream: Sets the compression quality of the video track. **setVideoDimension(int, int)** - Method in class bsim.export.quicktime.QuickTimeOutputStream: Sets the dimension of the video track. **setVisc(double)** - Method in class bsim.BSim: Set the viscosity of the environment. **shiftState(Vector<double[]>)** - Static method in class bsim.dde.BSimDdeSolver: Shifts all elements in the state vector by one timestep. **shortTermMemoryDuration** - Variable in class bsim.particle.BSimBacterium: **shortTermMemoryLength** - Variable in class bsim.particle.BSimBacterium: sim.timesteps(shortTermMemoryDuration) **sim** - Variable in class bsim.BSimChemicalField: Simulation the chemical field is associated with. **sim** - Variable in class bsim.draw.BSimDrawer: The simulation. **sim** - Variable in class bsim.export.BSimExporter: Associated simulation. **sim** - Variable in class bsim.particle.BSimParticle: **size()** - Method in class bsim.export.quicktime.AtomDataOutputStream: Returns the current value of the counter `written`, the number of bytes written to this data output stream so far. **speed** - Variable in class bsim.export.BSimMovExporter: Speed of the movie. **sphere(Vector3d, double, Color, int)** - Method in class bsim.draw.BSimP3DDrawer: Draw sphere; helper function which draws a parametrised sphere. **stokesCoefficient()** - Method in class bsim.particle.BSimParticle: **subNodes** - Variable in class bsim.BSimOctreeField: subNodes of octree, these can have subnodes of their own. **surfaceArea(double)** - Method in class bsim.particle.BSimParticle: **surfaceAreaGrowthRate** - Variable in class bsim.particle.BSimBacterium

---


|  |  |  |  |  |  |  |  |  |  |  |
| --- | --- | --- | --- | --- | --- | --- | --- | --- | --- | --- |
| |  |  |  |  |  |  |  |  | | --- | --- | --- | --- | --- | --- | --- | --- | | **Overview** | Package | Class | Use | **Tree** | **Deprecated** | **Index** | **Help** | | |  |
| **PREV LETTER**   **NEXT LETTER** | **FRAMES**    **NO FRAMES**     **All Classes** |


A B C D E F G H I K L M N O P Q R S T U V W X Y Z 

---
