# Supplementary material for: BSim: An Agent-Based Tool for Modeling Bacterial Populations in Systems and Synthetic Biology
Source: PLoS One. 2012 Aug 24;7(8):e42790. doi: 10.1371/journal.pone.0042790 (PMC3427305; doi:10.1371/journal.pone.0042790)
Supplement: Software S1 — Snapshot of the BSim software from 18th July 2012. For the latest version see: http://bsim-bccs.sf.net. The BSim software requires Java version 1.6 or higher. (ZIP) [file pone.0042790.s014.zip › BSimSoftware/docs/javadoc/index-files/index-19.html]

T-Index


---


|  |  |  |  |  |  |  |  |  |  |  |
| --- | --- | --- | --- | --- | --- | --- | --- | --- | --- | --- |
| |  |  |  |  |  |  |  |  | | --- | --- | --- | --- | --- | --- | --- | --- | | **Overview** | Package | Class | Use | **Tree** | **Deprecated** | **Index** | **Help** | | |  |
| **PREV LETTER**   **NEXT LETTER** | **FRAMES**    **NO FRAMES**     **All Classes** |


A B C D E F G H I K L M N O P Q R S T U V W X Y Z 

---


## **T**

**t** - Variable in class bsim.geometry.BSimCollision: **threadedTick(int, int)** - Method in class bsim.BSimThreadedTickerWorker: Run each timestep in parallel, use the threadID to figure out which part of the problem to work on. **threadID** - Variable in class bsim.BSimThreadedTickerWorker: The ID of this worker. **threads** - Variable in class bsim.BSimThreadedTicker: Total number of threads (including main one). **threads** - Variable in class bsim.BSimThreadedTickerWorker: Total number of threads in the pool. **tick()** - Method in class bsim.BSimThreadedTicker: Called at each time step by the BSim simulation object. **tick()** - Method in class bsim.BSimTicker: Run each timestep, this is where you should update particle properties by calling interaction(), action() and updatePosition() methods **time()** - Method in class bsim.draw.BSimP3DDrawer: Draw the formatted simulation time to screen. **timeStamp()** - Static method in class bsim.BSimUtils: Returns a string representation of the current date and time. **timeStamp** - Variable in class bsim.geometry.BSimTriangle: Time stamp. **timesteps(double)** - Method in class bsim.BSim: Returns the number of complete timesteps in the duration d. **totalQuantity()** - Method in class bsim.BSimChemicalField: Returns the total quantity of chemical in the field. **translate(Vector3d)** - Method in class bsim.geometry.BSimMesh: Translate the mesh in an arbitrary direction. **translateAbsolute(Vector3d)** - Method in class bsim.geometry.BSimMesh: Translate the whole mesh so that it is centred on a new point in 3d space. **tumbleAngle()** - Method in class bsim.particle.BSimBacterium: Return a tumble angle in radians distributed according to Fig. **tVertices** - Variable in class bsim.geometry.BSimTriangle: **typicalVesicleSurfaceArea** - Variable in class bsim.particle.BSimBacterium

---


|  |  |  |  |  |  |  |  |  |  |  |
| --- | --- | --- | --- | --- | --- | --- | --- | --- | --- | --- |
| |  |  |  |  |  |  |  |  | | --- | --- | --- | --- | --- | --- | --- | --- | | **Overview** | Package | Class | Use | **Tree** | **Deprecated** | **Index** | **Help** | | |  |
| **PREV LETTER**   **NEXT LETTER** | **FRAMES**    **NO FRAMES**     **All Classes** |


A B C D E F G H I K L M N O P Q R S T U V W X Y Z 

---
