# Supplementary material for: BSim: An Agent-Based Tool for Modeling Bacterial Populations in Systems and Synthetic Biology
Source: PLoS One. 2012 Aug 24;7(8):e42790. doi: 10.1371/journal.pone.0042790 (PMC3427305; doi:10.1371/journal.pone.0042790)
Supplement: Software S1 — Snapshot of the BSim software from 18th July 2012. For the latest version see: http://bsim-bccs.sf.net. The BSim software requires Java version 1.6 or higher. (ZIP) [file pone.0042790.s014.zip › BSimSoftware/docs/javadoc/index-files/index-2.html]

B-Index


---


|  |  |  |  |  |  |  |  |  |  |  |
| --- | --- | --- | --- | --- | --- | --- | --- | --- | --- | --- |
| |  |  |  |  |  |  |  |  | | --- | --- | --- | --- | --- | --- | --- | --- | | **Overview** | Package | Class | Use | **Tree** | **Deprecated** | **Index** | **Help** | | |  |
| **PREV LETTER**   **NEXT LETTER** | **FRAMES**    **NO FRAMES**     **All Classes** |


A B C D E F G H I K L M N O P Q R S T U V W X Y Z 

---


## **B**

**barrier1** - Static variable in class bsim.BSimThreadedTickerWorker: Shared barrier to enable synchronisation of all threads at start of update. **barrier2** - Static variable in class bsim.BSimThreadedTickerWorker: Shared barrier to enable synchronisation of all threads at end of update. **before()** - Method in class bsim.export.BSimExporter: Called before a simulation starts (overwrite). **before()** - Method in class bsim.export.BSimLogger: Called before a simulation starts. **before()** - Method in class bsim.export.BSimMovExporter: Called before the simulation starts. **before()** - Method in class bsim.export.BSimPngExporter: Called before a simulation starts. **BOLTZMANN** - Static variable in class bsim.BSim: Boltzmann constant. **bounceAbove(double, double)** - Method in class bsim.particle.BSimParticle: **bounceBelow(double, double)** - Method in class bsim.particle.BSimParticle: **bound** - Variable in class bsim.BSimChemicalField: sim.getBound(). **bound** - Variable in class bsim.draw.BSimP3DDrawer: Size of the simulation. **boundaries()** - Method in class bsim.draw.BSimP3DDrawer: Draw the default cuboid boundary of the simulation as a partially transparent box with a wireframe outline surrounding it. **boundaryOutline()** - Method in class bsim.draw.BSimP3DDrawer: Draw the default cuboid boundary of the simulation as a wireframe outline. **boundCentre** - Variable in class bsim.draw.BSimP3DDrawer: Centre of the simulation. **box** - Variable in class bsim.BSimChemicalField: Box size. **boxCoords(Vector3d)** - Method in class bsim.BSimChemicalField: Returns the integer coordinates of the box containing the position v. **boxes** - Variable in class bsim.BSimChemicalField: Number of boxes in each dimension. **boxVolume** - Variable in class bsim.BSimChemicalField: Volume of each box (microns^3). **brownianForce()** - Method in class bsim.particle.BSimParticle: Applies a Brownian force to the particle. **brownianForceMagnitude** - Variable in class bsim.particle.BSimParticle: **bsim** - package bsim: **BSim** - Class in bsim: Main simulation class. **BSim()** - Constructor for class bsim.BSim: **bsim.dde** - package bsim.dde: **bsim.draw** - package bsim.draw: **bsim.export** - package bsim.export: **bsim.export.quicktime** - package bsim.export.quicktime: **bsim.geometry** - package bsim.geometry: **bsim.ode** - package bsim.ode: **bsim.particle** - package bsim.particle: **BSimBacterium** - Class in bsim.particle: Class representing a bacterium whose run-tumble motion is affected in a simple way by a single goal chemical. **BSimBacterium(BSim, Vector3d)** - Constructor for class bsim.particle.BSimBacterium: Creates a RUNNING bacterium at the specified position, facing in a random direction **BSimBacterium.MotionState** - Enum in bsim.particle: **BSimChemicalField** - Class in bsim: Standard chemical field (uniform division of space) . **BSimChemicalField(BSim, int[], double, double)** - Constructor for class bsim.BSimChemicalField: Constructor that creates a new chemical field with attached to a particular simulation and with a specified number of boxes, chemical diffusivity and decay rate. **BSimCollision** - Class in bsim.geometry: Collision related methods. **BSimCollision()** - Constructor for class bsim.geometry.BSimCollision: Constructor for an empty collision. **BSimDdeSolver** - Class in bsim.dde: Solver routines for numerical simulation of DDEs (Fixed time-step). **BSimDdeSolver()** - Constructor for class bsim.dde.BSimDdeSolver: **BSimDdeSystem** - Interface in bsim.dde: Interface used for defining a system of DDEs. **BSimDrawer** - Class in bsim.draw: Drawer base class. **BSimDrawer(BSim, int, int)** - Constructor for class bsim.draw.BSimDrawer: Constructor for a drawer. **BSimExporter** - Class in bsim.export: Exporter base class. **BSimExporter(BSim)** - Constructor for class bsim.export.BSimExporter: Constructor of a basic exporter. **BSimLogger** - Class in bsim.export: Text file exporter. **BSimLogger(BSim, String)** - Constructor for class bsim.export.BSimLogger: Constructor for a file logger. **BSimMesh** - Class in bsim.geometry: Abstract 3-D mesh surface class. **BSimMesh()** - Constructor for class bsim.geometry.BSimMesh: Default constructor, initialises the vertex and triangle lists. **BSimMeshUtils** - Class in bsim.geometry: Utility functions for meshes. **BSimMeshUtils()** - Constructor for class bsim.geometry.BSimMeshUtils: **BSimMovExporter** - Class in bsim.export: Movie file exporter. **BSimMovExporter(BSim, BSimDrawer, String)** - Constructor for class bsim.export.BSimMovExporter: Constructor for the movie exporter. **BSimNotifier** - Class in bsim: Notifier used for multi-threaded tickers. **BSimNotifier()** - Constructor for class bsim.BSimNotifier: Constructor of a notifier (no options available). **BSimOBJMesh** - Class in bsim.geometry: Wavefront OBJ importer. **BSimOBJMesh()** - Constructor for class bsim.geometry.BSimOBJMesh: Mesh constructor **BSimOctreeField** - Class in bsim: Octree chemical field (non-uniform division of space). **BSimOctreeField()** - Constructor for class bsim.BSimOctreeField: Constructor for a basic BSimOctreeField (default settings). **BSimOctreeField(Vector3d, double)** - Constructor for class bsim.BSimOctreeField: Root Constructor - only used to make first root Octree Node **BSimOdeSolver** - Class in bsim.ode: Solver routines for numerical simulation of ODEs (Fixed time-step): Euler's method second order Runge-Kutta fourth order Runge-Kutta Each method will estimate the change of the dependent variable based on the previous value of the dependent (y) and independent (x) variables, and return the new value of the dependent variable. The methods are in order of increasing accuracy for a given time-step; Euler's method is the most basic, but the fastest as a result of having to perform relatively few calculations, while the Runge-Kutta methods use an intermediate trial step at the midpoint of an interval to cancel lower order error terms. **BSimOdeSolver()** - Constructor for class bsim.ode.BSimOdeSolver: **BSimOdeSystem** - Interface in bsim.ode: Interface used for defining a system of ODEs. **BSimP3DDrawer** - Class in bsim.draw: Scene preview and visualisation renderer (extends BSimDrawer). **BSimP3DDrawer(BSim, int, int)** - Constructor for class bsim.draw.BSimP3DDrawer: Default constructor for initialising a Processing3D rendering context. **BSimParticle** - Class in bsim.particle: **BSimParticle(BSim, Vector3d, double)** - Constructor for class bsim.particle.BSimParticle: **BSimPngExporter** - Class in bsim.export: Image file exporter. **BSimPngExporter(BSim, BSimDrawer, String)** - Constructor for class bsim.export.BSimPngExporter: Constructor for the image exporter **BSimSphereMesh** - Class in bsim.geometry: Sphere mesh, uses face-vertex representation. **BSimSphereMesh(Vector3d, double, int)** - Constructor for class bsim.geometry.BSimSphereMesh: Main constructor. **BSimThreadedTicker** - Class in bsim: Multi-threaded ticker. **BSimThreadedTicker(int)** - Constructor for class bsim.BSimThreadedTicker: Constructor that creates a threaded ticker that uses a fixed size pool of threads. **BSimThreadedTickerWorker** - Class in bsim: Multi-threaded ticker worker. **BSimThreadedTickerWorker(int, int)** - Constructor for class bsim.BSimThreadedTickerWorker: Constructor to create a new worker for the BSimThreadedTicker. **BSimTicker** - Class in bsim: Standard ticker. **BSimTicker()** - Constructor for class bsim.BSimTicker: **BSimTriangle** - Class in bsim.geometry: Triangular face of a 3-D mesh surface. **BSimTriangle(int, int, int, BSimMesh)** - Constructor for class bsim.geometry.BSimTriangle: Constructor: New triangular face from three individual vertex indices. **BSimTriangle(int[], BSimMesh)** - Constructor for class bsim.geometry.BSimTriangle: Constructor: New triangular face from array of vertex indices. **BSimTriangle(BSimTriangle)** - Constructor for class bsim.geometry.BSimTriangle: **BSimUtils** - Class in bsim: Utility functions. **BSimUtils()** - Constructor for class bsim.BSimUtils: **BSimVertex** - Class in bsim.geometry: Mesh vertex. **BSimVertex(double, double, double)** - Constructor for class bsim.geometry.BSimVertex: Constructor: create a new mesh vertex from three points; x,y,z **BSimVertex(Vector3d)** - Constructor for class bsim.geometry.BSimVertex: Constructor: create a new mesh vertex from a Vector3d **BSimVesicle** - Class in bsim.particle: **BSimVesicle(BSim, Vector3d, double)** - Constructor for class bsim.particle.BSimVesicle: Constructor for a vesicle at a position and of a given size. **bufferedWriter** - Variable in class bsim.export.BSimLogger: Object to write output to.

---


|  |  |  |  |  |  |  |  |  |  |  |
| --- | --- | --- | --- | --- | --- | --- | --- | --- | --- | --- |
| |  |  |  |  |  |  |  |  | | --- | --- | --- | --- | --- | --- | --- | --- | | **Overview** | Package | Class | Use | **Tree** | **Deprecated** | **Index** | **Help** | | |  |
| **PREV LETTER**   **NEXT LETTER** | **FRAMES**    **NO FRAMES**     **All Classes** |


A B C D E F G H I K L M N O P Q R S T U V W X Y Z 

---
