# Supplementary material for: BSim: An Agent-Based Tool for Modeling Bacterial Populations in Systems and Synthetic Biology
Source: PLoS One. 2012 Aug 24;7(8):e42790. doi: 10.1371/journal.pone.0042790 (PMC3427305; doi:10.1371/journal.pone.0042790)
Supplement: Software S1 — Snapshot of the BSim software from 18th July 2012. For the latest version see: http://bsim-bccs.sf.net. The BSim software requires Java version 1.6 or higher. (ZIP) [file pone.0042790.s014.zip › BSimSoftware/docs/javadoc/index-files/index-21.html]

V-Index


---


|  |  |  |  |  |  |  |  |  |  |  |
| --- | --- | --- | --- | --- | --- | --- | --- | --- | --- | --- |
| |  |  |  |  |  |  |  |  | | --- | --- | --- | --- | --- | --- | --- | --- | | **Overview** | Package | Class | Use | **Tree** | **Deprecated** | **Index** | **Help** | | |  |
| **PREV LETTER**   **NEXT LETTER** | **FRAMES**    **NO FRAMES**     **All Classes** |


A B C D E F G H I K L M N O P Q R S T U V W X Y Z 

---


## **V**

**val** - Variable in class bsim.geometry.KdNode.Indexed3d: **valueOf(String)** - Static method in enum bsim.export.quicktime.QuickTimeOutputStream.VideoFormat: Returns the enum constant of this type with the specified name. **valueOf(String)** - Static method in enum bsim.particle.BSimBacterium.MotionState: Returns the enum constant of this type with the specified name. **values()** - Static method in enum bsim.export.quicktime.QuickTimeOutputStream.VideoFormat: Returns an array containing the constants of this enum type, in the order they are declared. **values()** - Static method in enum bsim.particle.BSimBacterium.MotionState: Returns an array containing the constants of this enum type, in the order they are declared. **vecGetCoord(Vector3d, int)** - Static method in class bsim.geometry.KdNode: ...GHH **vector(Vector3d, Vector3d, double, Color)** - Method in class bsim.draw.BSimP3DDrawer: Draw a 'vector' originating at a point, represented by a line. **vertex(Vector3d)** - Method in class bsim.draw.BSimP3DDrawer: Define a p3d vertex when constructing shapes, directly from a Point3d. **vertices** - Variable in class bsim.geometry.BSimMesh: The actual locations (3D coordinates) of all mesh vertices **vesicleList** - Variable in class bsim.particle.BSimBacterium: The external list of vesicles. **vesicleRadius** - Variable in class bsim.particle.BSimBacterium: **vesicleRadius()** - Method in class bsim.particle.BSimBacterium: **vesicleRadius(double)** - Method in class bsim.particle.BSimBacterium: **vesiculate()** - Method in class bsim.particle.BSimBacterium: **visit(BSimOctreeField)** - Method in class bsim.BSimOctreeField: The visit method simply prints the location and depth of a node, useful for troubleshooting. **volume** - Variable in class bsim.BSimOctreeField: Volume of node (simply length^3).

---


|  |  |  |  |  |  |  |  |  |  |  |
| --- | --- | --- | --- | --- | --- | --- | --- | --- | --- | --- |
| |  |  |  |  |  |  |  |  | | --- | --- | --- | --- | --- | --- | --- | --- | | **Overview** | Package | Class | Use | **Tree** | **Deprecated** | **Index** | **Help** | | |  |
| **PREV LETTER**   **NEXT LETTER** | **FRAMES**    **NO FRAMES**     **All Classes** |


A B C D E F G H I K L M N O P Q R S T U V W X Y Z 

---
