# Supplementary material for: BSim: An Agent-Based Tool for Modeling Bacterial Populations in Systems and Synthetic Biology
Source: PLoS One. 2012 Aug 24;7(8):e42790. doi: 10.1371/journal.pone.0042790 (PMC3427305; doi:10.1371/journal.pone.0042790)
Supplement: Software S1 — Snapshot of the BSim software from 18th July 2012. For the latest version see: http://bsim-bccs.sf.net. The BSim software requires Java version 1.6 or higher. (ZIP) [file pone.0042790.s014.zip › BSimSoftware/docs/javadoc/index-files/index-22.html]

W-Index


---


|  |  |  |  |  |  |  |  |  |  |  |
| --- | --- | --- | --- | --- | --- | --- | --- | --- | --- | --- |
| |  |  |  |  |  |  |  |  | | --- | --- | --- | --- | --- | --- | --- | --- | | **Overview** | Package | Class | Use | **Tree** | **Deprecated** | **Index** | **Help** | | |  |
| **PREV LETTER**   **NEXT LETTER** | **FRAMES**    **NO FRAMES**     **All Classes** |


A B C D E F G H I K L M N O P Q R S T U V W X Y Z 

---


## **W**

**waitForNotify()** - Method in class bsim.BSimNotifier: Places object that calls this method in wait cycle. **width** - Variable in class bsim.draw.BSimDrawer: Width of the display (pixels). **workers** - Variable in class bsim.BSimThreadedTicker: List of workers to call upon. **wrapAbove(double, double)** - Method in class bsim.particle.BSimParticle: **wrapBelow(double, double)** - Method in class bsim.particle.BSimParticle: **write(String)** - Method in class bsim.export.BSimLogger: Writes text to the output file. **write(byte[], int, int)** - Method in class bsim.export.quicktime.AtomDataOutputStream: Writes `len` bytes from the specified byte array starting at offset `off` to the underlying output stream. **write(int)** - Method in class bsim.export.quicktime.AtomDataOutputStream: Writes the specified byte (the low eight bits of the argument `b`) to the underlying output stream. **write(int)** - Method in class bsim.export.quicktime.FilterImageOutputStream: Writes the specified `byte` to this output stream. **write(byte[], int, int)** - Method in class bsim.export.quicktime.FilterImageOutputStream: Writes `len` bytes from the specified `byte` array starting at offset `off` to this output stream. **writeBCD2(int)** - Method in class bsim.export.quicktime.AtomDataOutputStream: Writes a `BCD2` to the underlying output stream. **writeBCD4(int)** - Method in class bsim.export.quicktime.AtomDataOutputStream: Writes a `BCD4` to the underlying output stream. **writeByte(int)** - Method in class bsim.export.quicktime.AtomDataOutputStream: Writes out a `byte` to the underlying output stream as a 1-byte value. **writeFixed16D16(double)** - Method in class bsim.export.quicktime.AtomDataOutputStream: Writes 32-bit fixed-point number divided as 16.16. **writeFixed2D30(double)** - Method in class bsim.export.quicktime.AtomDataOutputStream: Writes 32-bit fixed-point number divided as 2.30. **writeFixed8D8(float)** - Method in class bsim.export.quicktime.AtomDataOutputStream: Writes 16-bit fixed-point number divided as 8.8. **writeFrame(BufferedImage, int)** - Method in class bsim.export.quicktime.QuickTimeOutputStream: Writes a frame to the video track. **writeFrame(File, int)** - Method in class bsim.export.quicktime.QuickTimeOutputStream: Writes a frame from a file to the video track. **writeFrame(InputStream, int)** - Method in class bsim.export.quicktime.QuickTimeOutputStream: Writes a frame to the video track. **writeInt(int)** - Method in class bsim.export.quicktime.AtomDataOutputStream: Writes an `int` to the underlying output stream as four bytes, high byte first. **writeLong(long)** - Method in class bsim.export.quicktime.AtomDataOutputStream: **writeMacTimestamp(Date)** - Method in class bsim.export.quicktime.AtomDataOutputStream: Writes a 32-bit Mac timestamp (seconds since 1902). **writePString(String)** - Method in class bsim.export.quicktime.AtomDataOutputStream: Writes a Pascal String. **writeShort(int)** - Method in class bsim.export.quicktime.AtomDataOutputStream: Writes a signed 16 bit integer value. **writeType(String)** - Method in class bsim.export.quicktime.AtomDataOutputStream: Writes an Atom Type identifier (4 bytes). **writeUInt(long)** - Method in class bsim.export.quicktime.AtomDataOutputStream: Writes an unsigned 32 bit integer value. **writeUShort(int)** - Method in class bsim.export.quicktime.AtomDataOutputStream: **written** - Variable in class bsim.export.quicktime.AtomDataOutputStream: The number of bytes written to the data output stream so far.

---


|  |  |  |  |  |  |  |  |  |  |  |
| --- | --- | --- | --- | --- | --- | --- | --- | --- | --- | --- |
| |  |  |  |  |  |  |  |  | | --- | --- | --- | --- | --- | --- | --- | --- | | **Overview** | Package | Class | Use | **Tree** | **Deprecated** | **Index** | **Help** | | |  |
| **PREV LETTER**   **NEXT LETTER** | **FRAMES**    **NO FRAMES**     **All Classes** |


A B C D E F G H I K L M N O P Q R S T U V W X Y Z 

---
