# Supplementary material for: BSim: An Agent-Based Tool for Modeling Bacterial Populations in Systems and Synthetic Biology
Source: PLoS One. 2012 Aug 24;7(8):e42790. doi: 10.1371/journal.pone.0042790 (PMC3427305; doi:10.1371/journal.pone.0042790)
Supplement: Software S1 — Snapshot of the BSim software from 18th July 2012. For the latest version see: http://bsim-bccs.sf.net. The BSim software requires Java version 1.6 or higher. (ZIP) [file pone.0042790.s014.zip › BSimSoftware/docs/javadoc/index-files/index-3.html]

C-Index


---


|  |  |  |  |  |  |  |  |  |  |  |
| --- | --- | --- | --- | --- | --- | --- | --- | --- | --- | --- |
| |  |  |  |  |  |  |  |  | | --- | --- | --- | --- | --- | --- | --- | --- | | **Overview** | Package | Class | Use | **Tree** | **Deprecated** | **Index** | **Help** | | |  |
| **PREV LETTER**   **NEXT LETTER** | **FRAMES**    **NO FRAMES**     **All Classes** |


A B C D E F G H I K L M N O P Q R S T U V W X Y Z 

---


## **C**

**calcVertexFaces()** - Method in class bsim.geometry.BSimMesh: Compute which faces index each vertex, and store this as a list parameter in each vertex object. **centre** - Variable in class bsim.BSimOctreeField: Location of centre of node in space. **childList** - Variable in class bsim.particle.BSimBacterium: The external list of children. **cleanUp(boolean)** - Method in class bsim.geometry.BSimMesh: Trim down the arrayLists, and compute vertex-face connectivity. **close()** - Method in class bsim.export.quicktime.FilterImageOutputStream: Closes this output stream and releases any system resources associated with the stream. **close()** - Method in class bsim.export.quicktime.QuickTimeOutputStream: Closes the movie file as well as the stream being filtered. **closestPtPointTriangle(Vector3d, Vector3d, Vector3d, Vector3d)** - Static method in class bsim.geometry.BSimMeshUtils: Closest point on triangle to a given point in 3d space. **collideAndCross(Vector3d, Vector3d, BSimMesh)** - Static method in class bsim.geometry.BSimCollision: Check if mesh is crossed. **collideAndReflect(Vector3d, Vector3d, BSimMesh)** - Static method in class bsim.geometry.BSimCollision: Check to see if intersection with mesh and reflect. **collideAndRepel(BSimParticle, BSimMesh)** - Static method in class bsim.geometry.BSimCollision: Check for collision between particle and mesh, and add repulsion force. **colorFromCentre(BSimOctreeField)** - Method in class bsim.BSimOctreeField: Sets the nodeColor value as a function of the position of octree, useful for troubleshooting. **colorFromConc()** - Method in class bsim.BSimOctreeField: Sets nodeCololr value as a function of amount of chemical in box. **computeNormal(BSimTriangle)** - Method in class bsim.geometry.BSimMesh: Compute the normal vector of a face. **computeNormals()** - Method in class bsim.geometry.BSimMesh: Compute all normals of the mesh **createMesh()** - Method in class bsim.geometry.BSimMesh: Abstract method in which the vertices and faces of the mesh should be defined. **createMesh()** - Method in class bsim.geometry.BSimOBJMesh: **createMesh()** - Method in class bsim.geometry.BSimSphereMesh: Defines the vertices and faces for a geodesic sphere. **createMesh()** - Method in class bsim.geometry.KdNode.TestMesh: **createWorker(int, int)** - Method in class bsim.BSimThreadedTicker: For the user to overwrite to create suitable workers for this ticker.

---


|  |  |  |  |  |  |  |  |  |  |  |
| --- | --- | --- | --- | --- | --- | --- | --- | --- | --- | --- |
| |  |  |  |  |  |  |  |  | | --- | --- | --- | --- | --- | --- | --- | --- | | **Overview** | Package | Class | Use | **Tree** | **Deprecated** | **Index** | **Help** | | |  |
| **PREV LETTER**   **NEXT LETTER** | **FRAMES**    **NO FRAMES**     **All Classes** |


A B C D E F G H I K L M N O P Q R S T U V W X Y Z 

---
