# Supplementary material for: BSim: An Agent-Based Tool for Modeling Bacterial Populations in Systems and Synthetic Biology
Source: PLoS One. 2012 Aug 24;7(8):e42790. doi: 10.1371/journal.pone.0042790 (PMC3427305; doi:10.1371/journal.pone.0042790)
Supplement: Software S1 — Snapshot of the BSim software from 18th July 2012. For the latest version see: http://bsim-bccs.sf.net. The BSim software requires Java version 1.6 or higher. (ZIP) [file pone.0042790.s014.zip › BSimSoftware/docs/javadoc/index-files/index-4.html]

D-Index


---


|  |  |  |  |  |  |  |  |  |  |  |
| --- | --- | --- | --- | --- | --- | --- | --- | --- | --- | --- |
| |  |  |  |  |  |  |  |  | | --- | --- | --- | --- | --- | --- | --- | --- | | **Overview** | Package | Class | Use | **Tree** | **Deprecated** | **Index** | **Help** | | |  |
| **PREV LETTER**   **NEXT LETTER** | **FRAMES**    **NO FRAMES**     **All Classes** |


A B C D E F G H I K L M N O P Q R S T U V W X Y Z 

---


## **D**

**decay()** - Method in class bsim.BSimChemicalField: Decay the chemical present in the field. **decay(BSimOctreeField, double, double)** - Method in class bsim.BSimOctreeField: Decays the chemical field in an octreeField Node,visits each node in the tree structure using a post-order traverse. **decayRate** - Variable in class bsim.BSimChemicalField: Fraction of chemical decaying per second, quantity(t+dt) = quantity(t)\*(1-decayRate\*dt). **depth** - Variable in class bsim.BSimOctreeField: Depth in octree structure, root has depth 0. **derivativeSystem(double, double[], Vector<double[]>)** - Method in interface bsim.dde.BSimDdeSystem: Defines a system of derivatives dy[0] = ..., dy[1] = ..., etc and returns dy[] **derivativeSystem(double, double[])** - Method in interface bsim.ode.BSimOdeSystem: Define a system of derivatives - dy[0] = ..., dy[1] = ..., etc then return dy[] **diffuse()** - Method in class bsim.BSimChemicalField: Diffuse the chemical present in the field. **diffuse(BSimOctreeField, double, double, int)** - Method in class bsim.BSimOctreeField: Diffuses chemicals through whole the octreeField structure, using Fick's law to determine how much of the chemical gets pushed into neighboring nodes over each time iteration **diffusivity** - Variable in class bsim.BSimChemicalField: Diffusivity of the chemical field. **diffusivity** - Variable in class bsim.BSimOctreeField: Diffusivity of node. **direction** - Variable in class bsim.particle.BSimBacterium: Direction that the cell exerts its flagellar force. **directory** - Variable in class bsim.export.BSimPngExporter: Directory to output images to. **distance(BSimParticle)** - Method in class bsim.particle.BSimParticle: Distance between particle centres (always positive) **draw(Graphics2D)** - Method in class bsim.draw.BSimDrawer: Abstract method to draw the simulation to a graphics context (to be overwritten by the user). **draw(Graphics2D)** - Method in class bsim.draw.BSimP3DDrawer: Render all simulation and scene elements to the Processing3D graphics context 'p3d' (effectively the render buffer), then draw the rendered contents to the native Java graphics context. **draw(BSimParticle, Color)** - Method in class bsim.draw.BSimP3DDrawer: Draw a BSimParticle as a point if it is very small (radius < 1), or a sphere otherwise. **draw(BSimVesicle, Color)** - Method in class bsim.draw.BSimP3DDrawer: Draw a vesicle as a pixel surrounded by a 'halo' to make it easier to spot. **draw(BSimMesh, Color, double)** - Method in class bsim.draw.BSimP3DDrawer: Draw a mesh with a given colour (draws each triangle of the mesh individually). **draw(BSimMesh, double)** - Method in class bsim.draw.BSimP3DDrawer: Draw a mesh, default colour. **draw(BSimChemicalField, Color, double, double)** - Method in class bsim.draw.BSimP3DDrawer: Draws a chemical field structure based on its defined parameters, with custom transparency (alpha) parameters. **draw(BSimChemicalField, Color, float)** - Method in class bsim.draw.BSimP3DDrawer: Draw a chemical field structure based on its defined parameters (default alpha). **draw(BSimOctreeField, Color, float)** - Method in class bsim.draw.BSimP3DDrawer: Draw a BSimOctreeField in given colour. **draw(BSimOctreeField, float)** - Method in class bsim.draw.BSimP3DDrawer: Draw a BSimOctreeField. **drawer** - Variable in class bsim.export.BSimMovExporter: Drawer to generate a movie frame. **drawer** - Variable in class bsim.export.BSimPngExporter: Drawer to generate each image. **dt** - Variable in class bsim.export.BSimExporter: Timestp. **during()** - Method in class bsim.export.BSimExporter: Called each timestep (overwrite). **during()** - Method in class bsim.export.BSimMovExporter: Called at each timestep of the simulation. **during()** - Method in class bsim.export.BSimPngExporter: Called at each timestep of the simulation.

---


|  |  |  |  |  |  |  |  |  |  |  |
| --- | --- | --- | --- | --- | --- | --- | --- | --- | --- | --- |
| |  |  |  |  |  |  |  |  | | --- | --- | --- | --- | --- | --- | --- | --- | | **Overview** | Package | Class | Use | **Tree** | **Deprecated** | **Index** | **Help** | | |  |
| **PREV LETTER**   **NEXT LETTER** | **FRAMES**    **NO FRAMES**     **All Classes** |


A B C D E F G H I K L M N O P Q R S T U V W X Y Z 

---
