# Supplementary material for: BSim: An Agent-Based Tool for Modeling Bacterial Populations in Systems and Synthetic Biology
Source: PLoS One. 2012 Aug 24;7(8):e42790. doi: 10.1371/journal.pone.0042790 (PMC3427305; doi:10.1371/journal.pone.0042790)
Supplement: Software S1 — Snapshot of the BSim software from 18th July 2012. For the latest version see: http://bsim-bccs.sf.net. The BSim software requires Java version 1.6 or higher. (ZIP) [file pone.0042790.s014.zip › BSimSoftware/docs/javadoc/index-files/index-6.html]

F-Index


---


|  |  |  |  |  |  |  |  |  |  |  |
| --- | --- | --- | --- | --- | --- | --- | --- | --- | --- | --- |
| |  |  |  |  |  |  |  |  | | --- | --- | --- | --- | --- | --- | --- | --- | | **Overview** | Package | Class | Use | **Tree** | **Deprecated** | **Index** | **Help** | | |  |
| **PREV LETTER**   **NEXT LETTER** | **FRAMES**    **NO FRAMES**     **All Classes** |


A B C D E F G H I K L M N O P Q R S T U V W X Y Z 

---


## **F**

**faces** - Variable in class bsim.geometry.BSimMesh: List of faces, each face stores the indices of the vertices which compose that face. **faces** - Variable in class bsim.geometry.BSimVertex: List of the indices of the faces which use this vertex. **filename** - Variable in class bsim.export.BSimLogger: Filename of output. **filename** - Variable in class bsim.export.BSimMovExporter: Filename of the output movie. **FilterImageOutputStream** - Class in bsim.export.quicktime: FilterImageOutputStream adapts a ImageOutputStream to the FilterOutputStream interface. **FilterImageOutputStream(ImageOutputStream)** - Constructor for class bsim.export.quicktime.FilterImageOutputStream: **findMedian(KdNode.Indexed3d[], int, int, int, int)** - Method in class bsim.geometry.KdNode: **finish()** - Method in class bsim.export.quicktime.QuickTimeOutputStream: Finishes writing the contents of the QuickTime output stream without closing the underlying stream. **flagellarForce()** - Method in class bsim.particle.BSimBacterium: Applies the flagellar force. **flipNormal()** - Method in class bsim.geometry.BSimTriangle: Flip the face normal if you want it to point the other way. **flipNormals()** - Method in class bsim.geometry.BSimMesh: Flip normals of all faces **flipNormals(int[])** - Method in class bsim.geometry.BSimMesh: Flip normals of selected faces **flush()** - Method in class bsim.export.quicktime.FilterImageOutputStream: Flushes this output stream and forces any buffered output bytes to be written out to the stream. **font** - Variable in class bsim.draw.BSimP3DDrawer: Font used when rendering text. **force** - Variable in class bsim.particle.BSimParticle: **forceMagnitude** - Variable in class bsim.particle.BSimBacterium: Magnitude of the flagellar force produced by the cell whilst RUNNING.

---


|  |  |  |  |  |  |  |  |  |  |  |
| --- | --- | --- | --- | --- | --- | --- | --- | --- | --- | --- |
| |  |  |  |  |  |  |  |  | | --- | --- | --- | --- | --- | --- | --- | --- | | **Overview** | Package | Class | Use | **Tree** | **Deprecated** | **Index** | **Help** | | |  |
| **PREV LETTER**   **NEXT LETTER** | **FRAMES**    **NO FRAMES**     **All Classes** |


A B C D E F G H I K L M N O P Q R S T U V W X Y Z 

---
