# Supplementary material for: BSim: An Agent-Based Tool for Modeling Bacterial Populations in Systems and Synthetic Biology
Source: PLoS One. 2012 Aug 24;7(8):e42790. doi: 10.1371/journal.pone.0042790 (PMC3427305; doi:10.1371/journal.pone.0042790)
Supplement: Software S1 — Snapshot of the BSim software from 18th July 2012. For the latest version see: http://bsim-bccs.sf.net. The BSim software requires Java version 1.6 or higher. (ZIP) [file pone.0042790.s014.zip › BSimSoftware/docs/javadoc/index-files/index-7.html]

G-Index


---


|  |  |  |  |  |  |  |  |  |  |  |
| --- | --- | --- | --- | --- | --- | --- | --- | --- | --- | --- |
| |  |  |  |  |  |  |  |  | | --- | --- | --- | --- | --- | --- | --- | --- | | **Overview** | Package | Class | Use | **Tree** | **Deprecated** | **Index** | **Help** | | |  |
| **PREV LETTER**   **NEXT LETTER** | **FRAMES**    **NO FRAMES**     **All Classes** |


A B C D E F G H I K L M N O P Q R S T U V W X Y Z 

---


## **G**

**generateDirectoryPath(String)** - Static method in class bsim.BSimUtils: Checks the existence of a desired path. **getBound()** - Method in class bsim.BSim: Return the simulation bounds (microns). **getBox()** - Method in class bsim.BSimChemicalField: Return the size of each box (x,y,z) in microns. **getBoxes()** - Method in class bsim.BSimChemicalField: Return the number of boxes in (x,y,z) directions. **getCentre()** - Method in class bsim.BSimOctreeField: Return the centre. **getConc(Vector3d)** - Method in class bsim.BSimChemicalField: Gets the concentration of the field at the position v in molecules/(micron)^3. **getConc(int, int, int)** - Method in class bsim.BSimChemicalField: Gets the concentration of the field in the box (x,y,z) in molecules/(micron)^3. **getDelayedState(Vector<double[]>, double, double)** - Static method in class bsim.dde.BSimDdeSolver: Calculates a delayed (historic) state. **getDepth()** - Method in class bsim.BSimOctreeField: Return the depth. **getDiffusivity()** - Method in class bsim.BSimOctreeField: Return the diffusivity. **getDirection()** - Method in class bsim.particle.BSimBacterium: **getDt()** - Method in class bsim.BSim: Return the timestep. **getDt()** - Method in class bsim.export.BSimExporter: Return the timestep. **getFace(int)** - Method in class bsim.geometry.BSimMesh: **getFaces()** - Method in class bsim.geometry.BSimMesh: **getFaces()** - Method in class bsim.geometry.BSimVertex: **getForce()** - Method in class bsim.particle.BSimParticle: **getFormattedTime()** - Method in class bsim.BSim: Return a formatted version of the current time of the simulation. **getHeight()** - Method in class bsim.draw.BSimDrawer: Return the height of the display (pixels). **getICs()** - Method in interface bsim.dde.BSimDdeSystem: Get the initial conditions: y1(0), y2(0), etc.. **getICs()** - Method in interface bsim.ode.BSimOdeSystem: Get the initial conditions: y1(0), y2(0), etc.. **getInitialState(BSimDdeSystem, double)** - Static method in class bsim.dde.BSimDdeSolver: Create an initial state, including history. **getLeaky()** - Method in class bsim.BSim: Return whether the boundaries are leaky. **getLeakyRate()** - Method in class bsim.BSim: Return the rate that chemicals can escape from the simulation (if the boundary is leaky). **getLength()** - Method in class bsim.BSimOctreeField: Return the length. **getLocation()** - Method in class bsim.geometry.BSimCollision: Return location of collision. **getLocation()** - Method in class bsim.geometry.BSimVertex: **getMaxDelay()** - Method in interface bsim.dde.BSimDdeSystem: Get the maximum delay for the system **getMemoryDuration()** - Method in class bsim.particle.BSimBacterium: **getMiddle(int, int)** - Method in class bsim.geometry.BSimSphereMesh: Create the middle vertex between two vertices if it doesn't already exist. **getMotionState()** - Method in class bsim.particle.BSimBacterium: **getnodeColor()** - Method in class bsim.BSimOctreeField: Return the node colour. **getNormal()** - Method in class bsim.geometry.BSimTriangle: Gets the triangle normal vector. **getNumEq()** - Method in interface bsim.dde.BSimDdeSystem: Get the number of equations in the system (Corresponding to the number in derivativeSystem) **getNumEq()** - Method in interface bsim.ode.BSimOdeSystem: Get the number of equations in the system (Corresponding to the number in derivativeSystem) **getP1()** - Method in class bsim.geometry.BSimTriangle: **getP2()** - Method in class bsim.geometry.BSimTriangle: **getP3()** - Method in class bsim.geometry.BSimTriangle: **getParentMesh()** - Method in class bsim.geometry.BSimTriangle: **getPoints()** - Method in class bsim.geometry.BSimTriangle: **getPosition()** - Method in class bsim.particle.BSimParticle: **getQuantity()** - Method in class bsim.BSimOctreeField: Return the chemical quantity. **getRadius()** - Method in class bsim.particle.BSimParticle: **getSimulationTime()** - Method in class bsim.BSim: Return the length of the simulation. **getSolid()** - Method in class bsim.BSim: Return whether the boundaries are solid (reflecting) or wrapping (periiodic). **getsubNode(int)** - Method in class bsim.BSimOctreeField: Return the subNode (i is index of subnode). **getSubSet(KdNode.Indexed3d[], int, int)** - Method in class bsim.geometry.KdNode: **getSurfaceArea()** - Method in class bsim.particle.BSimParticle: **getTCentre(BSimTriangle)** - Method in class bsim.geometry.BSimMesh: Compute the coordinates of the centre of a triangle **getTemperature()** - Method in class bsim.BSim: Return the temperature of the environment. **getTime()** - Method in class bsim.BSim: Return the current time of the simulation. **getTimeScale()** - Method in class bsim.export.quicktime.QuickTimeOutputStream: Returns the time scale of this media. **getTimeStamp()** - Method in class bsim.geometry.BSimTriangle: **getTimestep()** - Method in class bsim.BSim: Return the current timestep of the simulation. **getTVal()** - Method in class bsim.geometry.BSimCollision: Return the t value. **getVertCoords(int)** - Method in class bsim.geometry.BSimMesh: **getVertCoords(int)** - Method in class bsim.geometry.BSimTriangle: Get the vertex coordinates of a given triangle **getVertCoordsOfTri(BSimTriangle, int)** - Method in class bsim.geometry.BSimMesh: Get the vertex coordinates of a given triangle **getVertex(int)** - Method in class bsim.geometry.BSimMesh: **getVertices()** - Method in class bsim.geometry.BSimMesh: **getVideoCompressionQuality()** - Method in class bsim.export.quicktime.QuickTimeOutputStream: Returns the video compression quality. **getVisc()** - Method in class bsim.BSim: Return the viscosity of the environment. **getWidth()** - Method in class bsim.draw.BSimDrawer: Return the width of the display (pixels). **goal** - Variable in class bsim.particle.BSimBacterium: Bacteria tend to swim towards higher concentrations of this chemical field. **grow()** - Method in class bsim.particle.BSimBacterium

---


|  |  |  |  |  |  |  |  |  |  |  |
| --- | --- | --- | --- | --- | --- | --- | --- | --- | --- | --- |
| |  |  |  |  |  |  |  |  | | --- | --- | --- | --- | --- | --- | --- | --- | | **Overview** | Package | Class | Use | **Tree** | **Deprecated** | **Index** | **Help** | | |  |
| **PREV LETTER**   **NEXT LETTER** | **FRAMES**    **NO FRAMES**     **All Classes** |


A B C D E F G H I K L M N O P Q R S T U V W X Y Z 

---
