# Supplementary material for: BSim: An Agent-Based Tool for Modeling Bacterial Populations in Systems and Synthetic Biology
Source: PLoS One. 2012 Aug 24;7(8):e42790. doi: 10.1371/journal.pone.0042790 (PMC3427305; doi:10.1371/journal.pone.0042790)
Supplement: Software S1 — Snapshot of the BSim software from 18th July 2012. For the latest version see: http://bsim-bccs.sf.net. The BSim software requires Java version 1.6 or higher. (ZIP) [file pone.0042790.s014.zip › BSimSoftware/docs/javadoc/index-files/index-9.html]

I-Index


---


|  |  |  |  |  |  |  |  |  |  |  |
| --- | --- | --- | --- | --- | --- | --- | --- | --- | --- | --- |
| |  |  |  |  |  |  |  |  | | --- | --- | --- | --- | --- | --- | --- | --- | | **Overview** | Package | Class | Use | **Tree** | **Deprecated** | **Index** | **Help** | | |  |
| **PREV LETTER**   **NEXT LETTER** | **FRAMES**    **NO FRAMES**     **All Classes** |


A B C D E F G H I K L M N O P Q R S T U V W X Y Z 

---


## **I**

**incCount(int)** - Method in class bsim.export.quicktime.AtomDataOutputStream: Increases the written counter by the specified value until it reaches Long.MAX\_VALUE. **inOrderfull(BSimOctreeField)** - Static method in class bsim.BSimOctreeField: In-Order traverse, traverses from the deepest subnode, to the root and then back down to other deep nodes. **intersection(Vector)** - Method in class bsim.particle.BSimParticle: Tests if this particle is intersecting with any in the vector **intersectPlaneAAB(Vector3d, double, Vector3d, Vector3d)** - Static method in class bsim.geometry.BSimMeshUtils: Test for intersection between a plane and an axis-aligned box. **intersectSpherePlane(BSimParticle, Vector3d, Vector3d)** - Static method in class bsim.geometry.BSimMeshUtils: Computes the intersection of a sphere with a plane **intersectSphereTriangle(BSimParticle, Vector3d, Vector3d, Vector3d, Vector3d)** - Static method in class bsim.geometry.BSimMeshUtils: Intersection of a sphere with a triangle **intersectTriangleAAB(BSimTriangle, Vector3d, Vector3d)** - Static method in class bsim.geometry.BSimMeshUtils: Test for intersection of a triangle against an axis aligned box **intersectTriOctreeNode(BSimTriangle, BSimOctreeField)** - Static method in class bsim.geometry.BSimMeshUtils: Test for intersection of a triangle against an octree node **intersectVectorKdNode(Vector3d, Vector3d, KdNode)** - Static method in class bsim.geometry.KdNode: Intersect a direction vector segment with KdNode (or hierarchy) **intersectVectorPlane(Vector3d, Vector3d, Vector3d, double)** - Static method in class bsim.geometry.BSimMeshUtils: Compute the intersection of a vector p1 + t\*dir (line segment) and a plane **intersectVectorTriangle(Vector3d, Vector3d, BSimTriangle)** - Static method in class bsim.BSimOctreeField: **intersectVectorTriangle(Vector3d, Vector3d, BSimTriangle, BSimCollision)** - Static method in class bsim.geometry.BSimMeshUtils: Computes intersection of a vector in 3d space (e.g.

---


|  |  |  |  |  |  |  |  |  |  |  |
| --- | --- | --- | --- | --- | --- | --- | --- | --- | --- | --- |
| |  |  |  |  |  |  |  |  | | --- | --- | --- | --- | --- | --- | --- | --- | | **Overview** | Package | Class | Use | **Tree** | **Deprecated** | **Index** | **Help** | | |  |
| **PREV LETTER**   **NEXT LETTER** | **FRAMES**    **NO FRAMES**     **All Classes** |


A B C D E F G H I K L M N O P Q R S T U V W X Y Z 

---
