# Supplementary material for: BSim: An Agent-Based Tool for Modeling Bacterial Populations in Systems and Synthetic Biology
Source: PLoS One. 2012 Aug 24;7(8):e42790. doi: 10.1371/journal.pone.0042790 (PMC3427305; doi:10.1371/journal.pone.0042790)
Supplement: Software S1 — Snapshot of the BSim software from 18th July 2012. For the latest version see: http://bsim-bccs.sf.net. The BSim software requires Java version 1.6 or higher. (ZIP) [file pone.0042790.s014.zip › BSimSoftware/docs/javadoc/overview-tree.html]

Class Hierarchy


---


|  |  |  |  |  |  |  |  |  |  |  |
| --- | --- | --- | --- | --- | --- | --- | --- | --- | --- | --- |
| |  |  |  |  |  |  |  |  | | --- | --- | --- | --- | --- | --- | --- | --- | | **Overview** | Package | Class | Use | **Tree** | **Deprecated** | **Index** | **Help** | | |  |
| PREV   NEXT | **FRAMES**    **NO FRAMES**     **All Classes** |


---


## Hierarchy For All Packages

**Package Hierarchies:**: bsim, bsim.dde, bsim.draw, bsim.export, bsim.export.quicktime, bsim.geometry, bsim.ode, bsim.particle

---

## Class Hierarchy

- java.lang.Object
  - bsim.**BSim**- bsim.**BSimChemicalField**- bsim.geometry.**BSimCollision**- bsim.dde.**BSimDdeSolver**- bsim.draw.**BSimDrawer**
            - bsim.draw.**BSimP3DDrawer**- bsim.export.**BSimExporter**
              - bsim.export.**BSimLogger**- bsim.export.**BSimMovExporter**- bsim.export.**BSimPngExporter**- bsim.geometry.**BSimMesh**
                - bsim.geometry.**BSimOBJMesh**- bsim.geometry.**BSimSphereMesh**- bsim.geometry.**KdNode.TestMesh**- bsim.geometry.**BSimMeshUtils**- bsim.**BSimNotifier**- bsim.**BSimOctreeField**- bsim.ode.**BSimOdeSolver**- bsim.particle.**BSimParticle**
                          - bsim.particle.**BSimBacterium**- bsim.particle.**BSimVesicle**- bsim.**BSimThreadedTickerWorker** (implements java.lang.Runnable)- bsim.**BSimTicker**
                              - bsim.**BSimThreadedTicker**- bsim.geometry.**BSimTriangle**- bsim.**BSimUtils**- bsim.geometry.**BSimVertex**- bsim.geometry.**KdNode**- bsim.geometry.**KdNode.Indexed3d**- java.io.OutputStream (implements java.io.Closeable, java.io.Flushable)
                                          - java.io.FilterOutputStream
                                            - bsim.export.quicktime.**AtomDataOutputStream**- bsim.export.quicktime.**FilterImageOutputStream**- bsim.export.quicktime.**QuickTimeOutputStream**

## Interface Hierarchy

- bsim.dde.**BSimDdeSystem**- bsim.ode.**BSimOdeSystem**

## Enum Hierarchy

- java.lang.Object
  - java.lang.Enum<E> (implements java.lang.Comparable<T>, java.io.Serializable)
    - bsim.export.quicktime.**QuickTimeOutputStream.VideoFormat**- bsim.particle.**BSimBacterium.MotionState**

---


|  |  |  |  |  |  |  |  |  |  |  |
| --- | --- | --- | --- | --- | --- | --- | --- | --- | --- | --- |
| |  |  |  |  |  |  |  |  | | --- | --- | --- | --- | --- | --- | --- | --- | | **Overview** | Package | Class | Use | **Tree** | **Deprecated** | **Index** | **Help** | | |  |
| PREV   NEXT | **FRAMES**    **NO FRAMES**     **All Classes** |


---
