# Supplementary material for: BSim: An Agent-Based Tool for Modeling Bacterial Populations in Systems and Synthetic Biology
Source: PLoS One. 2012 Aug 24;7(8):e42790. doi: 10.1371/journal.pone.0042790 (PMC3427305; doi:10.1371/journal.pone.0042790)
Supplement: Software S1 — Snapshot of the BSim software from 18th July 2012. For the latest version see: http://bsim-bccs.sf.net. The BSim software requires Java version 1.6 or higher. (ZIP) [file pone.0042790.s014.zip › BSimSoftware/docs/userguide/index.html]

BSim User Guide


# User Guide

## Table of Contents

- **BSim in Action**

- Initialisation
- The Ticker
- Drawers
- Exporters
- Starting the Simulation

- **Features**

- Force-Based Dynamics

- Brownian Motion

- Boundary Types
- Multiple Particles
- Custom Particle Behaviours
- Particle Interactions

- Collisions

- Reaction Forces
- Delivery

- Chemical Fields
- Bacteria

- Motility

- Rotational Diffusion
- Chemotaxis

- Growth

- Replication
- Vesiculation

- ODE Solvers
- DDE Solvers
- Meshes

- Mesh Collisions
- Spatial Varying Parameters

- **References**

## BSim in Action

As a way of getting to grips with BSim, we shall go through a simulation that features a single grain of pollen moving with Brownian motion:
`public class BSimPollenExample {  
   public static void main(String [] args) {  
  
   BSim sim = new BSim();  
  
   class PollenGrain extends BSimParticle {  
      public PollenGrain(BSim sim, Vector3d position, double radius) {  
         super(sim, position, radius);  
      }  
   }  
   final PollenGrain grain = new PollenGrain(sim, new Vector3d(50, 50, 50), 1);  
  
   sim.setTicker(new BSimTicker() {  
  
      public void tick() {  
         grain.action();  
         grain.updatePosition();  
      }  
  
   });  
  
   sim.setDrawer(new BSimP3DDrawer(sim, 800, 600) {  
      public void scene(PGraphics3D p3d) {  
         draw(grain, Color.YELLOW);  
      }  
   });  
  
   sim.addExporter(new BSimLogger(sim, "position.csv") {  
      public void before() {  
         super.before();  
         write("time, x, y, z");  
      }  
      public void during() {  
         write(sim.getFormattedTime() + ", "  
              + grain.getPosition().x + ", "  
              + grain.getPosition().y + ", "  
              + grain.getPosition().z + ", ");  
      }  
   });  
  
   sim.preview();  
  
   }  
}`

### Initialisation

The first thing to notice is that BSim simulations are standard Java programs, complete with a main() method. A BSim is created by instantiating an instance of the BSim class:
`BSim sim = new BSim();`

Without further modification, this consists of an empty fluid-filled cube of side 100 μm with wrapping boundaries. The size, boundary type (solid or wrapping), viscosity and temperature of the environment can all be customised at this stage.

The basic agent in BSim is a BSimParticle, and all other particle types extend this (abstract) class. For example, the BSimBacterium class extends BSimParticle with a variety of bacterial behaviours, including motility, chemotaxis, growth, vesiculation and replication. Here we define PollenGrain as a trivial extension of the BSimParticle class, adding no new methods:
`class PollenGrain extends BSimParticle {  
  
   public PollenGrain(BSim sim, Vector3d position, double radius) {  
      super(sim, position, radius);  
   }  
  
}`

The next line creates an single instance of PollenGrain:
`final PollenGrain grain = new PollenGrain(sim, new Vector3d(50, 50, 50), 1);`
We pass the BSim environment with which the grain will interact, a vector defining its initial position in 3D space, and a radius in μm. The grain is marked 'final' for technical reasons.

### The Ticker

The tick() method on the ticker is called each timestep (default \( \Delta t \) = 0.01 seconds) to update the properties of particles in the scene. In this instance, the action() method simply applies a Brownian force to the grain. The updatePosition() method then calculates the new position of the particle based on the net force.
`sim.setTicker(new BSimTicker() {  
  
   public void tick() {  
      grain.action();  
      grain.updatePosition();  
   }  
  
});`

### Drawers

The scene() method defined here is called each timestep to draw particles to the screen during graphical previews. Here we instruct the drawer to draw our pollen grain in yellow.
`sim.setDrawer(new BSimP3DDrawer(sim, 800, 600) {  
  
   public void scene(PGraphics3D p3d) {  
      draw(grain, Color.YELLOW);  
   }  
  
});`

### Exporters

BSim comes with exporters for outputting simulation data as movies, text files and images, and the extensible design means it is possible for users to add other exporters. The before() method of an exporter is called before the simulation starts; here we write a header to a spreadsheet. By default, the during() method is called each simulation timestep, though this can be changed. In this instance it writes the current time and the position of the grain.
`sim.addExporter(new BSimLogger(sim, "position.csv") {  
  
   public void before() {  
      super.before();  
      write("time, x, y, z");  
   }  
  
   public void during() {  
      write(sim.getFormattedTime() + ", "  
           + grain.getPosition().x + ", "  
           + grain.getPosition().y + ", "  
           + grain.getPosition().z + ", ");  
   }  
  
});`

### Starting the Simulation

BSim has two running modes: preview and export. The preview() method produces immediate and indefinite graphical output of the simulation, ignoring exporters. It is designed to allow users to assess the scene has been set up correctly before running export(). The export() method has no graphical output, but calls the exporters at each timestep.

With the last line of this example calling preview(), running the file BSimPollenExample.java will cause a window to pop up as shown below where we will observe a single, jiggling yellow blob. Changing the command to export() will write a file position.csv containing the position of the particle over time.


## Features

Having seen how to create a simple BSim, we proceed to outline the features on offer that allow users to create complex simulations. We present results to show that several fundamental mechanisms are working correctly, that is to say, the implementation produces results that match those in the literature or of established theory.

### Force-Based Dynamics

The simulation environment in BSim is a fluid with viscosity \( \mu \) and temperature \( T \) . A simple equation of motion for a particle in fluid is
\[
m \frac{d V}{dt} = -\lambda V + F
\]
where \( m \) is the particle mass, \( V \) is the particle velocity, \( \lambda \) is the coefficient of friction and \( F \) is the sum of external forces, not including the drag term \( -\lambda V \).

We assume that particles in BSim are small enough such that external forces equilibrate with the drag force near instantaneously . Hence at timestep i the velocity is calculated from the total external force as
\[
V\_i = \frac{F\_i}{\lambda}.
\]
We assume all particles are spherical so that the drag coefficient is
\[
\lambda = 6 \pi \mu a
\]
where \( \mu \) is the dynamic viscosity of the fluid and a is the particle radius. The position of the particle is updated according to
\[
X\_{i+1} = X\_i + \Delta X\_i
\]
with \( \Delta X\_i = V\_i \Delta t = \frac{\Delta t}{\lambda} F\_i \). The position of a particle starting at \( x\_0 \) after \( n \) timesteps is
\[
X\_n = x\_0 + \sum\_{i=0}^{n-1} \Delta X\_i.
\]
Forces are applied to particles using the addForce() function. As an example,
consider the brownianForce() method, called in BSimParticle#action():
`public void brownianForce() {  
   Vector3d f = new Vector3d(rng.nextGaussian(), rng.nextGaussian(), rng.nextGaussian());  
   f.scale(brownianForceMagnitude);  
   addForce(f);  
}`

#### Brownian Motion

As we saw above, Brownian motion is handled by including a Brownian force in the sum of external forces acting upon the particle. Each component of the force at the \( i \) th timestep is calculated according \( F\_i = q G\_i \) where \( G\_i \sim Norm(0, 1) \). The \( G\_i \) are independent and identically distributed with mean \( \langle G\_i \rangle = 0 \) and correlation \( \langle G\_i G\_j \rangle = \delta i j \).

We pick \( q \) such that a particle experiencing only a Brownian force has the appropriate displacement statistics \( \langle X\_n \rangle = x\_0 \) and \( \langle (X\_n - x\_0)^2 \rangle = 2 D t \) with diffusivity \( D = k\_B T / \lambda \) and \( t = n \Delta t \) [Berg 1993]. The moment \( \langle X\_n \rangle \) tells us nothing since
\[
\begin{align}
\langle X\_n \rangle &= x\_0 + \sum\_{i=0}^{n-1} \langle \Delta X\_i \rangle \\
&= x\_0 + \frac{q \Delta t}{\lambda} \sum\_{i=0}^{n-1} \langle G\_i \rangle \\
&= x\_0.
\end{align}
\]
However,
\[
\begin{align}
\langle (X\_n - x\_0)^2 \rangle &= \left( \frac{q \Delta t}{\lambda} \right)^2 \sum\_{i=0}^{n-1} \langle G\_i G\_j \rangle \\
&= \left( \frac{q \Delta t}{\lambda} \right)^2 n.
\end{align}
\]
Then to achieve \( \langle (X\_n - x\_0)^2 \rangle = 2 k\_B T n \Delta t / \lambda \) we should pick
\[
q = \sqrt{ \frac{2 k\_B T \lambda}{\Delta t} }.
\]
The following plot presents verification of this result. It shows traces of \( \langle (X\_n - x\_0)^2 \rangle \) against time in seconds for 100 particles with radius 20 nm in a fluid with \( T \) = 305 K, \( μ \) = 2.7 \( \times 10^{-3}\). The straight blue line is the theoretical result. This result has been derived by another means in [Ounis 1991].


### Boundary Types

If an update to one of a particle’s Cartesian coordinates causes the particle to stray beyond a solid boundary, the coordinate is updated again to place the particle an the same distance inside the boundary as it was outside. Wrapping boundaries cause the particle to reappear an same distance away from the opposing face of the box.

Boundaries wrap by default. This can be modified with a call to BSim#setSolid(). To have solid boundaries in the x dimension but wrapping ones in the y and z directions, call
`sim.setSolid(true, false, false);`

By overriding the methods BSimParticle#xAbove(), BSimParticle#xBelow() and so on, it is possible to define custom behaviour at the boundaries.

### Multiple Particles

To have multiple bacteria or other particles in the simulation, define a list (Vector) of bacteria as follows:
`final Vector bacteria = new Vector();  
 while(bacteria.size() < 100)  
   bacteria.add(new BSimBacterium(sim, new Vector3d());`
To create a uniform density of non-overlapping bacteria, we may write:
`while(bacteria.size() < 100) {  
   BSimBacterium b = new BSimBacterium(sim, new Vector3d(  
      Math.random()∗sim.getBound().x,  
      Math.random()∗sim.getBound().y,  
      Math.random()∗sim.getBound().z));  
   if(!b.intersection(bacteria)) bacteria.add(b);  
}`

The call to intersection() checks whether the particle is intersecting with any of the particles already in the list.

### Custom Particle Behaviours

To add a custom behaviour to a particle, extend the appropriate class and override the action() method:
`class CustomParticle extends BSimParticle {  
   public void action() {  
      super.action()  
      /∗ Perform additional behaviour ∗/  
   }  
}`

Calling super.action() instructs the CustomParticle to perform all of the actions its parent would (in this instance, just brownianForce()). This is exactly how extra functionality is added to the BSimBacterium class.

### Particle Interactions

Say we have defined two particle types, ParticleA and ParticleB, by extending BSimParticle, and have created lists aParticles and bParticles as above. To specify an interaction, define an interaction() function on one of the particle types:
`class ParticleA extends BSimParticle {  
   public void interaction(ParticleB b) {  
      /∗ Specify interaction ∗/  
   }  
}`
To apply the interaction, add the following code to the ticker:
`for(ParticleA a : aParticles)  
   for(ParticleB b : bParticles)  
      a.interaction(b);`
If the interaction modifies the positions of the particles, it should occur before updatePosition() is is called.

If we wanted every ParticleA to interact with every other ParticleA once per timestep, the loop in the ticker should read:
`for(int i = 1; i < aParticles.size(); i++)  
   for(int j = i+1; j < aParticles.size(); j++)  
      aParticles.get(i).interaction(aParticles.get(j));￼￼￼`

#### Collisions

Often we wish for particles to interact only when they collide. Collisions can be detected using the outerDistance() function, which returns the distance between particle edges:
`public void interaction(ParticleB b) {  
   if (outerDistance(b) < 0 )  
      /∗ Particles are colliding ∗/  
}`

##### Reaction Forces

A common response to a collision is a reaction force between particles. This can be handled with the reaction() method which applies a force of a specified magnitude towards each of the particles. For convenience, a method logReaction() is provided which exerts a force of magnitude
\[
F(d) = -k \mathrm{ln} \left( \frac{d}{a\_1 + a\_2} \right)
\]
where \( d \) is the distance between particle centres and \( a1 \), \( a2 \) are the particle radii. The magnitude of this force is zero when \( d = a1 + a2 \) (the particles are just touching) and tends to infinity as \( d \rightarrow 0 \). The parameter \( k \) controls the strength of the reaction force. For a particle exerting a internal force of magnitude \( f \), the minimum distance of approach to its collision partner is
\[
d = (a\_1 + a\_2) \mathrm{exp} \left( - \frac{f}{k} \right).
\]
That is, if the particle exerts a force 1 pN, then \( k \) = 1 will prevent it from coming closer than \( (a1 + a2 ) / e \).

##### Delivery

Another common type of collision is the delivery of a small particle (a molecule perhaps) to the collision partner (a bacterium). To achieve this effect, define the molecule class and a list of molecules to remove at each timestep:
`class Molecule extends BSimParticle {}  
final Vector moleculesToRemove = new Vector();`
Next define the bacterium that will accept the molecules:
`class AcceptingBacterium extends BSimBacterium {  
   public void interaction(Molecule m) {  
      if (outerDistance(m) < 0) {  
         /∗ Do something interesting ∗/  
         moleculesToRemove.add(m);  
      }  
   }  
}`
In the ticker:
`for (AcceptingBacterium b : acceptingBacteria) {  
   for (Molecule m : molecules)  
      b.interaction(m);  
   molecules.removeAll(moleculesToRemove);  
   moleculesToRemove.clear();  
}`
Things would be simpler if we could remove molecules from the list in b.interaction() directly. Unfortunately, it is not possible to remove objects from a list that is currently being looped over, hence the need for the additional moleculesToRemove list.

### Chemical Fields

A BSimChemicalField divides the simulation space into a number of boxes and allows a quantity of chemical \(Q\_{ijk} \) to exist in the box \( (i,j,k) \), specified as a number of molecules. The concentration of chemical in a box \( C\_{ijk} \) is found by dividing the quantity of chemical by the box volume. Chemicals diffuse between boxes according to a simple discretisation of Fick’s law \( J\_x = -D \frac{\partial C}{\partial x} \) where \( D \) is \( \partial x \) the field diffusivity. Chemicals decay according to \( Q\_{ijk}^{t = \Delta t} = ( 1 - \chi \Delta t) Q\_{ijk}^{t} \) so that \( \chi \) is the fraction of chemical decaying per second. Since we are considering quantities in molecules, an alternative interpretation of \( \chi \) is the probability per unit time for a molecule to decay.

Chemical fields are created similarly to particles:
`final BSimChemicalField field = new BSimChemicalField(sim, boxes, diffusivity, decayRate);`

The number of boxes in the x, y and z directions is specified by passing a three element array of integers, boxes. The field’s diffusivity is specified in μm2/s. Finally, the decayRate is specified in s-1.

At this point, no chemical exists in the field. A linear gradient in the z direction may be created using the function linearZ(). Quantities of chemical in individual boxes can be manipulated using the functions setConc() and addQuantity(). In addition, setConc() can be used to set the concentration of every box in the field to a particular value.

To allow the field to diffuse and decay, we must update the field in the ticker:
`public void tick() {  
   field.update();  
}`
To see the field, add a line to the drawer:
`public void scene(PGraphics3D p3d) {  
   draw(field, Color.BLUE, alphaGrad);  
}`
The argument alphaGrad specifies the alpha (transparency) of the field per unit concentration (the maximum value of alpha is 255).

### Bacteria

#### Motility

Bacterial motility in BSim is based on study of the common intestinal organism *Escherichia coli* [Berg 1972]. We seek a model that replicates the results for the AW405 wild type strain on a statistical level.

The motion appears as an alternating sequence of 'runs', where the cell moves through the medium, and 'tumbles', where the cell almost stops. Changes in direction from the end of one run to the beginning of the next are dramatic and have a particular distribution. Changes in direction during runs are gradual; the drift is about what one would expect from rotational diffusion. The distribution of run (tumble) durations is approximately exponential, indicating that the probability per unit time for a run (tumble) to end is constant.

We translate these observations into the action() method for the BSimBacterium class as below:
`public void action() {  
   super.action();  
  
   switch (motionState) {  
   case RUNNING:  
      if (Math.random() < pEndRun()∗sim.getDt())  
         motionState = MotionState.TUMBLING;  
      break;  
   case TUMBLING:  
      if (Math.random() < pEndTumble()∗sim.getDt()) {  
         /∗ Change the direction at the end of a tumble phase ∗/  
         BSimUtils.rotatePerp(direction, tumbleAngle());  
         motionState = MotionState.RUNNING;  
      }  
      break;  
   default:  
      assert false : motionState;  
   }  
  
   if (motionState == MotionState.RUNNING) {  
      rotationalDiffusion();  
      flagellarForce();  
   }  
   ...  
}`

The following plot shows that the motion of a bacterium in BSim appears qualitatively the same as an AW405 bacterium (BSim results are shown on left and literature results on right).

Furthermore the following plots show that the distributions of tumble angles and run/tumble durations do in fact match that of the AW405 bacteria (BSim results are shown on left and literature results on right).


#### Rotational Diffusion

The method rotationalDiffusion() rotates the direction of the particle at the timestep \( i \), \( d\_i \), by an angle \( \Delta \theta\_i = p G\_i \) where \( G\_i \sim Norm(0,1) \) as before.

Similar to Brownian motion, we try to pick \( p \) such that a particle experiencing only rotational diffusion has the appropriate variance \( \langle \theta^2 \rangle = 4 D\_r t \) with rotational diffusivity \( D\_r = k\_B T / \lambda\_r \) and rotational Stokes’ coefficient \( \lambda\_r = 8 \pi \mu a^3 \) [Berg 1993].

Define \( \rho( x, \delta) \) as the vector obtained by rotating the vector \( x \) by an angle \( \delta \), and \( \Theta (x,y) \) as the angle between the vectors \( x \) and \( y \). Then although we can obtain an expression for \( \theta\_1 \) directly in terms of \( \Delta \theta\_0 \),
\[
\begin{align}
\theta\_1 &= \Theta (d\_1, d\_0)\\
&= \Theta (\rho (d\_0, \Delta \theta\_0), d\_0)\\
&= \Delta \theta\_0
\end{align}
\]
we cannot obtain \( \theta\_{i>1} \) directly in terms of the \( \Delta \theta\_i \) :
\[
\begin{align}
\theta\_2 &= \Theta (d\_2, d\_0)\\
&= \Theta (\rho (d\_1, \Delta \theta\_1), d\_0)\\
&= \Theta (\rho (\rho (d\_0, \Delta \theta\_0), \Delta \theta\_1), d\_0)
\end{align}
\]
Hence we cannot match \( \langle \theta\_{n}^{2} \rangle = 4 D\_r t \) in the way we matched \( \langle (X\_n - x\_0)^2 \rangle = 2 D t \). Nevertheless we can simply set \( \langle \theta\_{1}^{2} \rangle = \langle \Delta \theta\_{0}^{2} = p^2 = 4 D\_r \Delta t \) so
\[
p = \sqrt{4 D\_r \Delta t}.
\]
In practice this seems to work well.

#### Chemotaxis

The distributions of run and tumble durations are unaffected the presence of an isotropic concentration of aspartate. In the presence of aspartate gradient, however, the distribution of runs when the bacterium is moving up the gradient shifts towards longer run durations. This indicates that the probability per unit time for a run to end is decreased when the cell realises it is moving up an aspartate gradient, and results in bacteria moving up aspartate gradients on the average in a so-called 'biased random walk'. These observations determine the pEndRun() method:
`public double pEndRun() {  
   if (goal != null && movingUpGradient())  
      return pEndRunUp;  
   else  
      return pEndRunElse;  
}`
The goal field (asparate) is a BSimChemicalField set via bacterium.setGoal().

How does a cell decide it is moving up an aspartate gradient? A later study is consistent with the explanation that the cell compares the mean concentration experienced over the last second, with the mean concentration experienced over the 3 seconds before that [Segall 1986]. Hence the method movingUpGradient() reads:
`public boolean movingUpGradient() {  
   return shortTermMean() − longTermMean() > sensitivity;  
}`

The durations over which the cell compares the mean concentrations can be set with the methods setShortTermMemoryDuration() and setLongTermMemoryDuration().

### Growth

BSim features a basic model of bacterial growth where bacteria grow according to a surfaceAreaGrowthRate parameter [Reshes 2008]. The grow() method is called as part of the BSimBacterium#action() method after the motility block.

#### Replication

Bacteria replicate when reaching a threshold radius. Unfortunately, we have the same problem as in particle delivery where we cannot modify a list that is actively being looped over. Hence we must create a list of 'child' bacteria that is processed after the loop in tick():
`final Vector bacteria = new Vector();  
final Vector children = new Vector();  
  
while(bacteria.size() < 10) {  
   BSimBacterium b = new BSimBacterium(sim, new Vector3d());   
   b.setGrowthRate(1);  
   b.setChildList(children);  
   bacteria.add(b);  
}  
  
sim.setTicker(new BSimTicker() {  
   public void tick() {  
      for (BSimBacterium b : bacteria) {  
         b.action();  
         b.updatePosition();  
      }  
      bacteria.addAll(children);  
      children.clear();  
   }  
});`

#### Vesiculation

According to [Deatherage 2009], membrane vesicle (MV) production is not due to random membrane instability, but rather is the result of essential processes of cell growth and division. The paper presents a model of MV biogenesis where localised envelope remodelling induces the release of small MVs at regions of lower-density membrane-peptidoglycan connections.

The following figure illustrates a model of MV biogenesis, due to [Deatherage 2009]. (left) Localised envelope remodelling induces release of small MVs at regions of lower-density OM-PG connections along cell body. (right) Larger MVs are released during division due to temporary disruption of septal OM-PG-IM complexes. MV, membrane vesicle; OM, outer membrane; PG, peptidoglycan; IM, inner membrane.

We model MV release as a Poisson interval process with a certain probability per vesicle surface area growth of a bacteria releasing a vesicle \( p\_v \). In [Gankema 1980] the authors found that "medium vesicles... accounted for 3 to 5% of the total cellular outer membrane", hence reasonable values for \( p\_v \) are around 0.05.

### ODE Solvers

BSim comes with several Ordinary Differential Equation (ODE) solvers that can be used to model gene regulatory networks and other internal biochemical processes. The solvers are defined in the BSimOdeSolver class and objects that need to use ODE dynamics must implement the methods defined in the BSimOdeSystem interface:
`class Rossler extends BSimParticle {  
  
   protected RosslerOdeSystem odeSys;  
   protected double[] y, yNew;  
  
   public Rossler(BSim sim, Vector3d position){  
      super(sim, position);  
      odeSys = new RosslerOdeSystem();  
      y = odeSys.getICs();  
   }  
  
   public void action() {  
      super.action();  
      yNew = BSimOdeSolver.rungeKutta45(odeSys, sim.getTime(), y, sim.getDt());  
      y = yNew;  
   }  
  
   class RosslerOdeSystem implements BSimOdeSystem {  
  
      public double[] derivativeSystem(double x, double[] y) {  
         double[] dy = new double[3];  
         dy[0] = -y[1] - y[2];  
         dy[1] = y[0] + 0.2 * y[1];  
         dy[2] = 0.2 + y[2] * (y[0] - 5.7);  
         return dy;  
      }  
  
      public double[] getICs() {  
         double[] ics = new double[3];  
         ics[0] = 1.0;  
         ics[1] = 3.5;  
         ics[2] = 7.3;  
         return ics;  
      }  
  
      public int getNumEq() {  
         return 3;  
      }  
   }  
}`
Here we define a Rossler particle that contains a RosslerOdeSystem (Rossler chaotic attractor) that is solved as the action() method is called during a simulation.

### DDE Solvers

Many complex biological processes involve many steps that can lead to large systems of ODEs being necessary to describe their dynamics. In some cases, these steps can be simplified (incorporated into) to a delay that allows for a reduced set of equations defining the dynamics of a system. This system is then defined using Delayed Differential Equations (DDEs).

BSim provides a set of DDE solvers in the BSimDdeSolver class and any object that needs to use DDE dynamics must implement the methods defined in the BSimDdeSystem interface. These are similar to that for ODE systems, but also allow for historic (delayed) states to be accessed using the BSimDdeSolver.getDelayedState() method.

### Meshes

Complex environments can be defined using meshes within BSim. To load a mesh from file (in Wavefront .obj format) we use the BSimOBJMesh object and the load() method:
`final BSimOBJMesh theMesh = new BSimOBJMesh();  
theMesh.load("MyMesh.obj");`
The BSimOBJMesh imports the mesh in the same coordinate space as the mesh was generated. Sometimes this will need to be scaled and translated. This can be performed using the scale() and translateAbsolute() methods:
`theMesh.scale(4400);  
theMesh.translateAbsolute(new Vector3d(4000,4000,4000));`

#### Mesh Collisions

For meshes to define physical barriers within an environment it is necessary to handle collisions between particles and the mesh. This is carried out using the BSimCollision.collideAndRepel() method and placed within the action() method of any particles that should detect collisions:
`public void action() {  
   super.action();  
   BSimCollision.collideAndRepel(this, theMesh);  
}`

#### Spatial Varying Parameters

Sometimes meshes may not define physical barriers, but instead regions in which different model parameters are experienced by particles. For example, the location where a light input is being shone. To allow for particles to detect transitions from one parameter regime to another we can use the BSimCollision.collideAndCross() method in the updatePosition() method of a particle:
`public void updatePosition() {  
   super.updatePosition();  
   if (BSimCollision.collideAndCross(oldPos, position, theMesh))  
      inside = !inside;  
}`
In this case, the inside boolean flag must be correctly initialised based on the particle starting inside or outside the mesh, and then crossings will result in a flipping of the state. Variables oldPos and position are the position of the particle in the previous and current timestep (to be defined and managed by the programmer).

## References

[**Berg 1972**] H.C. Berg and D.A. Brown. Chemotaxis in *Escherichia coli* analysed by three-dimensional tracking. *Nature*, **239**:500-504, 1972. Link  
  
[**Berg 1993**] H.C. Berg. Random walks in biology. Princeton University Press, 1993. Link  
  
[**Deatherage 2009**] B.L. Deatherage, J.C. Lara, T. Bergsbaken, S.L.R. Barrett, S. Lara, and B.T. Cookson. Biogenesis of bacterial membrane vesicles. *Molecular Microbiology*, **72**(6):1395-1407, 2009. Link  
  
[**Gankema 1980**] H. Gankema, J. Wensink, P.A.M. Guinee, W.H. Jansen, and B. Witholt. Some characteristics of the outer membrane material released by growing enterotoxigenic *Escherichia coli*. *Infection and Immunity*, **29**(2):704–713, 1980. Link  
  
[**Ounis 1991**] H. Ounis, G. Ahmadi, and JB McLaughlin. Brownian diffusion of submicrometer particles in the viscous sublayer. *Journal of Colloid and Interface Science*, **143**(1):266–277, 1991. Link  
  
[**Reshes 2008**] G. Reshes, S. Vanounou, I. Fishov, and M. Feingold. Cell shape dynamics in *Escherichia coli*. *Biophysical Journal*, **94**(1):251–264, 2008. Link  
  
[**Segall 1986**] J.E. Segall, S.M. Block, and H.C. Berg. Temporal comparisons in bacterial chemotaxis. *Proceedings of the National Academy of Sciences*, **83**(23):8987– 8991, 1986. Link
