# Supplementary material for: BSim: An Agent-Based Tool for Modeling Bacterial Populations in Systems and Synthetic Biology
Source: PLoS One. 2012 Aug 24;7(8):e42790. doi: 10.1371/journal.pone.0042790 (PMC3427305; doi:10.1371/journal.pone.0042790)
Supplement: Software S1 — Snapshot of the BSim software from 18th July 2012. For the latest version see: http://bsim-bccs.sf.net. The BSim software requires Java version 1.6 or higher. (ZIP) [file pone.0042790.s014.zip › BSimSoftware/libs/deps/LICENSE.html]

BSim Dependancies


# BSim Dependancies

BSim depends on several external libraries to provide additional functionality. The following section outlines these dependancies and the licensing of these libraries.

## Processing

http://www.processing.org

The Processing library is used to carry out the rendering of the three-dimensional visualisations and assist in the export of animated videos. The library is released under the GNU Lesser General Public License (LGPL).

## OBJ\_IMPORT

http://www.pixelnerve.com/processing/libraries/objimport/

This library is written by Fabien Sanglard and used to read mesh files in the Wavefront .obj format. The library is released under the GNU Lesser General Public License (LGPL).

## QuickTimeWriter

http://www.randelshofer.ch/blog/2010/10/writing-quicktime-movies-in-pure-java/

This library is written by Werner Randelshofer and used to export simulations to video files. The library is released under the Creative Commons Attribution 3.0 license.

## Java3D

http://java3d.java.net/

BSim only uses the vecmath part of this library to carry out vector operations. The library is released under the GNU General Public License (GPL), version 2, with the CLASSPATH exception.
