# Supplementary material for: BSim: An Agent-Based Tool for Modeling Bacterial Populations in Systems and Synthetic Biology
Source: PLoS One. 2012 Aug 24;7(8):e42790. doi: 10.1371/journal.pone.0042790 (PMC3427305; doi:10.1371/journal.pone.0042790)
Supplement: Software S1 — Snapshot of the BSim software from 18th July 2012. For the latest version see: http://bsim-bccs.sf.net. The BSim software requires Java version 1.6 or higher. (ZIP) [file pone.0042790.s014.zip › BSimSoftware/README.html]

BSim: An Agent-Based Tool for Modeling Bacterial Populations in Systems and Synthetic Biology


# An Agent-Based Tool for Modeling Bacterial Populations in Systems and Synthetic Biology

Thomas E Gorochowski^1,\*^, Antoni Matyjaszkiewicz^1^, Thomas Todd^1^, Neeraj Oak^1^, Kira Kowalska^2^, Stephen Reid^1^, Krasimira T Tsaneva-Atanasova^2^, Nigel J Savery^3^, Claire S Grierson^4^, and Mario di Bernardo^2,5^.

^1^ Bristol Centre for Complexity Sciences, Department of Engineering Mathematics, University of Bristol, Bristol, UK.  
^2^ Department of Engineering Mathematics, University of Bristol, Bristol, UK.  
^3^ School of Biochemistry, Medical Sciences, University Walk, Bristol, UK.  
^4^ School of Biological Sciences, University of Bristol, Bristol, UK.  
^5^ Department of Systems and Computer Engineering, University of Naples Federico II, Via Claudio 21, 80125, Napoli, Italy.  
^\*^ E-mail: thomas.gorochowski@bristol.ac.uk

## Introduction

BSim is an agent-based modelling tool designed to allow for the study of bacterial populations in systems and synthetic biology. By enabling the description of bacterial behaviours, it attempts to provide an environment in which to investigate how local interactions between individual bacterium leads to the emergence of population level features, such as cooperation and synchronisation. This is possible through the incorporation of physical processes such as bacterial movement, interactions and chemical diffusion.

This document and the additional supporting files provide information about getting started with the BSim library as well as access to to the source code and compiled binaries that can be used to create new models. Simulations presented in the paper are also included to allow for reproduction of the reported results.

It should be noted that this package contains BSim version 3.0. Due to the continual development of the software, for latest news and versions of the source code see the project website at: http://bsim-bccs.sf.net.

## Source Code and Binaries

Source code for BSim version 3.0 is provided within the src directory. This code requires Java version 1.6 or higher is installed and configured correctly. Due to a number of dependancies on external libraries (found in the directory libs/deps) we recommend using a development environment such as Eclipse to compile the code.

To reduce the effort required when developing with BSim, we also provide a pre-compiled version of the library. This is recommended for use by end-users and can be found in the libs directory. In addition, we also include other libraries that BSim depends on within the libs/deps directory. For information about the licensing of these libraries see the LICENSE.html file in this directory. The pre-compiled bsim3.0.jar file does not require these additional files as they have been incorporated into the release. However, using the source code directly will require the inclusion of these dependancies.

Using the pre-compiled BSim library, to compile a simulation contained within the mySim.java file, the following command should be used:
 `javac -cp bsim.3.0.jar mySim.java` 
This assumes that the bsim3.0.jar file is within the current directory.

## Documentation

BSim provides a user guide to help end-users get started in producing simulations and more detailed developer documentation that describes the function of classes and methods in the library. User the following links to see the specific documentation:

- **User Guide**
- **Developer Documentation**

## Simulations

To allow for reproduction of the results presented in the paper, we have included the source code of the simulations that we developed and instructions for how these can be run. These can be found within the sims directory and include README.html files to help getting started. Use the following links to find out more about a particular simulation:

- **Multicellular Computing Model**
- **Population Dynamics of the *lac* Operon Model**
- **Synchronized Genetic Oscillators Model**

In addition, a wide range of example simulations that are not presented in the paper, but which help to show the broad range of available features can be found within the examples directory.
