# Supplementary material for: BSim: An Agent-Based Tool for Modeling Bacterial Populations in Systems and Synthetic Biology
Source: PLoS One. 2012 Aug 24;7(8):e42790. doi: 10.1371/journal.pone.0042790 (PMC3427305; doi:10.1371/journal.pone.0042790)
Supplement: Software S1 — Snapshot of the BSim software from 18th July 2012. For the latest version see: http://bsim-bccs.sf.net. The BSim software requires Java version 1.6 or higher. (ZIP) [file pone.0042790.s014.zip › BSimSoftware/sims/computing/README.html]

Multicellular Computing Model


# Multicellular Computing Model

## Introduction

The multicellular computing model is found within the sims/computing directory and simulates the system described in Tamsir et al. "Robust multicellular computing using genetically encoded NOR gates and chemical wires." Nature 469:212-215 (2011). Specific details of the model are found in the Supporting Text S1.

The model consists of several classes that define the environment and placement of bacterial populations and the specific types of bacteria that can be used:

- BSimLogic.java - Defines the BSim model including the setup of bacterial populations, the chemical fields that are used, and the export of data to files.
- BSimLogicBacterium.java - Base class for all types of bacteria. This contains no function and instead is extended by the following classes.
- BSimBufferBacterium.java - Buffer bacterium that takes an input chemical field and uses that to activate an output reporter.
- BSimNORBacterium.java - NOR bacterium that takes two input chemical fields and calculates the NOR logical operation of these, outputting the result to a third chemical field.
- BSimNOTBacterium.java - NOT bacterium that takes a single input chemical field and calculates the NOT logical operation of this, outputting the result to a second chemical field.
- BSimORBacterium.java - OR bacterium that takes two input chemical fields and calculates the OR logical operation of these, outputting the result to a third chemical field.

## Running the Simulation

To reproduce the results found in the main text, the BSimLogic.java must be correctly configured. In particular, there are three main sections of this file that define the length of the simulation and whether data is exported, the state of the input chemical fields (ON or OFF), and the position and type of the bacterial populations. Comments are included in the code to assist with editing of particular features.

Lines 44 and 45 define whether the data should be exported or if a preview of the simulation should be displayed instead, as well as the total simulation time:
 `exportData = false;  
sim.setSimulationTime(3600);` 
By default the simulations display a visual preview and have a simulation running time of 1 hour (3600 seconds).

Lines 68 and 69 define the state of the input chemical fields:
 `AraOn = false;  
aTcOn = false;` 
By alternating these states each of the different boolean input combinations can be assessed.

Lines 109 to 166 define the bacterial populations calculating their position, realistic delays, and generating the correct bacterium objects to make up the populations. For example, the following code generates the second population of NOR bacteria that use the Ara and LasI chemical fields as inputs, and output their result to the Rhll chemical field:
 `while(bacteria2.size() < popSize) {  
   BSimNORBacterium p = new BSimNORBacterium(sim,  
      getNextBacteriumPos(sim, p2x, p2y, 5.0, popSpread, popSpread),  
      fAra, 30115,  
      fLasI, 30115,  
      fRhll, 2675,  
      getDecayTime(prodDly, prodDlySpread),  
      getDecayTime(repDly, repDlySpread));  
   bacteria2.add(p);  
}` 
By altering the types of bacteria that are produced and the delays, input, and output chemical fields all the different types of logic circuit in the paper can be reproduced.

To compile and run the simulation we provide the scripts compile\_and\_run.sh for UNIX, Linux and Mac OS X, and compile\_and\_run.cmd for Windows. These assume that Java version 1.6 or higher has been installed and configured correctly. These should be run directly from the sims/computing directory.

### Example: EQUALS Circuit

To illustrate this for a single type of circuit, by default the model is setup to implement the EQUALS logic function (see Figure 2 in the main text of the paper). As shown above the two chemical field inputs are set to OFF and a visual preview is displayed. Compiling and running the code will see a similar output to that seen in Figure 2. Due to the number of bacteria, the drawing of the display may be slow leading to jumps in the displayed time. However, if viewed for long enough the same features as shown in the figure will be displayed.

To export the data that the simulation is generating, it is necessary to update the BSimLogic.java file in the BSimLogic directory. Specifically, line 44 should be updated to:
 `exportData = false;` 
This will turn off preview mode and export the data to CSV files. Compiling and running the simulation now will result in a new folder being created called results in which another folder is generated with a time stamp containing files with output data for each of the populations (Population1.csv, Population2.csv, Population3.csv, Population4.csv). Furthermore, to generate the graphs shown in the Supporting Text S1 it is also necessary to increase the simulation running time to 10 hours (36000 seconds). Therefore, line 45 becomes:
 `sim.setSimulationTime(36000);` 
The time series are then calculated by dividing the nRepON column of each population data file by 20000, to give the proportion of the population that is activated (ON). Moreover, altering the input chemical field states on lines 68 and 69, it is possible to generate all the time series shown in Figure S3 of the Supporting Text S1. The other logic circuits can be generated by altering the layout and type of bacterial populations in lines 109 to 166.
