# Supplementary material for: BSim: An Agent-Based Tool for Modeling Bacterial Populations in Systems and Synthetic Biology
Source: PLoS One. 2012 Aug 24;7(8):e42790. doi: 10.1371/journal.pone.0042790 (PMC3427305; doi:10.1371/journal.pone.0042790)
Supplement: Software S1 — Snapshot of the BSim software from 18th July 2012. For the latest version see: http://bsim-bccs.sf.net. The BSim software requires Java version 1.6 or higher. (ZIP) [file pone.0042790.s014.zip › BSimSoftware/sims/lac/README.html]

Population Dynamics of the lac Operon Model


# Population Dynamics of the lac Operon Model

## Introduction

The lac operon model is located within the sims/sync directory. It simulates individual and population behaviour of the lac operon. We implement the model described in Chung J, Stephanopoulos G "On physiological multiplicity and population heterogeneity of biological systems", Chemical Engineering Science 51:1509-1521. Further details of the implementation can be found in our paper and the Supporting Text S1.

The model consists of two classes that define the simulation (environment and placement of bacterial populations) and a custum model of a bacterium with an ODE based model of lac operon behaviour embedded inside:

- BSimLacOperon.java - Defines the BSim model including the setup of bacterial populations, the chemical fields that are used, and the export of data to files.
- BSimLacBacterium.java - extension of a BSimBacterium with an embedded system of ODEs describing the model of lac outlined in Chung and Stephanopoulos.

## Running the Simulation

To reproduce the results found in the main text, the BSimLacOperon.java must be correctly configured. Firstly, at lines 38 to 45 it is possible to specify parameters that can be set through the command line when starting the simulation. This was added to ease scripting multiple runs of the simulation, and by default allows the user to set an output director and the simulation length in seconds as the first and second parameters respectively. Line 51 specifies the concentration of the external chemical field which influences behaviour of the ODEs in the bacteria. Line 52 can be altered to allow the chemical field concentration to vary (in a ramped fashion) over time, and bydefault is set to remain at a static value.

By setting line 62
 `boolean exportData = true;` 
the simulation can be set up to export data to .csv files.

Lines 94 to 121 set up the bacterial populations. The value of populationLimit configured on line 94 will set the stable population limit, the number of bacteria present in the simulation.

If it is desired to allow the chemical field to ramp up and down, then this can be configured at lines 141 to 181. Specifically, lines 160 to 165 are used to specify the gradient over time, and maximum concentration of the chemical field as follows:
 `int t_ramp_up_1 = 3600;  
int t_plateau = 42000;  
int t_ramp_down = 14400;  
double chemical_1 = 125;` 
The variables t\_ramp\_up\_1 t\_plateau and t\_ramp\_down specify the duration in seconds that the chemical field spends increasing, at a steady state, and decreasing respectively. chemical\_1 specifies the maximum concentration during the plateau period.

Further simulation parameters specific to the bacteria themselves must be configured in the file BSimLacBacterium.java. Lines 68 to 71 allow the user to specify the initial conditions of the lac GRN, and whether the bacterial growth rate is affected by lac induction:
 `grn.RANDOM_ICS = true;  
grn.POPULATION_VARIANCE = true;  
INDUCTION_AFFECTS_GROWTH = false;`

Lines 102 and 103 specify the average time in seconds over which new bacteria grow or die. Lines 195 to 297 describe the lac GRN behaviour as a system of ODEs. Specifically, ODE parameters as defined in Chung and Stephanopoulos are defined at lines 230 to 237.

By altering the parameters describing how bacteria are affected by the lac operon, the results presented in our paper can be reproduced. To compile and run the simulation we provide the scripts compile\_and\_run.sh for UNIX, Linux and Mac OS X, and compile\_and\_run.cmd for Windows. These assume that Java version 1.6 or higher has been installed and configured correctly. These should be run directly from the sims/computing directory. To pass command line arguments to the simulation, the required parameter values should be passed on execution i.e., added to the scripts at line 2. However this is not necessary to run the simulation as it stands, since default values can be automatically generated within the simulation file.

### Default settings

By default the simulation will not output any results and will instead run in a window showing the bacteria with internal concentrations of lax inducer indicated by their color, and the chemical field concentration rendered around them. The simulation is set to run for 60000 seconds with an external chemical field that ramps up to a concentration of 125 units over a period of 3600 seconds, remains at this value for 42000 seconds, then ramps down to zero over 14400 seconds. The bacteria start with random initial conditions in the GRN, at both induced and uninduced states. Induction is set to not affect growth.

These default values should be sufficient to reproduce results akin to those shown in Figure 5(c). The internal inducer concentration in the bacteria increases gradually with time (remaining un-induced) until the external concentration reaches a threshold. At this point the bacteria move to their induced state, and there is a sudden jump in internal lac inducer concentration. This then decreases slowly as the external concentration is ramped down once more to zero.

By enabling the export mode (line 62) as detailed above, the simulation can be set to produce four files with simulation output:

- Settings.csv - some general simulation statistics.
- internalLactose.csv - internal lactose inducer concentration for all bacteria.
- internalPermease.csv - internal lac permease concentration for all bacteria.
- Hysteresis.csv - general time varying statistics: time; external inducer concentration; average, minimum, and maximum internal inducer concentrations; proportion of bacteria that are induced; total bacterial population.

Results like those illustrated in Figure 5(c) can be reproduced purely from Hysteresis.csv, by plotting the average, minimum and maximum concentrations against the external inducer concentration. Generated results may not correspond exactly to Figure 5(c) due to the stochastic nature of the simulation but should be extremely similar at a high enough population density (in fact the default value of 500 is sufficient). The hysteresis curve (red) was produced using a numerical continuation software (matcont), and is not part of the data generated by BSim.
