# Supplementary material for: BSim: An Agent-Based Tool for Modeling Bacterial Populations in Systems and Synthetic Biology
Source: PLoS One. 2012 Aug 24;7(8):e42790. doi: 10.1371/journal.pone.0042790 (PMC3427305; doi:10.1371/journal.pone.0042790)
Supplement: Software S1 — Snapshot of the BSim software from 18th July 2012. For the latest version see: http://bsim-bccs.sf.net. The BSim software requires Java version 1.6 or higher. (ZIP) [file pone.0042790.s014.zip › BSimSoftware/sims/sync/README.html]

Synchronized Genetic Oscillators Model


# Synchronized Genetic Oscillators Model

## Introduction

The synchronized repressilators model is located in the sims/sync directory, and simulates the system described in Garcia-Ojalvo et al. "Modelling a synthetic multicellular clock: Repressilators coupled by quorum sensing", PNAS 101(30):10955-10960 (2004). Specific details of the model as implemented in BSim are found in the Supporting Text S1.

The model consists of a single class:

- BSimCoupledRepressilators.java - Defines the model as a BSim simulation, including the creation of chemical fields and bacterial populations. Contains an inner class BSimRepressilatorBacterium, described below, in which bacterial behaviour is defined.

The inner class BSimRepressilatorBacterium (line 73) extends a prototypical BSimBacterium with the addition of a repressilator genetic regulatory network. The GRN is defined as a set of ODEs in the QuorumRepressilator extension of a BSimODESystem (line 128).

## Running the Simulation

To reproduce the results found in the main text, the BSimCoupledRepressilators.java must be correctly configured. Specifically, the parameters of the chemical field must be varied, as well as the parameters controlling the behaviour of the GRN. Additionally the simulation can be set to either preview results graphically or export them to a .csv file in the results subdirectory. Throughout the code, comments have been provided to describe how to alter the functionality of the simulation.

By default the simulations display a visual preview and have a simulation running time of 100 minutes (line 51):
 `sim.setSimulationTime(6000); // sim. time in seconds`

Lines 60 to 63 define the behavioural parameters for the extracellular autoinducer concentration, represented by a chemical field:
 `final double diffusivity = 100;   
final double decayRate = 0.01/60;   
final double cellWallDiffusivity = 2.0;` 
By changing these values, the rate at which the chemical moves and decays in space, and the rate at which it is removed from the field object when a bacterium is in the vicinity, can be adjusted.

Line 64 defines whether the initial conditions of the ODE system should be uniform, ICS\_UNIFORM, or random, ICS\_RANDOM.

Lines 73 to 199 define the BSimRepressilatorBacterium, where lines 128 to 198 define the GRN inside each bacterium. Lines 138 to 143 define the ODE system parameters, and have a direct correspondence to the parameter names used in "Modelling a synthetic multicellular clock: Repressilators coupled by quorum sensing".

The results presented in our paper can be reproduced by running the simulation multiple times while varying the GRN and chemical field parameters.

Line 353 sets whether the simulation should run in export or preview mode. When set to export mode, the simulation will output general simulation statistics, and the concentrations of lacI and internal autoinducer for all bacteria at specified time intervals.
 `sim.preview();` 
or
 `sim.export();`

To compile and run the simulation we provide the scripts compile\_and\_run.sh for UNIX, Linux and Mac OS X, and compile\_and\_run.cmd for Windows. These assume that Java version 1.6 or higher has been installed and configured correctly. These should be run directly from the sims/lac directory.

### Example: synchronization from random initial conditions

By default the model is set to run such that each bacterium in the population has a randomised set of initial conditions created for its internal GRN. Compiling and running the code will result in the unsynchronised bacterial population visible on the screen, swimming within a blue-colored chemical field representing the autoinducer. The oscillations of the internal concentrations of lacI mRNA (represented by the color of bacteria) will eventually synchronize. Due to the number of bacteria, the drawing of the display may be slow leading to jumps in the displayed time. However, if viewed for long enough the color fluctuations of the bacteria will be seen to synchronize.

To export the data that the simulation is generating, it is necessary to update the BSimCoupledRepressilators.java file at line 353, as mentioned above.
This will turn off preview mode and export the data to .csv files. Compiling and running the simulation now will result in a new folder being created called results in which another folder is generated with a time stamp containing files with output data. The time series of the internal lacI concentration for all bacteria over time can be directly plotted from the lacI\_ALL.csv file. The bacteria may need a longer period of time to synchronise depending on the simulation parameters, in which case the simulation time can be adjusted to, for example, 10 hours:
 `sim.setSimulationTime(36000);`
